# Effectiveness of the sterile insect technique in controlling *Aedes albopictus* as part of an integrated control measure: evidence from a first small-scale field trial in Switzerland

**DOI:** 10.1186/s40249-025-01360-2

**Published:** 2025-08-22

**Authors:** Diego Parrondo Monton, Damiana Ravasi, Valentina Campana, Francesco Pace, Arianna Puggioli, Matteo Tanadini, Eleonora Flacio

**Affiliations:** 1 Institute of Microbiology, Department for Environment Constructions and Design, University of Applied Sciences and Arts of Southern Switzerland (SUPSI), Via Flora Ruchat-Roncati 15, 6850 Mendrisio, Switzerland; 2 Centro Agricoltura Ambiente “G. Nicoli”, Via Sant’Agata 835, 40014 Crevalcore, Italy; 3 Zurich Data Scientists GmbH, Sihlquai 131, 8005 Zurich, Switzerland

**Keywords:** Integrated vector management, Mosquito control, Sterile insect technique, *Aedesalbopictus*, Urban environment, Vector-borne diseases, Spatiotemporal variability, Population dynamics

## Abstract

**Background:**

The invasive Asian tiger mosquito (*Aedes albopictus*) poses growing health risks across Europe. In Switzerland, a preliminary field trial was conducted to assess the feasibility of integrating the sterile insect technique (SIT) into existing integrated vector management (IVM), which includes breeding site removal and application of biological larvicides. SIT involves repeated releases of irradiated sterile males, which mate with wild females, producing non-viable eggs and leading to population decline.

**Methods:**

Following a preliminary release test in 2022, a small-scale SIT trial took place in 2023 in Morcote, Switzerland. Approximately 150,000 sterile males were released weekly over a 45-hectare area throughout the entire mosquito activity season, from May to September. This SIT area also received routine IVM. Population dynamics were compared with a control area where only IVM was applied. Monitoring included egg counts, hatch rates, and adult female densities. Generalized additive mixed-effects models (GAMM) and generalized additive models (GAM) accounted for spatial, temporal, and random effects. Model selection used AIC, BIC, and Chi-square tests (significance at 5%).

**Results:**

The SIT-treated area showed a significant mosquito population reduction. Egg counts dropped by 57% (GAMM regression coefficient: − 0.8513, *P* < 0.001), with temporal patterns differing between SIT-treated and control areas (*P* < 0.001). Egg hatch rates were also lower in the SIT area, with odds of hatching reduced by 1.24 log-odds units (*P* < 0.001). Adult female densities declined by 66% (regression coefficient: − 1.0818, *P* < 0.001). Spatial GAMs revealed heterogeneous effects: up to 90% egg reduction in the western release area, while the eastern edge, bordering untreated zones, showed up to 300% higher egg counts. Similar spatial trends were observed for hatch rates and adult females (*P* < 0.01). These findings highlight both the overall effectiveness of SIT and the influence of mosquito immigration on spatial patterns.

**Conclusions:**

This trial demonstrated the potential of SIT as a complementary tool in Swiss vector control. Public interest and acceptance were high. To improve cost-effectiveness, further optimization of male production, sterilization, transport, and release processes is needed. Continued implementation over multiple seasons is recommended to enhance long-term effectiveness.

**Graphical Abstract:**

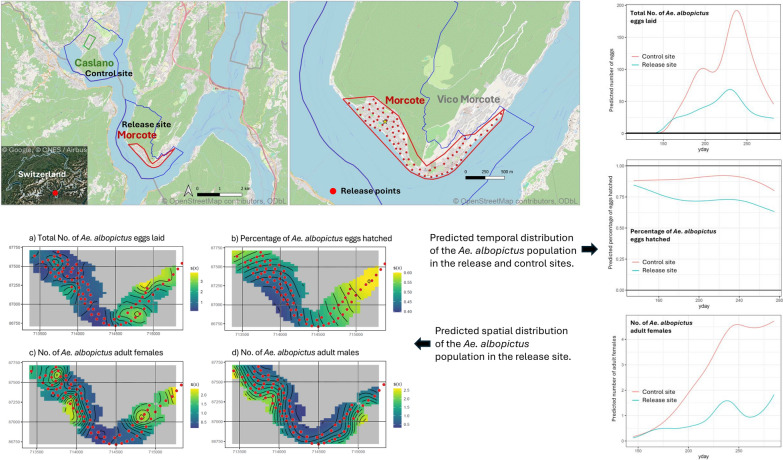

**Supplementary Information:**

The online version contains supplementary material available at 10.1186/s40249-025-01360-2.

## Background

The tiger mosquito (*Aedes albopictus*, Skuse, subgenus: *Stegomyia*; Diptera: Culicidae) is a competent vector for several arboviruses of significant public health concern, including dengue, chikungunya, Zika, and yellow fever [1–3]. While these viruses have historically been prevalent in tropical and subtropical regions, their frequency and the scale of outbreaks are increasing globally. Indeed, in 2023, the World Health Organization (WHO) warned that dengue rates are rising worldwide, with approximately half of the global population now at risk [4]. In Europe, both dengue and chikungunya are considered emerging threats [5].

Since prophylactic drugs and/or vaccines are often unavailable, vector control is crucial for preventing and managing the transmission of these and other mosquito-borne pathogens. However, the widespread use of chemical biocides in traditional control programs has led to resistance in target vector populations, as well as environmental and health concerns [5, 6]. Furthermore, the global resurgence of *Aedes*-borne disease outbreaks highlights the limitations of traditional control methods and the need for an integrated approach that combines multiple strategies [7]. Integrated vector management (IVM) is a rational decision-making framework that promotes the optimal use of resources for efficient, cost-effective, and sustainable vector control [8].

In Switzerland, an IVM program has been active for over 20 years on the southern side of the Alps, where *Ae. albopictus* is established in most urban areas [9, 10]. Here, the IVM control measures primarily focus on the aquatic phase of mosquitoes with removal of breeding sites and application of microbial larvicides in public spaces. Private spaces are targeted through extensive information campaigns, such as community education events, door-to-door distribution of educational materials (leaflets), school programs, and mass media outreach. Citizens are strongly encouraged to eliminate temporary water containers from private properties and either cover or treat permanent water containers with obtainable larvicides. The use of adulticides, which are not ecologically sustainable, is reserved for extraordinary situations when an imported disease case is confirmed, to prevent local outbreaks [11].

The IVM program has proven effective in controlling *Ae. albopictus* populations and reducing human nuisance [12, 13]. However, estimated population densities remain above the threshold considered to pose an epidemic risk in the event of arbovirus introduction [12]. Moreover, the effectiveness of the control measures is believed to have reached a limit, due in part to the presence of cryptic or inaccessible breeding sites in both public and private spaces, lack of control in specific locations (such as construction sites, secondary homes, and abandoned properties), and challenges in keeping public engagement alive over time. Consequently, there is still a need to improve control methods to overcome these limitations.

The sterile insect technique (SIT) represents a potentially sustainable solution. The technique consists of producing large numbers of male mosquitoes, irradiating them to induce sterility, and releasing them in a target area to mate with wild females. Wild females mated with sterile males produce eggs that do not hatch, which leads, over time and with successive releases, to a reduction in the target mosquito population [14, 15]. So, this technique does not rely, as the control methods already used, on the detection and removal or treatment of breeding sites, but rather on the large numbers of sterile males released that disperse across the target area and intercept wild females [15]. SIT also provides the advantage of being, specifically targeted, spatially constrained, not persistent in the environment, and not generating resistance [16, 17]. Additionally, SIT appears to be most effective at low population densities and is ideally suited to complement other control measures, such as source reduction, that are already part of an IVM approach [18–22].

Here, we evaluated the potential for integrating SIT into the existing vector control program in southern Switzerland to further reduce the presence of *Ae. albopictus*. Following the phased conditional approach outlined by WHO and the International Atomic Energy Agency (IAEA) [23], in 2022 we conducted a preliminary release of sterile *Ae. albopictus* males at an urban site using the mark release and recapture (MRR) method. The primary objectives of this test were to assess the dispersal and survival of the released males to determine the optimal number of release stations, their spacing, and the frequency of releases. We evaluated the feasibility of the transport and release of sterile males, to gain field and laboratory experience, and to refine experimental parameters. Additionally, we monitored public and media responses to the implementation of an SIT release program. In 2023, as the next phase of the conditional approach, we conducted a small-scale field trial based on established experimental designs and three further MRR studies [19, 22–24]. Periodic releases of sterile *Ae. albopictus* males were carried out throughout the entire mosquito breeding season within a target area of 45 hectares. Populations of *Ae. albopictus* were monitored at both the release site and a control site to evaluate the effectiveness of the SIT intervention.

## Methods

### Study sites

**Fig. 1:**
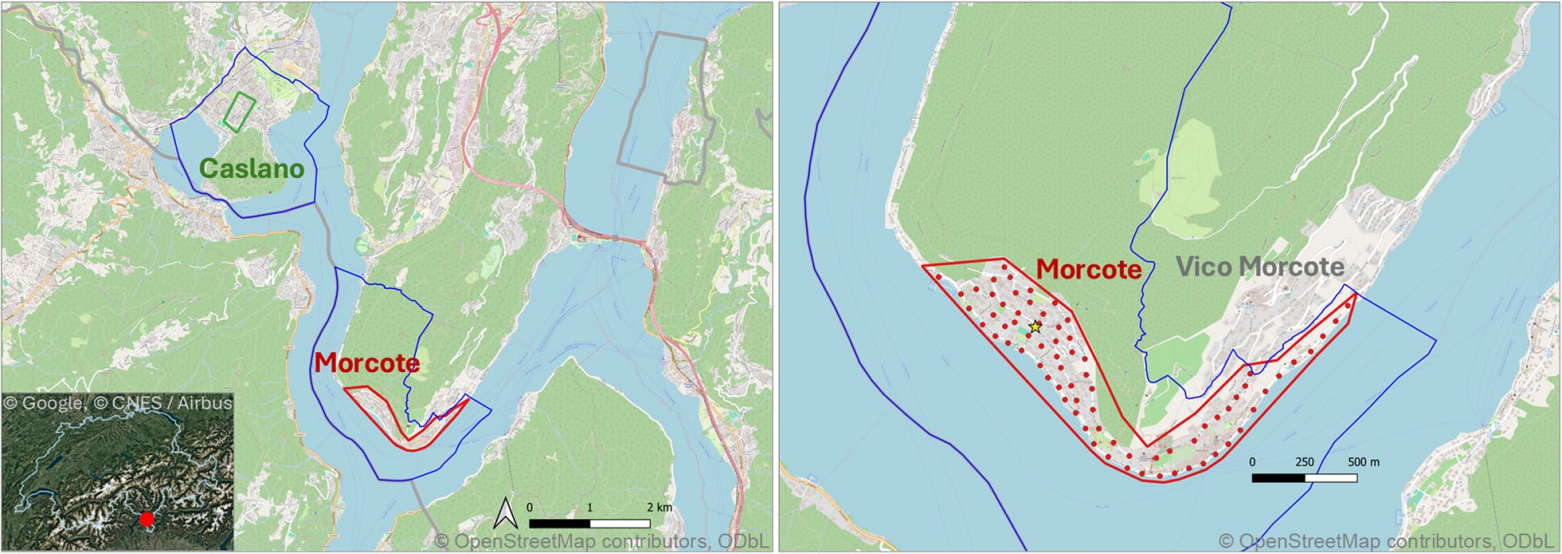
Release (red) and control (green) areas in southern Switzerland. The boundaries of the study municipalities, Morcote and Caslano, are outlined in blue. On the right, the yellow star marks the release point for the 2022 and 2023 MRR, while the red points indicate the 75 release stations for the 2023 SIT trial. MRR: Mark release and recapture; SIT: Sterile insect technique

The release trials were conducted in Morcote (45° 55′ 30″ N 8° 54′ 58″ E, 292 m a.s.l.), a small town in southern Switzerland (Fig. 1). A permit to release sterile male tiger mosquitoes in Morcote (BAFU-217.23–64633/7) was obtained from the Swiss Federal Office for the Environment in accordance with the Ordinance on the Handling of Organisms in the Environment (No. 814.911). Geographically, the main urban area of Morcote is divided into two parts: the eastern part, which includes the historic center of the village and a more residential area that borders the nearby town of Vico Morcote to the northeast, and the western part, which consists solely of residential areas, with a significant proportion (over 20%) of secondary residences [25]. The release area, approximately 45 hectares, covered the entire urban area and was therefore almost entirely isolated from other towns except for the northeastern side bordering Vico Morcote. The control site, of about 14 hectares, was set in Caslano (45° 58′ 16″ N 8° 52′ 59″ E, 278 m a.s.l., 50 ha), a small town about 5.5 km straight-line distance from Morcote, with similar geographical and housing characteristics (Fig. 1).

Both towns follow an IVM since 2000 [9, 13]. In 2022 and 2023, the integrated control measures consisted in the regular treatment of catch basins in public spaces with the larvicide VectoMax® FG (Valent BioSciences Corp., Libertyville, IL, USA), applied every 6 weeks [26]. Citizens are encouraged to remove temporary water containers from their properties and to cover or treat permanent ones with the larvicide VectoBac® G (Valent Biosciences Corp., Libertyville, IL, USA). During 2021, before starting the sterile male releases, no significant differences were found in the average number of eggs collected per ovitrap in the SIT and control area (Fig. S1) although the data include another invasive *Aedes* species (i.e., *Ae. japonicus*) in addition to *Ae. albopictus*.

### Mass rearing of *Aedes albopictus*

Sterile *Ae. albopictus* males were bred at the Centro Agricoltura Ambiente “G. Nicoli” (CAA) production facility in Italy, approximately 250 km from the release site, using methods outlined in [27]. The mosquito colony used for the SIT trials was initiated from eggs collected in Ticino in 2019 (strain CH) and maintained under standard conditions of 28 ± 1 °C, 85% relative humidity, and a photoperiod of 14∶10 h (L∶D). Mosquito larvae were mass-reared following the IAEA protocols [28, 29].

### Preliminary mark release and recapture (MRR) study

The release of sterile, marked *Ae. albopictus* males took place on August 2nd, 2022, at the peak of the tiger mosquito reproductive season in southern Switzerland. At the CAA production facility, male pupae (strain CH F11) aged 20–48 h were separated from female pupae (with a residual female presence below 1%, considered acceptable in areas non endemic for arboviruses [23, 30, 31]) using an automatic version of the Fay-Morlan glass plate separator (Guangzhou Wolbaki Biotech Co., Ltd, Guangzhou, China) [32, 33] and irradiated with 55 Gy in water using a Radgil2 X-Ray irradiator (Gilardoni Ltd., Mandello del Lario, Italy). After irradiation and emergence, 22,500 sterile males were cold shock-anaesthetized at 8 ± 1 °C for 30 min and marked with a fluorescent pink powder according to the Food and Agriculture Organization of the United Nations (FAO) and IAEA 2017 protocol [34]. The males were then transferred to 15 plastic cups (MONOUSO ENVALIA GROUP S.L., Valencia, Spain; 10 cm diameter, 5 cm height, 350 cc capacity; 1500 individuals per cup), which were stacked into a cardboard cylinder. The cylinder was placed inside a polystyrene container, and the temperature was maintained at 10–12 °C using phase change materials (PCM ice gel packs; Blu Ice, Green Ice, Dryce Srl, Milano, Italy).

The polystyrene box was placed in a cardboard box and transported by car for 3 h to the receiving laboratory. Upon arrival, the marked sterile males were immediately transferred to the designated release station in Morcote (Fig. 1) and released at 18:00 local time. The sterility level of released males was checked through a residual fertility test at the receiving laboratory (Method S1) and their quality was evaluated by assessing the mean mortality rate after transportation, based on the number of males that did not fly out of a sub-sample of four containers within 1 h at the release station (males that were not yet awake were counted as dead).

From August 3rd to 9th, human landing collections (HLC) were conducted every 24 h at 32 evenly distributed points within a 200-m radius of the release station (Method S2, Fig. S2). The mosquitoes collected were brought to the laboratory, euthanized, and examined using a stereomicroscope. The species, sex, and presence of marked sterile males was recorded. Several parameters were estimated based on the data recorded, including male recapture rate (ϴ), male daily survival rate (s), average life expectancy, and mean dispersal distance [35–37].

### Small-scale SIT field trial

In 2023, the releases of sterile males were conducted throughout the entire mosquito season, from early May to the end of September, at 75 fixed stations covering the entire release area (Fig. 1). Based on the results of the 2022 MRR, the release stations were evenly distributed at distances of less than 90 m from one another (range: 50–80 m). Approximately 2000 sterile males were released per station each week (3000 sterile males per hectare), for a total of about 150,000 males released each week. The releases were carried out manually by two operators, who moved between the stations by car. On average, the release process took about 3 h. In the first 8 and last 2 weeks of the season, releases were carried out once a week (Table S1). During the hottest part of the season (from July to mid-September), sterile males were released twice a week to maintain a higher sterile-to-wild ratio and ensure their continuous activity throughout the week. In this case, instead of a single weekly release of 150,000 males, two releases of 75,000 males each were carried out. In total, approximately 3,300,000 males were released over 22 weeks in the release area.

#### Production and transportation of sterile males

At CAA, male pupae aged 2–48 h (CH F13-F16) were irradiated with 40 Gy in water using a Radgil2 X-Ray irradiator (Gilardoni Ltd., Mandello del Lario, Italy). The males were packaged similarly to the 2022 protocol, with 2000 individuals per cup for the first four releases, and 1000 individuals per cup for all subsequent releases (Table S1). At each release station, the cups were placed in suitable locations where microclimatic conditions were favorable for their awakening and takeoff and where the ants would not reach.

For most of the study period (until week 26), males were shipped via courier, with transportation time to the receiving laboratory averaging approximately 18 h. In week 27, we transitioned to direct transportation by car or train, reducing delivery time to approximately 3 h (Table S1). Sterile males were 2–4 days old at the time of release and were marked only for MRR events.

Residual fertility of the sterile males was assessed three times during the release season (May 3, June 27, and August 8, 2023) at the receiving laboratory, using the same method as in 2022 (see methods in Additional file 1). Mortality following transportation was assessed at each release by selecting five randomly chosen release stations. The residual presence of females in the cups was assessed when MRRs were carried out.

#### MRR studies

Three MRR tests were conducted in 2023: one at the beginning of the season (June 13th, 10,000 males), one in the middle (July 11th, 20,000 males), and one at the end of the season (August 22nd, 20,000 males). After emergence, the sterile males were powdered-marked following the same protocol as in 2022, packed in stacked plastic cups (1000 males per cup), and shipped by courier to the receiving laboratory. The marked males were released at the same location as in 2022 (Fig. 1) and recaptured at the same 32 spots as in 2022 (Fig. S2), but this time with adult traps (BG-Pro, Biogents, Regensburg, Germany, sentinel mode, baited with CO_2_) instead of HLC. Several studies have shown that HLC and adult trap catches are comparable [38]. Adult traps offer the advantage of covering the entire day and do not depend on operator attractiveness, as is the case with HLC. The traps were checked daily for a week or until marked males were no longer captured. The same parameters as in 2022 were estimated.

#### Entomological monitoring to assess the effectiveness of SIT application

The population of *Ae. albopictus* was monitored at both the release and control sites through three entomological parameters: the total number of *Ae. albopictus* eggs laid, the percentage of *Ae. albopictus* eggs that hatched, and the number of *Ae. albopictus* adult females. Eggs were collected using oviposition traps (ovitraps), which consisted of black 1.5-L plastic jars (Ramona Ø13/H12, Luwasa® Interhydro AG, Allmendingen, Switzerland) with a top-border efflux hole. Each jar contained 1.2 L of tap water and a wooden slat (20 × 2.5 × 0.5 cm) that served as a substrate for female *Aedes* mosquitoes to lay their eggs [9, 10]. Forty-five and 14 ovitraps (one ovitrap per hectare) were set in the release and control site, respectively (Fig. S3). The wooden slats were collected and replaced with new ones once a week from mid-April until mid-October. In the laboratory, the eggs of *Ae. albopictus* were differentiated from other mosquito species and counted using established methods [39].

Egg hatching rates were assessed every 2 weeks. After counting the *Ae. albopictus* eggs, the wooden slats were subjected to a hatching protocol [34, 37]. The number of hatched eggs was counted, and egg hatching rate was calculated as the number of hatched eggs divided by the total number of eggs on the slats.

Adult mosquitoes were collected using CO_2_-baited BG-Pro traps (sentinel mode) evenly distributed (one trap every two hectares) across the release (25 traps) and control (8 traps) sites. The traps were set every 2 weeks, starting from the beginning of May until the end of September, 24 h after each release, and were checked after 40 to 48 h. *Aedes albopictus* mosquitoes were identified and counted by sex.

#### Statistical analyses

A generalized additive mixed-effects model (GAMM) was used to compare mosquito population parameters of the release and control sites across the season. Seasonal trends in these parameters were highly non-linear, with a peak in summer. To accurately capture these patterns, smoothers were applied within the GAMM framework. The analysis for the total number of *Ae. albopictus* eggs laid, the percentage of *Ae. albopictus* eggs that hatched, and the number of *Ae. albopictus* adult females is detailed in Supplementary file 2, Supplementary file 3, and Supplementary file 4, respectively. Briefly, the response variable was modelled using a generalised additive model (GAM) with a negative binomial (total number of *Ae. albopictus* eggs laid, number of *Ae. albopictus* adult females) or quasi binomial (percentage of hatched *Ae. albopictus* eggs) family to address overdispersion. The model included a smooth effect for “sampling date”, represented as the day of the year (yday, a numeric variable), which interacts with the variable municipality. The municipality variable is categorical and consists of two levels: Morcote (release site) and Caslano (control site); its effect was modelled as a fixed effect. To account for variations in trap exposure duration we incorporated Activation.time (a count variable ranging from 6 to 16 days) as an offset (we used the log of Activation.time to make the assumption that doubling the activation time would lead to proportional doubling in the number of eggs) in the model. We controlled for the non-independence of observations by including trap ID (unique.ID, a categorical variable) as a random effect. Model complexity was evaluated, and the best-fitting model was selected using a Chi-square test and Akaike information criterion (AIC) and Bayesian information criterion (BIC) criteria. All statistical analyses were conducted using R (version 4.4.2) [40]. The significance level was set at 5%.

A spatial GAM was used to test whether geographical location played a role in determining the three entomological parameters. In particular, this model estimated a two-dimensional spatial abundance surface to better understand the treatment’s effectiveness and how its impact diminishes toward the edges of the treated area. Two separate models were fitted to analyse the response variable, using a GAM with a negative binomial family to account for overdispersion. One model was fitted for Morcote (release site), and the other for Caslano (control site). Both models included a smooth effect for the “sampling date”, represented as day of the year (yday), and a combined smooth effect for geographic coordinates to capture spatial variability within each site. The best-fitting models were selected by evaluating AIC and BIC criteria. All statistical analyses were conducted using R (version 4.4.2) [40]. The significance level was set at 5%.

#### Public awareness

The IVM plan, followed in both release and control area, includes already several activities for communication to citizens: the distribution of leaflets on tiger mosquito and how to fight it to all households; dedicated web pages (www.supsi.ch/go/zanzare and www.ti.ch/zanzare); a service of personal counselling by phone or by email; specific spots “Let’s take away the water” and the video “Let’s fight the tiger mosquito together” broadcasted on national television during the breeding season and viewable on many municipal websites; active communication with the media, especially at the beginning of the breeding season and at the end of July, before the seasonal peak; and punctual educational activities in schools and other contexts. To raise public awareness about the SIT trial, the media were invited to the release of sterile males, both in 2022 and 2023, and several interviews were broadcast both in the press and on television the following days. A public presentation was conducted in June 2023 in the municipality of Morcote to inform the community and solicit their support.

## Results

On 2 August 2022, 22,500 marked sterile *Ae. albopictus* males were transported by car from the CAA production facility, in Italy, to the release site in Switzerland for the preliminary MRR study. The mortality rate of mosquitoes at the release site after the 3-h journey was 10.7%. The recapture rate (ϴ) by HLC was 0.21%, the daily survival rate (s) was estimated at 0.59, and the average life expectancy was 1.9 days (Table S2). Most of the recaptured males (83.1%) were within 100 m of the release point (Table S3). The mean distance travelled by the sterile males was 91 m, with a maximum distance of 175 m. The residual fertility of sterile males, tested at the receiving laboratory, was 1.5%, which is consistent with other studies [41]. For comparison, the fertility of non-irradiated males was 87.6%.

In 2023, sterile males of *Ae. albopictus* were released between May and September at weekly or biweekly intervals for a total of 34 release events. The average residual fertility of sterile males was 0.03 ± 0.06% (1.5% in 2022 due to different conditions of pupae during irradiation [42]). For comparison, the average fertility of non-irradiated males was 96.4 ± 1.8%. The average residual presence of females at the time of release was estimated at 0.3 ± 0.06%.

At the beginning of the release season in May, the mean mortality of sterile males at release was quite high (53.7 ± 6.7%) (Table S1) due to the low field temperatures in May, which prevented the males from quickly waking up and leaving the transport cups. From June to the end of August, the mortality rate decreased to 20.3 ± 7.6%, due to higher field temperatures and the reduction in mosquito density during transportation (which was halved from 2000 to 1000 individuals per cup). During this period, the males were shipped by courier, with transportation time to the receiving laboratory being approximately 18 h. In one instance, the package arrived with a 2-day delay. In week 27, we switched to direct transportation by car or train, reducing delivery time to 3 h. The mortality rate in the following period decreased to 10.1 ± 5.3%.

Regarding the results of the three MRR conducted in 2023, the overall mean (± *SD*) mortality rate observed following the release of sterile marked males after the 3-h journey was 40.2% (± 10%) (Table S2). This rate was higher compared to 2022 (10.7%). In comparison, the mortality rates reported in other studies ranged from 4.9 to 15.9% [35, 36]. The mean recapture rate (ϴ) of marked sterile males was 0.61% (± 0.84%), therefore higher than 2022 (0.21%). This range, despite the variability observed across time periods, fall within the typical range reported in MRR studies [35, 36]. The mean daily survival rate (s) was estimated at 0.63 (± 0.24) not much different from 2022 (0.59) and from previous MRR studies [35, 36]. The mean average life expectancy was 3.5 (± 3.3) days (1.9 days in 2022). In northern Italy, Balestrino et al. [35] observed an average life expectancy of 3.5 to 4.7 days. Since the life expectancy is half a week, it is recommended for sterile males to be released twice per week. As in 2022, most of the recaptured males (79.6%) were within 100 m of the release point (Table S3). The mean distance travelled by the sterile males was 97 (± 43.4) m (91 m in 2022), with a maximum distance of 184 m (175 m in 2022). Most of the marked males (91%) were caught from 24 to 72 h after their release. Their presence decreased considerably after the third day: from 72 to 96 h (day 4) we recaptured only 6.3% of them. After 96 h from the release, the presence of marked males was residual (5%).

We evaluated the impact of SIT on the temporal distribution of three population parameters (total number of *Ae. albopictus* eggs laid, percentage of *Ae. albopictus* eggs that hatched, and number of *Ae. albopictus* adult females). When looking at the seasonal patterns at the level of ovitraps or adult traps, we observed that in the release area the variability within a single ovitrap (Supplementary file 2, pages 9–10) or adult trap (Supplementary file 3, pages 9–10) was more pronounced compared to the control area. For example, looking at the total number of eggs, in the lower panel of the graphs, a sudden drop from 100 eggs in 1 week to zero the following week can be observed (Supplementary file 2, page 9).

**Fig. 2:**
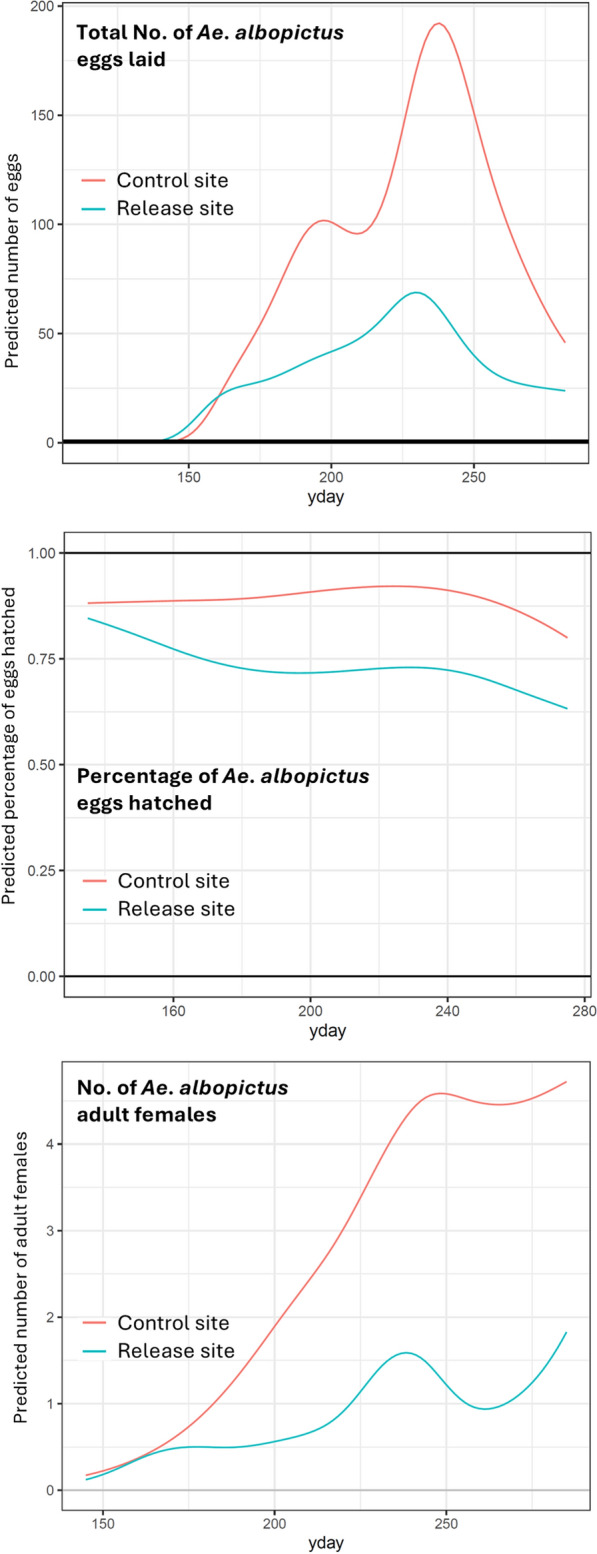
Predicted temporal distribution of the *Aedes albopictus* population in the release and control sites. yday: Day of the year

The models (i.e., GAMM) converged without issues and showed good fits (Table 1; Supplementary file 2, Supplementary file 3, Supplementary file 4). The estimated number of eggs followed the typical seasonal pattern at both study sites; however, in the release site, the numbers were much lower and more stable throughout the season compared to the control site (Fig. 2). Specifically, the release site showed an expected average reduction in egg counts of 57% compared to the control site (regression coefficient: − 0.8513, *P* < 0.001) after adjusting for the smooth seasonal effects specific to each site and including a random effect for each ovitrap (Supplementary file 2, page 12). Additionally, the temporal dynamics of the response variable differed significantly between the two sites (Chi-square test, *P* < 0.001). Both AIC and BIC criteria supported this result (AIC: 10,788.44 vs 10,819.23; BIC: 11,206.02 vs 11,209.66, Supplementary file 2, page 23). At the peak of the season, between mid and late August, the number of eggs in the control site (192) was estimated to be about three times higher than in the release site (69).

**Table 1:** Deviance explained by the models used to evaluate the impact of SIT on *Aedes albopictus* population parameters in the release and control sites

	GAMM	Spatial GAM	
		Release site (%)	Control site (%)
Total No. of *Ae. albopictus* eggs laid	53.3%	49.6	55.9
Percentage of *Ae. albopictus* eggs hatched	35%	10.8	16.1
Total No. of *Ae. albopictus* adult females	60.1%	47.3	62.3
Total No. of *Ae. albopictus* adult males	NA	31.5	53.3

*GAMM* Generalized additive mixed-effects model, *GAM* Generalized additive model, *NA* Not available, *SIT* Sterile insect technique

The estimated percentage of hatched eggs remained relatively stable across the season (Fig. 2), with only a slight increase during the peak period. The release site consistently had a lower hatching rate than the control site. In fact, the odds of an egg hatching in the release site were significantly lower compared to the control site (regression coefficient in log-odds: − 1.2354, *P* < 0.001), while accounting for trap-level random effects (Supplementary file 3, page 26). In this case, there was no evidence that it was necessary to allow different smooth shapes for the effect of the day of the year on the log-odds of an egg hatching for each site (Chi-square test, *P* = 0.2312; Supplementary file 3, page 27). To account for overdispersion, a quasi-binomial family was used; consequently, AIC and BIC could not be computed due to the absence of a true likelihood function. In the control site, the proportion of hatched eggs varied between 85 and 92% throughout the season, while in the release site, it ranged between 81 and 87% early in the season, dropping to between 69 and 75% later on. The average percentage of hatched eggs was predicted to be 85.07% in the control site and 69.04% in the release site. Interestingly, the variability in the hatching percentage was quite stable in the control site, while it was much more significant in the release site.

The number of adult females followed the same seasonal trend as the number of eggs (Fig. 2). Towards the end of the season, rather than decreasing as expected, the numbers increased again towards a peak. Indeed, at the end of the season, the model was predicting values outside the range of actual observations, making predictions for that period less reliable. In the release site, the number of adult females was consistently lower than in the control site. Specifically, the release site showed an expected average reduction in female counts of 66% compared to the control site (regression coefficient: − 1.0818, *P* < 0.001), after adjusting for trap-level random effects (Supplementary file 4, page 22). Again, there was no evidence that allowing different smooth shapes for the effect of the day of the year on the response for each site was necessary (Chi-square test, *P* = 0.1395; Supplementary file 4, page 23). AIC and BIC were disagreeing on the need of a smoother for each site (AIC: 971.9955 vs. 972.1002; BIC: 1123.925 vs 1111.913), therefore the most parsimonious model was chosen (Supplementary file 4, page 23). The estimated presence of adult females in the release site was 36.9% of that observed in the control site.

**Fig. 3:**
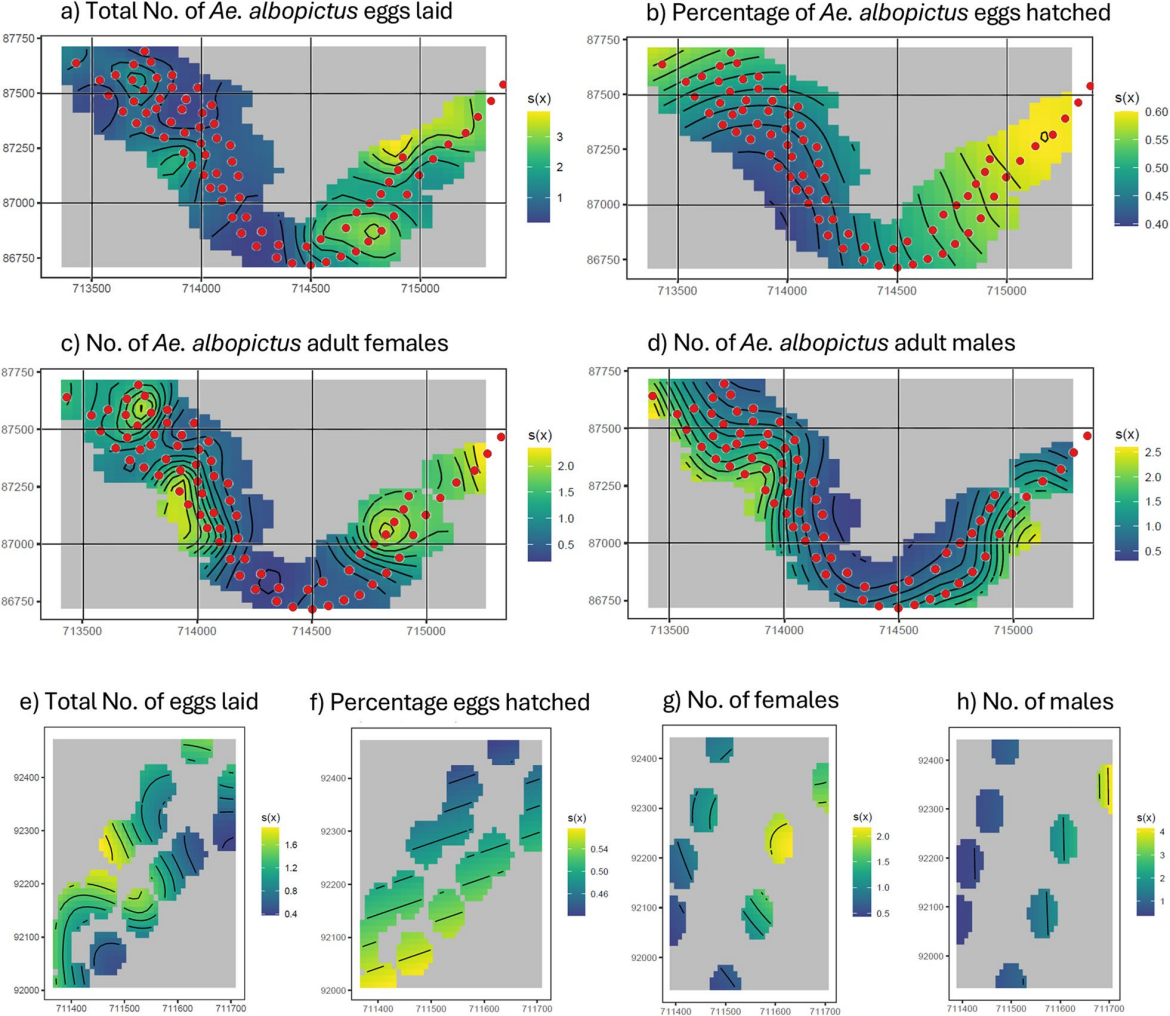
Predicted spatial distribution of the *Aedes albopictus* population in the release (**a**, **b**, **c**, **d**) and control (**e**, **f**, **g**, **h**) sites. The red points indicate the release stations. The x- and y-axes represent geographic coordinates in the CH1903 / LV03 coordinate system

We then evaluated the impact of SIT on the geographical distribution of the three population parameters. Each variable was modelled separately across the season and territory by incorporating geographical coordinates (Supplementary file 2, Supplementary file 3, Supplementary file 4). The models (i.e., spatial GAM) converged without issues and showed good fits (Table 1), and the results were visualized through a spatial GAM plot. We can conceptualize this plot as a surface, similar to a map, where the ‘contour lines’ represent the values of the smoother rather than the terrain itself. These lines indicate how much the value of the smoother deviates from the average, reflecting, for example, the relative abundance of the predicted total number of laid *Ae. albopictus* eggs (Fig. 3a). The greater the change in colour, the larger the variation in the smoother’s value, ultimately forming a surface with peaks and valleys. Consider dividing the area into small squares (similar to pixels). For each square, the value represents the number of ‘extra eggs’ relative to the average number of eggs in that area. This value corresponds to the smoother at that location. By ‘average’, we refer to the mean number of eggs across all squares within the specific area. For example, in regions where the smoother is approximately 1, the number of eggs is equal to the average. In contrast, areas where the smoother reaches values of 2 or 3 indicate that the number of eggs is twice or three times the average, respectively. The average value refers to the mean of all observed values used to fit the model. Figure 3a shows how the predicted total number of laid *Ae. albopictus* eggs varied across space in the release site. Note that the scale in the graph is multiplicative, meaning that each step represents a multiplication of the previous value (unlike a linear scale, where the distance between values is constant). Therefore, the number of laid eggs varied from one-tenth of the average to almost four times the average. In percentage terms, this means a range from a decrease of 90% (deep blue) to an increase of 300% (yellow) compared to the average (100%). The ranges of the scales varied according to the model’s predictions for a particular population parameter and study site.

The impact of the SIT on the total number of *Ae. albopictus* eggs in the release site was quite clear, with a strong spatial pattern (edf = 24.051, *P* < 0.001, Supplementary file 2, page 41; Fig. 3a). In the eastern part of the release site the estimates of egg numbers were higher compared to the western part. In fact, on the eastern side, the number of eggs increased to nearly four times the amount observed on the western part. In the control site, the ovitrap density was insufficient to make predictions for the entire surface (Fig. 3e). It was however possible to distinguish that spatial variation was much less pronounced compared to the release site (edf = 9.009, *P* < 0.001, Supplementary file 2, page 43), reaching only about twice as many eggs as the average. This might also be because the control site was smaller (30%) than the release site.

As for the estimated number of hatched *Ae. albopictus* eggs, the hatching rate was higher on the eastern part of the release site compared to the western part (edf = 5.409, *P* < 0.01, Supplementary file 3, page 42; Fig. 3b). The contour lines of the graph were smoother compared to those for the number of eggs because there were fewer observations for egg hatching. In the control site, aside from the insufficient ovitrap density, the spatial variation in egg hatching was lower (edf = 2, *P* = 0.20144, Supplementary file 3, page 43) than in the release site (Fig. 3f).

The estimated number of *Ae. albopictus* females showed a notable variability across the release site (from 0.5 to 2 times the number of females; edf = 17.077, *P* < 0.001, Supplementary file 4, page 39). Certain zones had a high number of females, while others had very low counts (Fig. 3c). The higher number of females on the eastern side corresponds to the results for the total number of eggs laid and the hatching rate. However, the distribution of adult females also concentrated in two spots in the western part, which was different from what was observed for the total number of eggs laid and hatching rates. Interestingly, the distribution of *Ae. albopictus* males was similar to that of females (Fig. 3d). In the control site, the range of spatial variation for adult females was similar to the release site (edf = 4.698, *P* < 0.001, Supplementary file 4, page 40; Fig. 3g), with limited variability except for one point in the north-east where estimates were higher. Adult males were also observed in the north-east area (Fig. 3h).

## Discussion

The aim of the study was to evaluate the efficacy of integrating SIT into the existing vector control program in southern Switzerland to further reduce the presence of *Ae. albopictus*. After conducting a preliminary release test in 2022, a small-scale field trial was carried out in 2023. Sterile males were released over a 45-ha target area, at 75 release points, throughout the entire tiger mosquito activity season from early May to late September. Overall, approximately 150,000 sterile males were released weekly, for a total of about 3.3 million sterile males released. The population dynamics of the tiger mosquito in the area treated with SIT were compared to those in a control area. Mosquito populations were monitored through three main parameters: number of *Ae. albopictus* eggs laid, hatching percentage of *Ae. albopictus* eggs, and number of *Ae. albopictus* adult females. The number of adult females is a critical parameter, as they act as disease vectors by biting humans, posing a significant health risk.

Previous studies indicate that tiger mosquito population densities in southern Switzerland remain low, thanks to the ongoing IVM program [13], but still sufficient to cause a potential risk of transmission of exotic infectious agents [12]. Indeed, most positive ovitraps (76%) at the release site carried fewer than 50 eggs and the rest of positive ovitraps carried between 50 and 250 eggs. At the control site, 55% of the positive ovitraps carried fewer than 50 eggs, 42% between 50 and 250 eggs and 3% between 250 and 305 eggs. In comparison, the number of *Ae. albopictus* eggs in neighbouring Italian municipalities without defined control measures is almost four times higher [13].

Furthermore, an additional reduction in the mosquito population was observed in the SIT trial area compared to the control area. During the seasonal peak of the tiger mosquito, between mid and late August, the estimated number of *Ae. albopictus* eggs in the release site was almost one third of that in the control site, indicating a reduction of 64%. Moreover, about 19% fewer *Ae. albopictus* eggs were likely to hatch and 63% fewer *Ae. albopictus* adult females were present at the release site. In particular, the reduction in the density of adult females is a critical factor for health risk [12]. The reduction in the presence of females was also indirectly confirmed by the perception of the community, which reported a reduction in the number of bites.

Our results are consistent with those reported in previous SIT trials against *Ae. albopictus*, although the differences in the methods used limit direct comparison and require caution in interpreting the observed similarities. In a SIT trial conducted in Spain, a reduction in *Ae. albopictus* egg density of 74.4% and 72.5% was observed in 2019 and 2020, respectively [22]. The induced sterility, similar to our reduction in rate of hatched eggs, was 31.7% in 2019 and 19.5% in 2020. And the reduction in adult female density was estimated to be 80.5% in 2019 and 71.3% in 2020 [22]. Therefore, despite a level of induced sterility below 50%, considered a critical threshold by other studies [19], a clear population reduction was observed. Similarly, with a reduction in hatching rate of 19%, we observed a clear impact of SIT on the *Ae. albopictus* adult female population, with a reduction of 63% compared to the control area.

Comparing the mosquito population between the release site and the control site gave us good insights into the impact of the SIT throughout the mosquito breeding season, but it did not provide information on the spatial variation of the impact, which we expected to be present. By spatially modeling the three population parameters we were able to capture the spatial variations in the impact of the SIT within the treated area. On the western part of the release area, which is more geographically isolated, we observed a significant reduction in the number of eggs. By contrast, on the eastern part the number of eggs increased as we approached the urban area of the neighbouring municipality Vico Morcote, not treated with sterile males, eventually becoming four times higher than on the western part. A similar spatial pattern was observed for the rate of hatched eggs and the number of adult females. What we observed on the eastern side is likely a decrease in the effectiveness of the SIT due to a border effect at the edges of the treated area, with gradually increasing exchange of mosquitoes and possible immigration of wild males and mated females from the untreated adjacent municipality Vico Morcote. This is consistent with previous trials where the highest levels of population suppression were observed in more isolated areas surrounded by vegetation and with limited transport links [43]. The same study also observed that less isolated zones nearer transportation routes with frequent traffic were relatively resistant to population suppression, suggesting that human activities facilitate mosquito immigration into release sites and compromise the efficiency of *Ae. albopictus* elimination [43].

While the spatial analysis allowed us to observe the impact of SIT on a small spatial scale, it also provided the opportunity to observe that using a geographically isolated area for the trial limited our ability to fully assess border effects. This is crucial, for example, to define the minimum number of sterile males required to achieve a certain reduction in the mosquito population in an area of a certain size and consequently quantify the cost effectiveness of the SIT. Therefore, we believe that it might be useful to conduct SIT trials in non-isolated areas using an experimental design allowing for a clearer evaluation of border effects. To our knowledge, very few studies have analyzed the influence of wild population migration in large SIT target areas [22].

Although unlikely, the spatial variation observed in the release site could also be due to other unmeasured factors even not related to the SIT treatment. Altitude did not seem to play a role, as we would expect more mosquitoes on the western part of the release area, where the slope is gentler, compared to the eastern part, which has a steeper gradient; however, this was not the case. Interestingly, on the western part of the release area, two spots show adult female numbers double the average, with a similar, though less pronounced, pattern for adult males. Given the absence of a border effect in this area, this concentration may be linked to weather conditions (e.g., wind, micro air currents), microhabitat features (e.g., feeding/resting sites, hosts), or human-related factors (e.g., untreated breeding hot spots). To optimize SIT deployment, it is paramount to replicate releases across diverse habitats to assess how SIT effectiveness varies in different environments.

The trial generated significant public interest and acceptance, with over 50 media (television, radio, newspapers) appearances in Switzerland, without any negative comment. Additionally, some municipalities and cantons expected the technique to be adopted right away in the regular surveillance and control system. Since it has been shown that the SIT has a greater effectiveness if releases are made 2 years in a row [22] the experiment has been repeated in 2024 (data analysis is currently underway).

Several aspects can still be optimized to enhance the cost-effectiveness of SIT. Key factors include the production, sterilization, transport, and release of males. The higher the quality of sterile males, the fewer males need to be released, resulting in a decrease in the costs associated with SIT application. For example, we observed that mortality rates at release decreased when mosquito density during transportation was halved and transportation time was reduced. We also observed that low field temperatures at the beginning of the season prevented the males from becoming active after transportation. In this context, it is essential to evaluate whether it is advisable to withhold the release of males when temperatures fall below a certain threshold, to avoid unnecessary costs. Finally, evaluation of mosquito population dynamics can also be improved. For instance, in addition to assessing the reduction in female numbers, it will be crucial to determine their age as well, given its significance in assessing disease transmission risk [44].

## Conclusions

This study demonstrated the efficacy of integrating SIT into existing vector control and surveillance activities in southern Switzerland. The results are noteworthy, with a 57% decrease in egg numbers and a 66% reduction in adult females in the target area. To better assess the effectiveness of SIT in realistic settings, we recommend conducting trials in geographically non-isolated areas and across a variety of habitats. This will provide clearer insights for stakeholders and decision-makers regarding the potential benefits of this technique for vector control.

## Supplementary Information

Supplementary Material 1. Supplementary methods, figures, and tables.

Supplementary Material 2. Analysis total number of *Ae. albopictus* eggs laid.

Supplementary Material 3. Analysis percentage of *Ae. albopictus* eggs that hatched.

Supplementary Material 4. Analysis number of *Ae. albopictus* adult females.

## Data Availability

The datasets used and/or analyzed during the current study are available from the corresponding author on reasonable request.

## Abbreviations

AIC: Akaike information criterion
BIC: Bayesian information criterion
CAA: Centro Agricoltura Ambiente “G. Nicoli”
FAO: Food and Agriculture Organization of the United Nations
GAMM: Generalized additive mixed-effects model
GAM: Generalized additive model
HLC: Human landing collection
IAEA: International Atomic Energy Agency
IVM: Integrated vector management
MRR: Mark release and recapture
SIT: Sterile insect technique
WHO: World Health Organization
