# Supplementary material for: Effectiveness of the sterile insect technique in controlling Aedes albopictus as part of an integrated control measure: evidence from a first small-scale field trial in Switzerland
Source: Infect Dis Poverty. 2025 Aug 22;14:90. doi: 10.1186/s40249-025-01360-2 (PMC12372186; doi:10.1186/s40249-025-01360-2)
Supplement: Supplementary file 1 — Supplementary Material 1. Supplementary methods, figures, and tables. [file 40249_2025_1360_MOESM1_ESM.pdf]

Supplementary file 1

Method S1: Residual fertility of sterile males

For this quality control test, a random sample of 200 sterile males was placed in a BugDorm rearing cage (W30 × D30 × H30 cm, BioQuip, Compton, CA, USA) along with 200 non-irradiated virgin females. An equal number of non-irradiated males and females were used as untreated control. The mosquitoes were provided with sugar and allowed to mate. Females were blood-fed twice, on days 3 and 4 after being introduced into the cage. The eggs produced were allowed to mature for one week before being subjected to the egg hatching protocol [1, 2]. Residual fertility was assessed by dividing the number of hatched eggs by the total number of eggs prior to the hatching protocol.

Method S2: Human landing collection

Human landing collections (HLC) were conducted every 24 hours from the 3rd to the 9th of August 2022, following the release of marked sterile males on the 2nd of August. The mark release and recapture (MRR) test area (Fig. S2) was divided into four concentric circles, 50 m apart, within which 32 HLC points were evenly distributed (distance between contiguous points: 50–100 m). The HLC points were located in areas favourable for mosquitoes resting (shaded and close to vegetation). Four field technicians rotated daily through the 32 collection points (8 collection points per technician) between 18:00 and 19:30 local time. HLCs were not conducted on the 6th and 7th of August due to stormy winds. Each HLC session lasted 5 minutes, during which mosquitoes were captured with manual aspirators.

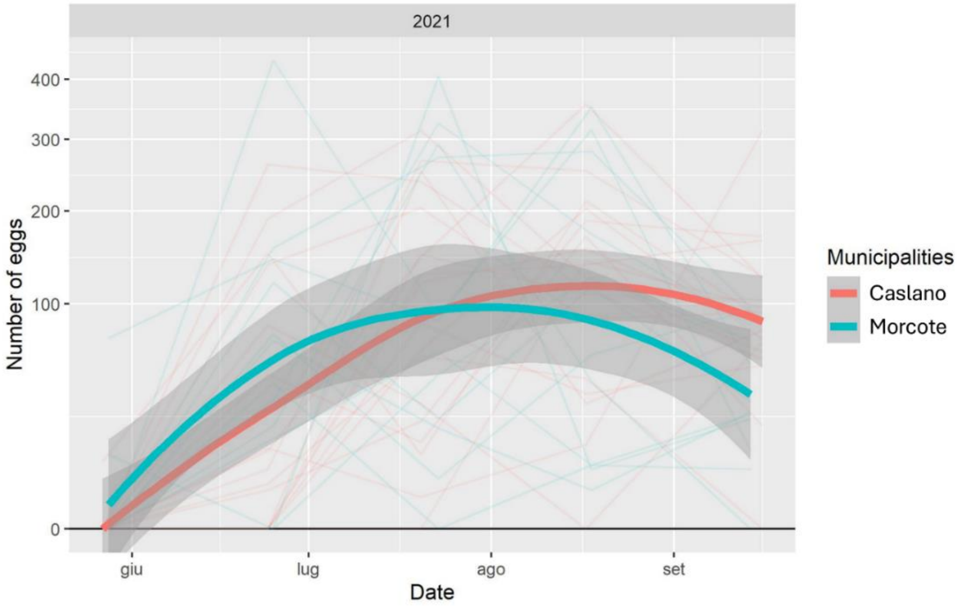

Fig. S1 Average number of invasive *Aedes* species eggs per ovitrap collected in 2021 in the municipalities of Morcote and Caslano.

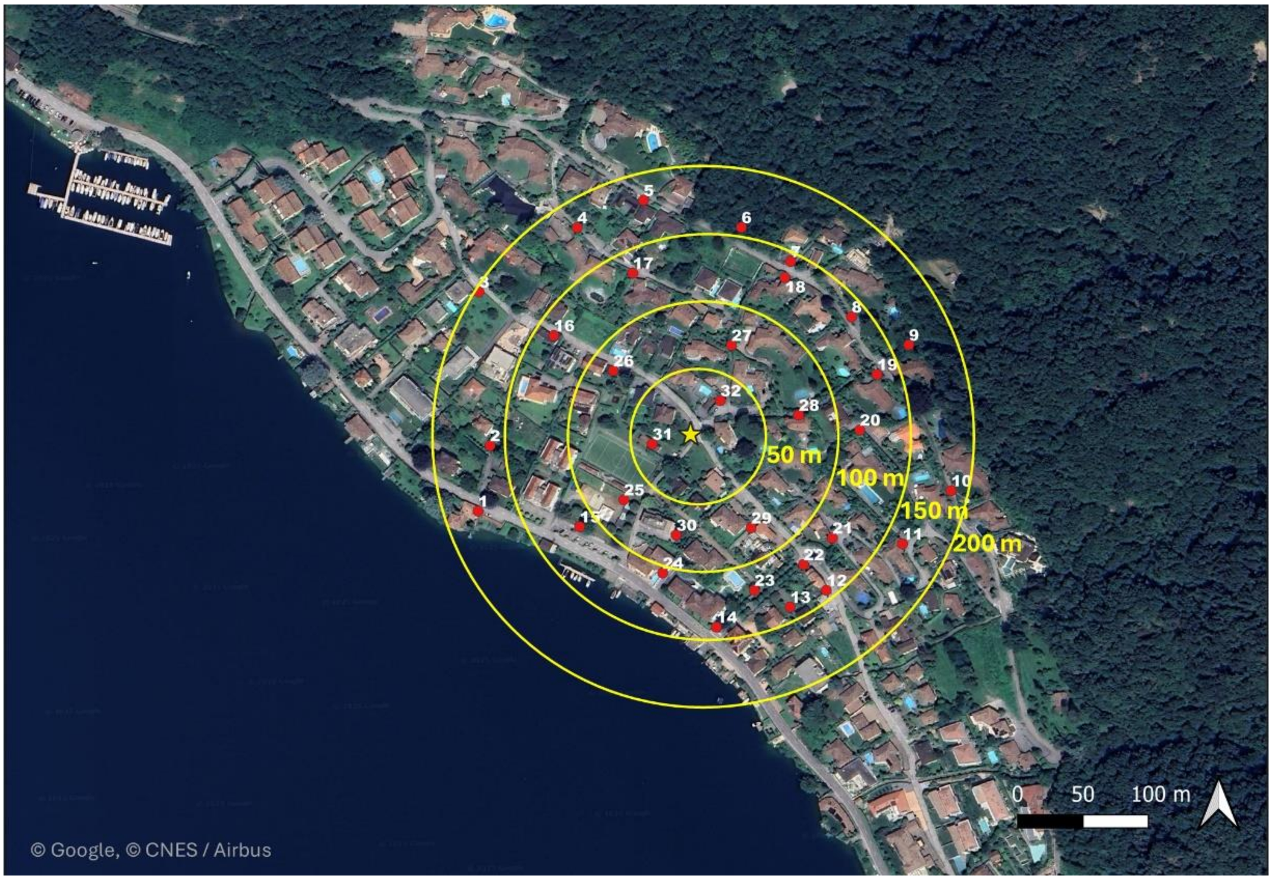

Fig. S2 2022 and 2023 MRR study at the release site. The yellow star marks the release point, while the red points indicate the 32 recapture stations. The concentric yellow circles indicate the recapture distances from the release point.

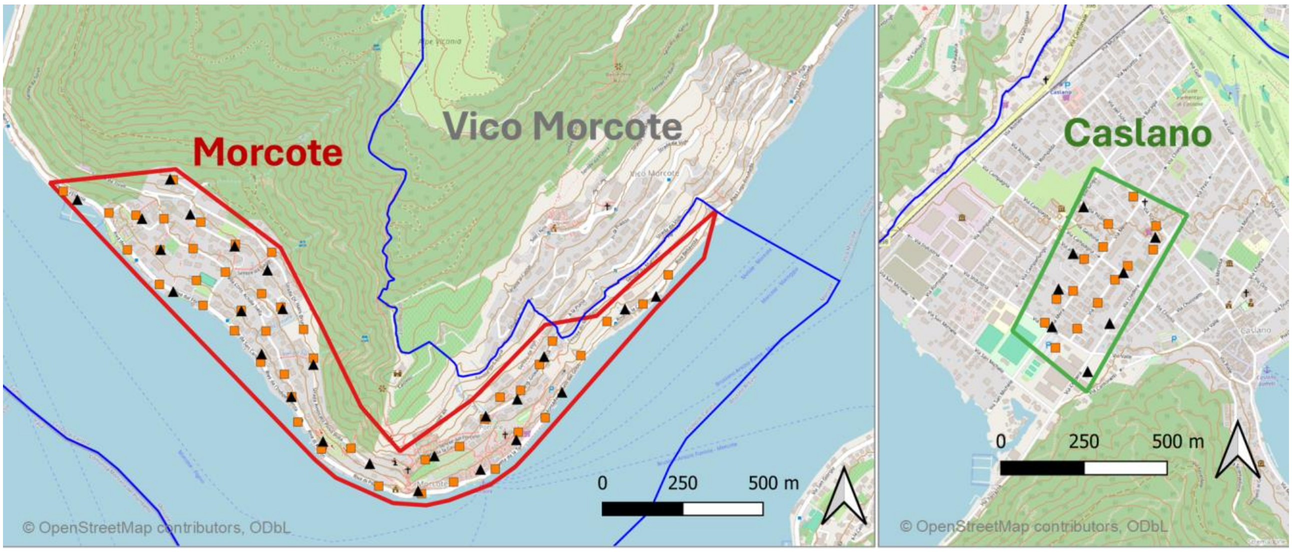

Fig. S3 Monitoring of *Ae. albopictus* eggs and adults at the release (left) and control (right) sites. Forty-five ovitraps (orange squares) were placed at the release site, and 14 at the control site. The number of adult female traps (black triangles) was 25 at the release site and 8 at the control site. The brownish lines represent contour lines at 20-meter intervals. The boundaries of the study municipalities, Morcote and Caslano, are outlined in blue.

Table S1 Transportation and release methods and mortality rate of sterile males at release.

**Transportation**		**Release**			**Average mortality rate inside the release cups after 1 hour**	
No. sterile males/cup	Delivery method	Release event	Date	No. releases/week	%	Notes
2000	Express courier (18 h)	1	03.05.2023	1	45.4	
2000	Express courier (18 h)	2	09.05.2023	1	56.4	Wake up 24h first in lab
2000	Express courier (18 h)	3	16.05.2023	1	48.9	
2000	Express courier (18 h)	4	23.05.2023	1	55.0	
1000	Express courier (18 h)	5	30.05.2023	1	62.7	
1000	Express courier (18 h)	6	06.06.2023	1	28.1	
1000	Express courier (18 h)	7	13.06.2023	1	27.4	
1000	Express courier (18 h)	8	20.06.2023	1	29.3	
1000	Express courier (18 h)	9	27.06.2023	2	18.4	
1000	Express courier (18 h)	10	30.06.2023	2	27.8	
1000	Express courier (18 h)	11	04.07.2023	2	9.0	
1000	Express courier (18 h)	12	07.07.2023	2	19.9	
1000	Express courier (18 h)	13	11.07.2023	2	26.1	
1000	Express courier (18 h)	14	14.07.2023	2	15.7	
1000	Express courier (18 h)	15	18.07.2023	2	37.6	
1000	Express courier (18 h)	16	21.07.2023	2	13.1	
1000	Express courier (18 h)	17	25.07.2023	2	16.7	
1000	Express courier (18 h)	18	28.07.2023	2	16.8	
1000	Express courier (18 h)	19	02.08.2023	2	24.6	
1000	Express courier (18 h)	20	04.08.2023	2	14.7	
1000	Express courier (18 h)	21	08.08.2023	2	14.5	
1000	Express courier (18 h)	22	11.08.2023	2	8.9	
1000	Express courier (18 h)	23	16.08.2023	2	39.4	2 days delivery
1000	Express courier (18 h)	24	18.08.2023	2	20.1	
1000	Express courier (18 h)	25	22.08.2023	2	-	
1000	Express courier (18 h)	26	24.08.2023	2	17.1	
1000	Direct transportation by car/train (3 h)	27	28.08.2023	2	15.4	
1000	Direct transportation by car/train (3 h)	28	01.09.2023	2	19.1	
1000	Direct transportation by car/train (3 h)	29	04.09.2023	2	6.2	
1000	Direct transportation by car/train (3 h)	30	08.09.2023	2	6.3	
1000	Direct transportation by car/train (3 h)	31	11.09.2023	2	5.4	
1000	Direct transportation by car/train (3 h)	32	15.09.2023	2	8.2	
1000	Direct transportation by car/train (3 h)	33	18.09.2023	1	14.1	
1000	Direct transportation by car/train (3 h)	34	29.09.2023	1	6.2	

Table S2 Mark release and recapture (MRR) results in the trial area in 2022 and 2023.

Release date	NAM	FP	MR	θ	s	ALE	MDD
2022.08.02	22,500	0.4	10.7	0.21	0.59	1.9	91.0
2023.06.13	10,000	0.3	43.0	0.25	0.62	2.1	72.5
2023.07.11	20,000	0.4	48.5	1.57	0.40	1.1	71.9
2023.08.22	20,000	0.3	29.1	0.01	0.87	7.2	147.4

NAM: No. of adult males released
FP: Female presence (%)
MR: Mortality rate of the marked males at release (%)
θ: Male recapture rate (%)
s: Survival rate (linear correction) (%)
ALE: Average life expectancy (days)
MDD: Mean distance dispersed (m)

Table S3 Range of dispersion of marked sterile males in 2022 and 2023.

Range from release point (m)	No. of males recaptured		% of males recaptured	
	2022	2023	2022	2023
0–50	30	87	46.2	35.5
50–100	24	108	36.9	44.1
100–150	8	41	12.3	16.7
150–200	3	9	4.6	3.7

References

1. Food and Agriculture Organization of the United Nations, International Atomic Energy Agency. Guidelines for Mark-Release-Recapture Procedures of *Aedes* Mosquitoes. Vienna: FAO & IAEA; 2020.
2. Joint FAO/IAEA Division of Nuclear Techniques in Food and Agriculture Vienna (Austria). Guidelines for Colonization of *Aedes* Mosquito Species. Version 1. 2018.
