# Supplementary material for: Effectiveness of the sterile insect technique in controlling Aedes albopictus as part of an integrated control measure: evidence from a first small-scale field trial in Switzerland
Source: Infect Dis Poverty. 2025 Aug 22;14:90. doi: 10.1186/s40249-025-01360-2 (PMC12372186; doi:10.1186/s40249-025-01360-2)
Supplement: Supplementary file 2 — Supplementary Material 2. Analysis total number of Ae. albopictus eggs laid. [file 40249_2025_1360_MOESM2_ESM.pdf]

Modelling: Paper Morcote Project

Number of eggs | 2023

Author: Nisia Trisconi & Dr. Matteo Tanadini & Dr. Luisa Barbanti | Zurich Data Scientists
Reviewer: TBD | Zurich Data Scientists

February 3, 2025

Contents

1 Freeze Package versions **2**

2 Settings **2**

3 Getting data **3**

4 Aim and design **5**

5 Generalised Additive Mixed-Effects Model (GAMM) **5**
5.1 Visualising the data . . . 5
5.2 Model for Morcote and Caslano . . . 10
5.2.1 Fitting the model . . . 10
5.2.2 Plotting the smoothers . . . 13
5.2.3 Fitted values . . . 16
5.2.4 Predicted values . . . 17
5.2.5 Model selection – Shape . . . 20
5.2.6 Checking the offset . . . 23
5.2.7 Residual analysis . . . 25
5.2.8 Comparing (over)dispersion in the two models . . . 33

6 Spatial Generalised Additive Model (spatial GAM) **36**
6.1 Visualising the data . . . 36
6.2 Fitting the models . . . 39
6.3 Residual analysis . . . 45
6.4 Comparing (over)dispersion in the two models . . . 49

7 Methods **49**
7.1 Generalised Additive Mixed-Effects Model (GAMM) . . . 49
7.2 Spatial Generalised Additive Model (spatial GAM) . . . 50

8 Results **50**
8.1 Generalised Additive Mixed-Effects Model (GAMM) . . . 50
8.2 Spatial Generalised Additive Model (spatial GAM) . . . 50

9 Conclusions **51**

10 References **51**

11 Session Information **52**

1 Freeze Package versions

```
## (messages are omitted from this chunk)
##
library(groundhog)
pkgs <- c("dplyr",
          "ggplot2",
          "lubridate",
          "mgcViz",
          "readxl") ## automatically loads mgcv
groundhog.library(pkgs, date = "2024-11-01")
```

2 Settings

Global settings:

```
Sys.setenv(lang = "en_US")
theme_set(theme_bw())

if (!dir.exists("Prepared_data_and_models")) {
  dir.create("Prepared_data_and_models")
}
```

3 Getting data

```r
## temporal analysis
d.ovitraps <- readRDS(file = paste0("Prepared_data_and_models/",
                                    "d.ovitraps_PreparedData.RDS"))
##
## spatial analysis, used later in the analysis
d.ovitraps.spatial <- readRDS(file = paste0("Prepared_data_and_models/",
                                            "d.ovitraps.spatial_PreparedData.RDS"))
```

For this file, we only consider the year 2023 and the municipalities Morcote and Caslano. So, we define a reduced data set:

```r
d.ovitraps.23 <- d.ovitraps %>%
  filter(Year == 2023 & municipality.fac %in% c("Morcote", "Caslano")) %>%
  droplevels()

## check
d.ovitraps.23 %>%
  select(Year) %>%
  unique()
```

```
# A tibble: 1 x 1
   Year
  <dbl>
1  2023
```

```r
d.ovitraps.23 %>%
  select(municipality.fac) %>%
  unique()
```

```
# A tibble: 2 x 1
  municipality.fac
  <fct>
1 Caslano
2 Morcote
```

Overview of the data:

```r
dim(d.ovitraps.23)
```

```
[1] 1637   33
```

```r
head(d.ovitraps.23)[1:min(ncol(d.ovitraps.23), 30)]
```

```
# A tibble: 6 x 30
  OriginalSheet Activation.time total.eggs total.albopictus.egg
  <chr>                   <dbl>      <dbl>                <dbl>
1 1                          14          0                    0
2 1                          14          0                    0
3 1                          14         15                   15
4 1                          14         57                    0
5 1                          14        113                    0
6 1                          14          0                    0
# i 26 more variables: WHOLE.albopictus.egg <dbl>,
#   DESSICATED.albopictus.egg <dbl>, HATCH.albopictus.egg <dbl>,
#   WHOLE.albopictus.egg..AFTER.HATCHING.PROCEDURE. <dbl>,
```

```
#   DESSICATED.albopictus.egg..AFTER.HATCHING.PROCEDURE. <dbl>,
#   HATCH.albopictus.egg..AFTER.HATCHING.PROCEDURE. <dbl>,
#   Perc.egg.hatch.per.trap <dbl>, Perc.egg.hatch.per.area <dbl>, Year <dbl>,
#   unique.ID <fct>, X.num <dbl>, Y.num <dbl>, round.fac <fct>, ...
```

```r
str(d.ovitraps.23)
```

```
tibble [1,637 x 33] (S3: tbl_df/tbl/data.frame)
 $ OriginalSheet                                       : chr [1:1637] "1" "1" "1" "1" ...
 $ Activation.time                                     : num [1:1637] 14 14 14 14 14 14 14 14 14 14 ...
 $ total.eggs                                          : num [1:1637] 0 0 15 57 113 0 0 0 0 0 ...
 $ total.albopictus.egg                                : num [1:1637] 0 0 15 0 0 0 0 0 0 0 ...
 $ WHOLE.albopictus.egg                                : num [1:1637] NA NA NA NA NA NA NA NA NA NA ...
 $ DESSICATED.albopictus.egg                           : num [1:1637] NA NA NA NA NA NA NA NA NA NA ...
 $ HATCH.albopictus.egg                                : num [1:1637] NA NA NA NA NA NA NA NA NA NA ...
 $ WHOLE.albopictus.egg..AFTER.HATCHING.PROCEDURE.     : num [1:1637] NA NA NA NA NA NA NA NA NA NA ...
 $ DESSICATED.albopictus.egg..AFTER.HATCHING.PROCEDURE.: num [1:1637] NA NA NA NA NA NA NA NA NA NA ...
 $ HATCH.albopictus.egg..AFTER.HATCHING.PROCEDURE.     : num [1:1637] NA NA NA NA NA NA NA NA NA NA ...
 $ Perc.egg.hatch.per.trap                             : num [1:1637] NA NA NA NA NA NA NA NA NA NA ...
 $ Perc.egg.hatch.per.area                             : num [1:1637] NA NA NA NA NA NA NA NA NA NA ...
 $ Year                                                : num [1:1637] 2023 2023 2023 2023 2023 ...
 $ unique.ID                                           : Factor w/ 77 levels "Caslano.1","Caslano.10",..
 $ X.num                                               : num [1:1637] NA NA NA NA NA NA NA NA NA NA ...
 $ Y.num                                               : num [1:1637] NA NA NA NA NA NA NA NA NA NA ...
 $ round.fac                                           : Factor w/ 34 levels "1","2","3","4",..: 6 6 6
 $ Setting_date.date                                   : Date[1:1637], format: "2023-05-11" "2023-05-11
 $ Sampling_date.date                                  : Date[1:1637], format: "2023-05-25" "2023-05-25
 $ municipality.ord                                    : Ord.factor w/ 2 levels "Caslano"<"Morcote": 1
 $ municipality.fac                                    : Factor w/ 2 levels "Caslano","Morcote": 1 1 1
 $ status.fac                                          : Factor w/ 3 levels "A","F","M": 2 2 2 2 2 2 2
 $ perc.manual                                         : num [1:1637] NA NA NA NA NA NA NA NA NA NA ...
 $ RowNumber                                           : int [1:1637] 577 578 579 580 581 582 583 584 5
 $ Year.fac                                            : Factor w/ 1 level "2023": 1 1 1 1 1 1 1 1 1 1
 $ hatched.albo.eggs.after.proc                        : num [1:1637] NA NA NA NA NA NA NA NA NA NA ...
 $ daily.albopictus.eggs                               : num [1:1637] 0 0 1.07 0 0 ...
 $ yday                                                : num [1:1637] 145 145 145 145 145 145 145 145 1
 $ non.hatched.albo.eggs.after.proc                    : num [1:1637] NA NA NA NA NA NA NA NA NA NA ...
 $ week                                                : num [1:1637] 21 21 21 21 21 21 21 21 21 21 ...
 $ unique.ID.Year                                      : Factor w/ 77 levels "Caslano.1.2023",..: 1 28
 $ super.municipality.year                             : Factor w/ 2 levels "Caslano:2023",..: 1 1 1 1
 $ municipality.fac.spatial                            : Factor w/ 2 levels "Caslano","Morcote": 1 1 1
```

```r
##
dim(d.ovitraps.spatial)
```

```
[1] 164   7
```

```r
head(d.ovitraps.spatial)[1:min(ncol(d.ovitraps.spatial), 30)]
```

```
# A tibble: 6 x 7
  unique.ID  Year.fac mean.eggs median.eggs  X.num  Y.num municipality.fac.spa~1
  <fct>      <fct>        <dbl>       <dbl>  <dbl>  <dbl> <fct>
1 Vico Morc~ 2023          14.6          10 7.15e5 87560  buffer area extended
2 Vico Morc~ 2024          54            14 7.15e5 87560  buffer area extended
3 Caslano.1~ 2023          37.6        18.5 7.12e5 92314. Caslano
4 Caslano.1~ 2024         104.          119 7.12e5 92314. Caslano
```

```
5 Morcote.1~ 2023          4.79         0   7.14e5 86745. Morcote
6 Morcote.1~ 2024         53.3         14   7.14e5 86745. Morcote
# i abbreviated name: 1: municipality.fac.spatial
```

```
str(d.ovitraps.spatial)
```

```
tibble [164 x 7] (S3: tbl_df/tbl/data.frame)
 $ unique.ID              : Factor w/ 348 levels "Caslano.1","Vico Morcote.1",..: 2 2 5 5 8 8 17 17 21
 $ Year.fac               : Factor w/ 2 levels "2023","2024": 1 2 1 2 1 2 1 2 1 2 ...
 $ mean.eggs              : num [1:164] 14.56 54 37.62 104.18 4.79 ...
 $ median.eggs            : num [1:164] 10 14 18.5 119 0 14 45 22 0 0 ...
 $ X.num                  : num [1:164] 714909 714909 711546 711546 714371 ...
 $ Y.num                  : num [1:164] 87560 87560 92314 92314 86745 ...
 $ municipality.fac.spatial: Factor w/ 14 levels "Ascona","Balerna",..: 4 4 6 6 13 13 6 6 13 13 ...
 - attr(*, "na.action")= 'omit' Named int [1:92] 1 4 9 10 11 12 17 18 23 24 ...
  ..- attr(*, "names")= chr [1:92] "1" "4" "9" "10" ...
```

4 Aim and design

In 2023, SUPSI received approval from the FOEN to release sterile male tiger mosquitoes in the municipality of Morcote as part of a field experiment aimed at reducing the number of eggs laid by female mosquitoes.

The experiment involved weekly releases of sterile male *Aedes albopictus* mosquitoes in Morcote during the active mosquito season, from May to September 2023. Approximately 3,000 sterile males per hectare were released each week through 75 predefined stations spaced 50 to 80 meters apart.

Ovitraps were sampled weekly in Morcote (treated) and Caslano (untreated) to assess whether the release of sterile males had an impact on the number of *Aedes albopictus* eggs. Additionally, ovitraps from other municipalities were sampled bi-weekly to provide further comparative data with the treated municipality. However, since only Morcote and Caslano are comparable, we have eliminated all other municipalities from the following analysis.

In total, the data was collected on `34` different dates from `2` distinct municipalities.

Morcote was sampled `25` times, whereas Caslano was sampled `34` times.

5 Generalised Additive Mixed-Effects Model (GAMM)

5.1 Visualising the data

The aim of this analysis is to determine whether the release of sterilised males in a given area helps reducing the number of laid eggs.

With this purpose in mind, we begin by displaying the number of eggs found in the traps sampled throughout the season. As we are dealing with count data, we apply a square-root transformation to the response variable *total.albopictus.egg* to stabilise variance.

Furthermore, observations from the same ovitrap are connected with a line to visualise trends over time. On top of these lines, we add a smoothed average that allows us to see the global trend.

```
## (messages and warnings are excluded from this chunk)
##
p <- ggplot(data = d.ovitraps.23,
            mapping = aes(y = `total.albopictus.egg`,
                          x = Sampling_date.date,
                          group = unique.ID)) +
  geom_hline(yintercept = 0) +
```

```
  geom_point(alpha = 0.2) +
  geom_line(alpha = 0.2) +
  scale_y_sqrt() +
  geom_smooth(mapping = aes(group = NULL))
p
```

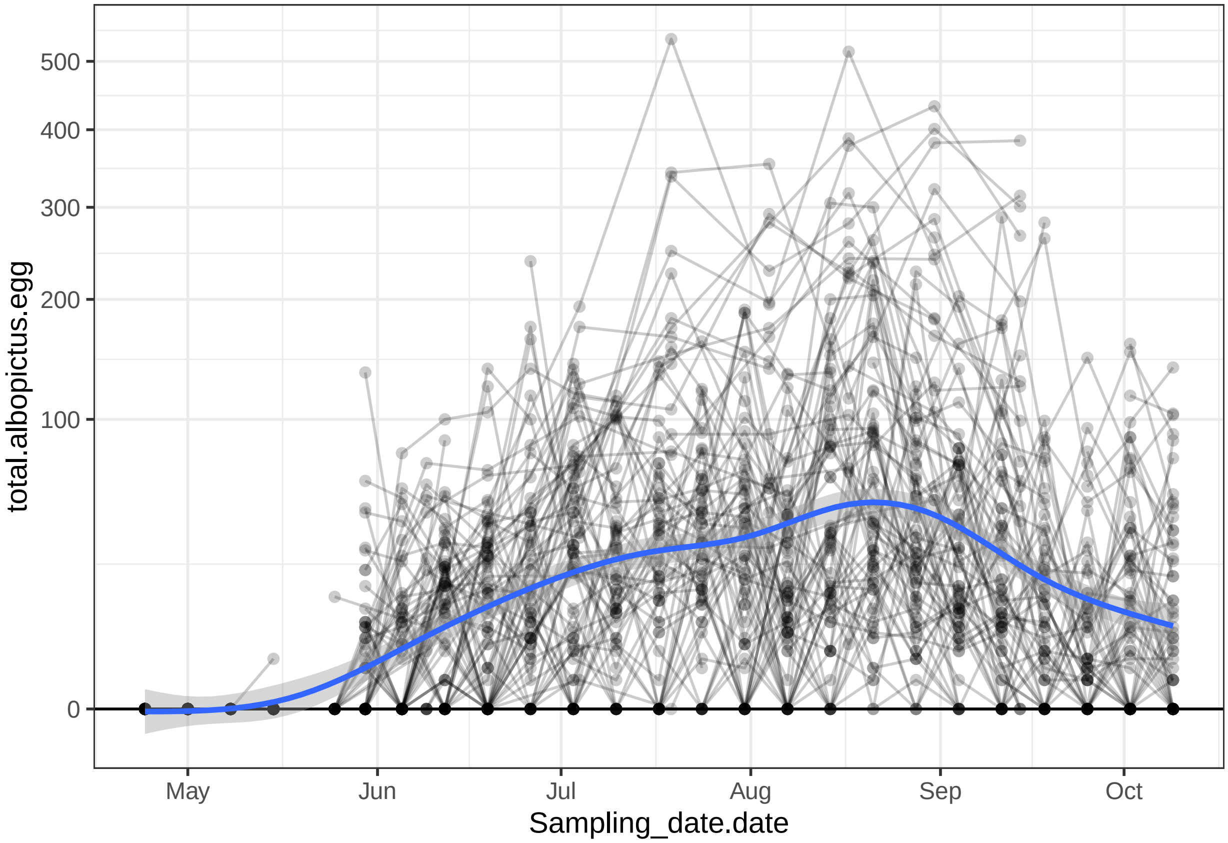


```
saveRDS(p, file = file.path("saved_figures", "2a_eggsOverTime.rds"))
```

The number of eggs laid seems to increase over the course of the season, peaking at the end of August, and then decreasing rapidly; however, fewer ovitraps are sampled towards the end.

The sterilised males were released only in Morcote, while Caslano served as control. For this reason, we now create a separate plot for each municipality to observe whether there are any differences between them.

```
## (messages and warnings are excluded from this chunk)
##
p <- ggplot(data = d.ovitraps.23,
            mapping = aes(y = `total.albopictus.egg`,
                          x = Sampling_date.date,
                          group = unique.ID)) +
  geom_hline(yintercept = 0) +
  geom_point(alpha = 0.2) +
  geom_line(alpha = 0.2) +
  scale_y_sqrt() +
  facet_wrap(~municipality.fac) +
  geom_smooth(mapping = aes(group = 1), method = "loess")
p
```

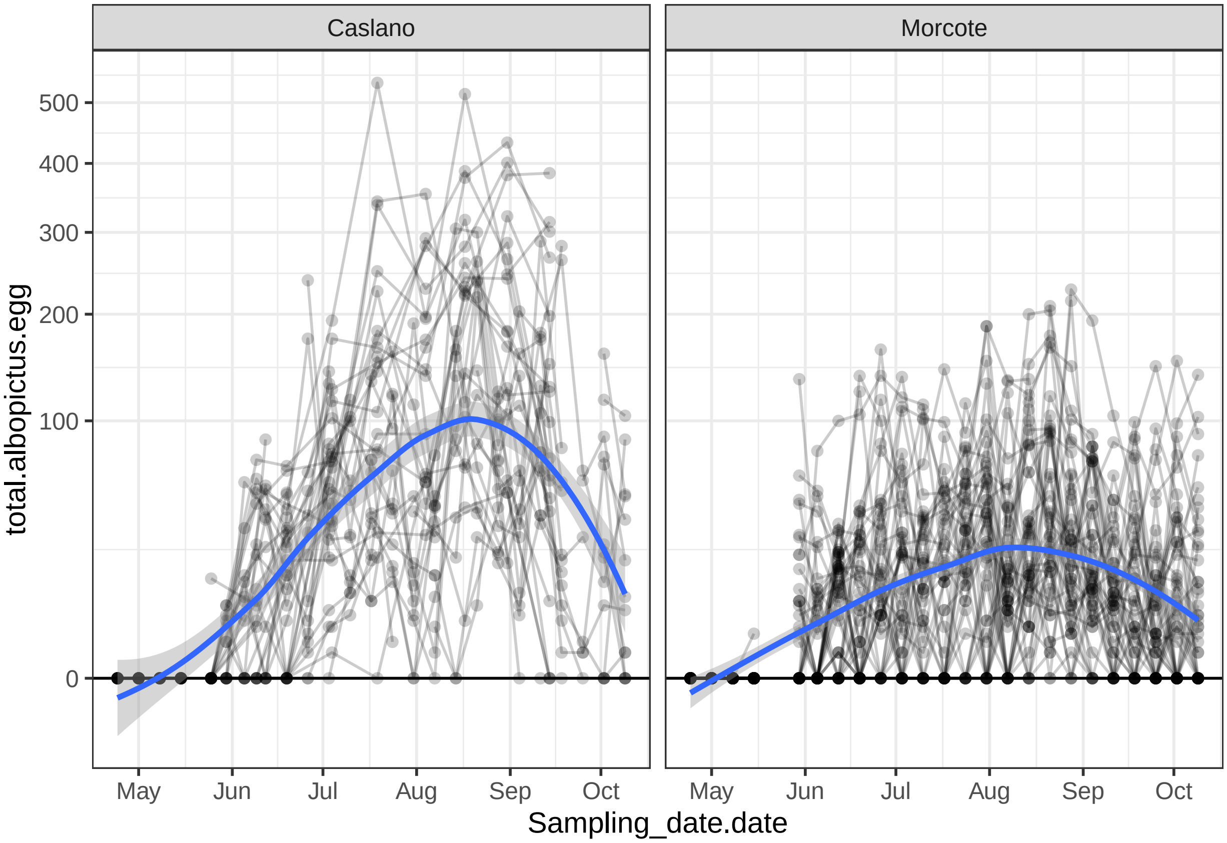


```
## save plot for future use:
saveRDS(p, file = file.path("saved_figures", "2a_TotalEggsMuniPanel.rds"))
```

Morcote seems to have, on average, fewer eggs laid than Caslano.

To facilitate comparison, rather than creating separate plots, we overlay the two municipalities on a single plot (colouring them with different colours) and add a smoother for each one.

```
## (messages and warnings are excluded from this chunk)
##
p <- ggplot(data = d.ovitraps.23,
            mapping = aes(y = `total.albopictus.egg`,
                          x = Sampling_date.date,
                          group = unique.ID,
                          colour = municipality.fac)) +
  geom_hline(yintercept = 0) +
  geom_point(alpha = 0.1) +
  geom_line(alpha = 0.1) +
  scale_y_sqrt(limits = c(0, NA)) +
  # geom_line(stat = "smooth",
  #           method = "loess",
  #           se = FALSE,
  #           mapping = aes(group = municipality.fac),
  #           alpha = 0.5)
  #
  geom_smooth(mapping = aes(group = municipality.fac),
              method = "loess",
```

```
              alpha = 0.2)
p
```

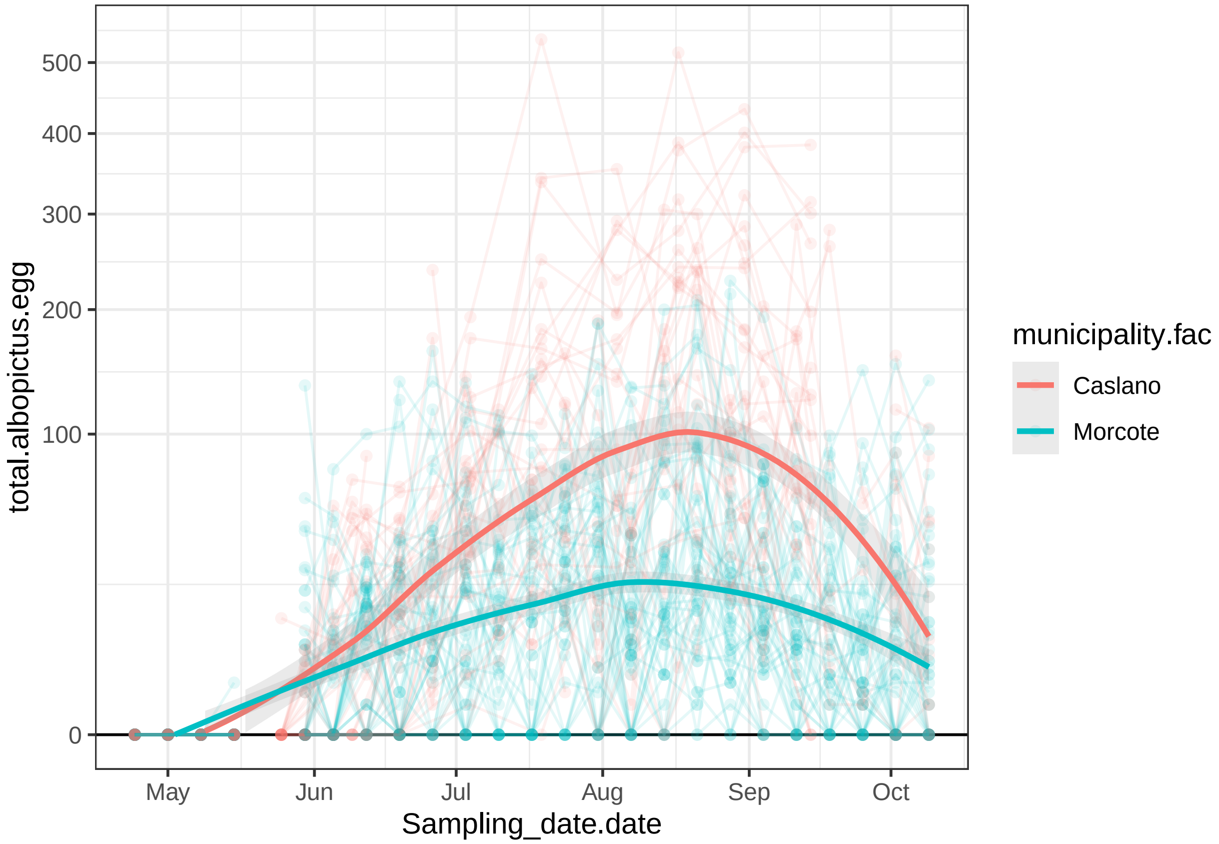


```
## save plot for future use:
saveRDS(p, file = file.path("saved_figures", "2a_TotalEggsMuniSmooth.rds"))
```

This graph confirms that the number of eggs laid in Morcote is lower than in Caslano for the 2023 season.

Finally, we focus on Morcote to examine seasonal differences among the ovitraps. Since we are not concerned with the behaviour of individual ovitraps, we remove the labels.

```
## (messages and warnings are excluded from this chunk)
##
p <- ggplot(data = filter(d.ovitraps.23, municipality.fac == "Morcote"),
            mapping = aes(y = `total.albopictus.egg`,
                          x = Sampling_date.date,
                          group = unique.ID)) +
  geom_hline(yintercept = 0) +
  geom_point() +
  geom_line() +
  scale_y_sqrt(limits = c(0, NA)) +
  facet_wrap(~unique.ID) +
  theme(
    strip.background = element_blank(),
    strip.text.x = element_blank(),
    axis.text.x = element_text(angle = 90)) +
  labs(title = "Morcote")
```

```
p
```

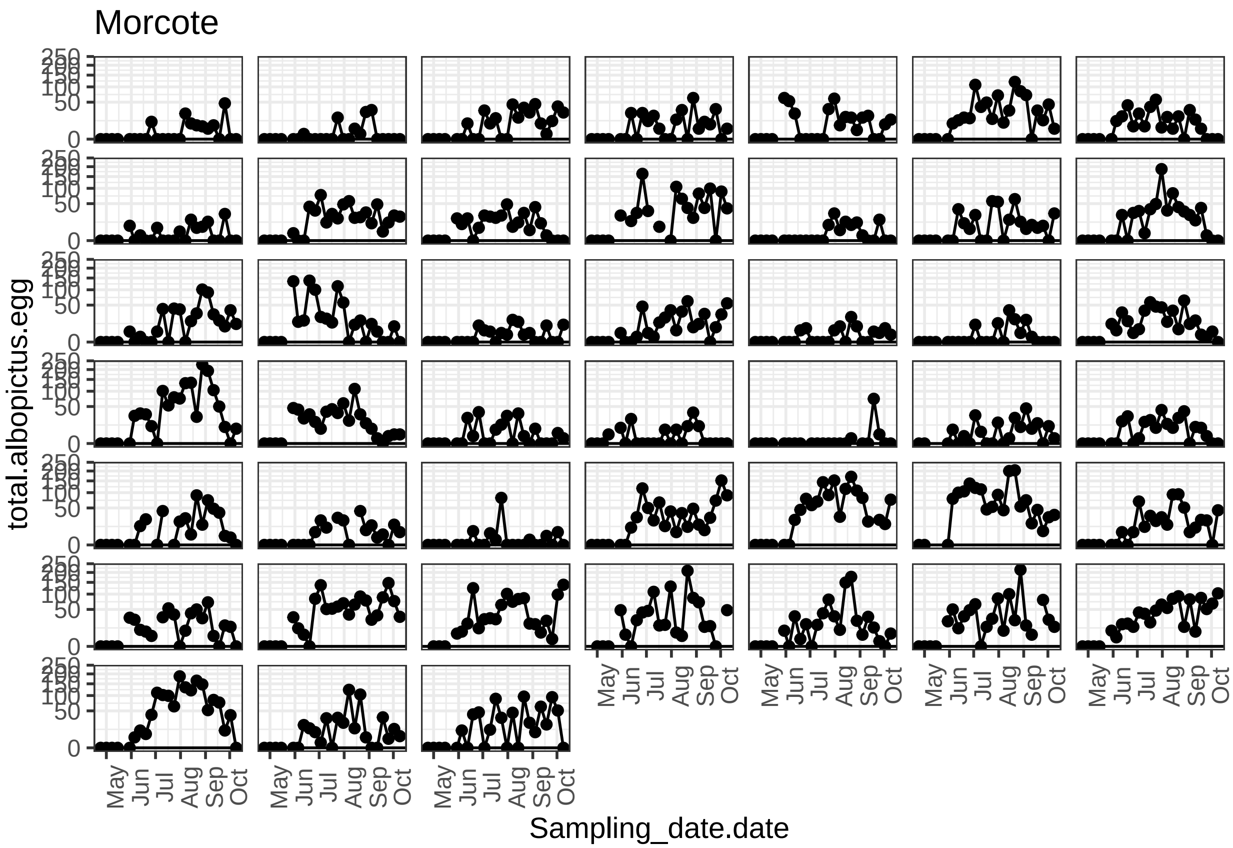


```
## save plot for future use:
saveRDS(p, file = file.path("saved_figures", "2a_MorcoteEggsOverTimeNolabels.rds"))
```

There is significant variation between ovitraps. For instance, in the bottom panel, we can observe an abrupt drop from 100 eggs one week to zero the next.

Internal comment: This is something very relevant to discuss for the design of the SIT.

We create the same plot for Caslano to determine whether the observed behaviour is a result of the release of sterilised males, or if it is a common pattern in the control municipalities as well.

```
## (messages and warnings are excluded from this chunk)
##
p <- ggplot(data = filter(d.ovitraps.23, municipality.fac == "Caslano"),
            mapping = aes(y = `total.albopictus.egg`,
                          x = Sampling_date.date,
                          group = unique.ID)) +
  geom_hline(yintercept = 0) +
  geom_point() +
  geom_line() +
  scale_y_sqrt(limits = c(0,NA), ) +
  facet_wrap(~unique.ID) +
  theme(
    strip.background = element_blank(),
    strip.text.x = element_blank(),
    axis.text.x = element_text(angle = 90)) +
```

```r
  labs(title = "Caslano")
p
```

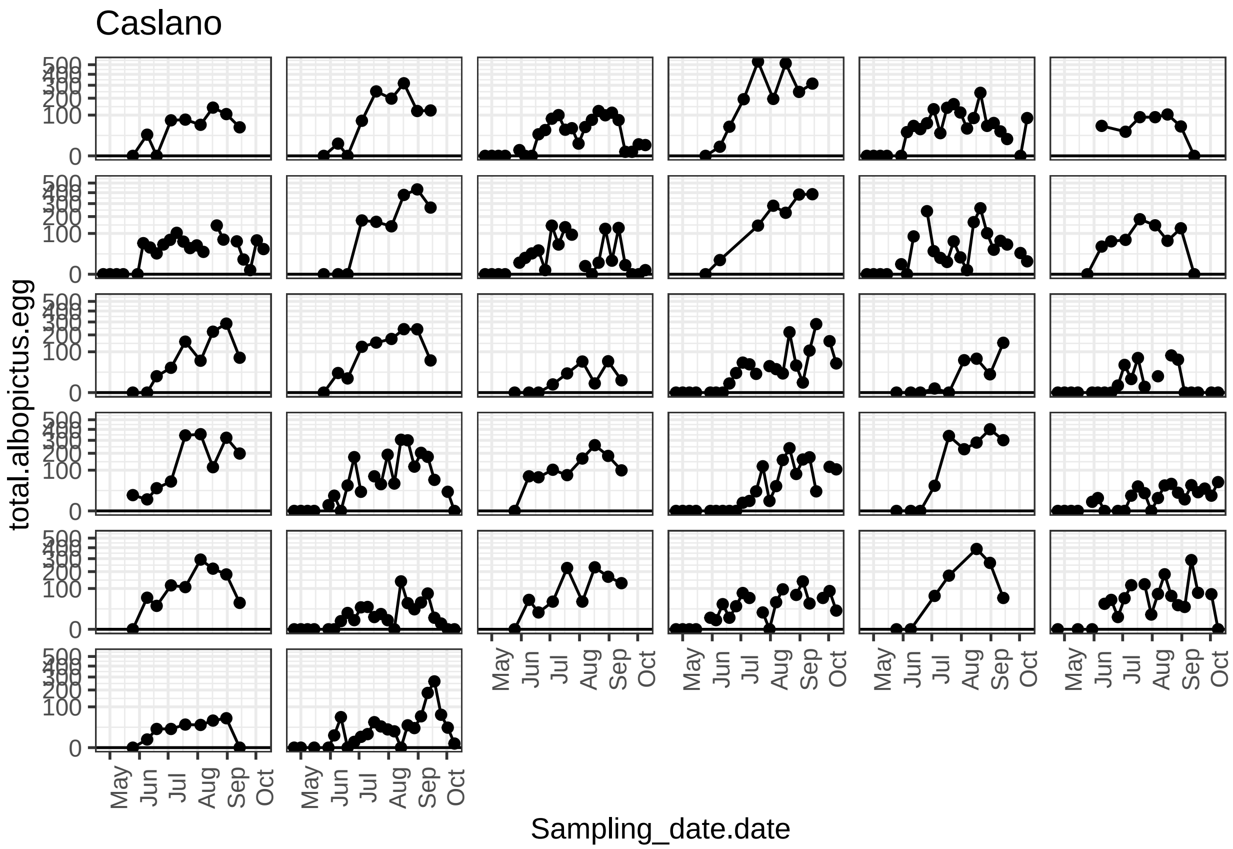


```r
## save plot for future use:
saveRDS(p, file = file.path("saved_figures", "2a_CaslanoEggsOverTimeNolabels.rds"))
```

There is still some variability, but it is less pronounced compared to Morcote.

@Diego: Morcote, which is treated, displays a lot of variability (probably because of the fact that males have often been released). In Caslano, there is less variability. Is this expected, as we are not interfering?

5.2 Model for Morcote and Caslano

5.2.1 Fitting the model

We aimed to model the total number of laid *Aedes albopictus* eggs (*total.albopictus.eggs*) across the season, represented by the day of the year (*yday*), while accounting for differences between municipalities (*municipality.fac*). Since sterilised mosquitoes were released only in Morcote, municipalities were treated as fixed effects rather than random effects, allowing us to assess actual differences in mosquito egg counts between municipalities.

To capture distinct seasonal patterns across municipalities, we included an interaction between *municipality.fac* and *yday*, thus avoiding the assumption that both municipalities follow the same seasonal trend. As *municipality.fac* is a categorical factor, it was included as a main effect in the model, and centered smooths were applied. In a subsequent analysis, we tested whether these distinct seasonal patterns were necessary.

yday was treated as a numeric variable. Given that *total.albopictus.eggs* represents count data, we employed a negative binomial model to account for overdispersion, which is common in count data.

To control for varying exposure times between ovitraps, we included *Activation.time* as an offset in the model. By log-transforming *Activation.time*, we assumed that doubling the activation time would lead to a proportional doubling in the number of eggs (in fact, having a log-log model corresponds to estimating the percentage increase in the response variable with a 1% increase in the explanatory variable, keeping all the rest constant). This assumption was further tested during the analysis.

Let's check which values are available for *Activation.time*

```
d.ovitraps.23 %>%
  group_by(municipality.fac) %>%
  reframe(range.activation.time.low = range(Activation.time, na.rm = TRUE)[1],
          range.activation.time.up = range(Activation.time, na.rm = TRUE)[2]) %>%
  ungroup()
```

```
# A tibble: 2 x 3
  municipality.fac range.activation.time.low range.activation.time.up
  <fct>                                <dbl>                    <dbl>
1 Caslano                                  6                       16
2 Morcote                                  6                        8
```

We utilised a Generalised Additive Model (GAM) to model the seasonal trends flexibly, without presuming a specific pattern. While this approach provides flexibility, it sacrifices some interpretability. The number of sampling dates in each municipality influenced the number of knots used in the smooth term for the seasonal trend.

Finally, to account for the lack of independence among observations from the same ovitrap, we included *unique.ID* (the unique ovitrap identifier) as a random effect. This adjustment helped to capture the variability specific to each ovitrap.

```
d.ovitraps.23 %>%
  group_by(municipality.fac) %>%
  summarise(nr.knots = n_distinct(yday)) %>%
  arrange(nr.knots)
```

```
# A tibble: 2 x 2
  municipality.fac nr.knots
  <fct>               <int>
1 Morcote                25
2 Caslano                34
```

Additionally, we will use a "point constraint" for *yday* (specifically at day 182), meaning all seasonal effects for *yday* are relative to the 1st of July.

```
pc.23 <- as.Date("2023-07-01") %>% yday()
pc.23
```

```
[1] 182
```

We first remove the observations having missing values for the relevant variables.

```
d.ovitraps.23.M.C <- d.ovitraps.23 %>%
  select(total.albopictus.egg, municipality.fac,
         Activation.time, unique.ID, Sampling_date.date,
         yday, municipality.ord) %>%
  na.omit() %>%
  droplevels()
##
```

```r
## check
dim(d.ovitraps.23)
```

```
[1] 1637   33
```

```r
dim(d.ovitraps.23.M.C)
```

```
[1] 1529    7
```

Now, we can fit the model. We use the `gamV()` function instead of `gam()`, because it fits the `gam()` model and automatically converts it to a `gamViz` object, making visualisation easier.

```r
## (this chunk is not evaluated. It takes about 1 minute to be evaluated)
##
gamm.tot.eggs.23.M.C <- gamV(total.albopictus.egg ~
                               s(yday, by = municipality.fac, pc = pc.23) +
                               municipality.fac +
                               offset(log(Activation.time)) +
                               s(unique.ID, bs = "re"),
                             family = "nb",
                             data = d.ovitraps.23.M.C)
##
saveRDS(gamm.tot.eggs.23.M.C,
        file = "Prepared_data_and_models/GAMM_tot_eggs.M.C_23.RDS")
```

We load the previously fitted model.

```r
##
gamm.tot.eggs.23.M.C <- readRDS("Prepared_data_and_models/GAMM_tot_eggs.M.C_23.RDS")
summary(gamm.tot.eggs.23.M.C)
```

```

Family: Negative Binomial(0.529)
Link function: log

Formula:
total.albopictus.egg ~ s(yday, by = municipality.fac, pc = pc.23) +
    municipality.fac + offset(log(Activation.time)) + s(unique.ID,
    bs = "re")

Parametric coefficients:
                        Estimate Std. Error z value Pr(>|z|)
(Intercept)               1.6531     0.2221   7.442 9.94e-14 ***
municipality.facMorcote  -0.8513     0.2788  -3.053  0.00226 **
---
Signif. codes:  0 '***' 0.001 '**' 0.01 '*' 0.05 '.' 0.1 ' ' 1

Approximate significance of smooth terms:
                                    edf Ref.df Chi.sq p-value
s(yday):municipality.facCaslano   6.977  7.702  275.7  <2e-16 ***
s(yday):municipality.facMorcote   7.583  8.205  265.0  <2e-16 ***
s(unique.ID)                     61.513 75.000  386.1  <2e-16 ***
---
Signif. codes:  0 '***' 0.001 '**' 0.01 '*' 0.05 '.' 0.1 ' ' 1

R-sq.(adj) =  0.561   Deviance explained = 53.3%
```

```
-REML = 5447.1  Scale est. = 1         n = 1529
```

We extract the standard deviation associated with the random effect.

```
gam.vcomp(gamm.tot.eggs.23.M.C) %>%
  tail(n = 1)
```

```
Standard deviations and 0.95 confidence intervals:

                                   std.dev      lower      upper
s(yday):municipality.facCaslano 0.02833105 0.01085469 0.07394483
s(yday):municipality.facMorcote 0.02384385 0.01231083 0.04618123
s(unique.ID)                    0.76272954 0.62586953 0.92951698

Rank: 3/3

               std.dev     lower    upper
s(unique.ID) 0.7627295 0.6258695 0.929517
```

5.2.2 Plotting the smoothers

We plot the smoothers of Caslano and Morcote together to check whether two different smoothers are necessary.

```
## Extract data from the plots
gamm.tot.eggs.23.M.C.plot.tmp <- lapply(plot(gamm.tot.eggs.23.M.C)$plots,
                                        function(x) x$data$fit)
##
gamm.tot.eggs.23.M.C.plot.tmp <- lapply(1:(length(gamm.tot.eggs.23.M.C.plot.tmp)-1),
                                        function(ii) {
                                          out <- gamm.tot.eggs.23.M.C.plot.tmp[[ii]]
                                          out$municipality <- ii
                                          return(out)
                                        } )

## Combine data in a unique data set
gamm.tot.eggs.23.M.C.plot <- do.call("rbind", gamm.tot.eggs.23.M.C.plot.tmp)
##
## Create a factor for the municipality group
gamm.tot.eggs.23.M.C.plot$municipality <- as.factor(gamm.tot.eggs.23.M.C.plot$municipality)
##
## Create CI
gamm.tot.eggs.23.M.C.plot$upper <- gamm.tot.eggs.23.M.C.plot$ty + 2 * gamm.tot.eggs.23.M.C.plot$se
gamm.tot.eggs.23.M.C.plot$lower <- gamm.tot.eggs.23.M.C.plot$ty - 2 * gamm.tot.eggs.23.M.C.plot$se
##
## Plot the data
p <- ggplot(data = gamm.tot.eggs.23.M.C.plot, mapping = aes(x = x, y = ty,
                                                            colour = municipality,
                                                            group = municipality,
                                                            fill = municipality)) +
  geom_line() +
  geom_ribbon(data = subset(gamm.tot.eggs.23.M.C.plot, lower < y & y < upper),
              aes(ymin = lower, ymax = upper),
              alpha = 0.3,
```

```
             lty = 2) +
  coord_cartesian(ylim = c(-15, 5))
p
```

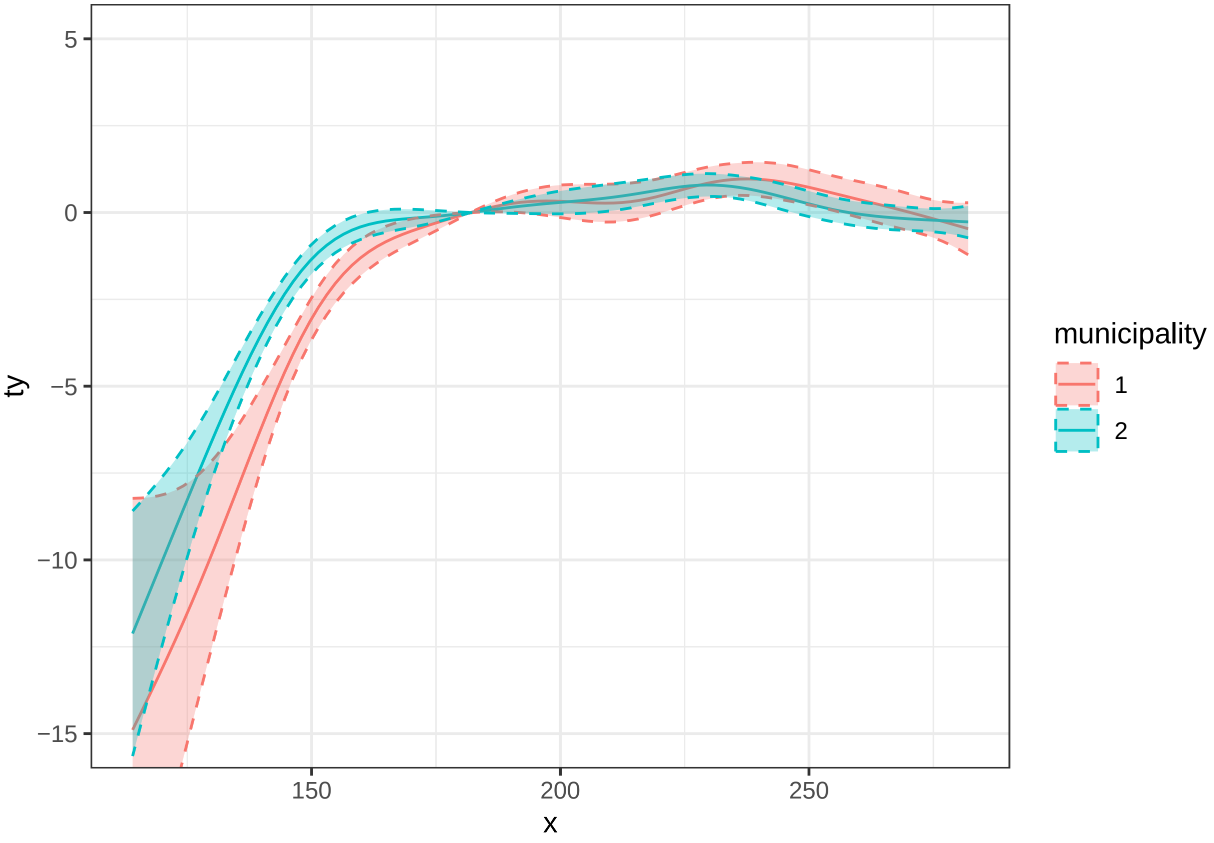


```
## save plot for future use:
saveRDS(p, file = file.path("saved_figures", "2a_plotSmooths_MC.rds"))
```

Again, it is not clear whether it is necessary to allow different shapes for the two smoothers. We will formally verify this later in the sections.

We draw again the same plots, separately, but adding the correct shift to all of them.

```
plot.gam(gamm.tot.eggs.23.M.C,
         select = 1,
         shift = coef(gamm.tot.eggs.23.M.C)["(Intercept)"],
         trans = exp,
         ylim = c(-1, 15),
         main = "Caslano")
```

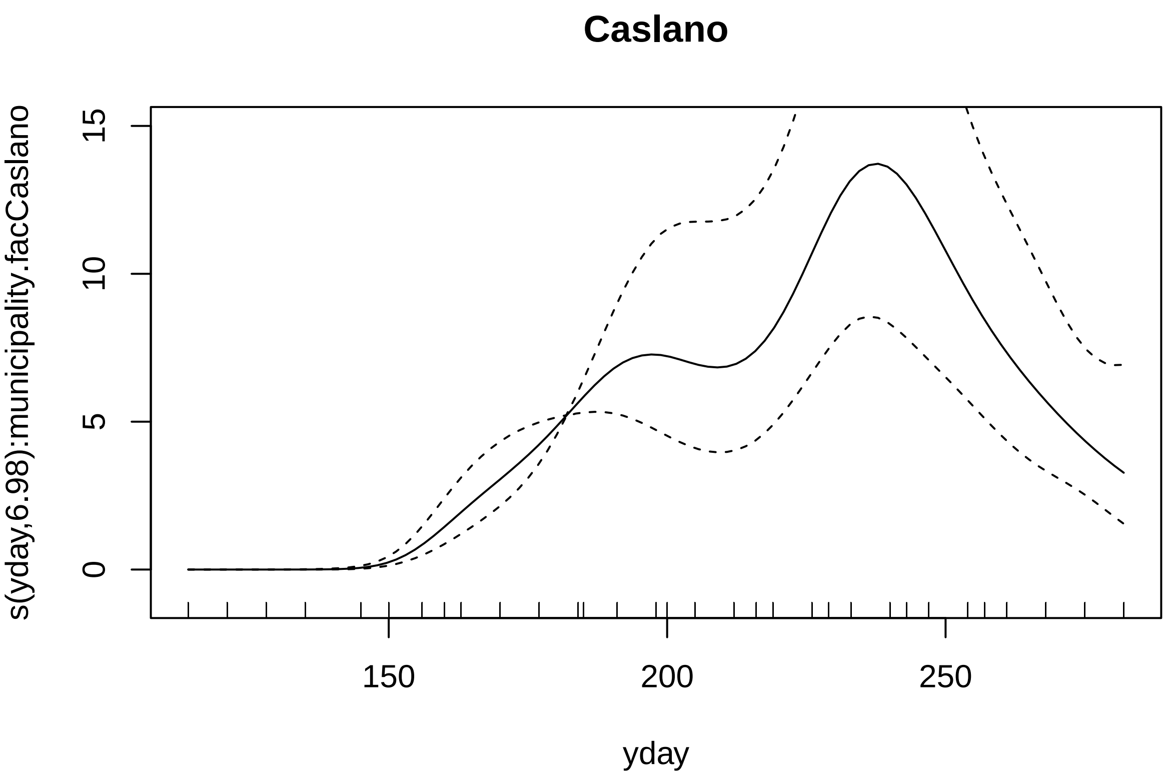


```
##
plot.gam(gamm.tot.eggs.23.M.C,
         select = 2,
         shift = coef(gamm.tot.eggs.23.M.C)["(Intercept)"] + coef(gamm.tot.eggs.23.M.C)[2],
         trans = exp,
         ylim = c(-1, 12),
         main = "Morcote")
```

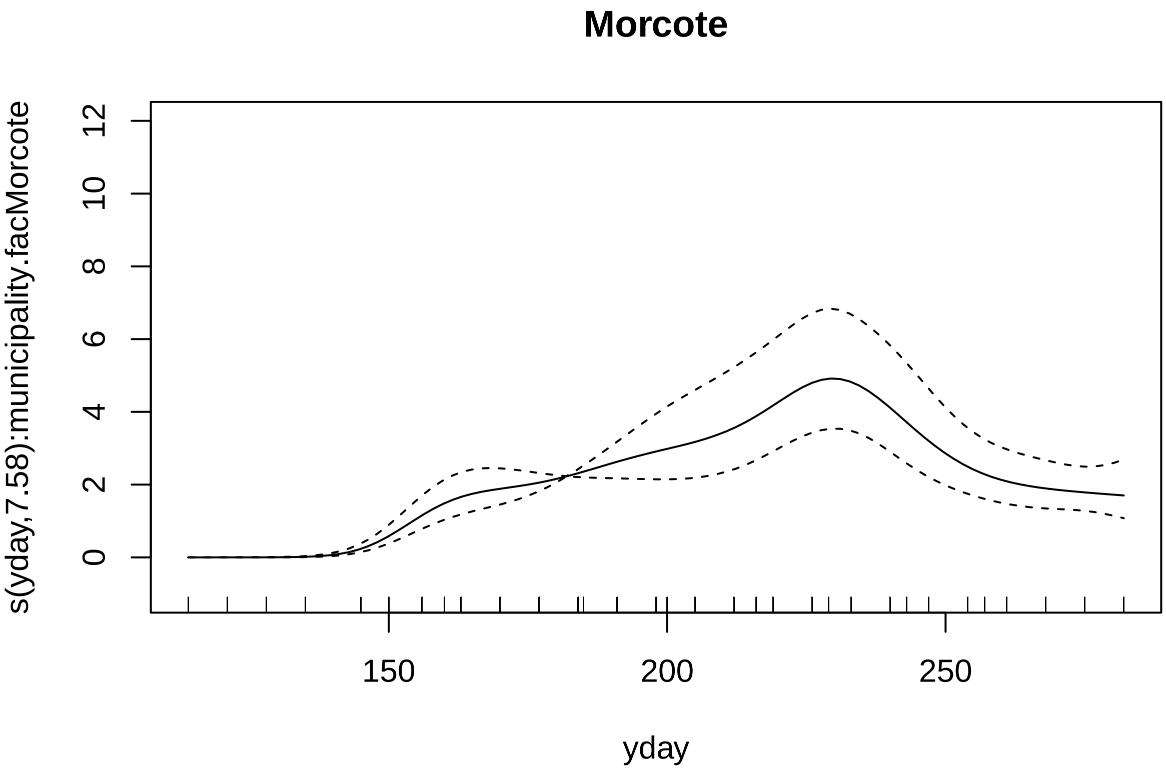

5.2.3 Fitted values

First, we begin by plotting the fitted values over time, creating a separate graph for each municipality. Observations from the same ovitrap will be connected by lines to visualise the trends.

```
d.ovitraps.23.M.C$fitted_gamm.tot.eggs.23.M.C <- fitted(gamm.tot.eggs.23.M.C)
##
p <- ggplot(data = d.ovitraps.23.M.C,
            mapping = aes(y = fitted_gamm.tot.eggs.23.M.C,
                          x = Sampling_date.date,
                          group = unique.ID)) +
  geom_hline(yintercept = 0) +
  geom_point(alpha = 0.1) +
  geom_line(alpha = 0.1) +
  scale_y_sqrt() +
  facet_wrap(~municipality.fac)
p
```

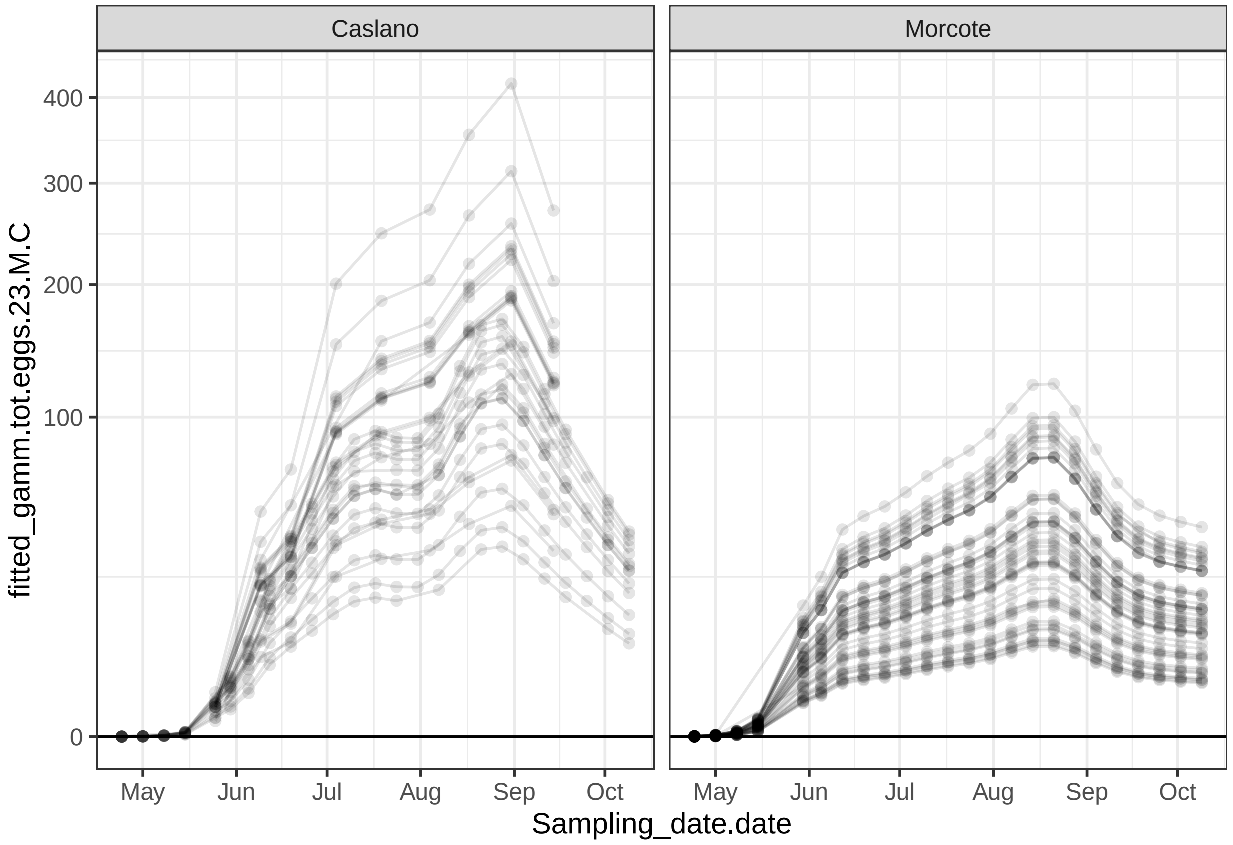


```
## save plot for future use:
saveRDS(p, file = file.path("saved_figures", "2a_fittedValeusGammTotEggs_MC.rds"))
```

Note that there is significant variability among ovitraps.

The graphs appear less "smooth" because they are based on a discrete number of observations.

5.2.4 Predicted values

Let's create the data set to make predictions on.

```
## (messages are omitted from this chunk)
##
formula(gamm.tot.eggs.23.M.C)
```

```
total.albopictus.egg ~ s(yday, by = municipality.fac, pc = pc.23) +
    municipality.fac + offset(log(Activation.time)) + s(unique.ID,
    bs = "re")
```

```
##
## 1. We create a data set with municipality.fac and ovitrap ID
d.muni.ovitrap.short <- d.ovitraps.23.M.C %>%
  select(municipality.fac, unique.ID) %>%
  unique()
##
head(d.muni.ovitrap.short)
```

```
# A tibble: 6 x 2
  municipality.fac unique.ID
```

```
  <fct>            <fct>
1 Caslano          Caslano.1
2 Caslano          Caslano.2
3 Caslano          Caslano.3
4 Caslano          Caslano.4
5 Caslano          Caslano.5
6 Caslano          Caslano.6
```

```r
nrow(d.muni.ovitrap.short)
```

```
[1] 77
```

```r
##
## 2. We create a data set with varying yday for each ovitrap ID
d.pred.gamm.23 <- expand.grid(
  yday = seq(from = min(d.ovitraps.23.M.C$yday),
             to = max(d.ovitraps.23.M.C$yday),
             length.out = 100),
  unique.ID = d.muni.ovitrap.short$unique.ID,
  ## We set the activation time equal to 14 days, i.e. two weeks.
  Activation.time = 14)
##
## 3. We join the two data sets.
## Note that not all ovitraps exist in all municipalities.
## So, we can't simply use expand.grid()
d.pred.gamm.23_aug <- left_join(d.pred.gamm.23, d.muni.ovitrap.short)
str(d.pred.gamm.23_aug)
```

```
'data.frame':   7700 obs. of  4 variables:
 $ yday            : num  114 116 117 119 121 ...
 $ unique.ID       : Factor w/ 77 levels "Caslano.1","Caslano.10",..: 1 1 1 1 1 1 1 1 1 1 ...
 $ Activation.time : num  14 14 14 14 14 14 14 14 14 14 ...
 $ municipality.fac: Factor w/ 2 levels "Caslano","Morcote": 1 1 1 1 1 1 1 1 1 1 ...
 - attr(*, "out.attrs")=List of 2
  ..$ dim     : Named int [1:3] 100 77 1
  .. ..- attr(*, "names")= chr [1:3] "yday" "unique.ID" "Activation.time"
  ..$ dimnames:List of 3
  .. ..$ yday           : chr [1:100] "yday=114.0000" "yday=115.6970" "yday=117.3939" "yday=119.0909" .
  .. ..$ unique.ID      : chr [1:77] "unique.ID=Caslano.1" "unique.ID=Caslano.2" "unique.ID=Caslano.3"
  .. ..$ Activation.time: chr "Activation.time=14"
```

We make the predictions on the newly created data set, at ovitrap and population level, and we plot the result.

```r
## prediction at ovitrap level
d.pred.gamm.23_aug$predicted_gamm.tot.eggs.23.M.C <- predict(
  gamm.tot.eggs.23.M.C,
  newdata = d.pred.gamm.23_aug,
  type = "response")
##
## prediction at population level
d.pred.gamm.23_aug$predicted_gamm.tot.eggs.23.M.C.pop <- predict(
  gamm.tot.eggs.23.M.C,
  newdata = d.pred.gamm.23_aug,
  type = "response",
  exclude = 's(unique.ID)')
```

```r
##
p <- ggplot(data = d.pred.gamm.23_aug,
            mapping = aes(y = predicted_gamm.tot.eggs.23.M.C,
                          x = yday,
                          group = unique.ID)) +
  geom_hline(yintercept = 0) +
  geom_line(alpha = 0.2) +
  scale_y_sqrt() +
  geom_line(mapping = aes(y = predicted_gamm.tot.eggs.23.M.C.pop), colour = "red") +
  facet_wrap(~municipality.fac) +
  geom_rug(data = d.ovitraps.23.M.C, mapping = aes(x = yday, y = NA), alpha = 0.5)
p
```

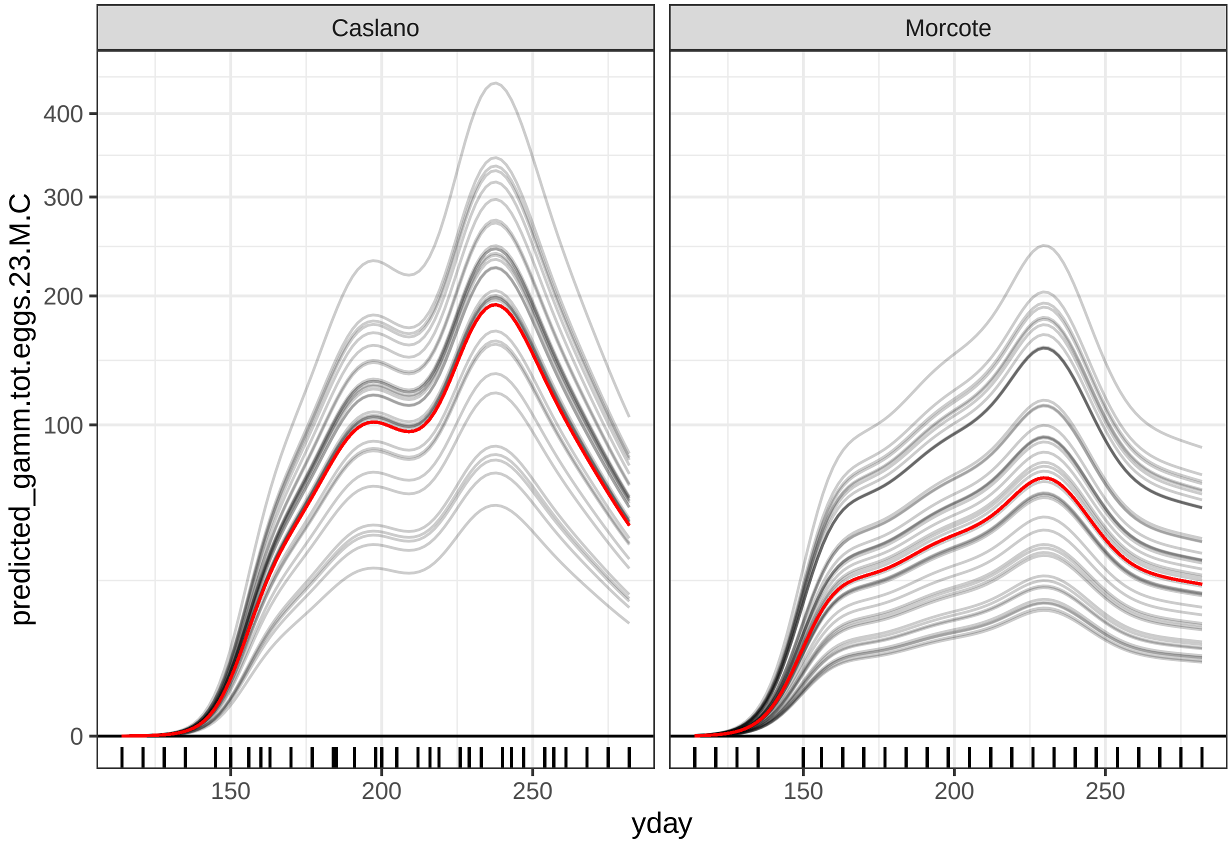


```r
## save plot for future use:
saveRDS(p, file = file.path("saved_figures",
                            "2a_MakePredictionGammTotEggs_MC.rds"))
```

Each black line corresponds to the prediction for a given ovitrap, whereas the red line corresponds to the prediction at population level.

The shapes of the trends can vary significantly between municipalities, and the variability within each municipality is also quite pronounced.

The smoothers may be a little overfitted. However, perfect inference is not paramount, so we do not try to correct it.

We plot the predictions at population level on the same plot.

```r
p <- ggplot(data = d.pred.gamm.23_aug,
            mapping = aes(y = predicted_gamm.tot.eggs.23.M.C.pop,
                          x = yday,
                          colour = municipality.fac)) +
  geom_line() +
  geom_hline(yintercept = c(0, 1))
p
```

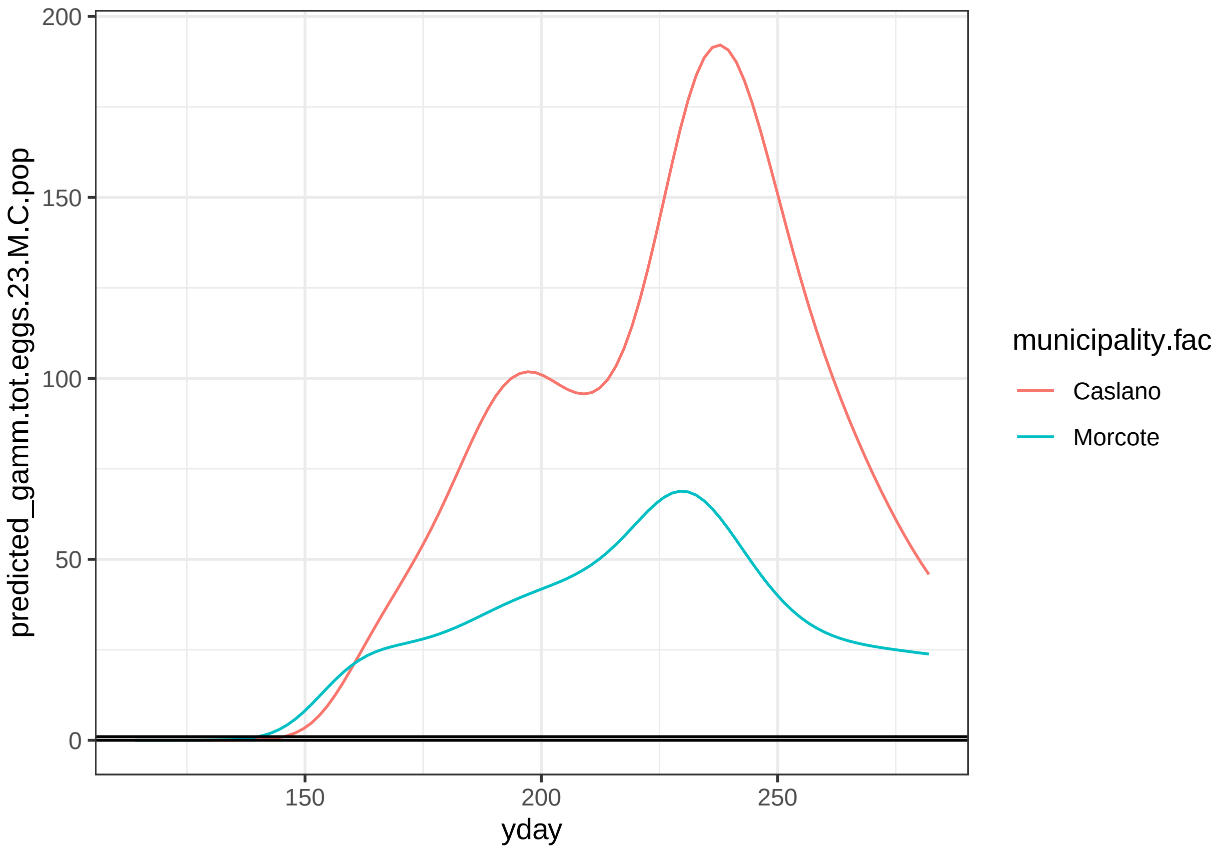


```r
## save plot for future use:
saveRDS(p, file = file.path("saved_figures",
                            "2a_predPopLevel_MC.rds"))
```

In the peak period, the number of mosquitoes in Caslano is estimated to be about three times higher than in Morcote. In general, it is estimated that the number of mosquitoes is greater in Caslano than in Morcote.

5.2.5 Model selection – Shape

We fitted a model allowing a different smoother for each municipality. Now, we will assess whether this flexibility is truly necessary.

With this purpose in mind, we refit the model, but this time without allowing for different shapes across municipalities. We will then compare the two models to determine if the difference between them is statistically significant.

For performing this comparison, we need to refit the first model using an ordered factor (*municipality.ord*) instead of the classical one. This allows us to have nested model matrices and formally compare the two models.

```r
## (this chunk is not evaluated)
gamm.tot.eggs.23.M.C.ord <- gam(
  total.albopictus.egg ~
    offset(log(Activation.time)) +
    s(unique.ID, bs = "re") +
    s(yday, pc = pc.23) +
    municipality.fac +
    s(yday, pc = pc.23, by = municipality.ord),
  family = "nb",
  data = d.ovitraps.23.M.C)
##
saveRDS(gamm.tot.eggs.23.M.C.ord,
        "Prepared_data_and_models/GAMM_tot_eggs_M_C_ord_23.RDS")
##
gamm.tot.eggs.23.M.C_one.smooth <- gam(
  total.albopictus.egg ~
    offset(log(Activation.time)) +
    s(unique.ID, bs = "re") +
    s(yday, pc = pc.23) +
    municipality.fac,
  # s(yday, pc = pc.23, by = municipality.ord),
  family = "nb",
  data = d.ovitraps.23.M.C)
##
saveRDS(gamm.tot.eggs.23.M.C_one.smooth,
        "Prepared_data_and_models/GAMM_tot_eggs_M_C_one_smooth_23.RDS")
```

Let's get the previously fitted models.

```r
gamm.tot.eggs.23.M.C.ord <- readRDS(paste0("Prepared_data_and_models/",
                                           "GAMM_tot_eggs_M_C_ord_23.RDS"))
gamm.tot.eggs.23.M.C_one.smooth <- readRDS(paste0("Prepared_data_and_models/",
                                                  "GAMM_tot_eggs_M_C_one_smooth_23.RDS"))
##
summary(gamm.tot.eggs.23.M.C.ord)
```

```

Family: Negative Binomial(0.529)
Link function: log

Formula:
total.albopictus.egg ~ offset(log(Activation.time)) + s(unique.ID,
    bs = "re") + s(yday, pc = pc.23) + municipality.fac + s(yday,
    pc = pc.23, by = municipality.ord)

Parametric coefficients:
                        Estimate Std. Error z value Pr(>|z|)
(Intercept)               1.5603     0.2032   7.678 1.62e-14 ***
municipality.facMorcote  -0.7369     0.2424  -3.040  0.00236 **
---
Signif. codes:  0 '***' 0.001 '**' 0.01 '*' 0.05 '.' 0.1 ' ' 1

Approximate significance of smooth terms:
                                   edf Ref.df Chi.sq p-value
```

```
s(unique.ID)                      61.471 75.000 385.02  <2e-16 ***
s(yday)                            7.725  8.239 311.55  <2e-16 ***
s(yday):municipality.ordMorcote  4.062  4.949  44.37  <2e-16 ***
---
Signif. codes:  0 '***' 0.001 '**' 0.01 '*' 0.05 '.' 0.1 ' ' 1

R-sq.(adj) =  0.569   Deviance explained = 53.2%
-REML = 5441.6  Scale est. = 1         n = 1529
```

```
summary(gamm.tot.eggs.23.M.C_one.smooth)
```

```

Family: Negative Binomial(0.514)
Link function: log

Formula:
total.albopictus.egg ~ offset(log(Activation.time)) + s(unique.ID,
    bs = "re") + s(yday, pc = pc.23) + municipality.fac

Parametric coefficients:
                        Estimate Std. Error z value Pr(>|z|)
(Intercept)               1.5709     0.1827   8.599  < 2e-16 ***
municipality.facMorcote  -0.7375     0.1991  -3.704 0.000212 ***
---
Signif. codes:  0 '***' 0.001 '**' 0.01 '*' 0.05 '.' 0.1 ' ' 1

Approximate significance of smooth terms:
                edf Ref.df Chi.sq p-value
s(unique.ID) 61.567 75.000  387.1  <2e-16 ***
s(yday)       7.764  8.309  441.8  <2e-16 ***
---
Signif. codes:  0 '***' 0.001 '**' 0.01 '*' 0.05 '.' 0.1 ' ' 1

R-sq.(adj) =  0.546   Deviance explained = 51.9%
-REML = 5455.7  Scale est. = 1         n = 1529
```

We check whether the two models are nested. If they are, then we can test whether the most complicated one is necessary, i.e., if the difference of the two models is statistically significant.

```
## Extract model matrices
m.gamm.tot.eggs.23.M.C.ord <- model.matrix(gamm.tot.eggs.23.M.C.ord)
m.gamm.tot.eggs.23.M.C_one.smooth <- model.matrix(gamm.tot.eggs.23.M.C_one.smooth) ##
dim(m.gamm.tot.eggs.23.M.C.ord)
```

```
[1] 1529   97
```

```
dim(m.gamm.tot.eggs.23.M.C_one.smooth)
```

```
[1] 1529   88
```

```
##
n1 <- ncol(m.gamm.tot.eggs.23.M.C_one.smooth)
## check that sum is zero
sum(matrix( m.gamm.tot.eggs.23.M.C_one.smooth[, 1:n1] - m.gamm.tot.eggs.23.M.C.ord[, 1:n1] ) != 0)
```

```
[1] 0
```

The two model matrices are nested, therefore we can now compare the two models with the Chi-square test.

```r
anova.gam(gamm.tot.eggs.23.M.C_one.smooth, gamm.tot.eggs.23.M.C.ord, test = "Chisq")
```

```
Analysis of Deviance Table

Model 1: total.albopictus.egg ~ offset(log(Activation.time)) + s(unique.ID,
    bs = "re") + s(yday, pc = pc.23) + municipality.fac
Model 2: total.albopictus.egg ~ offset(log(Activation.time)) + s(unique.ID,
    bs = "re") + s(yday, pc = pc.23) + municipality.fac + s(yday,
    pc = pc.23, by = municipality.ord)
  Resid. Df Resid. Dev     Df Deviance  Pr(>Chi)
1    1445.4      10673
2    1439.5      10632 5.9888   40.971 2.899e-07 ***
---
Signif. codes:  0 '***' 0.001 '**' 0.01 '*' 0.05 '.' 0.1 ' ' 1
```

The difference between the two models is statistically significant, which means that the additional flexibility, allowing different shapes for the smoother in each municipality, is necessary.

For good practice, we also look at AIC and BIC.

```r
AIC(gamm.tot.eggs.23.M.C.ord, gamm.tot.eggs.23.M.C_one.smooth)
```

```
                                      df      AIC
gamm.tot.eggs.23.M.C.ord        78.30986 10788.44
gamm.tot.eggs.23.M.C_one.smooth 73.21940 10819.23
```

```r
BIC(gamm.tot.eggs.23.M.C.ord, gamm.tot.eggs.23.M.C_one.smooth)
```

```
                                      df      BIC
gamm.tot.eggs.23.M.C.ord        78.30986 11206.02
gamm.tot.eggs.23.M.C_one.smooth 73.21940 11209.66
```

AIC and BIC agree that two different shapes are necessary.

5.2.6 Checking the offset

The offset makes the assumption that number of eggs is proportional to effort (i.e. "activation time"). Let's formally check this assumption by re-fitting the model without the offset.

If this assumption holds true, one can also show graphs with e.g. the "daily number of eggs".

```r
## (this chunk is not evaluated)
##
gamm.tot.eggs.23_effortSmooth <- gam(total.albopictus.egg ~
                                       s(yday, by = municipality.fac, pc = pc.23) +
                                       municipality.fac +
                                       ## k = 8 is necessary because there are only
                                       ## 8 distinct values for the variable
                                       ## *Activation.time*
                                       s(log(Activation.time), k = 8) +
                                       s(unique.ID, bs = "re"),
                                     family = "nb",
                                     data = d.ovitraps.23.M.C)
##
saveRDS(gamm.tot.eggs.23_effortSmooth,
        "Prepared_data_and_models/GAMM_tot_eggs_effort_smooth_23.RDS")
```

Let's get the previously fitted model. In this model we want to look at the estimate effect of effort (i.e. "activation time"). Note that in this model the point constraint is not used. If used the edf of the effort smoother is also affect (is about 7), but in the end is a flat line in practice.

```
gamm.tot.eggs.23_effortSmooth <- readRDS(paste0("Prepared_data_and_models/",
                                                "GAMM_tot_eggs_effort_smooth_23.RDS"))
##
summary(gamm.tot.eggs.23_effortSmooth)
```

```

Family: Negative Binomial(0.53)
Link function: log

Formula:
total.albopictus.egg ~ s(yday, by = municipality.fac, pc = pc.23) +
    municipality.fac + s(log(Activation.time), k = 8) + s(unique.ID,
    bs = "re")

Parametric coefficients:
                        Estimate Std. Error z value Pr(>|z|)
(Intercept)               3.6047     0.2453  14.696   <2e-16 ***
municipality.facMorcote  -0.7718     0.3077  -2.509   0.0121 *
---
Signif. codes:  0 '***' 0.001 '**' 0.01 '*' 0.05 '.' 0.1 ' ' 1

Approximate significance of smooth terms:
                                   edf Ref.df Chi.sq p-value
s(yday):municipality.facCaslano  7.016  7.721 273.99 < 2e-16 ***
s(yday):municipality.facMorcote  7.569  8.194 265.24 < 2e-16 ***
s(log(Activation.time))          1.000  1.001  10.57 0.00115 **
s(unique.ID)                    61.238 75.000 388.50 < 2e-16 ***
---
Signif. codes:  0 '***' 0.001 '**' 0.01 '*' 0.05 '.' 0.1 ' ' 1

R-sq.(adj) =  0.563   Deviance explained = 55.8%
-REML = 5448.4  Scale est. = 1         n = 1529
```

The estimated degrees of freedom of *Activation.time* is low, virtually 1, therefore we can remove *Activation.time* from the smooth terms. Note that we take it's log simply because the link function implicitly used here is the log. So, the results will be interpretable in the %-% scale.

```
## (this chunk is not evaluated)
##
gamm.tot.eggs.23_effort.lin <- gam(total.albopictus.egg ~
                                     s(yday, by = municipality.fac,
                                       pc = pc.23) +
                                     municipality.fac +
                                     log(Activation.time) +
                                     s(unique.ID, bs = "re"),
                                   family = "nb",
                                   data = d.ovitraps.23.M.C)
##
saveRDS(gamm.tot.eggs.23_effort.lin,
        "Prepared_data_and_models/GAMM_tot_eggs_effort_lin_23.RDS")
```

Get the previously fitted model.

```
gamm.tot.eggs.23_effort.lin <- readRDS(paste0("Prepared_data_and_models/",
                                              "GAMM_tot_eggs_effort_lin_23.RDS"))
##
( gamm.tot.eggs.23_effort.lin$coefficients["log(Activation.time)"] +
    c(-1, 1) * 2 *summary(gamm.tot.eggs.23_effort.lin)$se["log(Activation.time)"] )
```

```
[1] 0.4754388 1.9954922
```

The value 1 is contained in the confidence interval; this means that assuming proportionality is consistent with data.

5.2.7 Residual analysis

First of all, we apply the `gam.check()` function to the model, which produces some diagnostic information.

```
par(mfrow = c(2, 2))
gam.check(gamm.tot.eggs.23.M.C)
```

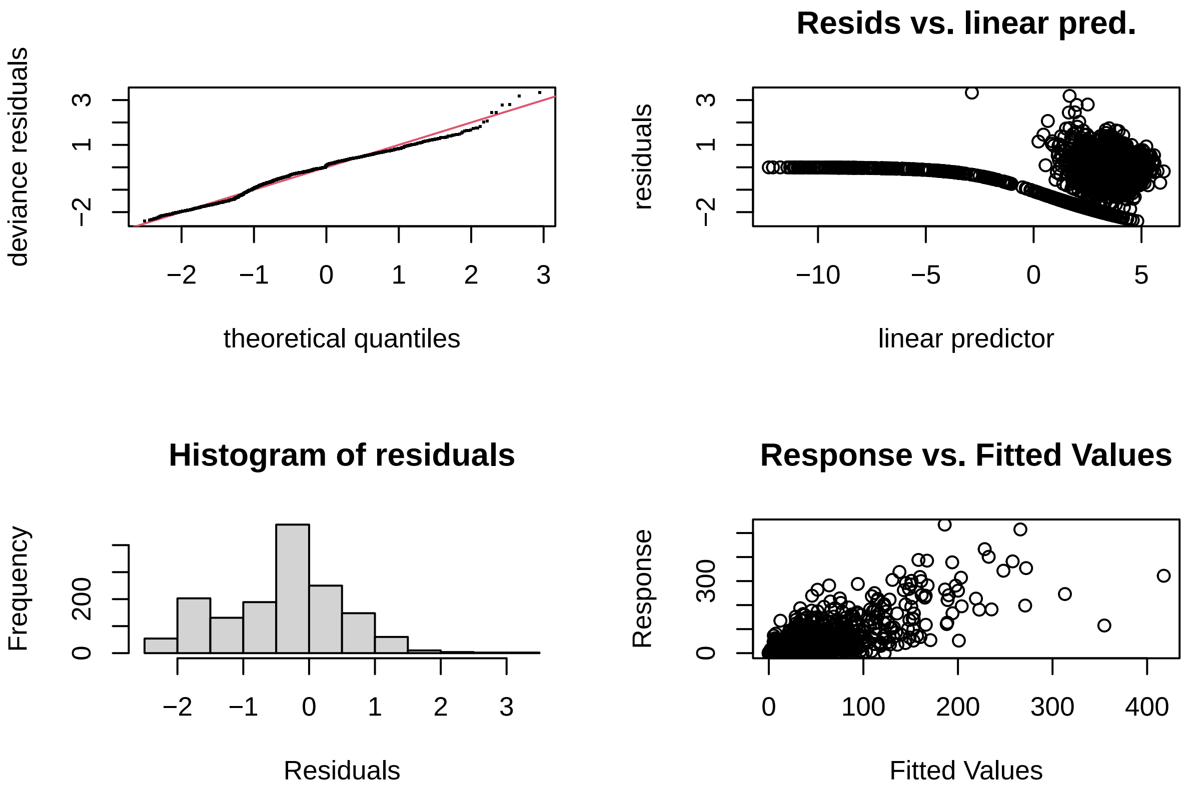


```
Method: REML   Optimizer: outer newton
full convergence after 5 iterations.
Gradient range [9.201031e-05,0.001004126]
(score 5447.064 & scale 1).
Hessian positive definite, eigenvalue range [1.040296,496.0682].
Model rank =  97 / 97

Basis dimension (k) checking results. Low p-value (k-index<1) may
indicate that k is too low, especially if edf is close to k'.
```

```
                                     k'   edf k-index p-value  
s(yday):municipality.facCaslano  9.00  6.98    0.81   0.030 *
s(yday):municipality.facMorcote  9.00  7.58    0.81   0.025 *
s(unique.ID)                    77.00 61.51      NA      NA  
---
Signif. codes:  0 '***' 0.001 '**' 0.01 '*' 0.05 '.' 0.1 ' ' 1
```

```
par(mfrow = c(1, 1))
```

Then, we store the pearson residuals in the original data frame, and we plot the residuals against the fitted values to see whether there is still structure in the data.

```
d.ovitraps.23.M.C$resid_gamm.tot.eggs.23.M.C <- resid(gamm.tot.eggs.23.M.C,
                                                       type = "pearson")
##
ggplot(data = d.ovitraps.23.M.C,
       mapping = aes(y = resid_gamm.tot.eggs.23.M.C,
                     x = fitted_gamm.tot.eggs.23.M.C)) +
  geom_hline(yintercept = 0) +
  geom_point(alpha = 0.2) +
  geom_smooth()
```

```
`geom_smooth()` using method = 'gam' and formula = 'y ~ s(x, bs = "cs")'
```

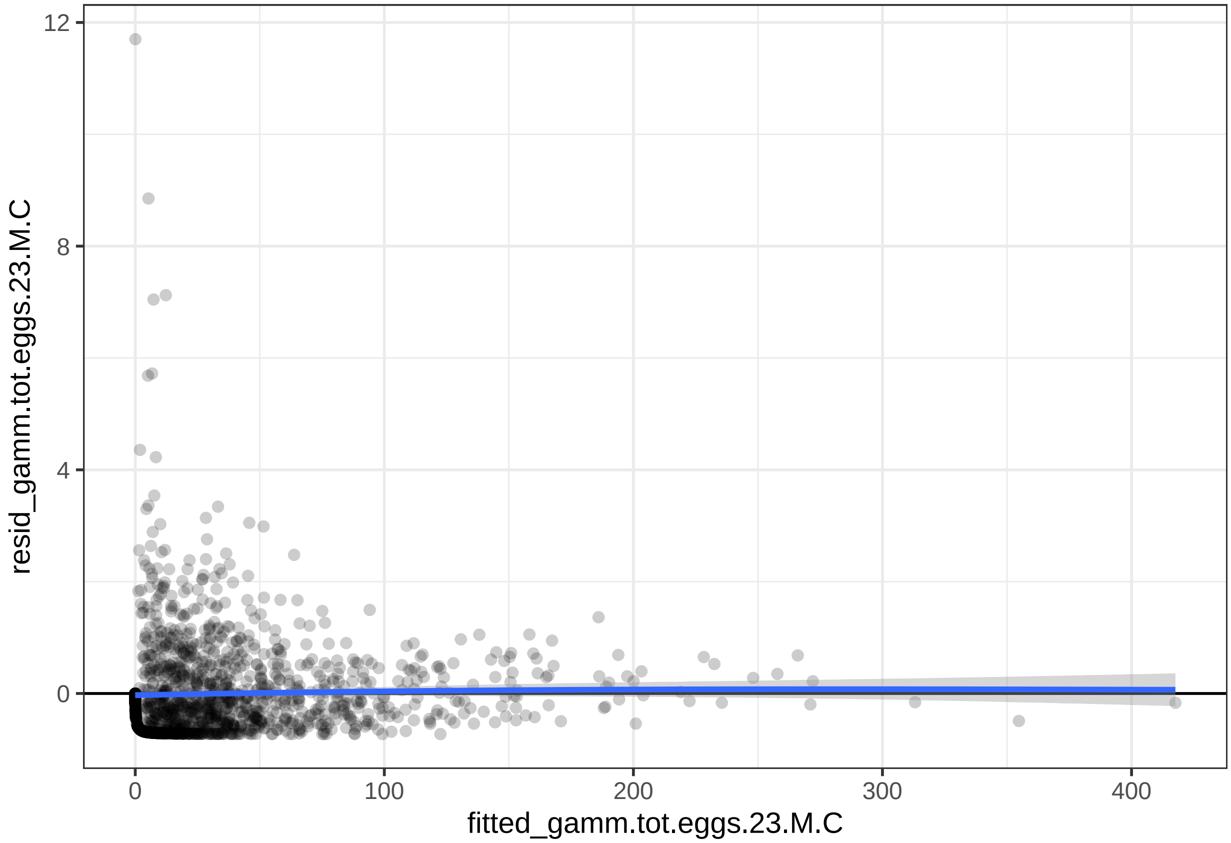

The blue line is located on the x-axis, indicating that there doesn't seem to be structure left in the residuals.

We plot the qq-plot for the random effects.

```r
## QQ for random effects
plot(sm(gamm.tot.eggs.23.M.C, 3))
```

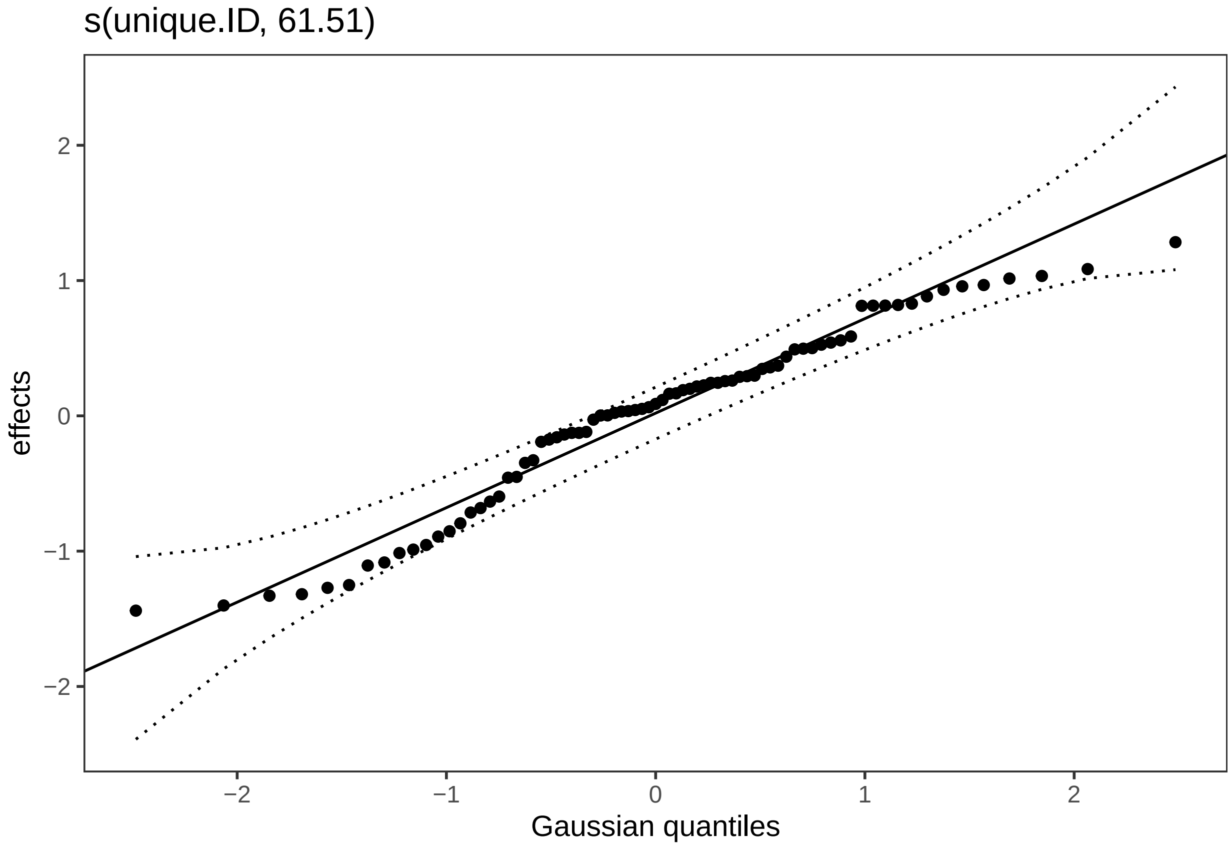

There doesn't seem to be a significant departure from the normality assumption.

We now plot the residuals over time to further check whether there is any structure left in the data.

```r
ggplot(data = d.ovitraps.23.M.C,
       mapping = aes(y = resid_gamm.tot.eggs.23.M.C,
                     x = yday)) +
  geom_hline(yintercept = 0) +
  geom_point(alpha = 0.2) +
  geom_smooth(method = "loess")
```

```
`geom_smooth()` using formula = 'y ~ x'
```

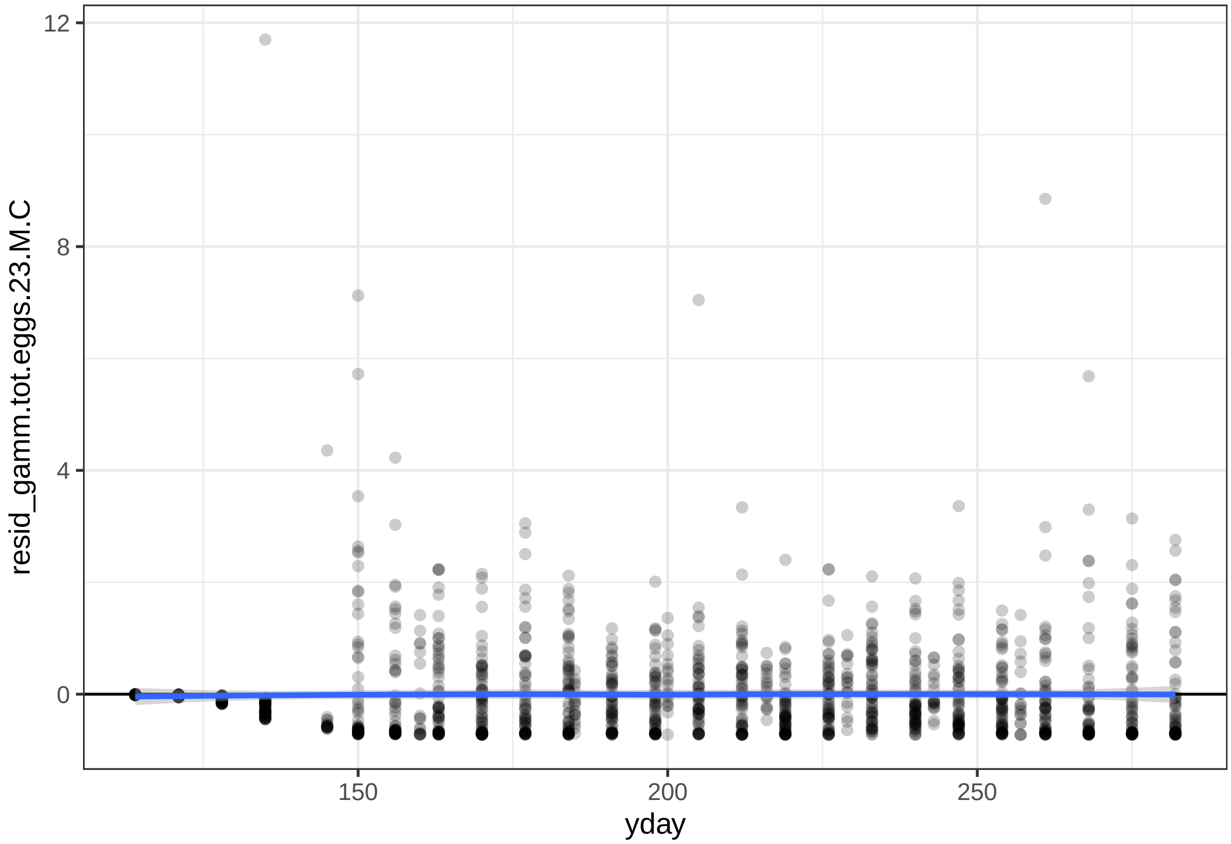

There does not seem to be any structure left in the data. Let's look into each single smoother (i.e. municipality).

```
ggplot(data = d.ovitraps.23.M.C,
       mapping = aes(y = resid_gamm.tot.eggs.23.M.C,
                     x = yday)) +
  geom_hline(yintercept = 0) +
  geom_point(alpha = 0.2) +
  geom_smooth(method = "loess") +
  facet_wrap(~municipality.fac, scales = "free")
```

```
`geom_smooth()` using formula = 'y ~ x'
```

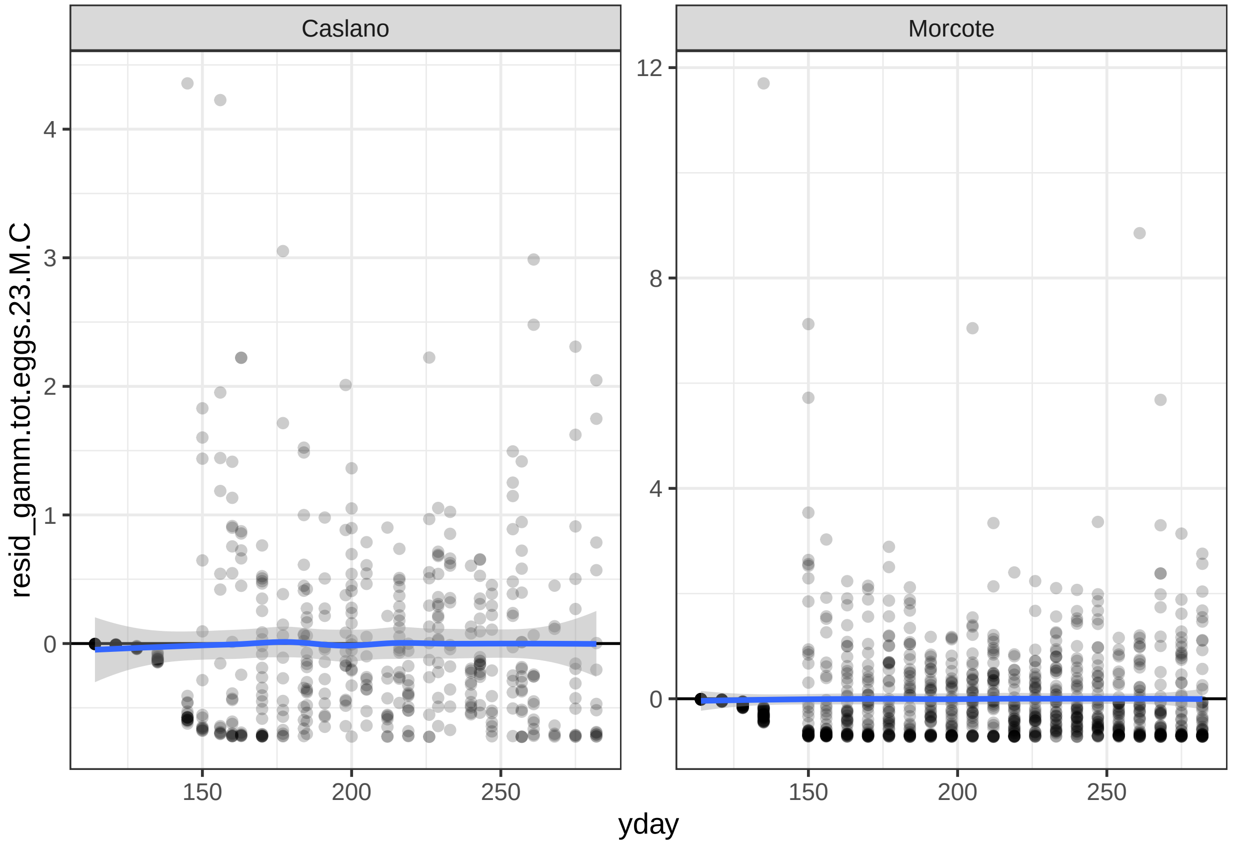

The blue lines are plotted on the x-axis (the confidence band contains it), as desired.

The following two plots display the residuals in Morcote and Caslano, respectively. The observations belonging to the same ovitrap are connected with a line. These plots are additionally drawn to check temporal correlation and variability.

```
## for Morcote
ggplot(data = filter(d.ovitraps.23.M.C,
                     municipality.fac == "Morcote"),
       mapping = aes(y = resid_gamm.tot.eggs.23.M.C,
                     x = yday,
                     group = unique.ID)) +
  geom_hline(yintercept = 0) +
  geom_point(alpha = 0.2) +
  geom_line(alpha = 0.2)
```

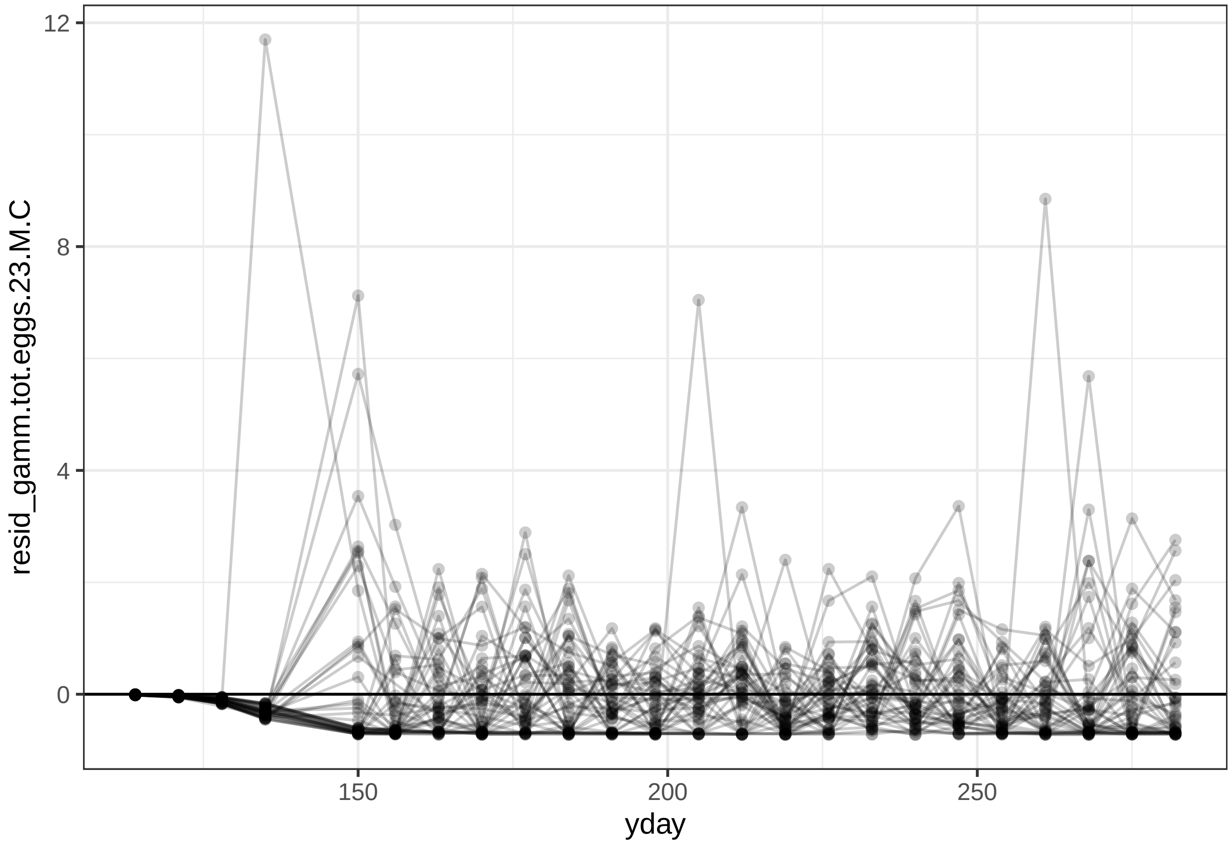


```
##
## for Caslano
ggplot(data = filter(d.ovitraps.23.M.C,
                     municipality.fac == "Caslano"),
       mapping = aes(y = resid_gamm.tot.eggs.23.M.C,
                     x = yday,
                     group = unique.ID)) +
  geom_hline(yintercept = 0) +
  geom_point(alpha = 0.2) +
  geom_line(alpha = 0.2)
```

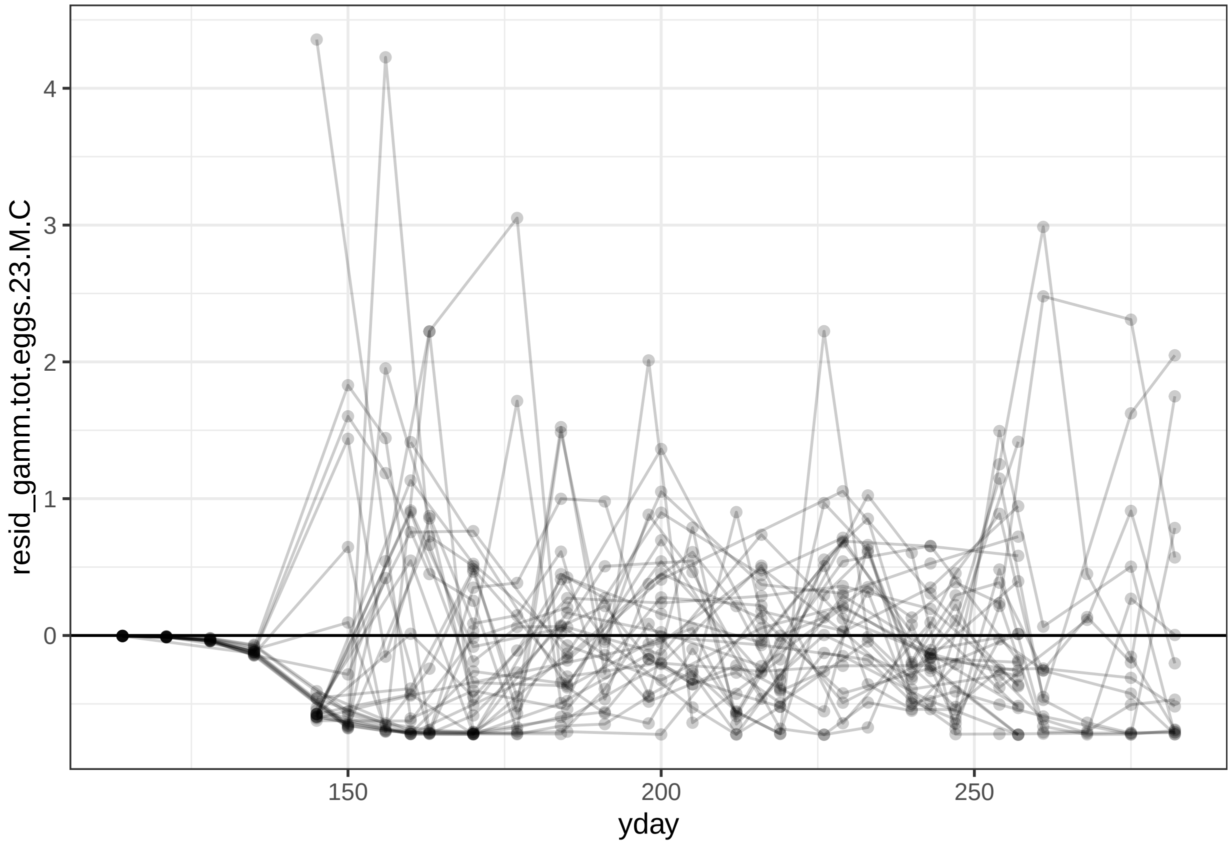

There is some high variability.

Let's look at the single ovitraps.

```
## for Morcote
ggplot(data = filter(d.ovitraps.23.M.C,
                     municipality.fac == "Morcote"),
       mapping = aes(y = resid_gamm.tot.eggs.23.M.C,
                     x = yday,
                     group = unique.ID)) +
  geom_hline(yintercept = 0) +
  geom_point() +
  geom_line() +
  facet_wrap(~unique.ID) +
  theme(
    strip.background = element_blank(),
    strip.text.x = element_blank()) +
  coord_cartesian(ylim = c(-1, 4))
```

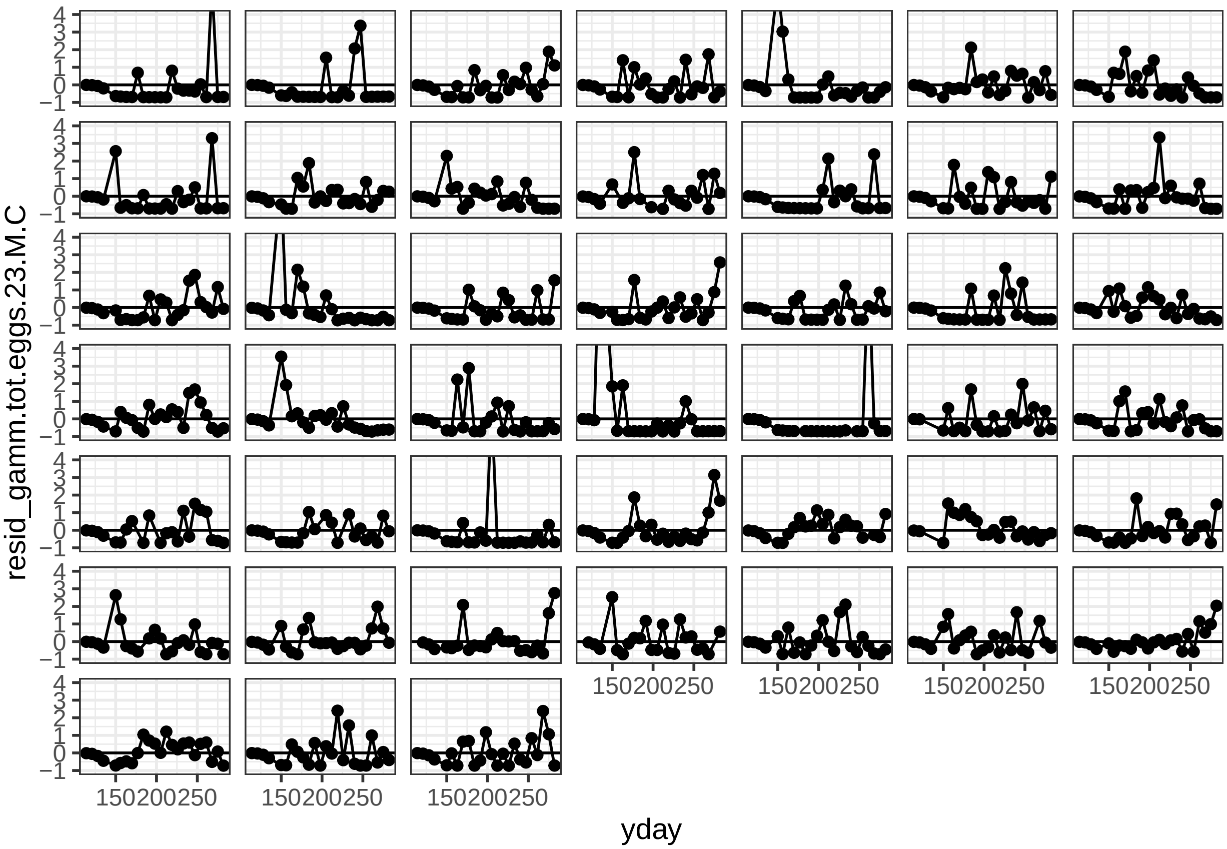


```
##
## for Caslano
ggplot(data = filter(d.ovitraps.23.M.C,
                     municipality.fac == "Caslano"),
       mapping = aes(y = resid_gamm.tot.eggs.23.M.C,
                     x = yday,
                     group = unique.ID)) +
  geom_hline(yintercept = 0) +
  geom_point() +
  geom_line() +
  facet_wrap(~unique.ID) +
  theme(
    strip.background = element_blank(),
    strip.text.x = element_blank())
```

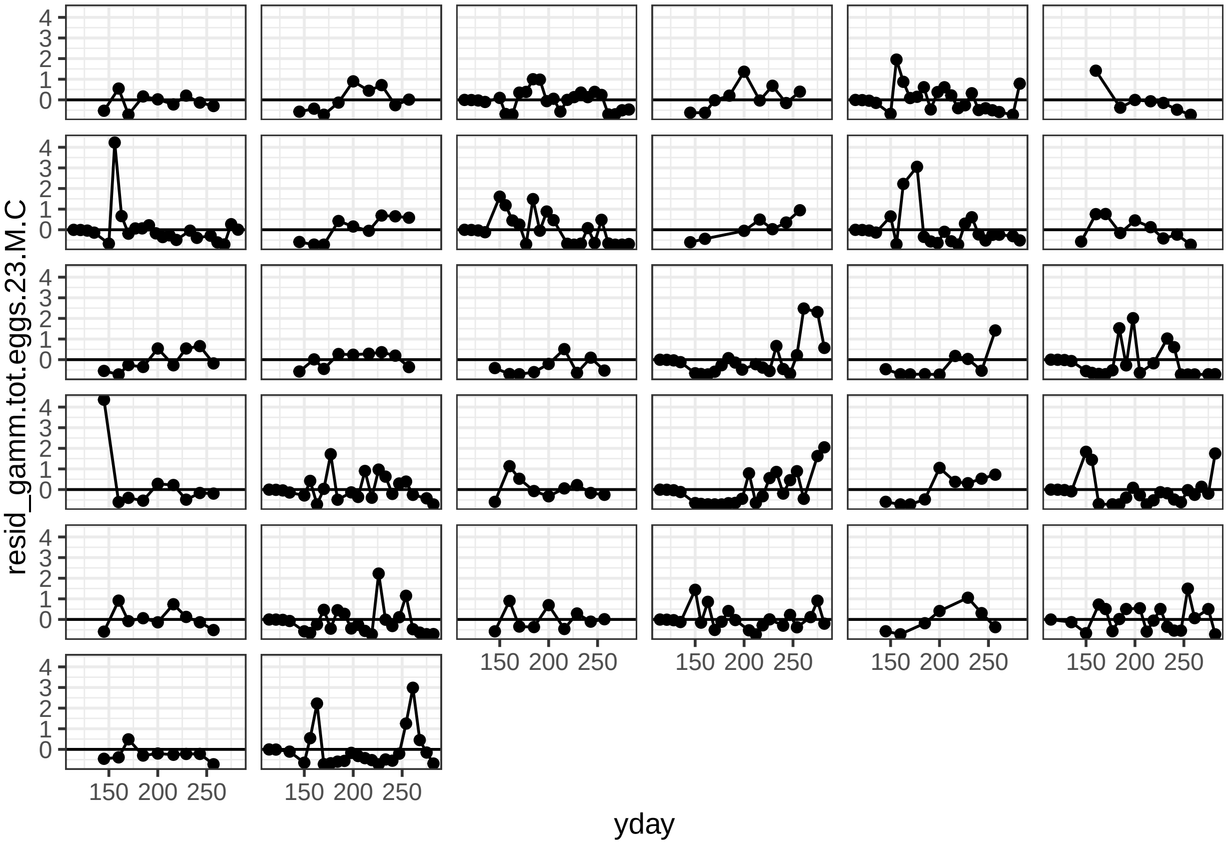

In both cases, there does not seem to be much structure left in the data.

If one were to do a formal test, there would probably be a temporal correlation. However, in this case we are more interested in modelling and understanding the effect rather than making a perfect inference, so the temporal correlation does not particularly affect our analysis.

In general, there seems to be a larger variability in Morcote.

5.2.8 Comparing (over)dispersion in the two models

In this section we formally check whether the overdispersion parameter is different for the two municipalities. With this purpose in mind, we fit two models, one for Morcote and one for Caslano, respectively.

```
## Model for Morcote
gam.tot.eggs.Morcote.23 <- gam(total.albopictus.egg ~
                                 s(yday, pc = pc.23) +
                                 offset(log(Activation.time)) +
                                 s(unique.ID, bs = "re"),
                               family = "nb",
                               data = filter(
                                 d.ovitraps.23.M.C,
                                 municipality.fac == "Morcote")) ## new element!
summary(gam.tot.eggs.Morcote.23)
```

```
Family: Negative Binomial(0.467)
Link function: log
```

```
Formula:
total.albopictus.egg ~ s(yday, pc = pc.23) + offset(log(Activation.time)) +
    s(unique.ID, bs = "re")

Parametric coefficients:
            Estimate Std. Error z value Pr(>|z|)
(Intercept)   0.7901     0.1831   4.316 1.59e-05 ***
---
Signif. codes:  0 '***' 0.001 '**' 0.01 '*' 0.05 '.' 0.1 ' ' 1

Approximate significance of smooth terms:
               edf Ref.df Chi.sq p-value
s(yday)      7.499  8.164  255.4  <2e-16 ***
s(unique.ID) 38.384 44.000  294.8  <2e-16 ***
---
Signif. codes:  0 '***' 0.001 '**' 0.01 '*' 0.05 '.' 0.1 ' ' 1

R-sq.(adj) =  0.391   Deviance explained = 49.6%
-REML = 3427.4  Scale est. = 1         n = 1061
```

```r
##
## Model for Caslano
gam.tot.eggs.Caslano.23 <- gam(total.albopictus.egg ~
                                 s(yday, pc = pc.23) +
                                 offset(log(Activation.time)) +
                                 s(unique.ID, bs = "re"),
                               family = "nb",
                               data = filter(
                                 d.ovitraps.23.M.C,
                                 municipality.fac == "Caslano")) ## new element!
summary(gam.tot.eggs.Caslano.23)
```

```
Family: Negative Binomial(0.686)
Link function: log

Formula:
total.albopictus.egg ~ s(yday, pc = pc.23) + offset(log(Activation.time)) +
    s(unique.ID, bs = "re")

Parametric coefficients:
            Estimate Std. Error z value Pr(>|z|)
(Intercept)   1.6554     0.1817   9.112   <2e-16 ***
---
Signif. codes:  0 '***' 0.001 '**' 0.01 '*' 0.05 '.' 0.1 ' ' 1

Approximate significance of smooth terms:
               edf Ref.df Chi.sq p-value
s(yday)      7.228  7.842 327.18  <2e-16 ***
s(unique.ID) 20.597 31.000  66.16  <2e-16 ***
---
Signif. codes:  0 '***' 0.001 '**' 0.01 '*' 0.05 '.' 0.1 ' ' 1

R-sq.(adj) =  0.543   Deviance explained = 55.9%
-REML = 2008.8  Scale est. = 1         n = 468
```

To compare overdispersion between the two models, we can extract the θ parameter, which controls for it. The lower the value of θ, the higher the overdispersion.

In fact, in the `gam()` model from the `{mgcv}` package, θ for negative binomial models is described to be the parameter such that

$$var(y) = \mu + \mu^2/\theta, \quad \text{where} \quad \mu = \mathbb{E}(y).$$

(see the help page of the `negbin()` function from the `{mgcv}` package).

Let's verify which model has lowest θ, i.e., highest overdispersion.

```
gam.tot.eggs.Morcote.23$family$getTheta(TRUE)
```

```
[1] 0.4672072
```

```
gam.tot.eggs.Caslano.23$family$getTheta(TRUE)
```

```
[1] 0.6855243
```

Morcote has the highest overdispersion between the two municipalities.

6 Spatial Generalised Additive Model (spatial GAM)

We are now interested in testing whether geographical location plays a role in determining the number of eggs laid.

We will only consider Morcote and Caslano because these two municipalities are the only ones sampled weekly. The others are sampled biweekly, so the comparison is not straightforward.

6.1 Visualising the data

Let us first plot the geographical distribution of the median number of eggs in Morcote. We observe the median because we are dealing with skewed data.

For this (and subsequent graphs) we use the `d.ovitraps.spatial` data set, created during data preparation. This data set contains the median number of eggs for the municipalities of interest during the season.

```
## (warnings are omitted from this chunk)
##
## Create two separate data sets, one for each municipality
d.ovitraps.spatial.caslano <- d.ovitraps.spatial %>%
  filter(municipality.fac.spatial == "Caslano" &
           ## There are traps in Caslano that are sampled bi-weekly
           ## (they came from a different data set).
           ## We filter those observations out.
           !is.na(X.num) &
           !is.na(Y.num) &
           Year.fac == 2023)
##
d.ovitraps.spatial.morcote <- d.ovitraps.spatial %>%
  filter(municipality.fac.spatial == "Morcote" &
           !is.na(X.num) &
           !is.na(Y.num) &
           Year.fac == 2023)
##
## We calculate the min and max for the median number of eggs.
## This is used in the plots, to maintain the same colour scale for the two
## municipalities and compare them more easily.
max.eggs.median <- max(c(d.ovitraps.spatial.caslano$median.eggs,
                         d.ovitraps.spatial.morcote$median.eggs))
##
min.eggs.median <- min(c(d.ovitraps.spatial.caslano$median.eggs,
                         d.ovitraps.spatial.morcote$median.eggs))
##
## Plot Morcote
p <- ggplot(d.ovitraps.spatial.morcote,
            mapping = aes(y = Y.num,
                          x = X.num,
                          colour = median.eggs)) +
  geom_point(size = 3) +
  scale_color_gradientn(colours = c("blue", "purple", "red"),
                        values = scales::rescale(c(min.eggs.median,
                                                   max.eggs.median)),
                        limits = c(min.eggs.median, max.eggs.median)) +
  # theme(aspect.ratio = 1) +
```

```r
  coord_fixed() +
  # theme(panel.grid.major = element_line(linewidth = 1)) +
  labs(title = "Morcote")
p
```

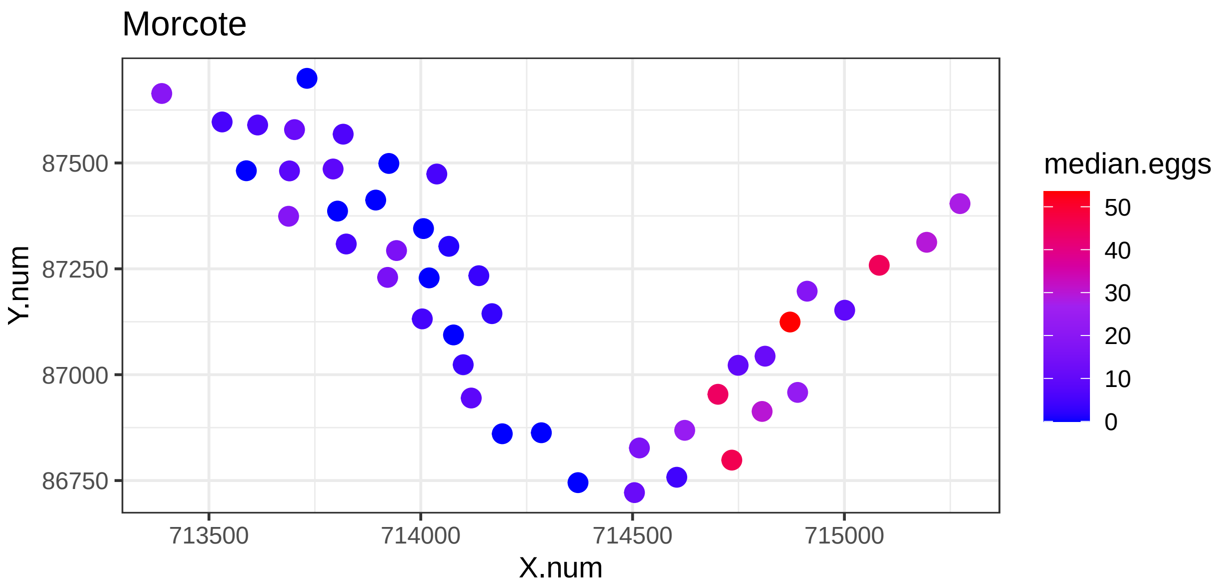


```r
## save plot for future use:
saveRDS(p, file = file.path("saved_figures",
                            "2a_meanEgssOverSpaceMorcote_graphForPaper.rds"))
```

As expected, there is a very clear spatial pattern. In fact, the right-hand side of Morcote shows a higher median count of mosquito eggs compared to the left-hand side. Moreover, the right-hand side also exhibits greater variability. This side of Morcote could be influenced by Vico Morcote, which is not treated. Therefore, there could be an exchange of mosquito males between the two municipalities. In contrast, the left-hand side of Morcote has no neighbouring municipality, making it more isolated. However, this difference might be due to other unkown factors.

We plot the release points on this map:

```r
d.release.points.morcote <-
  read_excel("../Original_data/Coordinate_Release_points.xlsx")
head(d.release.points.morcote)
```

```
# A tibble: 6 x 4
  Municipality Release_point LV03E      LV03N
  <chr>                <dbl> <chr>      <chr>
1 Morcote                  1 715'380.43 87'541.29
2 Morcote                  2 715'324.64 87'466.31
3 Morcote                  3 715'265.00 87'393.70
4 Morcote                  4 715'208.52 87'319.81
5 Morcote                  5 715'129.26 87'268.15
6 Morcote                  6 715'059.20 87'201.06
```

```r
str(d.release.points.morcote)
```

```
tibble [75 x 4] (S3: tbl_df/tbl/data.frame)
 $ Municipality : chr [1:75] "Morcote" "Morcote" "Morcote" "Morcote" ...
 $ Release_point: num [1:75] 1 2 3 4 5 6 7 8 9 10 ...
```

```
 $ LV03E        : chr [1:75] "715'380.43" "715'324.64" "715'265.00" "715'208.52" ...
 $ LV03N        : chr [1:75] "87'541.29" "87'466.31" "87'393.70" "87'319.81" ...
```

```
d.release.points.morcote <- d.release.points.morcote %>%
  mutate(LV03_E = as.numeric(gsub("'", "", LV03E)),
         LV03_N = as.numeric(gsub("'", "", LV03N)))
str(d.release.points.morcote)
```

```
tibble [75 x 6] (S3: tbl_df/tbl/data.frame)
 $ Municipality : chr [1:75] "Morcote" "Morcote" "Morcote" "Morcote" ...
 $ Release_point: num [1:75] 1 2 3 4 5 6 7 8 9 10 ...
 $ LV03E        : chr [1:75] "715'380.43" "715'324.64" "715'265.00" "715'208.52" ...
 $ LV03N        : chr [1:75] "87'541.29" "87'466.31" "87'393.70" "87'319.81" ...
 $ LV03_E       : num [1:75] 715380 715325 715265 715209 715129 ...
 $ LV03_N       : num [1:75] 87541 87466 87394 87320 87268 ...
```

```
## Plot Morcote
p <- ggplot(d.ovitraps.spatial.morcote,
            mapping = aes(y = Y.num,
                          x = X.num,
                          colour = median.eggs)) +
  geom_point(size = 3) +
  scale_color_gradientn(colours = c("blue", "purple", "red"),
                        values = scales::rescale(c(min.eggs.median,
                                                   max.eggs.median)),
                        limits = c(min.eggs.median, max.eggs.median)) +
  labs(title = "Morcote") +
  geom_point(data = d.release.points.morcote,
             mapping = aes(x = LV03_E, y = LV03_N),
             colour = "black", size = 1, pch = 4) +
    coord_fixed()
p
```

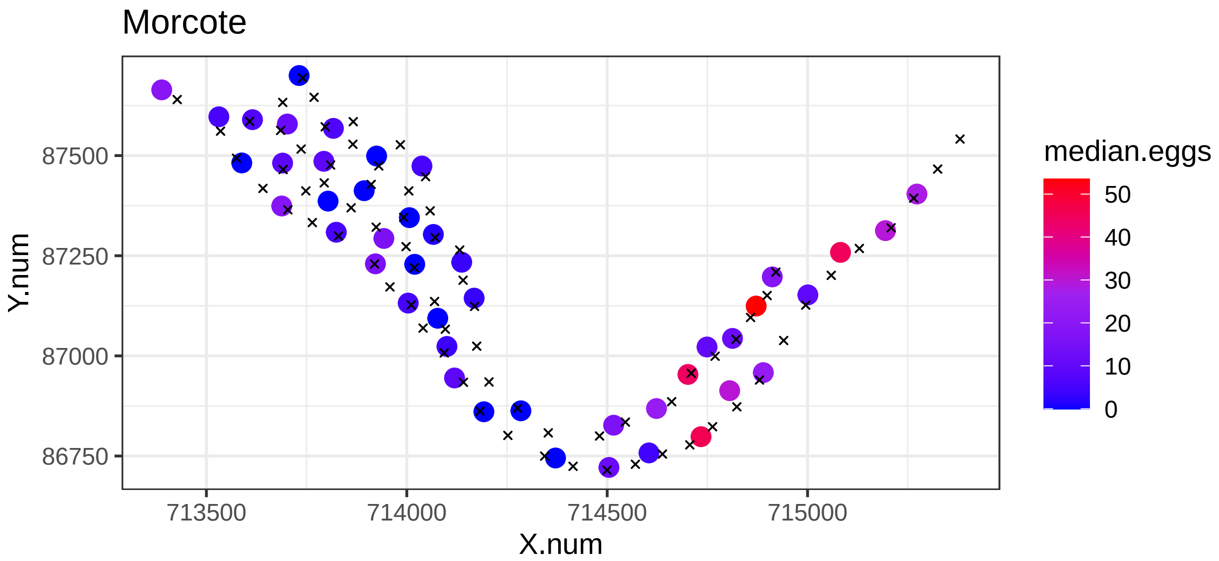


```
## save plot for future use:
saveRDS(p, file = file.path("saved_figures",
                            "2a_release_points.rds"))
```

We draw the same plot for Caslano.

```
## (warnings are omitted from this chunk)
##
p <- ggplot(d.ovitraps.spatial.caslano,
            mapping = aes(y = Y.num,
                          x = X.num,
                          colour = median.eggs)) +
  geom_point(size = 3) +
  scale_color_gradientn(colours = c("blue", "purple", "red"),
                        values = scales::rescale(c(min.eggs.median, max.eggs.median)),
                        limits = c(min.eggs.median, max.eggs.median)) +
  # theme(aspect.ratio = 1) +
  coord_fixed() +

  labs(title = "Caslano")
p
```

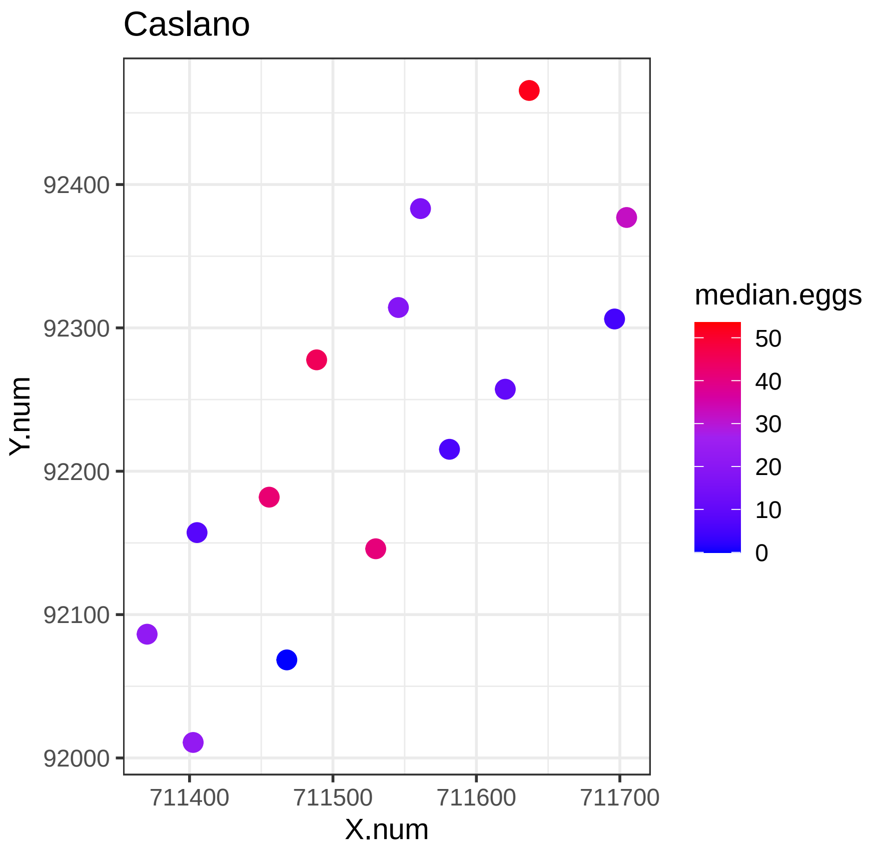


```
## save plot for future use:
saveRDS(p, file = file.path("saved_figures",
                            "2a_meanEgssOverSpaceCaslano_graphForPaper.rds"))
```

There are also some relevant spatial differences. However, there is no clear structure. For example, the lowest and highest median number of eggs are present in contiguous ovitraps.

6.2 Fitting the models

We create two data sets containing only Caslano and Morcote, respectively.

```r
d.ovitraps.23.tmp <- d.ovitraps.23 %>%
  select(`total.albopictus.egg`, `Activation.time`,
         municipality.fac.spatial, unique.ID,
         Sampling_date.date, yday,
         X.num, Y.num) %>%
  na.omit() %>%
  droplevels()
##
d.ovitraps.23.caslano <- d.ovitraps.23.tmp %>%
  filter(municipality.fac.spatial == "Caslano") %>%
  droplevels()
##
d.ovitraps.23.morcote <- d.ovitraps.23.tmp %>%
  filter(municipality.fac.spatial == "Morcote") %>%
  droplevels()
##
## check
unique(d.ovitraps.23.caslano$municipality.fac.spatial)
```

```
[1] Caslano
Levels: Caslano
```

```r
unique(d.ovitraps.23.morcote$municipality.fac.spatial)
```

```
[1] Morcote
Levels: Morcote
```

We fit two separate models, one for Morcote and one for Caslano.

We aim to model the total number of laid *Aedes albopictus* eggs (*total.albopictus.eggs*) over the season (*yday*) and territory (*X.num*, *Y.num*).

yday will be included as numeric variable; the same holds true for *X.num* and *Y.num*, which represent the swiss coordinates.

Given that *total.albopictus.eggs* represents count data, we will use a negative binomial model, which is appropriate for handling overdispersion in count data.

We don't want to assume any specific seasonal pattern, therefore we will fit a Generalised Additive Model (GAM), which provides flexibility in determining the best shape for the seasonal trend in the given municipality. However, this flexibility comes at the cost of interpretability.

We use a point-constrained for *yday* to the 1st of July.

```r
pc.23
```

```
[1] 182
```

Let's fit the model for Morcote.

Note that we use the element `s(X.num, Y.num)` to plot spatial data. We can use this isotropic smoothing because both predictors are spatial coordinates and we assume the spatial effect is isotropic (i.e., the same in all directions) and the two variables are on the same scale.

```r
## (this chunk is not evaluated)
##
gam.tot.eggs_space.Morcote.23 <- gamV(total.albopictus.egg ~
                                        s(yday, pc = pc.23) +
                                        ## Activation.time is almost always the
                                        ## same (and there isn't much difference
```

```r
                                          ## between 6, 7, 8 values, therefore
                                          ## we can comment it out)
                                          # Activation.time +
                                          s(X.num, Y.num), ## new element!

                                        family = "nb",
                                        data = d.ovitraps.23.morcote)
##
saveRDS(gam.tot.eggs_space.Morcote.23,
        file = "Prepared_data_and_models/GAM_tot_eggs_space_Morcote_23.RDS")
```

Let's get the previously fitted model.

```r
gam.tot.eggs_space.Morcote.23 <- readRDS(paste0("Prepared_data_and_models/",
                                                "GAM_tot_eggs_space_Morcote_23.RDS"))
##
summary(gam.tot.eggs_space.Morcote.23)
```

```

Family: Negative Binomial(0.447)
Link function: log

Formula:
total.albopictus.egg ~ s(yday, pc = pc.23) + s(X.num, Y.num)

Parametric coefficients:
            Estimate Std. Error z value Pr(>|z|)
(Intercept)   2.7941     0.1316   21.23   <2e-16 ***
---
Signif. codes:  0 '***' 0.001 '**' 0.01 '*' 0.05 '.' 0.1 ' ' 1

Approximate significance of smooth terms:
                  edf Ref.df Chi.sq p-value
s(yday)         7.587  8.201  236.9  <2e-16 ***
s(X.num,Y.num) 24.051 27.570  246.9  <2e-16 ***
---
Signif. codes:  0 '***' 0.001 '**' 0.01 '*' 0.05 '.' 0.1 ' ' 1

R-sq.(adj) =  0.367   Deviance explained = 47.3%
-REML =   3432  Scale est. = 1         n = 1061
```

Let's visualise the results.

```r
plot.gam(gam.tot.eggs_space.Morcote.23,
         ## Here we don't add the shift because it would be wrong.
         ## Infact the intercept also contains the coordinates.
         select = 1,
         trans = exp)
```

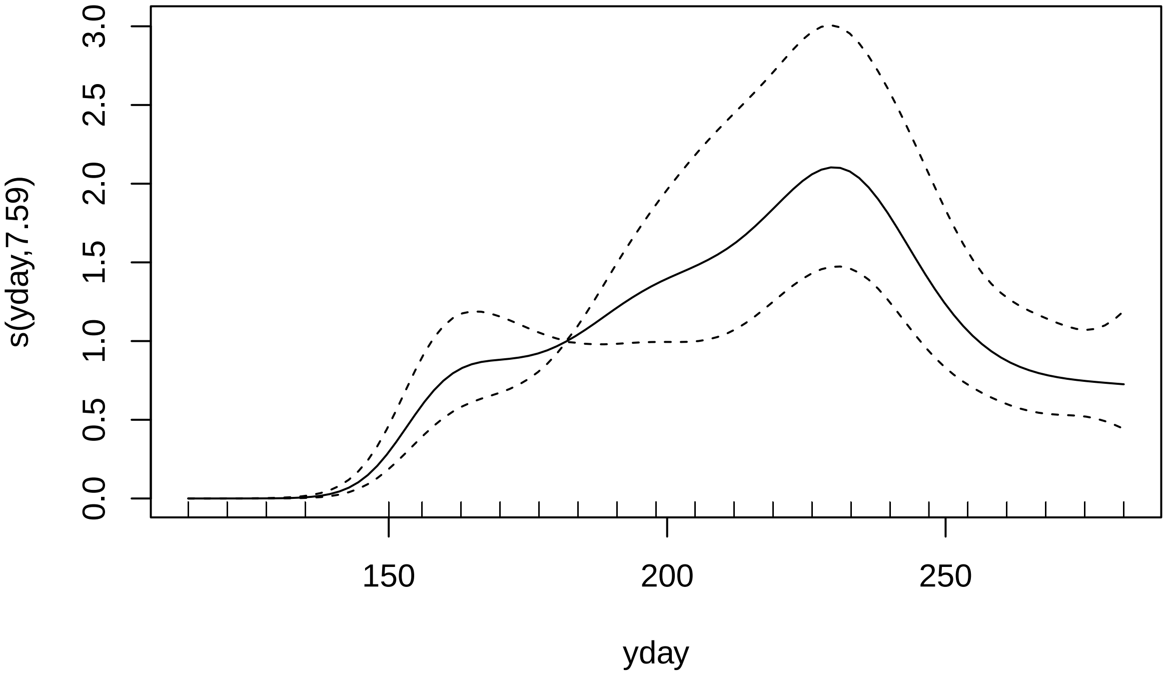


```r
##
plot.gamViz(gam.tot.eggs_space.Morcote.23,
            select = 2,
            trans = exp) +
  coord_fixed()
```

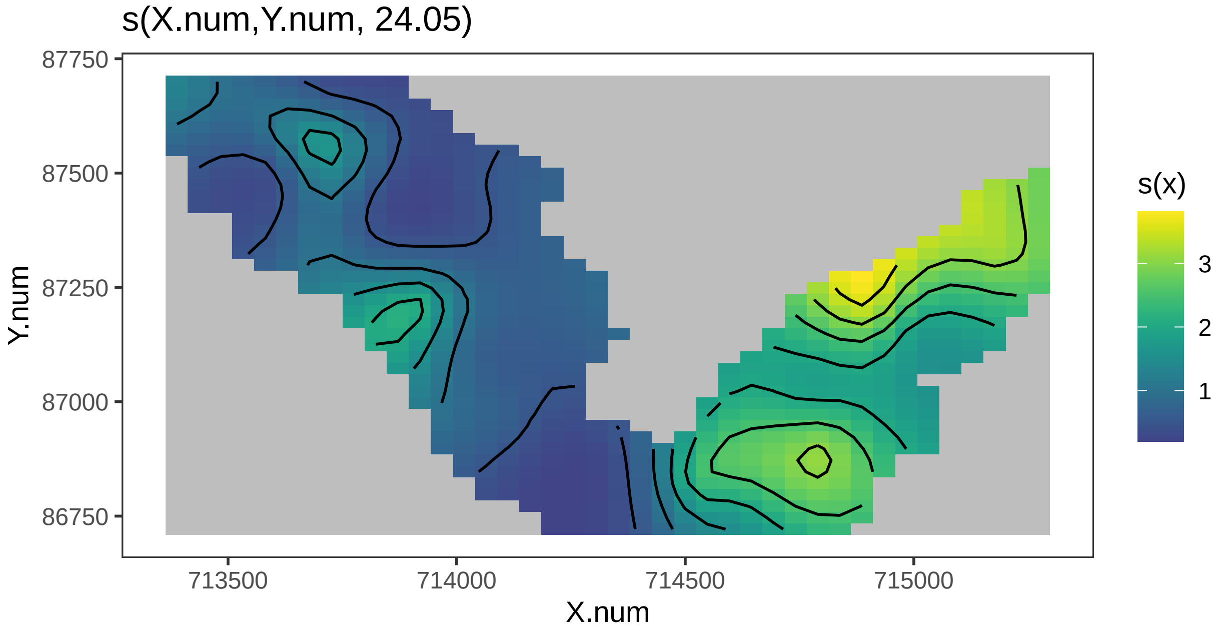

The impact of the treatment is quite evident from this graph. In particular, the isolated area of Morcote shows a sharp reduction in the estimated number of eggs laid, while the right-hand side, closer to Vico Morcote, exhibits a higher number of laid eggs. In fact, moving towards Vico Morcote, the number of laid eggs increases to nearly four times the amount observed on the left side of Morcote.

Furthermore, there does not appear to be any noticeable border effects. If such effects were present, we would expect to see a lower number of laid eggs in the center of the plot and a higher count along the edges.

We refit the same model for Caslano. For Caslano we have less unique coordinates, therefore we need to adapt k before fitting the model.

Let's see how large can be k for the bi-dimensional smoother.

```
d.ovitraps.spatial %>%
  filter(municipality.fac.spatial == "Caslano") %>%
  select(X.num, Y.num) %>%
  unique() %>%
  nrow()
```

```
[1] 20
```

Let's incorporate this information in the model call.

```
gam.tot.eggs_space.Caslano.23 <- gamV(total.albopictus.egg ~
                                          s(yday, pc = pc.23) +
                                          ## Activation.time is almost always the
                                          ## same (and there isn't much difference
                                          ## between 6, 7, 8 values, therefore
                                          ## we can comment it out)
                                          # Activation.time +
                                          s(X.num, Y.num, k = 14),  ## new element!
                                        family = "nb",
                                        data = d.ovitraps.23.caslano) ## new element!
##
saveRDS(gam.tot.eggs_space.Caslano.23,
        file = "Prepared_data_and_models/gam.tot.eggs_space.Caslano_23.RDS")
```

Let's get the previously fitted model.

```
gam.tot.eggs_space.Caslano.23 <- readRDS(paste0("Prepared_data_and_models/",
                                                "gam.tot.eggs_space.Caslano_23.RDS"))
##
summary(gam.tot.eggs_space.Caslano.23)
```

```

Family: Negative Binomial(0.615)
Link function: log

Formula:
total.albopictus.egg ~ s(yday, pc = pc.23) + s(X.num, Y.num,
    k = 14)

Parametric coefficients:
            Estimate Std. Error z value Pr(>|z|)
(Intercept)   3.7930     0.1909   19.87   <2e-16 ***
---
Signif. codes:  0 '***' 0.001 '**' 0.01 '*' 0.05 '.' 0.1 ' ' 1

Approximate significance of smooth terms:
                 edf Ref.df Chi.sq  p-value
s(yday)        6.487  7.446 155.03  < 2e-16 ***
s(X.num,Y.num) 9.009 11.091  31.75 0.000894 ***
---
Signif. codes:  0 '***' 0.001 '**' 0.01 '*' 0.05 '.' 0.1 ' ' 1
```

```
R-sq.(adj) =  0.315   Deviance explained = 56.6%
-REML =   1215  Scale est. = 1         n = 312
```

Let's visualise the results.

```r
plot.gam(gam.tot.eggs_space.Caslano.23,
         ## Here we don't add the shift because it would be wrong.
         ## Infact the intercept also contains the coordinates.
         select = 1,
         trans = exp)
```

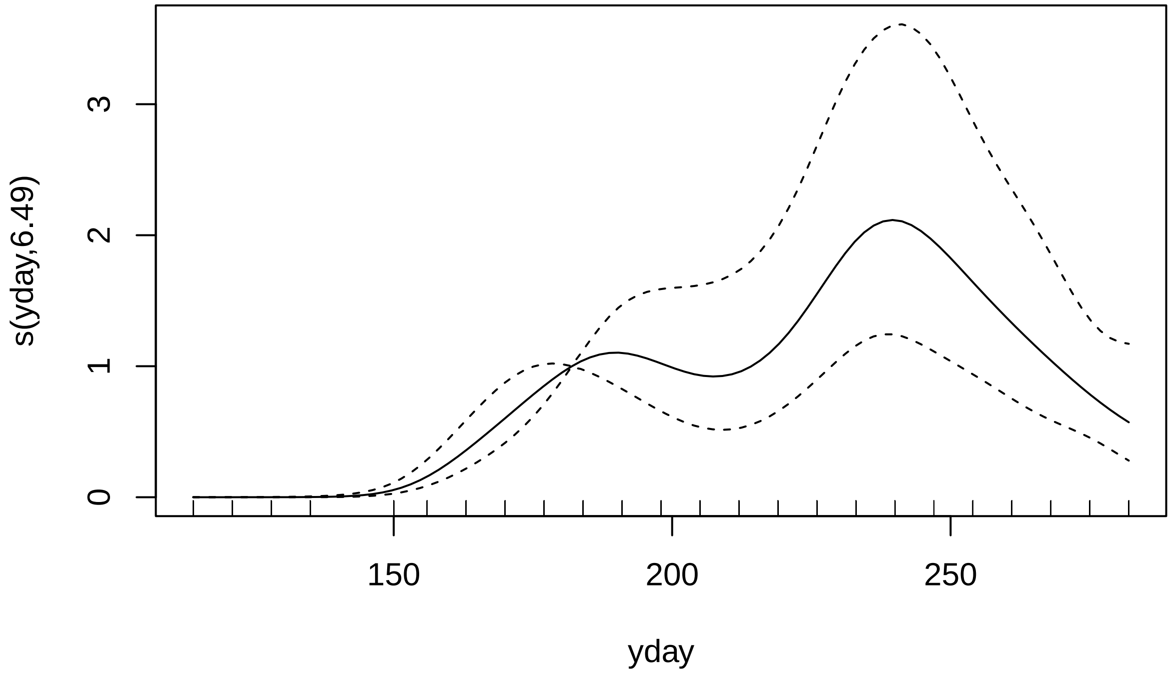


```r
##
plot.gamViz(gam.tot.eggs_space.Caslano.23,
            select = 2,
            trans = exp) +
  coord_fixed()
```

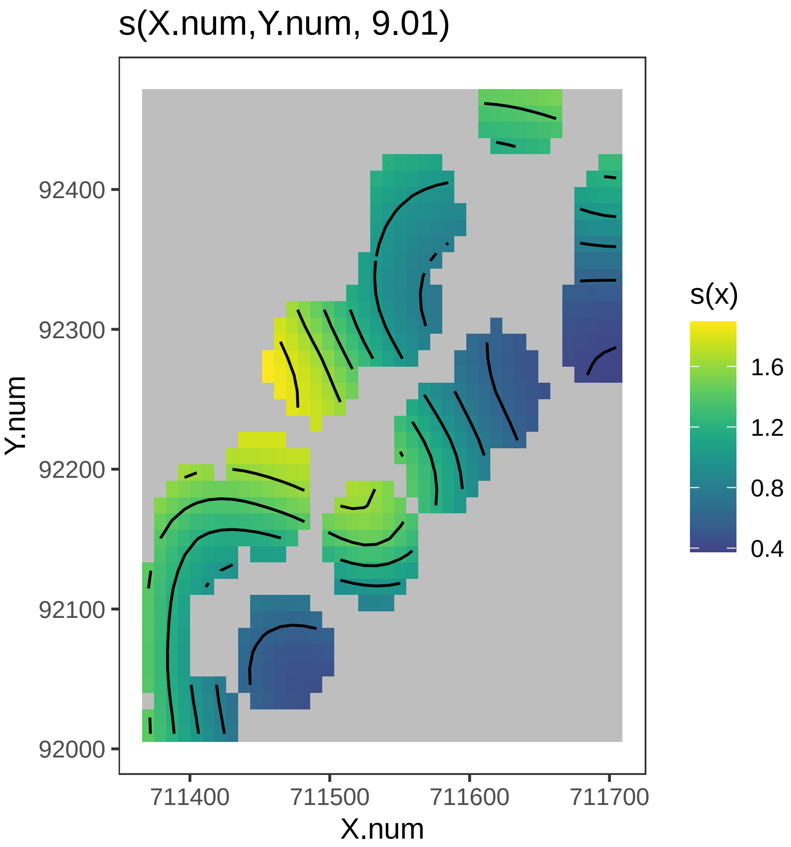

Apparently, the traps' density is not high enough to estimate the entire surface.

Internal comment: (relevant for SIT design).

Note also that the scale shows that the spatial variation is far less than in Morcote. In Morcote the scale goes up to 4 times more than average. Here at most about twice as many eggs compared to the average.

6.3 Residual analysis

First of all, we apply the `gam.check()` function to the models, which produces some diagnostic information.

```
## Morcote
par(mfrow = c(2, 2))
gam.check(gam.tot.eggs_space.Morcote.23)
```

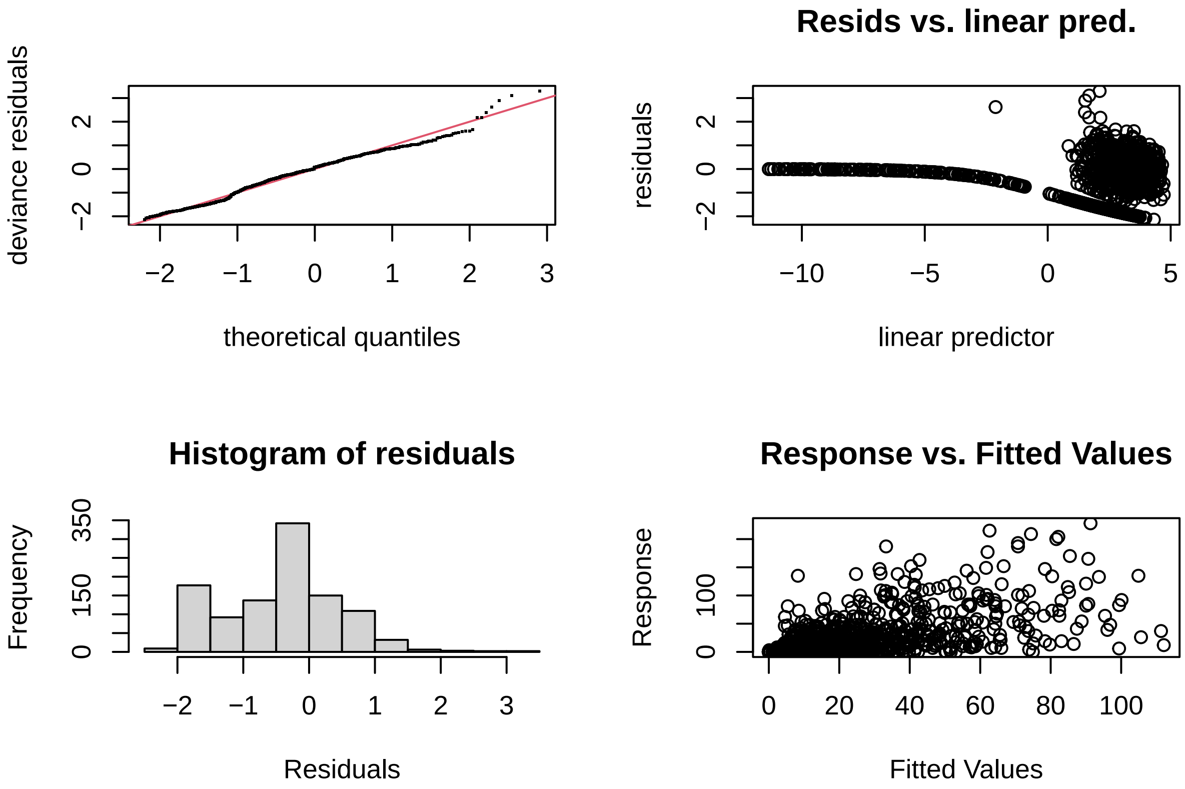


```
Method: REML   Optimizer: outer newton
full convergence after 5 iterations.
Gradient range [-1.997415e-05,0.0003596586]
(score 3431.951 & scale 1).
Hessian positive definite, eigenvalue range [2.118092,342.6768].
Model rank =  39 / 39

Basis dimension (k) checking results. Low p-value (k-index<1) may
indicate that k is too low, especially if edf is close to k'.

                  k'   edf k-index p-value
s(yday)         9.00  7.59    0.78   0.015 *
s(X.num,Y.num) 29.00 24.05    0.77  <2e-16 ***
---
Signif. codes:  0 '***' 0.001 '**' 0.01 '*' 0.05 '.' 0.1 ' ' 1
```

```r
par(mfrow = c(1, 1))
##
## Caslano
par(mfrow = c(2, 2))
gam.check(gam.tot.eggs_space.Caslano.23)
```

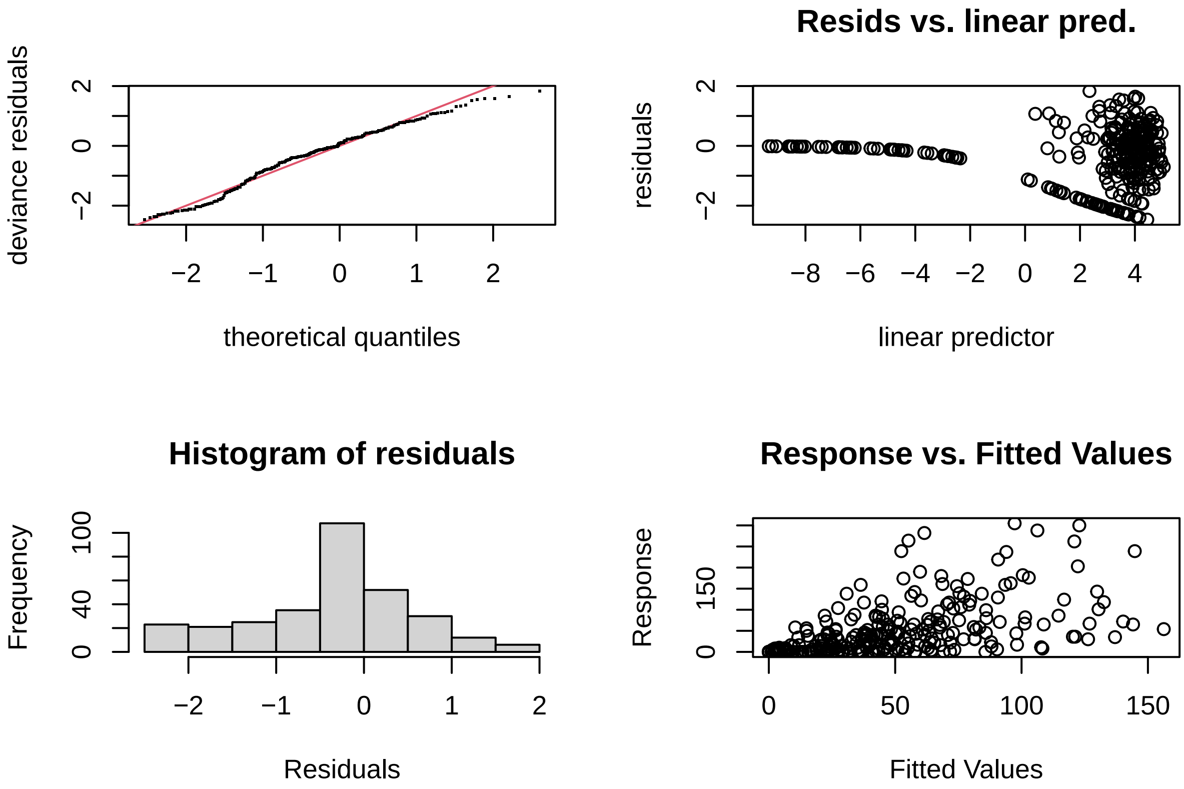


```
Method: REML   Optimizer: outer newton
full convergence after 6 iterations.
Gradient range [-2.750435e-06,3.600883e-06]
(score 1214.951 & scale 1).
Hessian positive definite, eigenvalue range [1.401462,114.4088].
Model rank =  23 / 23

Basis dimension (k) checking results. Low p-value (k-index<1) may
indicate that k is too low, especially if edf is close to k'.

                   k'   edf k-index p-value
s(yday)          9.00  6.49    0.85    0.30
s(X.num,Y.num) 13.00  9.01    0.87    0.43
```

```
par(mfrow = c(1, 1))
```

Then, we store the pearson residuals in the original data frame, and we plot the residuals against the fitted values to see whether there is still structure in the data.

```
## Morcote
d.ovitraps.23.morcote$resid_gam.tot.eggs_space.Morcote <- resid(gam.tot.eggs_space.Morcote.23,
                                                                type = "pearson")
##
d.ovitraps.23.morcote$fitted_gam.tot.eggs_space.Morcote <- fitted(gam.tot.eggs_space.Morcote.23)
##
ggplot(data = d.ovitraps.23.morcote,
       mapping = aes(y = resid_gam.tot.eggs_space.Morcote,
                     x = fitted_gam.tot.eggs_space.Morcote)) +
  geom_hline(yintercept = 0) +
```

```
  geom_point(alpha = 0.2) +
  geom_smooth()
```

```
`geom_smooth()` using method = 'gam' and formula = 'y ~ s(x, bs = "cs")'
```

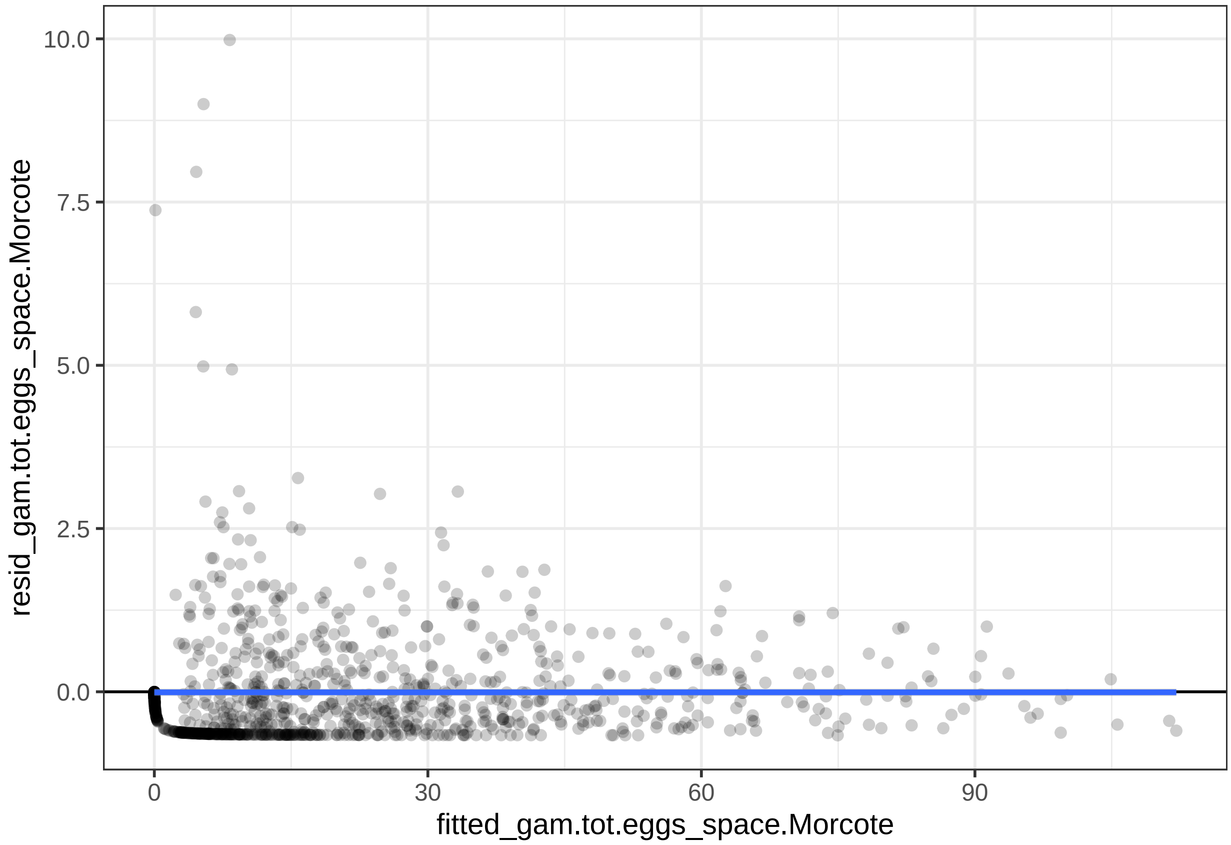


```
##
## Caslano
d.ovitraps.23.caslano$resid_gam.tot.eggs_space.Caslano <- resid(gam.tot.eggs_space.Caslano.23,
                                                                type = "pearson")
##
##
d.ovitraps.23.caslano$fitted_gam.tot.eggs_space.Caslano <- fitted(gam.tot.eggs_space.Caslano.23)
##
ggplot(data = d.ovitraps.23.caslano,
       mapping = aes(y = resid_gam.tot.eggs_space.Caslano,
                     x = fitted_gam.tot.eggs_space.Caslano)) +
  geom_hline(yintercept = 0) +
  geom_point(alpha = 0.2) +
  geom_smooth()
```

```
`geom_smooth()` using method = 'loess' and formula = 'y ~ x'
```

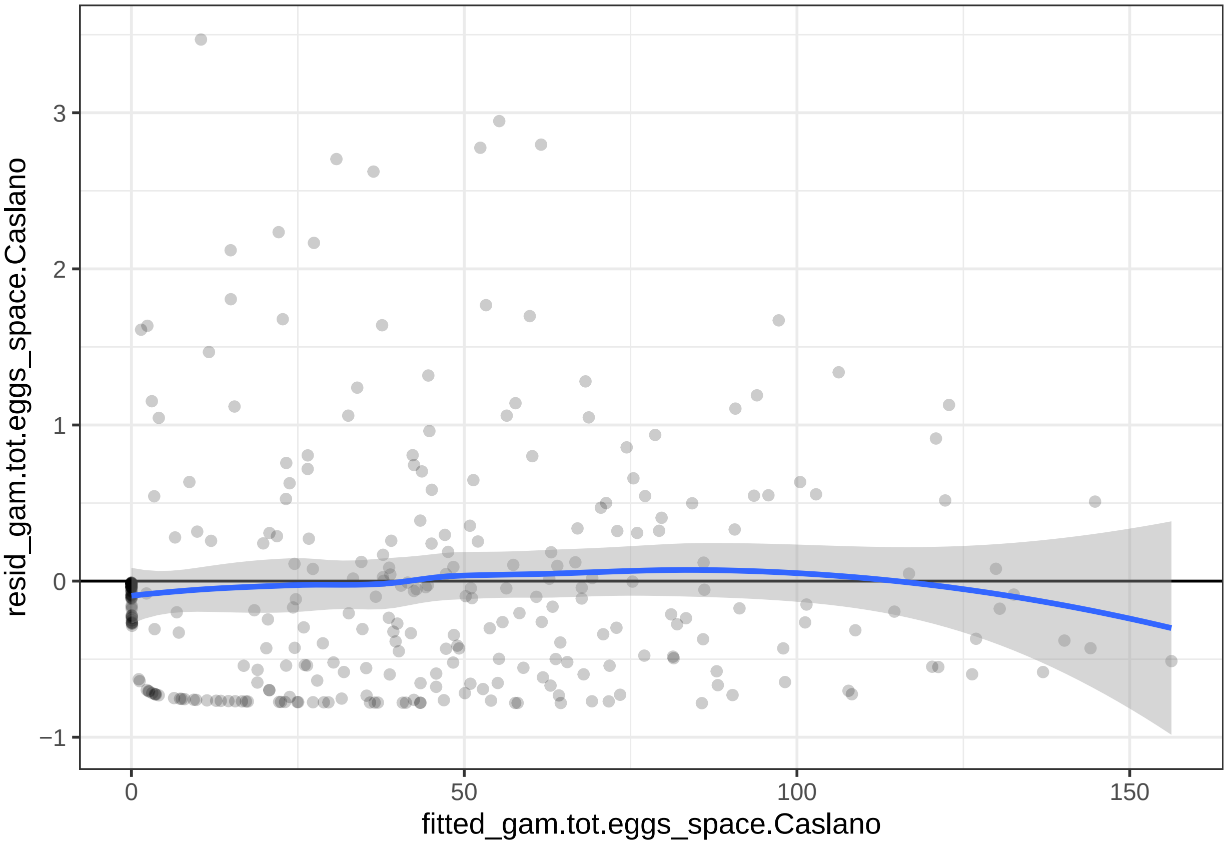

The blue lines are located on the x-axis (or the confidence band contains it), indicating that there doesn't seem to be structure left in the residuals.

6.4 Comparing (over)dispersion in the two models

We compare again the overdispersion in the two models.

As a reminder, in a negative binomial model the parameter θ is estimated to be such that:

$$var(y) = \mu + \mu^2/\theta, \quad \text{where} \quad \mu = \mathbb{E}(y).$$

Therefore, the lower the parameter value, the higher the overdispersion.

```
gam.tot.eggs_space.Morcote.23$family$getTheta(TRUE)
```

```
[1] 0.4466819
```

```
gam.tot.eggs_space.Caslano.23$family$getTheta(TRUE)
```

```
[1] 0.6154415
```

Overdispersion is higher in Morcote.

7 Methods

7.1 Generalised Additive Mixed-Effects Model (GAMM)

We modelled the response variable, "total number of *Ae. albopictus* eggs" (*total.albopictus.egg*, a count variable ranging from `0 to 535`), using a Generalized Additive Model (GAM) with a negative binomial

family to address overdispersion. The model included a smooth effect for “sampling date”, represented as the day of the year (*yday*, a numeric variable ranging from 114 to 282), which interacts with municipality (*municipality.fac*). *municipality.fac* is a categorical variable, consisting of 2 levels: Caslano, Morcote ; its effect was modelled as a fixed effect.

To account for variations in trap exposure duration, we incorporated *Activation.time* (a count variable ranging from 6 to 16 days) as an offset (we used the log of *Activation.time* to make the assumption that doubling the activation time would lead to proportional doubling in the number of eggs) in the model. We further controlled for the non-independence of observations by including *trap ID* (*unique.ID*, a categorical variable) as a random effect, with data from 77 distinct traps.
Model complexity was evaluated, and the best-fitting model was selected using a Chi-square test and AIC and BIC criteria. All statistical analyses were conducted using R.
The significance level was set at 5%.

7.2 Spatial Generalised Additive Model (spatial GAM)

We fitted two separate models to analyse the response variable, *total.albopictus.eggs*, using a Generalized Additive Model (GAM) with a negative binomial family to account for overdispersion. One model was fitted for the municipality of Morcote, and the other for Caslano.

Both models included a smooth effect for the “sampling date”, represented as day of the year (*yday*), and a combined smooth effect for geographic coordinates to capture spatial variability within each municipality. All statistical analyses were conducted using R. The significance level was set at 5%.

8 Results

8.1 Generalised Additive Mixed-Effects Model (GAMM)

- The model with Morcote and Caslano converged without problems, and explains

53.3 %

of the deviance.

- Allowing for different shapes for the municipalities was verified to be necessary for the model with only Morcote and Caslano (p-value

0).

AIC and BIC agree with anova.

In our analysis, we observed a higher variability in the number of Aedes albopictus in Morcote (overdispersion parameter: 0.4672072) compared to Calsano (overdispersion parameter: 0.6855243).

8.2 Spatial Generalised Additive Model (spatial GAM)

- Model for Morcote converged without problems. deviance explained is

49.6 %

- right-hand side of morcote, has almost 4 times higher counts for mosquitoes eggs than the left-hand side of Morcote.
- Model for Caslano converged without problems. Deviance explained is

55.9 %

Morcote has higher overdispersion (overdispersion parameter: `0.4466819`) compared to Calsano (overdispersion parameter: `0.6154415`).

Caslano doesn't have much variability in the number of eggs over the territory.

9 Conclusions

In the spatial analysis, it emerged that there was substantial variability in mosquito egg counts within Morcote. In fact, an important difference was present between the left and right sides, with the right side showing a higher median egg count and greater variability. In contrast, the left side had lower counts with less fluctuation.

One potential factor influencing this disparity could be Vico Morcote, a neighboring municipality of Morcote that was untreated and may be contributing to the higher egg counts in Morcote. However, this remains a hypothesis, and further analysis is needed to confirm it.

Caslano, by comparison, exhibited minimal geographical variation.

Our analysis highlights notable differences in mosquito egg counts between Morcote and Caslano, with Morcote exhibiting greater variability. Despite this variability, the overall number of Aedes albopictus eggs in Morcote was lower than in Caslano. A key factor influencing Morcote's variability appears to be the untreated neighbouring municipality of Vico Morcote, which may be contributing to the higher egg counts and fluctuations on the right-hand side of Morcote. The left-hand side, being more isolated, displayed lower egg counts and less variability.

10 References

```r
citation("mgcViz")
```

```
To cite the mgcViz package in publications use:

  Fasiolo, M., Nedellec, R., Goude, Y. and Wood, S.N., 2020. Scalable
  visualization methods for modern generalized additive models. Journal
  of computational and Graphical Statistics, 29(1), pp.78-86.

A BibTeX entry for LaTeX users is

  @Article{,
    title = {Scalable visualisation methods for modern Generalized Additive Models.},
    journal = {Journal of the Royal Statistical Society (B)},
    volume = {29},
    number = {1},
    pages = {78-86},
    year = {2020},
    author = {{Fasiolo} and {Matteo} and {Nedellec} and {Rapha{"e}l} and {Goude} and {Yannig} and {Wood]
  }

As mgcViz is often updated, you may want to cite its version number.
Find it with 'help(package=mgcViz)'.
```

```r
citation("lubridate")
```

```
To cite lubridate in publications use:
```

```
  Garrett Grolemund, Hadley Wickham (2011). Dates and Times Made Easy
  with lubridate. Journal of Statistical Software, 40(3), 1-25. URL
  https://www.jstatsoft.org/v40/i03/.

A BibTeX entry for LaTeX users is

  @Article{,
    title = {Dates and Times Made Easy with {lubridate}},
    author = {Garrett Grolemund and Hadley Wickham},
    journal = {Journal of Statistical Software},
    year = {2011},
    volume = {40},
    number = {3},
    pages = {1--25},
    url = {https://www.jstatsoft.org/v40/i03/},
  }
```

```r
citation()
```

```
To cite R in publications use:

  R Core Team (2024). _R: A Language and Environment for Statistical
  Computing_. R Foundation for Statistical Computing, Vienna, Austria.
  <https://www.R-project.org/>.

A BibTeX entry for LaTeX users is

  @Manual{,
    title = {R: A Language and Environment for Statistical Computing},
    author = {{R Core Team}},
    organization = {R Foundation for Statistical Computing},
    address = {Vienna, Austria},
    year = {2024},
    url = {https://www.R-project.org/},
  }

We have invested a lot of time and effort in creating R, please cite it
when using it for data analysis. See also 'citation("pkgname")' for
citing R packages.
```

11 Session Information

```r
sessionInfo()
```

```
R version 4.4.2 (2024-10-31)
Platform: aarch64-apple-darwin20
Running under: macOS Sequoia 15.0.1

Matrix products: default
BLAS:   /Library/Frameworks/R.framework/Versions/4.4-arm64/Resources/lib/libRblas.0.dylib
LAPACK: /Library/Frameworks/R.framework/Versions/4.4-arm64/Resources/lib/libRlapack.dylib;  LAPACK vers

locale:
```

```
[1] en_US.UTF-8/en_US.UTF-8/en_US.UTF-8/C/en_US.UTF-8/en_US.UTF-8

time zone: Europe/Zurich
tzcode source: internal

attached base packages:
[1] stats     graphics  grDevices utils     datasets  methods   base

other attached packages:
 [1] readxl_1.4.3    mgcViz_0.1.11   qgam_1.3.4      mgcv_1.9-1
 [5] nlme_3.1-166    lubridate_1.9.3 ggplot2_3.5.1   dplyr_1.1.4
 [9] groundhog_3.2.1 knitr_1.49

loaded via a namespace (and not attached):
 [1] gtable_0.3.6       xfun_0.49          GGally_2.2.1       lattice_0.22-6
 [5] vctrs_0.6.5        tools_4.4.2        generics_0.1.3     parallel_4.4.2
 [9] gamm4_0.2-6        tibble_3.2.1       fansi_1.0.6        pkgconfig_2.0.3
[13] Matrix_1.7-1       KernSmooth_2.23-24 RColorBrewer_1.1-3 lifecycle_1.0.4
[17] farver_2.1.2       compiler_4.4.2     tinytex_0.54       munsell_0.5.1
[21] codetools_0.2-20   httpuv_1.6.15      htmltools_0.5.8.1  yaml_2.3.10
[25] later_1.3.2        pillar_1.9.0       nloptr_2.1.1       tidyr_1.3.1
[29] MASS_7.3-61        iterators_1.0.14   viridis_0.6.5      boot_1.3-31
[33] foreach_1.5.2      mime_0.12          ggstats_0.7.0      tidyselect_1.2.1
[37] digest_0.6.37      purrr_1.0.2        labeling_0.4.3     splines_4.4.2
[41] fastmap_1.2.0      grid_4.4.2         colorspace_2.1-1   cli_3.6.3
[45] magrittr_2.0.3     utf8_1.2.4         withr_3.0.2        scales_1.3.0
[49] promises_1.3.0     timechange_0.3.0   rmarkdown_2.29     matrixStats_1.4.1
[53] lme4_1.1-35.5      gridExtra_2.3      cellranger_1.1.0   shiny_1.9.1
[57] evaluate_1.0.1     doParallel_1.0.17  miniUI_0.1.1.1     viridisLite_0.4.2
[61] rlang_1.1.4        isoband_0.2.7      Rcpp_1.0.13        xtable_1.8-4
[65] glue_1.8.0         rstudioapi_0.17.1  minqa_1.2.8        R6_2.5.1
[69] plyr_1.8.9
```
