# Supplementary material for: Effectiveness of the sterile insect technique in controlling Aedes albopictus as part of an integrated control measure: evidence from a first small-scale field trial in Switzerland
Source: Infect Dis Poverty. 2025 Aug 22;14:90. doi: 10.1186/s40249-025-01360-2 (PMC12372186; doi:10.1186/s40249-025-01360-2)
Supplement: Supplementary file 3 — Supplementary Material 3. Analysis percentage of Ae. albopictus eggs that hatched. [file 40249_2025_1360_MOESM3_ESM.pdf]

Modelling: Paper Morcote Project

Percentage of hatched eggs

Author: Nisia Trisconi & Dr. Matteo Tanadini | Zurich Data Scientists
Reviewer: Dr. Luisa Barbanti | Zurich Data Scientists

February 3, 2025

Contents

1 Freeze Package versions **3**

2 Settings **3**

3 Getting data **4**

4 Aim and design **5**

5 Generalised Additive Mixed-Effects Model (GAMM) **5**
5.1 Visualising the data . . . 5
5.2 Fitting the model . . . 12
5.3 Plotting the model . . . 15
5.4 Fitted values . . . 19
5.5 Predicted values . . . 21
5.6 Model selection – Shape . . . 24
5.7 Residual analysis . . . 27
5.8 Comparing (over)dispersion in the two models . . . 35

6 Spatial Generalised Additive Model (spatial GAM) **38**
6.1 Visualising the data . . . 38
6.2 Fitting the models . . . 41
6.3 Plotting the model . . . 43
6.3.1 Morcote and Vico Morcote . . . 43
6.3.2 Caslano . . . 44
6.4 Residual analysis . . . 46
6.5 Comparing (over)dispersion in the two models . . . 50

7 Methods **50**
7.1 Generalised Additive Mixed-Effects Model (GAMM) . . . 51
7.2 Spatial Generalised Additive Model (spatial GAM) . . . 51

8 Results **51**
8.1 Generalised Additive Mixed-Effects Model (GAMM) . . . 51
8.2 Spatial Generalised Additive Model (spatial GAM) . . . 51

9 Conclusions **52**

10 References **52**

11 Appendix **53**
11.1 Hatched eggs over discrete time . . . 53
11.2 Hatched Eggs over space (varying over time) . . . 56

12 Appendix – Models including Vico Morcote **58**
12.1 GAMM . . . 58
12.1.1 Visualising the data . . . 58
12.1.2 Fitting the model . . . 64
12.1.3 Plotting the model . . . 66
12.1.4 Fitted values . . . 70
12.1.5 Predicted values . . . 72
12.1.6 Model selection – Shape . . . 75
12.1.7 Residual analysis . . . 78
12.2 Spatial Generalised Additive Models . . . 86
12.2.1 Visualising the data . . . 86
12.2.2 Fitting the models . . . 88
12.2.3 Plotting the model . . . 90
12.2.4 Morcote and Vico Morcote . . . 90
12.2.5 Residual analysis . . . 91
12.2.6 Comparing (over)dispersion in the two models . . . 94

1 Freeze Package versions

```
## (messages are omitted from this chunk)
##
library(groundhog)
pkgs <- c("dplyr",
          "ggplot2",
          "lubridate",
          "glmmTMB",
          "mgcViz",
          "readxl")
groundhog.library(pkgs, date = "2024-11-01")
```

2 Settings

Global settings:

```
Sys.setenv(lang = "en_US")
theme_set(theme_bw())

if (!dir.exists("Prepared_data_and_models")) {
  dir.create("Prepared_data_and_models")
}
```

3 Getting data

```
d.ovitraps <- readRDS(file = paste0("Prepared_data_and_models/",
                                    "d.ovitraps_PreparedData.RDS"))
```

For this file, we only consider the year 2023. So we define a reduced data set:

```
d.ovitraps.23 <- d.ovitraps %>%
  filter(Year == 2023)

## check
d.ovitraps.23 %>%
  select(Year) %>%
  unique()
```

```
# A tibble: 1 x 1
   Year
  <dbl>
1  2023
```

Overview of the data:

```
dim(d.ovitraps.23)
```

```
[1] 2503   33
```

```
head(d.ovitraps.23)[1:min(ncol(d.ovitraps.23), 30)]
```

```
# A tibble: 6 x 30
  OriginalSheet Activation.time total.eggs total.albopictus.egg
  <chr>                   <dbl>      <dbl>                <dbl>
1 1                          14          0                    6
2 1                          14          0                    0
3 1                          14          6                    6
4 1                          14          0                    0
5 1                          14          0                    0
6 1                          14          0                    0
# i 26 more variables: WHOLE.albopictus.egg <dbl>,
#   DESSICATED.albopictus.egg <dbl>, HATCH.albopictus.egg <dbl>,
#   WHOLE.albopictus.egg..AFTER.HATCHING.PROCEDURE. <dbl>,
#   DESSICATED.albopictus.egg..AFTER.HATCHING.PROCEDURE. <dbl>,
#   HATCH.albopictus.egg..AFTER.HATCHING.PROCEDURE. <dbl>,
#   Perc.egg.hatch.per.trap <dbl>, Perc.egg.hatch.per.area <dbl>, Year <dbl>,
#   unique.ID <fct>, X.num <dbl>, Y.num <dbl>, round.fac <fct>, ...
```

```
str(d.ovitraps.23)
```

```
tibble [2,503 x 33] (S3: tbl_df/tbl/data.frame)
 $ OriginalSheet                                        : chr [1:2503] "1" "1" "1" "1" ...
 $ Activation.time                                      : num [1:2503] 14 14 14 14 14 14 14 14 14 14 ...
 $ total.eggs                                           : num [1:2503] 0 0 6 0 0 0 0 0 0 0 ...
 $ total.albopictus.egg                                 : num [1:2503] 6 0 6 0 0 0 0 0 0 0 ...
 $ WHOLE.albopictus.egg                                 : num [1:2503] NA NA NA NA NA NA NA NA NA NA ...
 $ DESSICATED.albopictus.egg                            : num [1:2503] NA NA NA NA NA NA NA NA NA NA ...
 $ HATCH.albopictus.egg                                 : num [1:2503] NA NA NA NA NA NA NA NA NA NA ...
 $ WHOLE.albopictus.egg..AFTER.HATCHING.PROCEDURE.      : num [1:2503] NA NA NA NA NA NA NA NA NA NA ...
 $ DESSICATED.albopictus.egg..AFTER.HATCHING.PROCEDURE.: num [1:2503] NA NA NA NA NA NA NA NA NA NA ...
```

```
 $ HATCH.albopictus.egg..AFTER.HATCHING.PROCEDURE.     : num [1:2503] NA NA NA NA NA NA NA NA NA NA ...
 $ Perc.egg.hatch.per.trap                             : num [1:2503] NA NA NA NA NA NA NA NA NA NA ...
 $ Perc.egg.hatch.per.area                             : num [1:2503] NA NA NA NA NA NA NA NA NA NA ...
 $ Year                                                : num [1:2503] 2023 2023 2023 2023 2023 ...
 $ unique.ID                                           : Factor w/ 348 levels "Caslano.1","Vico Morcote
 $ X.num                                               : num [1:2503] NA NA NA NA NA NA NA NA NA NA ...
 $ Y.num                                               : num [1:2503] NA NA NA NA NA NA NA NA NA NA ...
 $ round.fac                                           : Factor w/ 44 levels "1","2","3","4",..: 6 6 6 
 $ Setting_date.date                                   : Date[1:2503], format: "2023-05-11" "2023-05-11
 $ Sampling_date.date                                  : Date[1:2503], format: "2023-05-25" "2023-05-25
 $ municipality.ord                                    : Ord.factor w/ 17 levels "Ascona"<"Balerna"<..: 
 $ municipality.fac                                    : Factor w/ 17 levels "Ascona","Balerna",..: 2 2
 $ status.fac                                          : Factor w/ 5 levels "A","F","M","not dispayed",
 $ perc.manual                                         : num [1:2503] NA NA NA NA NA NA NA NA NA NA ...
 $ RowNumber                                           : int [1:2503] 1 2 3 4 5 6 7 8 9 10 ...
 $ Year.fac                                            : Factor w/ 2 levels "2023","2024": 1 1 1 1 1 1 
 $ hatched.albo.eggs.after.proc                        : num [1:2503] NA NA NA NA NA NA NA NA NA NA ...
 $ daily.albopictus.eggs                               : num [1:2503] 0.429 0 0.429 0 0 ...
 $ yday                                                : num [1:2503] 145 145 145 145 145 145 145 145 14
 $ non.hatched.albo.eggs.after.proc                    : num [1:2503] NA NA NA NA NA NA NA NA NA NA ...
 $ week                                                : num [1:2503] 21 21 21 21 21 21 21 21 21 21 ...
 $ unique.ID.Year                                      : Factor w/ 518 levels "Caslano.1.2023",..: 58 9
 $ super.municipality.year                             : Factor w/ 34 levels "Ascona:2023",..: 2 2 2 2 
 $ municipality.fac.spatial                            : Factor w/ 14 levels "Ascona","Balerna",..: 2 2
```

4 Aim and design

In 2023, SUPSI received approval from the FOEN to release sterile male tiger mosquitoes in the municipality of Morcote as part of a field experiment aimed at reducing the number of eggs laid by female mosquitoes.

The experiment involved weekly releases of sterile male *Aedes albopictus* mosquitoes in Morcote during the active mosquito season, from May to September 2023. Approximately 3,000 sterile males per hectare were released each week through 75 predefined stations spaced 50 to 80 meters apart.

Ovitraps were sampled multiple times over time in Morcote (treated) and Caslano (untreated) to assess whether the release of sterile males had an impact on the percentage of *Aedes albopictus* hatched eggs. Additionally, ovitraps from Vico Morcote, a neighbouring municipality of Morcote, were also sampled to check border effect of the treatment.

In total, the data was collected on `‘tot.sampling‘` different dates. Each municipality was sampled from a minimum of `6` times to a maximum of `12` times.

5 Generalised Additive Mixed-Effects Model (GAMM)

5.1 Visualising the data

The aim of this analysis is to determine whether the release of sterile males in a given area helps reducing the percentage of hatched eggs.

The hatching procedure was only performed for Caslano, Morcote and Vico Morcote. Moreover, this procedure was not performed every week, thus we remove all the observations having NAs for *hatched.albo.eggs.after.proc*.

```
d.ovitraps.23.hatch.proc <- d.ovitraps.23 %>%
  filter(!is.na(Perc.egg.hatch.per.trap)) %>%
  droplevels()
```

```
##
## check
levels(d.ovitraps.23.hatch.proc$municipality.fac)
```

```
[1] "Caslano"      "Morcote"      "Vico Morcote"
```

```
## makes sense
dim(d.ovitraps.23.hatch.proc)
```

```
[1] 475  33
```

```
dim(d.ovitraps.23)
```

```
[1] 2503   33
```

We also create a data set without Vico Morcote, keeping only Caslano and Morcote:

```
d.ovitraps.23.hatch.proc.MC <- d.ovitraps.23.hatch.proc %>%
  filter(municipality.fac %in% c("Caslano", "Morcote"))

## check
d.ovitraps.23.hatch.proc.MC %>%
  select(municipality.fac) %>%
  table()
```

```
municipality.fac
     Caslano      Morcote Vico Morcote
         108          352            0
```

```
## we drop Vico Morcote from the levels for plotting purposes
d.ovitraps.23.hatch.proc.MC <- d.ovitraps.23.hatch.proc.MC %>%
  mutate(municipality.fac = droplevels(municipality.fac))

## check
d.ovitraps.23.hatch.proc.MC %>%
  select(municipality.fac) %>%
  table()
```

```
municipality.fac
Caslano Morcote
    108     352
```

We start by displaying the percentage of eggs hatched in the traps sampled during the season. Observations from the same ovitrap are connected by lines to visualise trends over time. Above these lines, a smoothed average is plotted (with family "symmetric" to be more robust), allowing the overall trend of the percentage of eggs hatched to be seen.

```
## (messages and warnings are excluded from this chunk)
##
p <- ggplot(data = d.ovitraps.23.hatch.proc.MC,
            mapping = aes(y = `Perc.egg.hatch.per.trap`,
                          x = `Sampling_date.date`,
                          group = unique.ID)) +
  geom_hline(yintercept = c(0, 100), colour = "gray") +
  geom_point(alpha = 0.2) +
  geom_line(alpha = 0.2) +
  geom_smooth(mapping = aes(group = NULL),
              method = "loess",
```

```
            method.args = list(family = "symmetric"))
p
```

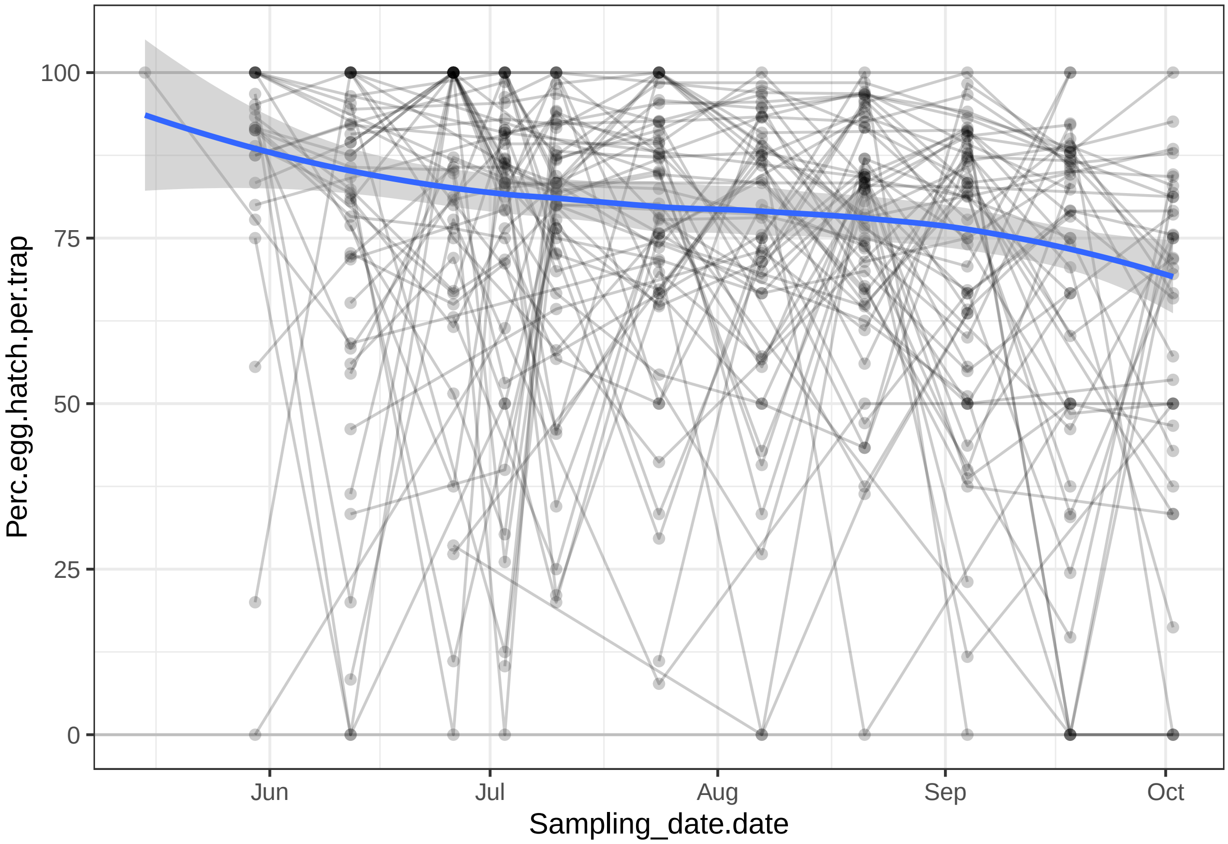


```
## save plot for future use:
saveRDS(p, file = file.path("saved_figures", "2b_eggsOverTime.rds"))
```

The sterile males were released only in one municipality (Morcote), while the others served as controls. For this reason, we now create a separate plot for each municipality to observe whether there are any differences between them.

```
## (messages and warnings are excluded from this chunk)
##
p <- ggplot(data = d.ovitraps.23.hatch.proc.MC,
            mapping = aes(y = `Perc.egg.hatch.per.trap`,
                          x = `Sampling_date.date`,
                          group = unique.ID)) +
  geom_hline(yintercept = c(0, 100), colour = "gray") +
  geom_point(alpha = 0.2) +
  coord_cartesian(ylim = c(0, 100)) +
  geom_line(alpha = 0.2) +
  facet_wrap(~municipality.fac) +
  geom_smooth(mapping = aes(group = 1), method = "loess",
            method.args = list(family = "symmetric"))
p
```

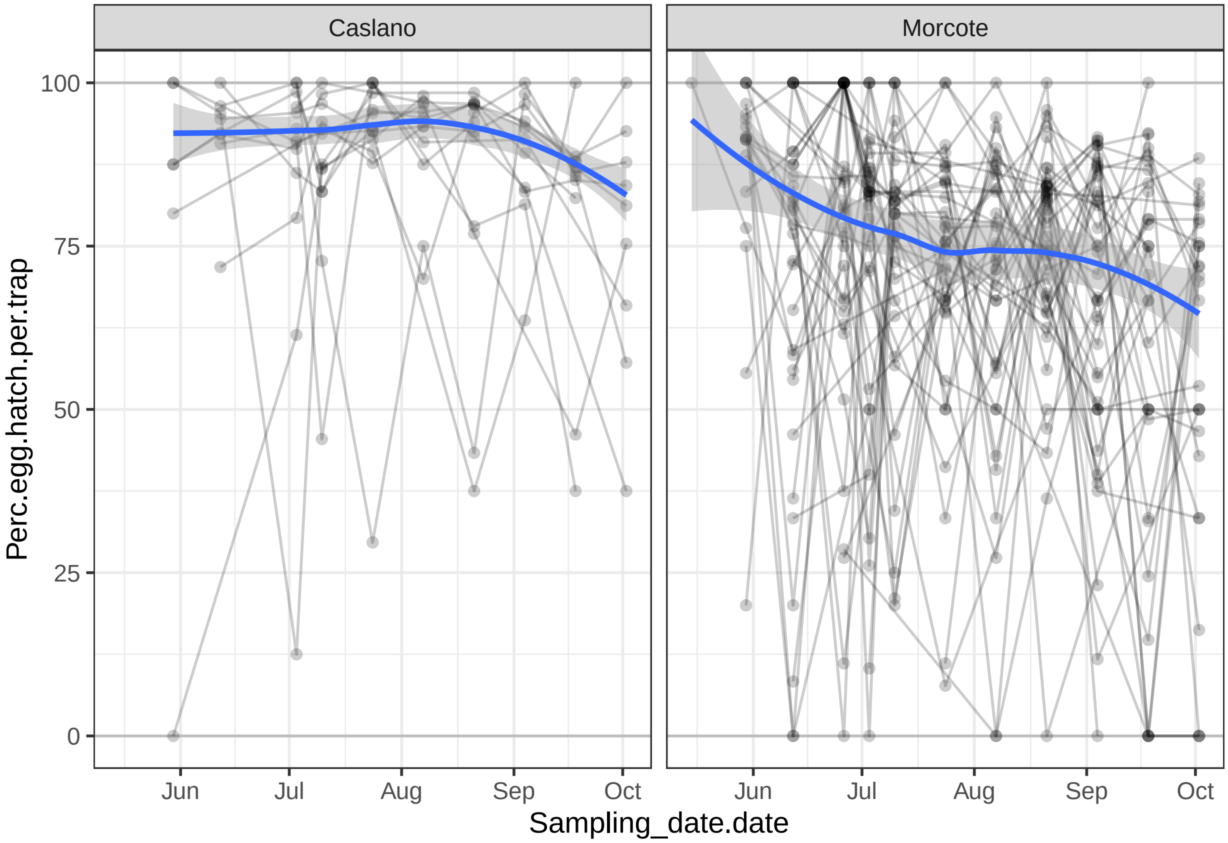


```
## save plot for future use:
saveRDS(p, file = file.path("saved_figures", "2b_TotalEggsMuniPanel.rds"))
```

To facilitate comparison, rather than creating separate plots, we will overlay the municipalities on a single plot (using colours to distinguish them) and add a smoother for each one.

```
## (messages and warnings are excluded from this chunk)
##
p <- ggplot(data = d.ovitraps.23.hatch.proc.MC,
            mapping = aes(y = `Perc.egg.hatch.per.trap`,
                          x = `Sampling_date.date`,
                          group = unique.ID,
                          colour = municipality.fac)) +
  geom_hline(yintercept = c(0, 100), colour = "gray") +
  geom_point(alpha = 0.1) +
  geom_line(alpha = 0.1) +
  coord_cartesian(ylim = c(0,100)) +
  geom_smooth(mapping = aes(group = municipality.fac),
              method = "loess",
              method.args = list(family = "symmetric"))
p
```

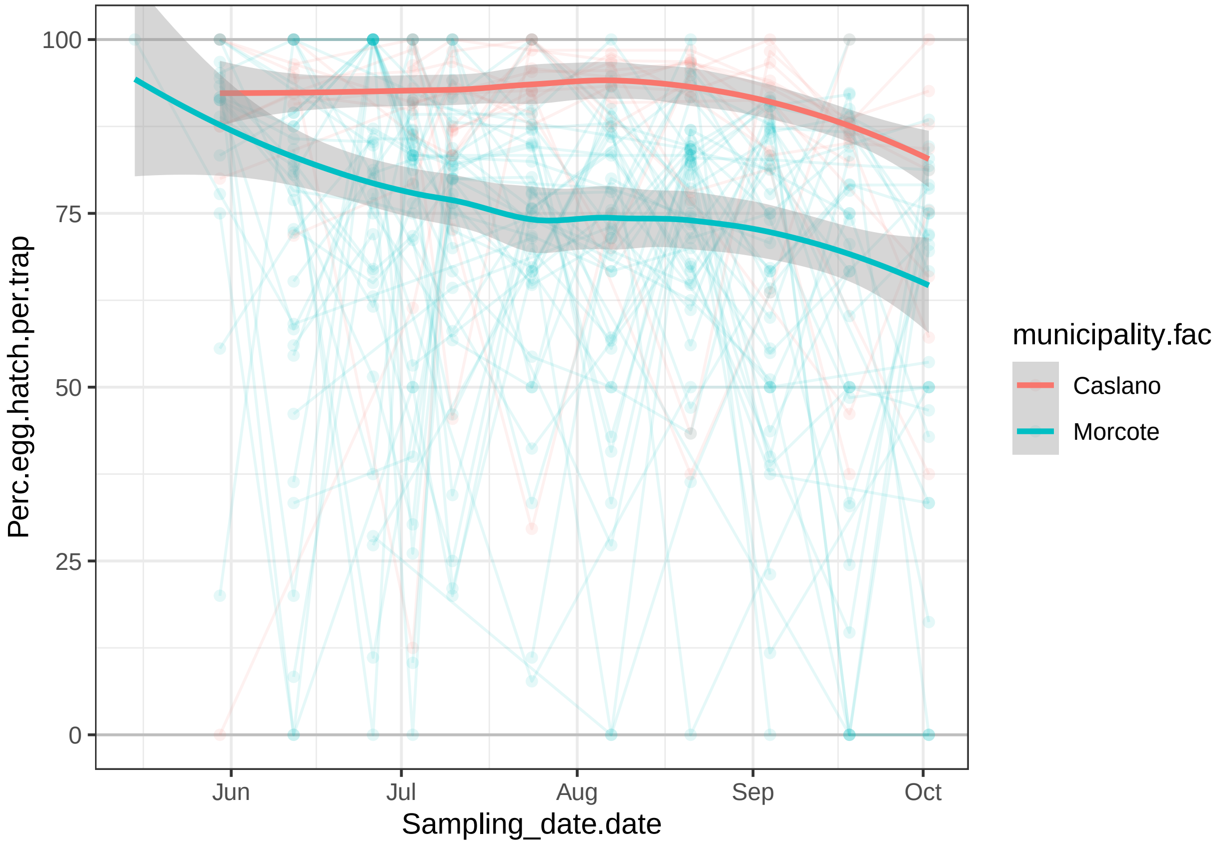


```r
## save plot for future use:
saveRDS(p, file = file.path("saved_figures", "2b_TotalEggsMuniSmooth.rds"))
```

There seems to be a systematic difference between Caslano a Morcote. In Vico Morcote there are few observations to draw any strong conclusions.

Let's look at the same graph, but with mean lines instead of smoothers.

```r
p <- ggplot(data = d.ovitraps.23.hatch.proc.MC,
            mapping = aes(y = Perc.egg.hatch.per.trap,
                          x = Sampling_date.date,
                          group = unique.ID,
                          colour = municipality.fac)) +
  geom_point(alpha = 0.1) +
  geom_line(alpha = 0.1) +
  coord_cartesian(ylim = c(0, 100)) +
  geom_hline(yintercept = c(0, 100), colour = "gray") +
  stat_summary(fun  = mean,
               fun.args = list(na.rm = TRUE),
               geom = "line",
               mapping = aes( group = municipality.fac))
p
```

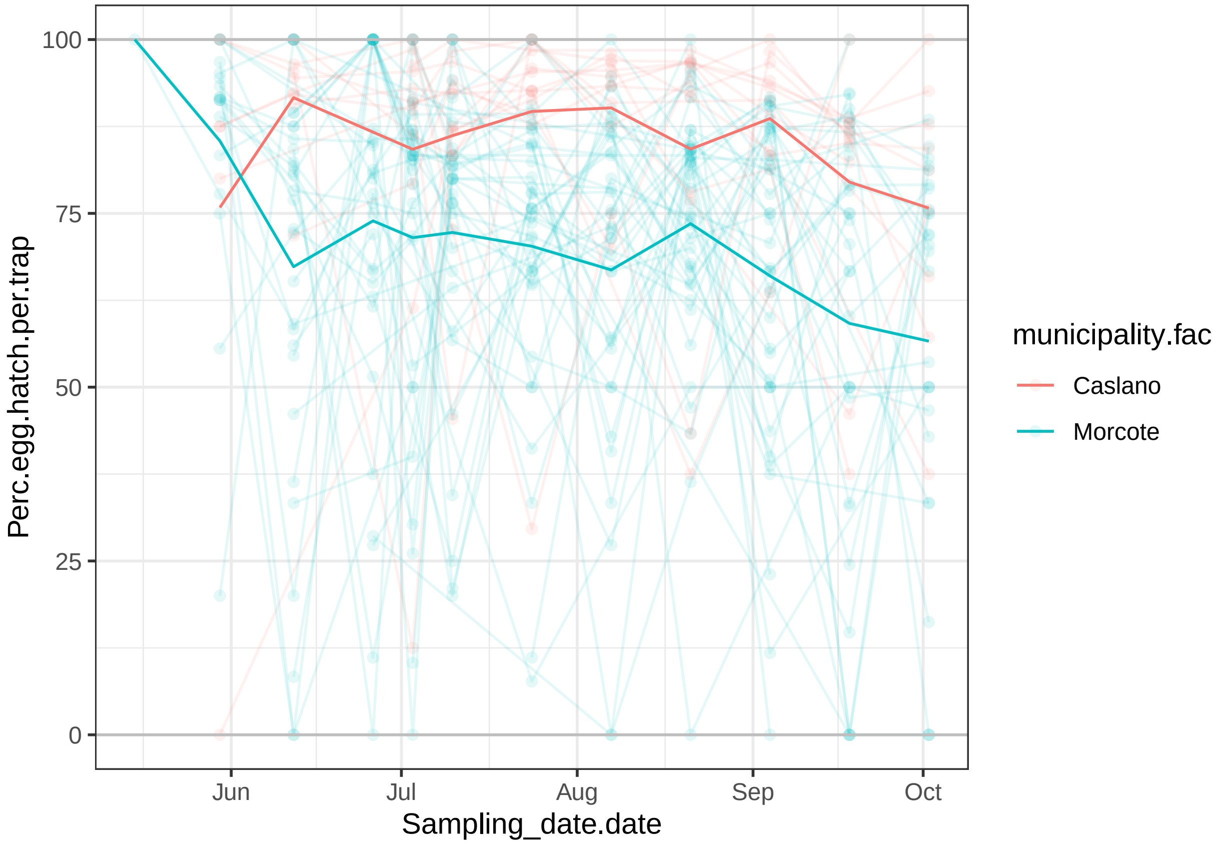


```r
## save plot for future use:
saveRDS(p, file = file.path("saved_figures", "2b_HatchedEggsOVertimeMeans.rds"))
```

The conclusion remains the same.

Finally, we focus on Morcote to examine seasonal differences among the ovitraps. Since we are not concerned with the behaviour of individual ovitraps, we remove the labels.

```r
## (messages and warnings are excluded from this chunk)
##
p <- ggplot(data = filter(d.ovitraps.23.hatch.proc.MC,
                          municipality.fac == "Morcote"),
            mapping = aes(y = `Perc.egg.hatch.per.trap`,
                          x = `Sampling_date.date`,
                          group = unique.ID)) +
  geom_hline(yintercept = c(0, 100), colour = "gray") +
  geom_point() +
  geom_line() +
  scale_y_sqrt(limits = c(0,NA), ) +
  facet_wrap(~unique.ID) +
  theme(
    strip.background = element_blank(),
    strip.text.x = element_blank(),
    axis.text.x = element_text(angle = 90))
p
```

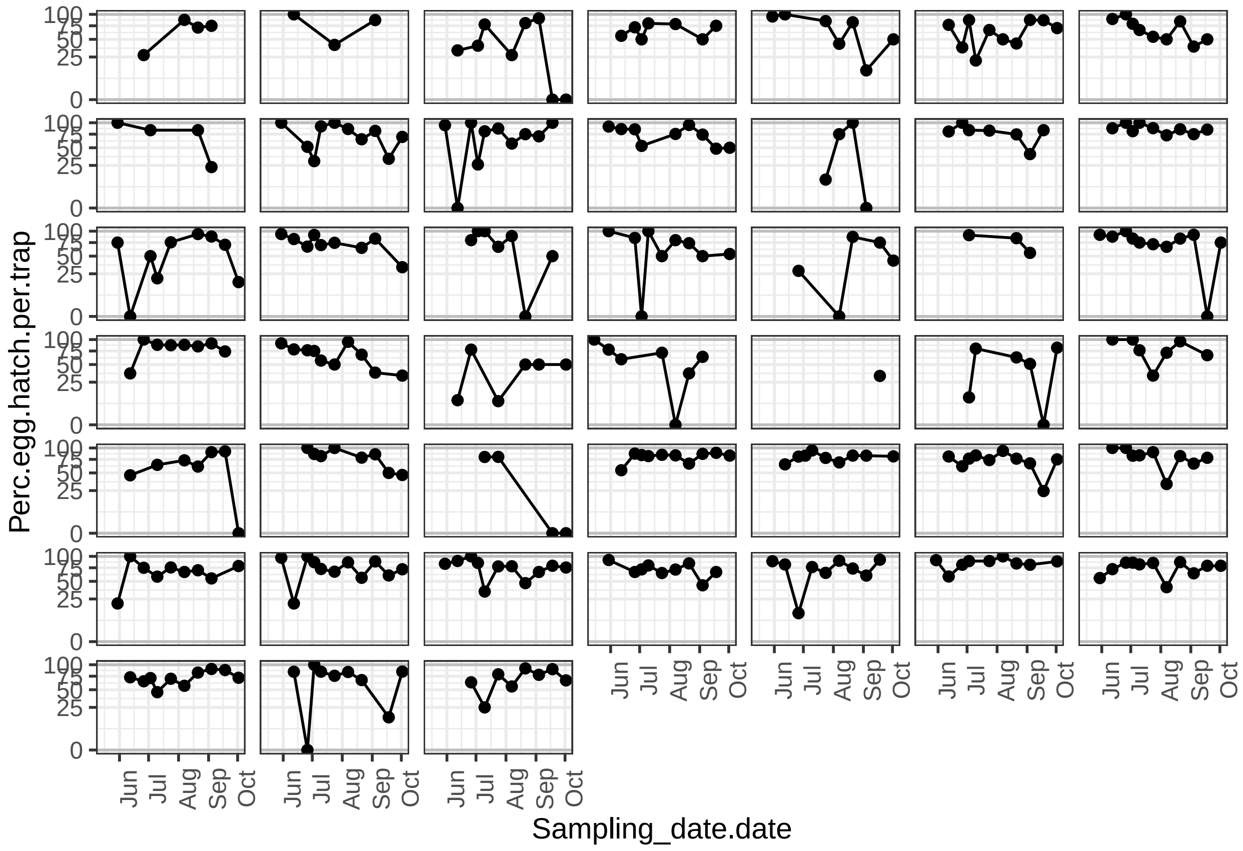


```
## save plot for future use:
saveRDS(p, file = file.path("saved_figures", "2b_MorcoteEggsOverTimeNolabels.rds"))
```

There is significant variation between ovitraps. For instance, in the second-last bottom panel, we can observe an abrupt drop from almost 100% hatched eggs one week to zero the next.

@Diego: There is one ovitrap with a single observation. Why is this the case?

Internal comment: This is something very relevant to discuss for the design of the SIT.

We create the same plot for Caslano to determine whether the observed behaviour is a result of the release of sterile males, or if it is a common pattern in the control municipalities as well.

```
## (messages and warnings are excluded from this chunk)
##
p <- ggplot(data = filter(d.ovitraps.23.hatch.proc.MC,
                          municipality.fac == "Caslano"),
            mapping = aes(y = `Perc.egg.hatch.per.trap`,
                          x = `Sampling_date.date`,
                          group = unique.ID)) +
  geom_hline(yintercept = c(0, 100), colour = "gray") +
  geom_point() +
  geom_line() +
  scale_y_sqrt(limits = c(0, NA)) +
  facet_wrap(~unique.ID) +
  theme(
    strip.background = element_blank(),
    strip.text.x = element_blank(),
```

```
    axis.text.x = element_text(angle = 90))
p
```

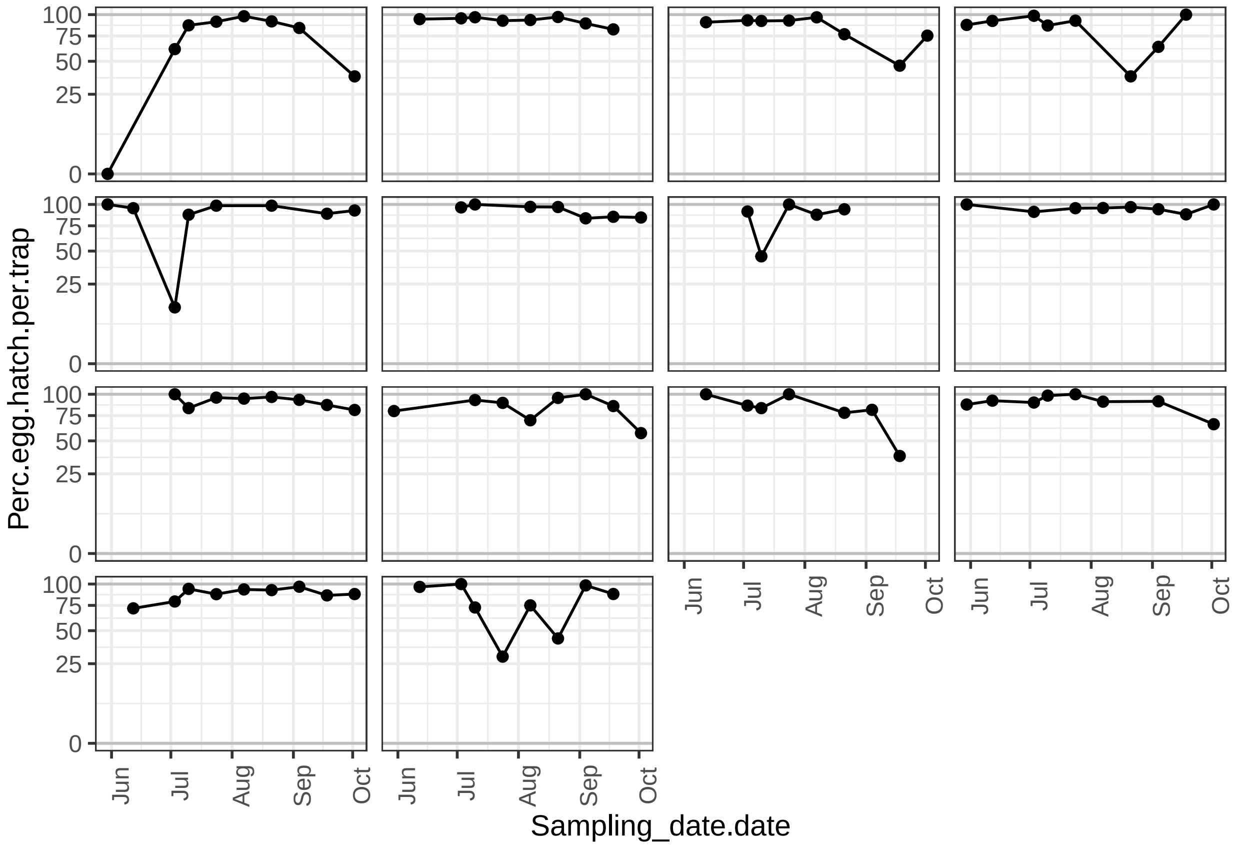


```
## save plot for future use:
saveRDS(p, file = file.path("saved_figures", "2b_CaslanoEggsOverTimeNolabels.rds"))
```

There is still some variability, but it is less pronounced compared to Morcote.

@Diego: Can this be due to the SIT males released?

5.2 Fitting the model

We aim to model the percentage of hatched *Aedes albopictus* eggs over the season (*yday*), while accounting for differences between municipalities (*municipality.fac*). Since sterile mosquitoes were released only in Morcote, municipalities will not be included as a random effect but rather as fixed effects to assess the actual differences.

The response variable is binomial, representing the counts of hatched versus non-hatched eggs (quantified by the variables *hatched.albo.eggs.after.proc* and *non.hatched.albo.eggs.after.proc*). To account for overdispersion, the quasi-binomial family is used for formal modelling.

Additionally, we will include an interaction between *municipality.fac* and *yday* to allow for distinct seasonal patterns across municipalities, avoiding the assumption that they behave similarly. These smooths will be centered, so the variable *municipality.fac* needs to be added as a main effect as well (because *municipality.fac* is a factor). In a subsequent section we will then test whether this distinct seasonal patterns across municipalities are needed.

yday will be included as numeric variable.

We begin without assuming any specific seasonal pattern, so we will fit a Generalised Additive Model (GAM), which provides flexibility in determining the best shape for the seasonal trend. However, this flexibility comes at the cost of interpretability.

Since observations within the same ovitrap are not independent, we will account for this dependency by setting *unique.ID* (the ovitrap variable) as a random effect. This adjustment captures the variability specific to each ovitrap.

Finally, some municipalities have fewer sampling dates, which impacts the number of knots used in the smooth term for the seasonal trend.

Let's check how many sampling dates there are for each municipality.

```
d.ovitraps.23 %>%
  group_by(municipality.fac) %>%
  summarise(nr.knots = n_distinct(yday)) %>%
  arrange(nr.knots)
```

```
# A tibble: 11 x 2
   municipality.fac  nr.knots
   <fct>                <int>
 1 Melide                   5
 2 Gravesano                6
 3 Manno                    6
 4 TaverneTorricella        6
 5 Vico Morcote             9
 6 Balerna                 10
 7 Canobbio                10
 8 Chiasso                 10
 9 Gordola                 10
10 Morcote                 25
11 Caslano                 34
```

Some municipalities have only five distinct values per *yday*. By default, when using the "by" argument into the `gam()` function, it adjust the maximum complexity based on the data available for each level of the variable specified in "by". For example, for municipalities like Morcote, which have more data points, the model will start from a larger basis dimension. However, this variation should not affect the overall conclusions.

Additionally, we will use a "point constraint" for *yday* (specifically at day 182), meaning all seasonal effects for *yday* are relative to the 1st of July.

```
pc.23 <- as.Date("2023-07-01") %>% yday()
pc.23
```

```
[1] 182
```

We will first remove the observations having missing values for the relevant variables.

```
d.ovitraps.23.mod <- d.ovitraps.23.hatch.proc.MC %>%
  select(hatched.albo.eggs.after.proc,
         municipality.fac,
         non.hatched.albo.eggs.after.proc,
         Activation.time, unique.ID,
         Sampling_date.date,
         yday,
         municipality.ord,
         X.num, Y.num) %>%
  na.omit() %>%
```

```r
  droplevels()
##
## check
dim(d.ovitraps.23)
```

```
[1] 2503   33
```

```r
dim(d.ovitraps.23.mod)
```

```
[1] 460  10
```

We rename *hatched.albo.eggs.after.proc* and *non.hatched.albo.eggs.after.proc* to have simpler and smaller variable names.

```r
d.ovitraps.23.mod <- d.ovitraps.23.mod %>%
  rename(hatched = hatched.albo.eggs.after.proc,
         non.hatched = non.hatched.albo.eggs.after.proc)
```

Now, we can fit the model. We use the `gamV()` function instead of `gam()`, because it fits the `gam()` model and automatically converts it to a `gamViz` object, making visualisation easier.
The model's estimates are exactly the same.

Note that the fitting procedure takes a few minutes. Therefore, the model is fitted and stored as RDS file and does not need to be refitted at each compilation.

```r
## (This chunk is not evaluated)
##
gamm.hatched.eggs.23 <- gamV(
  cbind(hatched, non.hatched) ~
    s(yday, by = municipality.fac, pc = pc.23) +
    municipality.fac +
    s(unique.ID, bs = "re"),
  family = "quasibinomial",
  data = d.ovitraps.23.mod)
##
saveRDS(gamm.hatched.eggs.23,
        file = "Prepared_data_and_models/GAMM_hatched_eggs.23.RDS")
```

We load the previously fitted model.

```r
gamm.hatched.eggs.23 <- readRDS("Prepared_data_and_models/GAMM_hatched_eggs.23.RDS")
summary(gamm.hatched.eggs.23)
```

```

Family: quasibinomial
Link function: logit

Formula:
cbind(hatched, non.hatched) ~ s(yday, by = municipality.fac,
    pc = pc.23) + municipality.fac + s(unique.ID, bs = "re")

Parametric coefficients:
                        Estimate Std. Error t value Pr(>|t|)
(Intercept)               2.1233     0.2042  10.399  < 2e-16 ***
municipality.facMorcote  -1.1531     0.2272  -5.076 5.75e-07 ***
---
Signif. codes:  0 '***' 0.001 '**' 0.01 '*' 0.05 '.' 0.1 ' ' 1
```

```
Approximate significance of smooth terms:
                                      edf Ref.df     F  p-value    
s(yday):municipality.facCaslano   2.779  3.437 3.981 0.005715 ** 
s(yday):municipality.facMorcote   3.219  3.977 3.179 0.013743 *  
s(unique.ID)                     22.252 57.000 0.738 0.000602 ***
---
Signif. codes:  0 '***' 0.001 '**' 0.01 '*' 0.05 '.' 0.1 ' ' 1

R-sq.(adj) =  0.266   Deviance explained =   35%
-REML = -64.547  Scale est. = 5.7957    n = 460
```

We also extract the standard deviation corresponding to the random effect.

```r
gam.vcomp(gamm.hatched.eggs.23) %>%
  tail(n = 1)
```

```
Standard deviations and 0.95 confidence intervals:

                                    std.dev        lower      upper
s(yday):municipality.facCaslano 0.004589059 0.0014252781 0.01477568
s(yday):municipality.facMorcote 0.002861387 0.0007738984 0.01057960
s(unique.ID)                    0.302575045 0.1867103784 0.49034049
scale                           2.443562358 2.2783607868 2.62074252

Rank: 4/4

       std.dev    lower    upper
scale 2.443562 2.278361 2.620743
```

5.3 Plotting the model

We now plot the fitted model. Since we allowed the model to fit a different trend for each municipality, there will be a separate plot for each one.

```r
print(plot(gamm.hatched.eggs.23,
           trans = plogis),
      pages = 1)
```

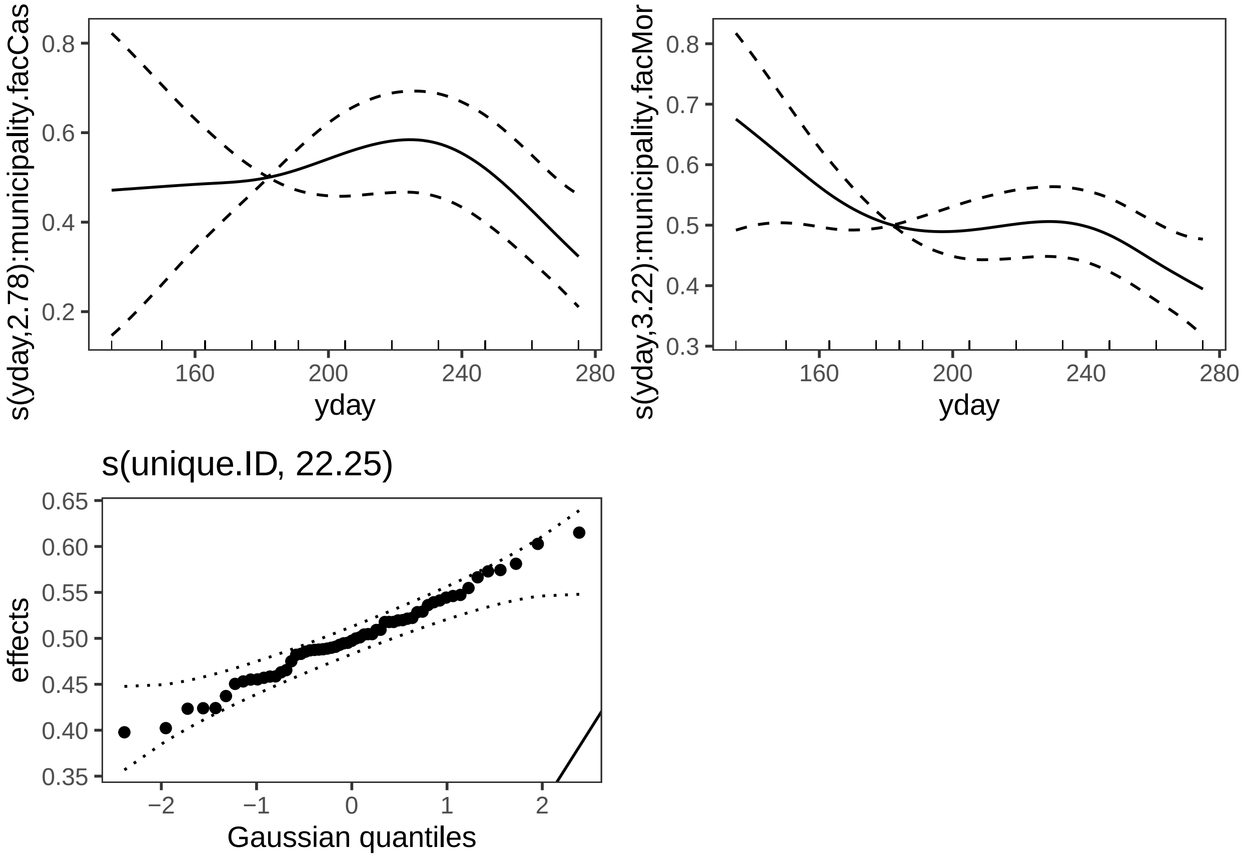

We can draw the graphs on the same plot with confidence intervals. This is performed to compare the different shapes.

```
## Extract data from the plots
gamm.hatched.eggs.23.plot.tmp <- lapply(plot(gamm.hatched.eggs.23)$plots,
                                        function(x) x$data$fit)
##
gamm.hatched.eggs.23.plot.tmp <- lapply(1:(length(gamm.hatched.eggs.23.plot.tmp)-1),
                                        function(ii) {
                                          out <- gamm.hatched.eggs.23.plot.tmp[[ii]]
                                          out$municipality <- ii
                                          return(out)
                                        } )
##
## Combine data in a unique data set
gamm.hatched.eggs.23.plot <- do.call("rbind", gamm.hatched.eggs.23.plot.tmp)
##
## Create a factor for the municipality group
gamm.hatched.eggs.23.plot$municipality <- as.factor(gamm.hatched.eggs.23.plot$municipality)
##
## Create CI
gamm.hatched.eggs.23.plot$upper <- gamm.hatched.eggs.23.plot$ty + 2 * gamm.hatched.eggs.23.plot$se
gamm.hatched.eggs.23.plot$lower <- gamm.hatched.eggs.23.plot$ty - 2 * gamm.hatched.eggs.23.plot$se
##
## Plot the data
ggplot(data = gamm.hatched.eggs.23.plot, mapping = aes(x = x, y = ty,
```

```
                                                     colour = municipality,
                                                     group = municipality,
                                                     fill = municipality)) +
  geom_line() +
  geom_ribbon(data = subset(gamm.hatched.eggs.23.plot, lower < y & y < upper),
              aes(ymin = lower, ymax = upper),
              alpha = 0.3,
              lty = 2) +
  coord_cartesian(ylim = c(-1, 2))
```

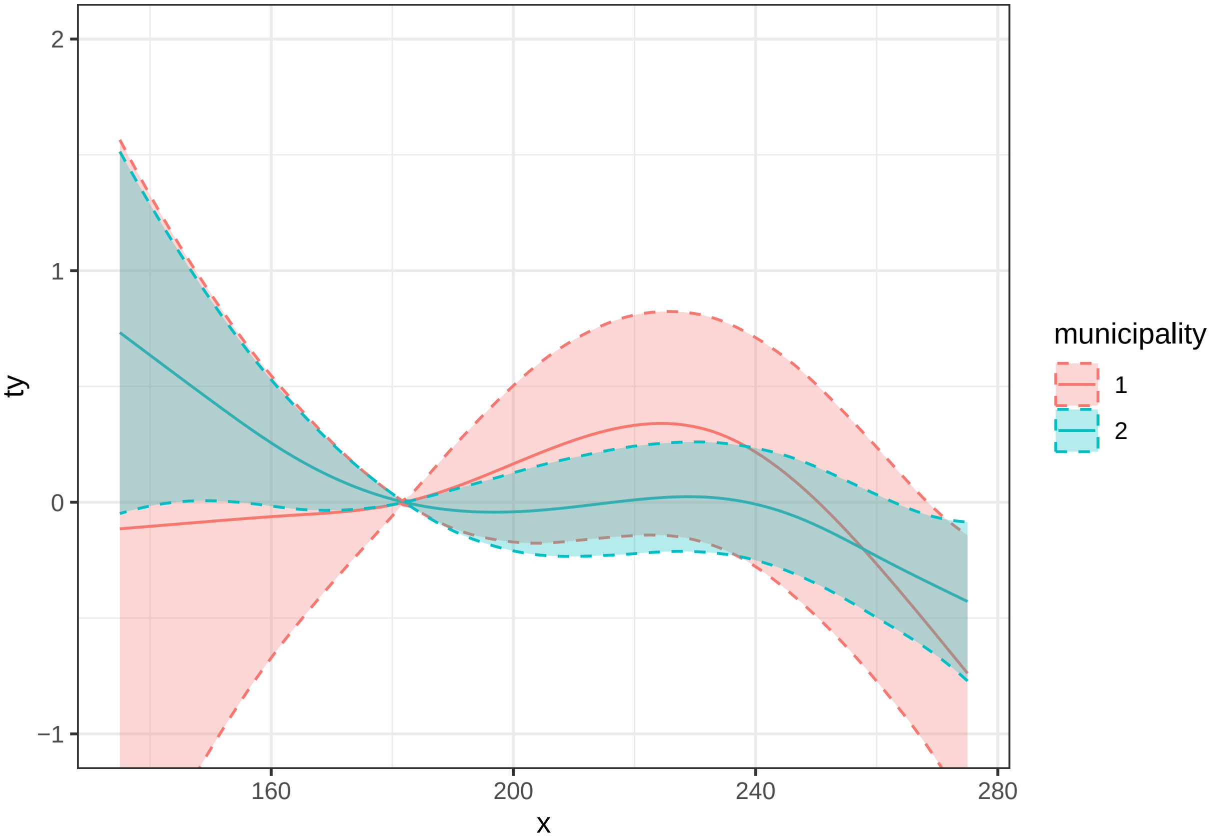

From this graph, allowing different shapes for the smoothers seems necessary. We will formally verify this later in the sections.

We draw again the same plots, separately, but adding the correct shift to all of them.

```
plot.gam(gamm.hatched.eggs.23,
         trans = plogis,
         select = 1,
         shift = coef(gamm.hatched.eggs.23)["(Intercept)"],
         main = levels(d.ovitraps.23.mod$municipality.fac)[1])
abline(h = c(0, 1), col = "grey", lty = 1)
```

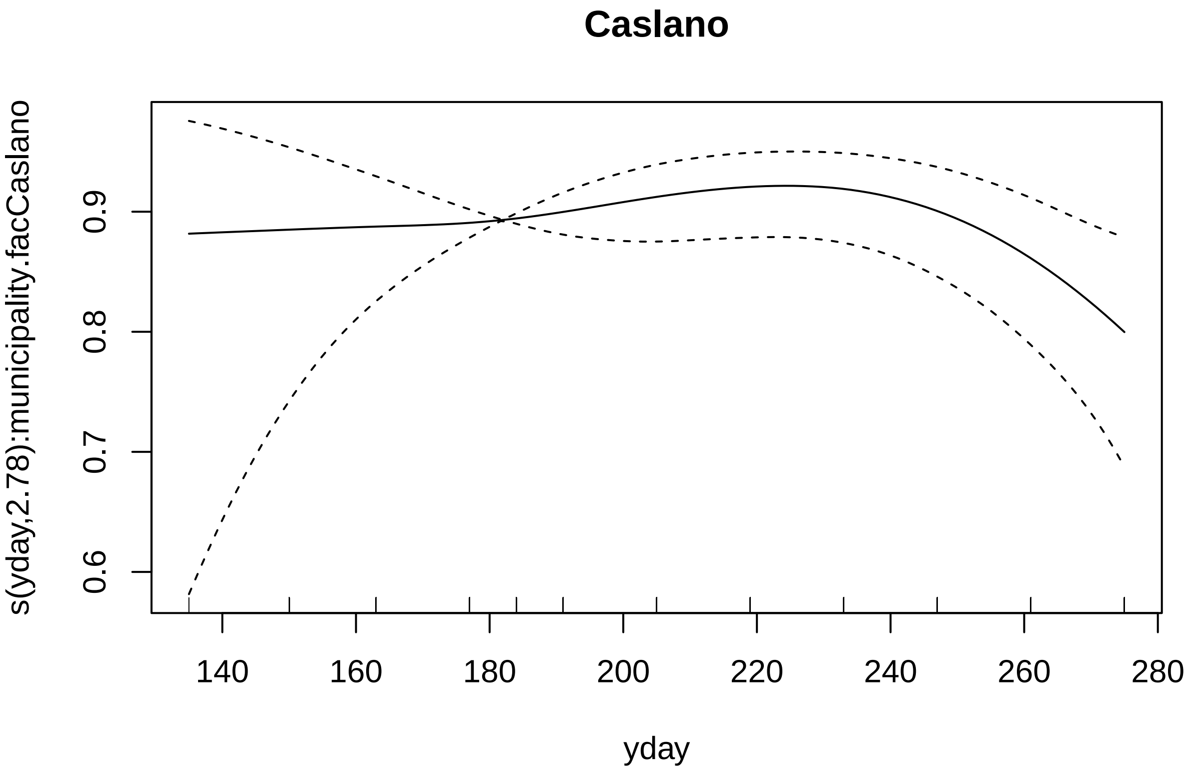


```
##
for (i in 2:nlevels(d.ovitraps.23.mod$municipality.fac)) {

  plot.gam(gamm.hatched.eggs.23,
           trans = plogis,
           select = i,
           shift = coef(gamm.hatched.eggs.23)["(Intercept)"] + coef(gamm.hatched.eggs.23)[i],
           main =levels(d.ovitraps.23.mod$municipality.fac)[i])
  abline(h = c(0, 1), col = "grey", lty = 1)

}
```

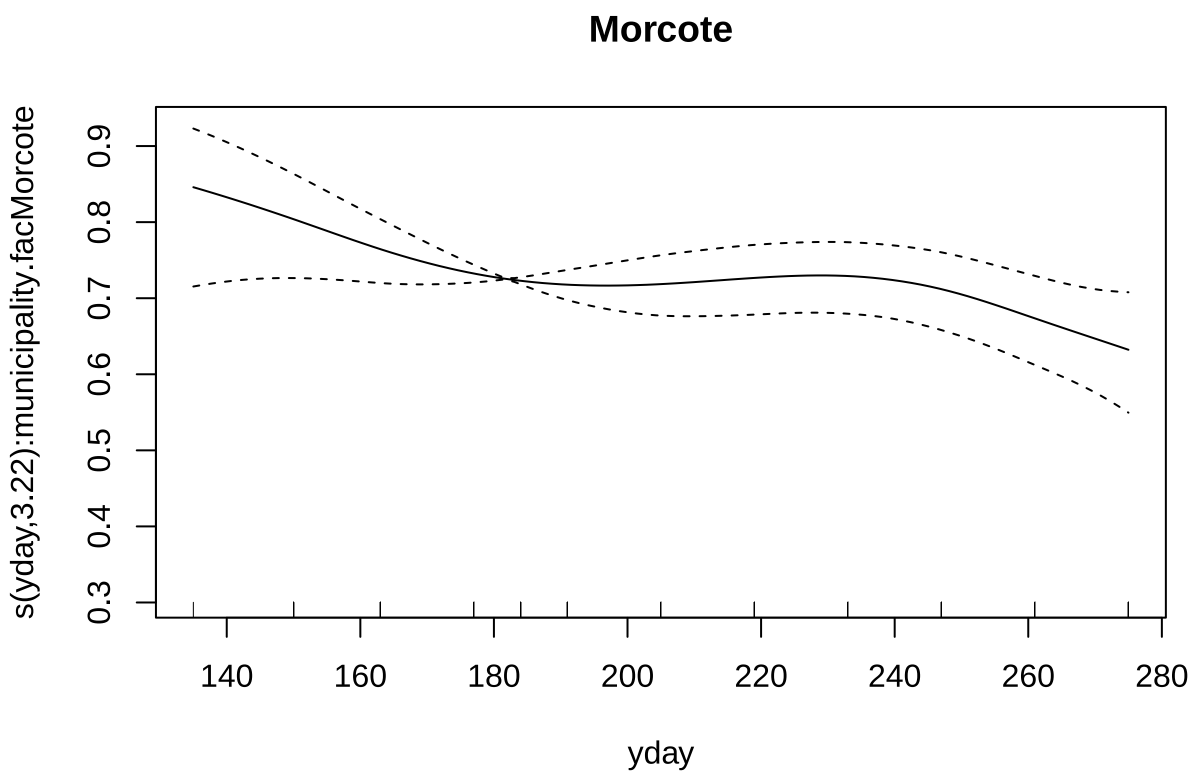

5.4 Fitted values

First, we begin by plotting the fitted values over time, creating a separate graph for each municipality. Observations from the same ovitrap will be connected by lines to visualise the trends.

```
d.ovitraps.23.mod$fitted_gamm.hatched.eggs.23 <- fitted(gamm.hatched.eggs.23)
##
ggplot(data = d.ovitraps.23.mod,
       mapping = aes(y = fitted_gamm.hatched.eggs.23,
                     x = Sampling_date.date,
                     group = unique.ID)) +
  geom_hline(yintercept = c(0, 1)) +
  geom_point(alpha = 0.1) +
  geom_line(alpha = 0.1) +
  scale_y_sqrt() +
  facet_wrap(~municipality.fac)
```

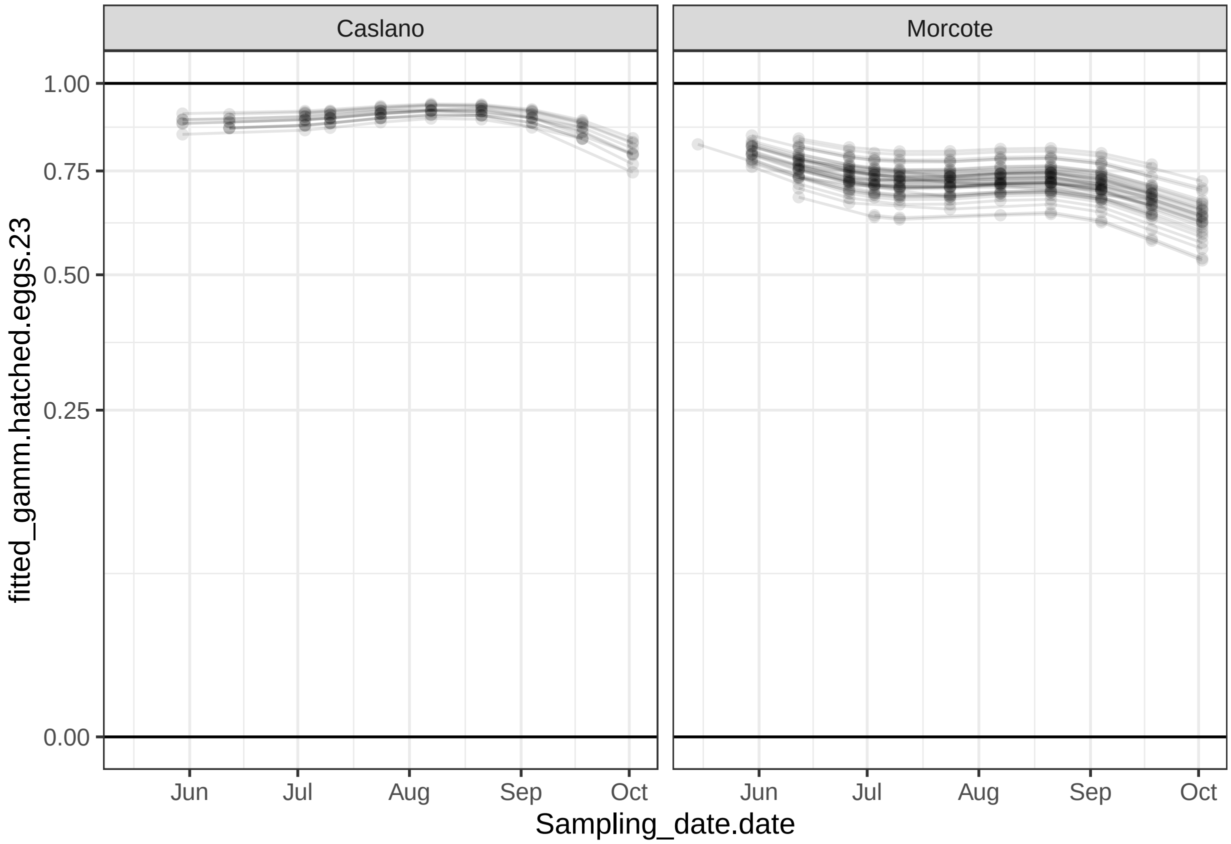

Note that there is quite some variability among ovitraps within Morcote.

We plot the three municipalities on the same graph to facilitate comparison.

```
ggplot(data = d.ovitraps.23.mod,
       mapping = aes(y = fitted_gamm.hatched.eggs.23,
                     x = Sampling_date.date,
                     group = unique.ID,
                     colour = municipality.fac)) +
  geom_point(alpha = 0.3) +
  geom_line(alpha = 0.3) +
  geom_hline(yintercept = c(0,1))
```

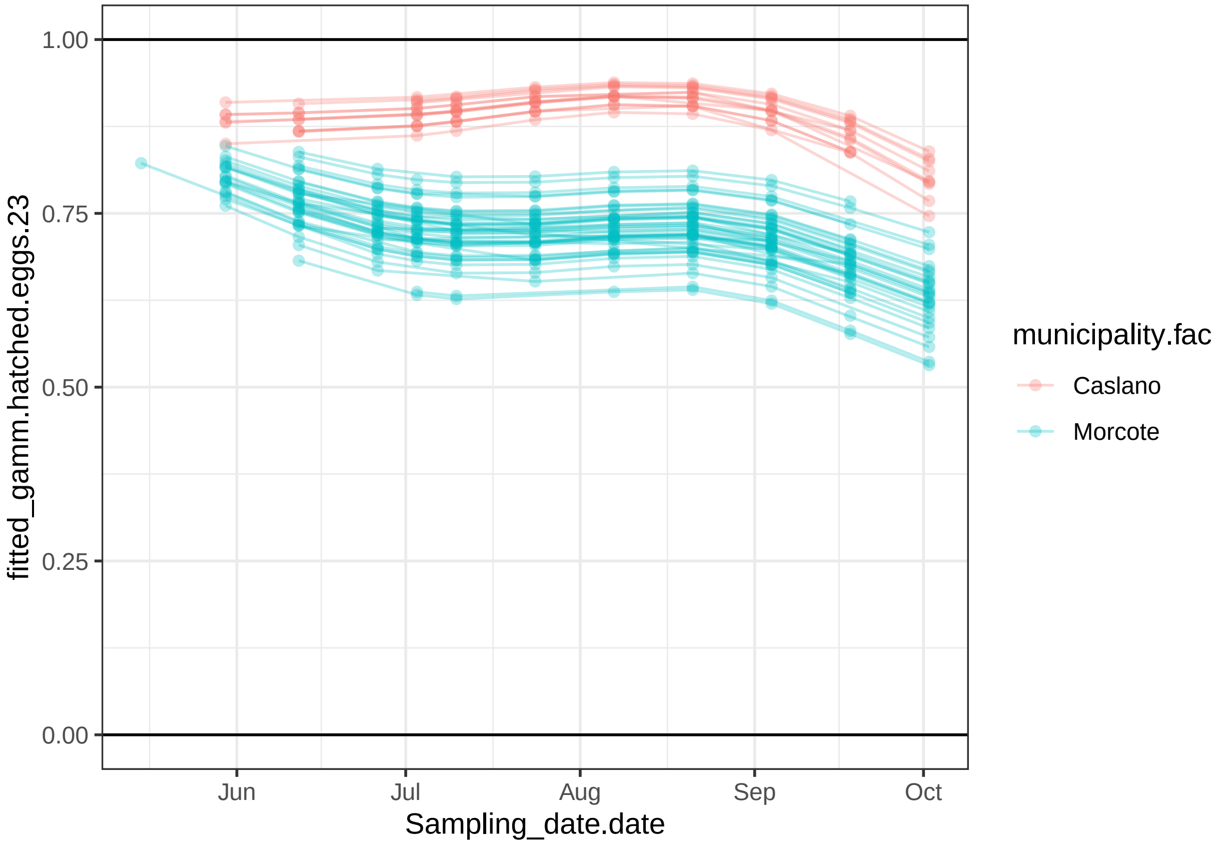

The fitted values for Morcote are systematically lower than Caslano.

5.5 Predicted values

Let's create the data set to make predictions on.

```
formula(gamm.hatched.eggs.23)
```

```
cbind(hatched, non.hatched) ~ s(yday, by = municipality.fac,
    pc = pc.23) + municipality.fac + s(unique.ID, bs = "re")
```

```
##
## 1. We create a dataset with municipality and ovitrap ID
d.muni.ovitrap.short <- d.ovitraps.23.mod %>%
  select(municipality.fac, unique.ID) %>%
  unique()
##
head(d.muni.ovitrap.short)
```

```
# A tibble: 6 x 2
  municipality.fac unique.ID
  <fct>            <fct>
1 Morcote          Morcote.32a
2 Morcote          Morcote.1a
3 Morcote          Morcote.4a
4 Morcote          Morcote.5a
5 Morcote          Morcote.6a
6 Morcote          Morcote.14a
```

```r
nrow(d.muni.ovitrap.short)
```

```
[1] 59
```

```r
##
## 2. We create dataset with varying yday and ovitrap ID
d.pred.gamm <- expand.grid(
  yday = seq(from = min(d.ovitraps.23.mod$yday),
             to = max(d.ovitraps.23.mod$yday),
             length.out = 100),
  # municipality = unique(d.modelling$municipality),
  unique.ID = d.muni.ovitrap.short$unique.ID)
##
## 3. We join the two datasets
## note that not all ovitraps exist in all municipalities.
## So, we can't simply use expand.grid()
d.pred.gamm_aug <- left_join(d.pred.gamm, d.muni.ovitrap.short,
                             by = join_by("unique.ID"))
str(d.pred.gamm_aug)
```

```
'data.frame':   5900 obs. of  3 variables:
 $ yday            : num  135 136 138 139 141 ...
 $ unique.ID       : Factor w/ 59 levels "Caslano.10a",..: 32 32 32 32 32 32 32 32 32 32 ...
 $ municipality.fac: Factor w/ 2 levels "Caslano","Morcote": 2 2 2 2 2 2 2 2 2 2 ...
 - attr(*, "out.attrs")=List of 2
  ..$ dim     : Named int [1:2] 100 59
  .. ..- attr(*, "names")= chr [1:2] "yday" "unique.ID"
  ..$ dimnames:List of 2
  .. ..$ yday     : chr [1:100] "yday=135.0000" "yday=136.4141" "yday=137.8283" "yday=139.2424" ...
  .. ..$ unique.ID: chr [1:59] "unique.ID=Morcote.32a" "unique.ID=Morcote.1a" "unique.ID=Morcote.4a" "un
```

We make the predictions on the newly created data set, at ovitrap and population level, and we plot the result.

```r
## Predictions at ovitrap level
d.pred.gamm_aug$predicted_gamm.hatched.eggs.23 <- predict(
  gamm.hatched.eggs.23,
  newdata = d.pred.gamm_aug,
  type = "response")
##
## Predictions at population level
d.pred.gamm_aug$predicted_gamm.hatched.eggs.23.pop <- predict(
  gamm.hatched.eggs.23,
  newdata = d.pred.gamm_aug,
  type = "response",
  exclude = 's(unique.ID)')
##
p <- ggplot(data = d.pred.gamm_aug,
            mapping = aes(y = predicted_gamm.hatched.eggs.23,
                          x = yday,
                          group = unique.ID)) +
  geom_line(alpha = 0.2) +
  geom_line(mapping = aes(y = predicted_gamm.hatched.eggs.23.pop),
            colour = "red",
            linewidth = 1) +
```

```
  geom_hline(yintercept = c(0, 1)) +
  facet_wrap(~municipality.fac)
p
```

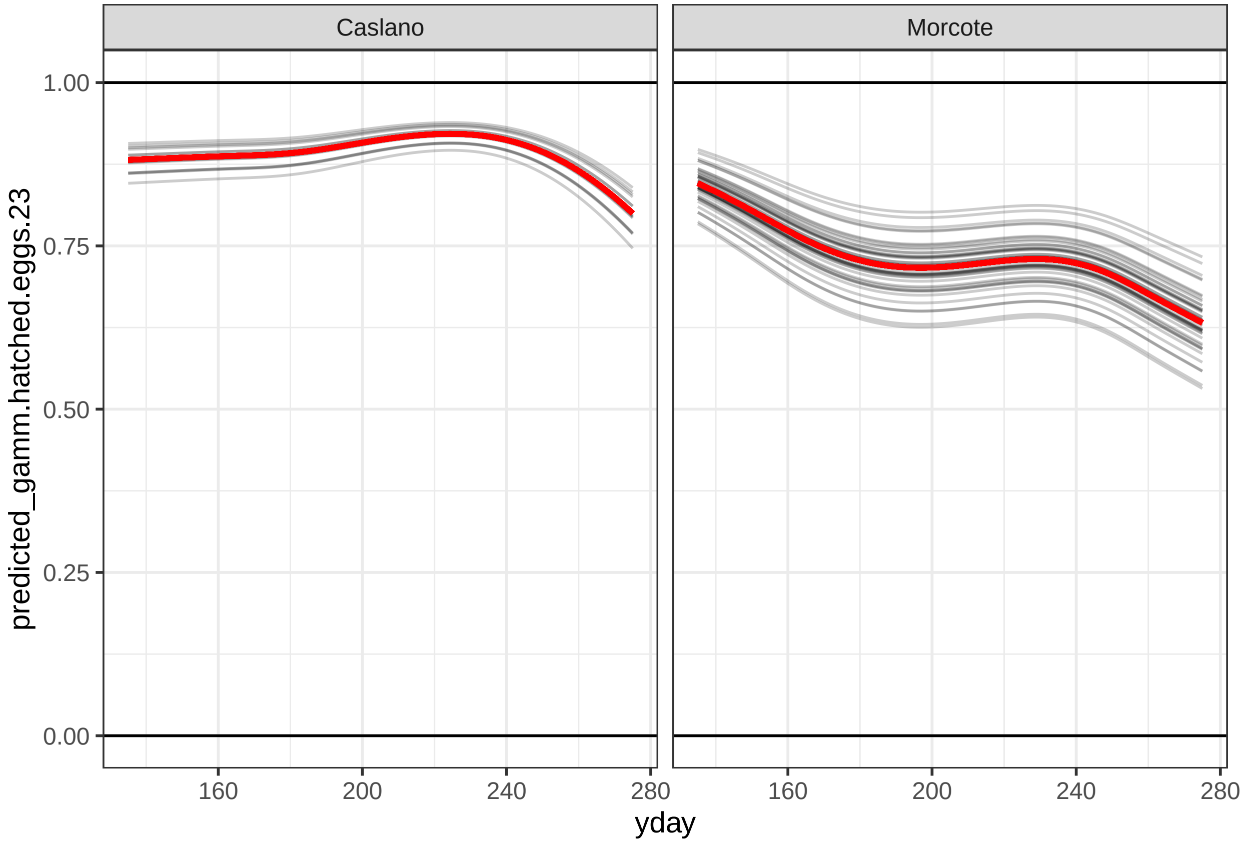


```
## save plot for future use:
saveRDS(p, file = file.path("saved_figures",
                            "2b_MakePredictionGammHatchedEggs_CM.rds"))
```

Each black line corresponds to the prediction for a given ovitrap, whereas the red line corresponds to the prediction at population level.

The shapes can be quite different between municipalities. The variability within Morcote is also quite significant.
Note that Vico Morcote has fewer observations than the other municipalities. Also, the measurements started later in the season and stopped earlier for this municipality, so the predicted values at the extremes cannot be reliable.

We plot the three predictions at population level on the same plot.

```
p <- ggplot(data = d.pred.gamm_aug,
            mapping = aes(y = predicted_gamm.hatched.eggs.23.pop,
                          x = yday,
                          colour = municipality.fac)) +
  geom_line() +
  geom_hline(yintercept = c(0, 1))
p
```

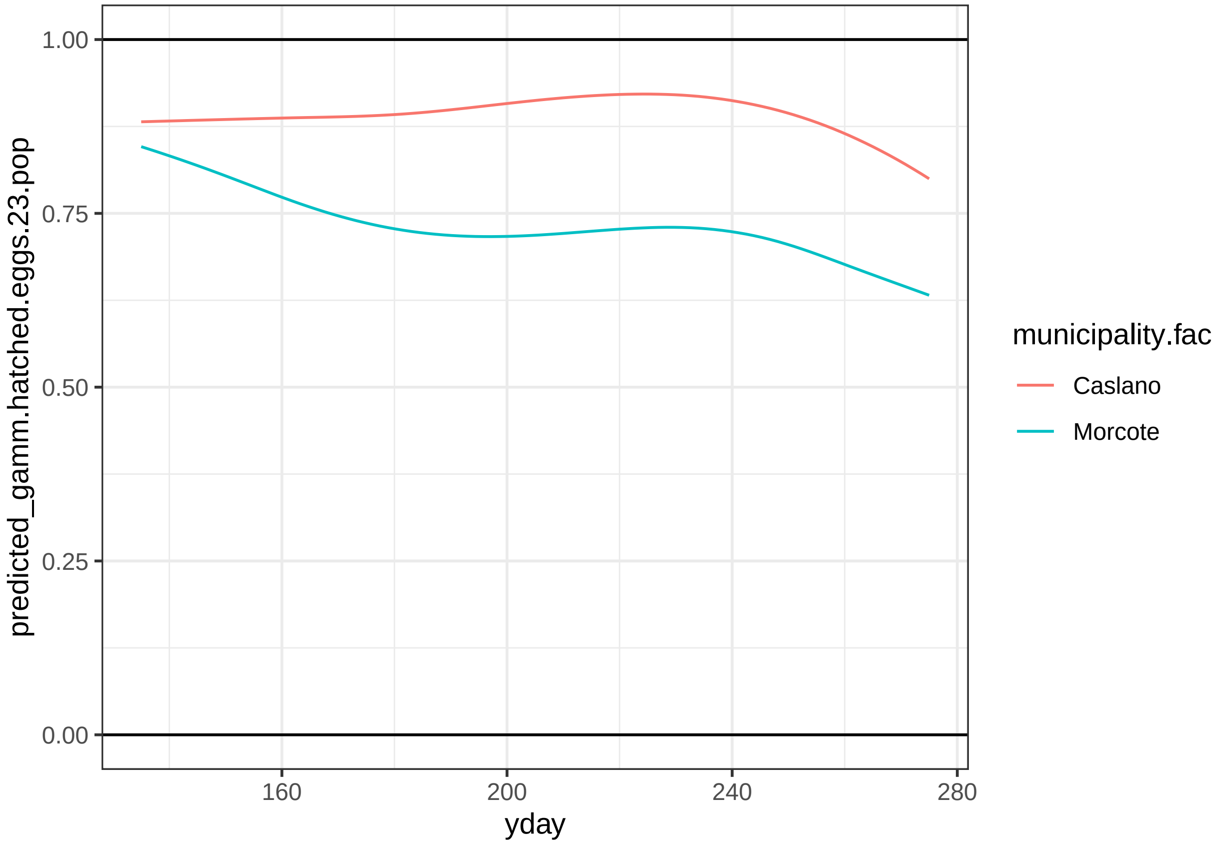


```
## save plot for future use:
saveRDS(p, file = file.path("saved_figures",
                            "2b_MakePredictionGammHatchedEggs_CM_popLevel.rds"))
```

As already mentioned, Vico Morcote has fewer observations than the other municipalities, in particular at the beginning and end of the season, therefore the predictions are not reliable. However, for Caslano and Morcote there is a systematic difference, with Morcote systematically lower than Caslano.

5.6 Model selection – Shape

We fitted a model allowing a different smoother for each municipality. Now, we will assess whether this flexibility is truly necessary.

With this purpose in mind, we refit the model, but this time without allowing for different shapes across municipalities. We will then compare the two models to determine if the difference between them is statistically significant.

For performing this comparison, we need to refit the first model using an ordered factor (*municipality.ord*) instead of the classical one. This allows us to have nested model matrices and formally compare the two models.

```
## (this chunk is not evaluated)
gamm.hatched.eggs.23.ord <- gam(
  cbind(hatched, non.hatched) ~
    s(unique.ID, bs = "re") +
    municipality.fac +
    s(yday, pc = pc.23) +
```

```r
    s(yday, by = municipality.ord, pc = pc.23),
  family = "quasibinomial",
  data = d.ovitraps.23.mod)
##
saveRDS(gamm.hatched.eggs.23.ord,
        "Prepared_data_and_models/GAMM_hatched_eggs_ord.23.RDS")
##
gamm.hatched.eggs.23.smooth <- gam(
  cbind(hatched, non.hatched) ~
    s(unique.ID, bs = "re") +
    municipality.fac +
    s(yday, pc = pc.23),

  # s(yday, by = municipality.ord) +,
  family = "quasibinomial",
  data = d.ovitraps.23.mod)
##
saveRDS(gamm.hatched.eggs.23.smooth,
        "Prepared_data_and_models/GAMM_hatched_eggs_one_smooth.23.RDS")
```

Let's get the previously fitted models.

```r
gamm.hatched.eggs.23.ord <- readRDS(paste0("Prepared_data_and_models/",
                                           "GAMM_hatched_eggs_ord.23.RDS"))
gamm.hatched.eggs.23.smooth <- readRDS(paste0("Prepared_data_and_models/",
                                              "GAMM_hatched_eggs_one_smooth.23.RDS"))
##
summary(gamm.hatched.eggs.23.ord)
```

```

Family: quasibinomial
Link function: logit

Formula:
cbind(hatched, non.hatched) ~ s(unique.ID, bs = "re") + municipality.fac +
    s(yday, pc = pc.23) + s(yday, by = municipality.ord, pc = pc.23)

Parametric coefficients:
                        Estimate Std. Error t value Pr(>|t|)
(Intercept)               2.1473     0.2003  10.723  < 2e-16 ***
municipality.facMorcote  -1.2283     0.2162  -5.683 2.46e-08 ***
---
Signif. codes:  0 '***' 0.001 '**' 0.01 '*' 0.05 '.' 0.1 ' ' 1

Approximate significance of smooth terms:
                                   edf Ref.df     F  p-value
s(unique.ID)                    24.476 57.000 0.835 0.000317 ***
s(yday)                          3.915  4.778 3.420 0.006197 **
s(yday):municipality.ordMorcote  2.029  2.512 0.797 0.470958
---
Signif. codes:  0 '***' 0.001 '**' 0.01 '*' 0.05 '.' 0.1 ' ' 1

R-sq.(adj) =  0.273   Deviance explained = 35.9%
GCV = 6.3719  Scale est. = 5.7144    n = 460
```

```r
summary(gamm.hatched.eggs.23.smooth)
```

```

Family: quasibinomial
Link function: logit

Formula:
cbind(hatched, non.hatched) ~ s(unique.ID, bs = "re") + municipality.fac +
    s(yday, pc = pc.23)

Parametric coefficients:
                        Estimate Std. Error t value Pr(>|t|)
(Intercept)               2.1600     0.1517  14.237  < 2e-16 ***
municipality.facMorcote  -1.2354     0.1508  -8.192 2.97e-15 ***
---
Signif. codes:  0 '***' 0.001 '**' 0.01 '*' 0.05 '.' 0.1 ' ' 1

Approximate significance of smooth terms:
                edf Ref.df     F  p-value
s(unique.ID) 24.551 57.000 0.846 0.000258 ***
s(yday)       3.937  4.822 5.234 0.000168 ***
---
Signif. codes:  0 '***' 0.001 '**' 0.01 '*' 0.05 '.' 0.1 ' ' 1

R-sq.(adj) =  0.273   Deviance explained = 35.4%
GCV = 6.3638  Scale est. = 5.6755    n = 460
```

We check whether the two models are nested. If they are, then we can test whether the most complicated one is necessary, i.e., if the difference of the two models is statistically significant.

```r
m.gamm.hatched.eggs.23.ord <- model.matrix(gamm.hatched.eggs.23.ord)
m.gamm.hatched.eggs.23.smooth <- model.matrix(gamm.hatched.eggs.23.smooth) ##
dim(m.gamm.hatched.eggs.23.ord)
```

```
[1] 460  79
```

```r
dim(m.gamm.hatched.eggs.23.smooth)
```

```
[1] 460  70
```

```r
##
n1 <- ncol(m.gamm.hatched.eggs.23.smooth)
##
## check that sum is zero
sum(matrix( m.gamm.hatched.eggs.23.smooth[, 1:n1] -
              m.gamm.hatched.eggs.23.ord[, 1:n1] ) != 0)
```

```
[1] 0
```

The two model matrices are nested, therefore we can now compare the two models with the Chi-square test.

```r
anova.gam(gamm.hatched.eggs.23.smooth, gamm.hatched.eggs.23.ord, test = "Chisq")
```

```
Analysis of Deviance Table

Model 1: cbind(hatched, non.hatched) ~ s(unique.ID, bs = "re") + municipality.fac +
    s(yday, pc = pc.23)
Model 2: cbind(hatched, non.hatched) ~ s(unique.ID, bs = "re") + municipality.fac +
```

```
    s(yday, pc = pc.23) + s(yday, by = municipality.ord, pc = pc.23)
  Resid. Df Resid. Dev     Df Deviance Pr(>Chi)
1    416.42     2552.2
2    414.05     2532.5 2.3716   19.698   0.2312
```

The difference between the two models is statistically significant, which means that the additional flexibility, allowing different shapes for the smoother in each municipality, is necessary.

Please note that AIC and BIC cannot be calculated for models having a "quasi" family, thus we cannot double check the anova result with these additional tools.

5.7 Residual analysis

Note that the residual analysis is run on the main model (i.e., *gamm.hatched.eggs.23*).

First of all, we apply the `gam.check()` function to the model, which produces some diagnostic information.

```
par(mfrow = c(2, 2))
gam.check(gamm.hatched.eggs.23)
```

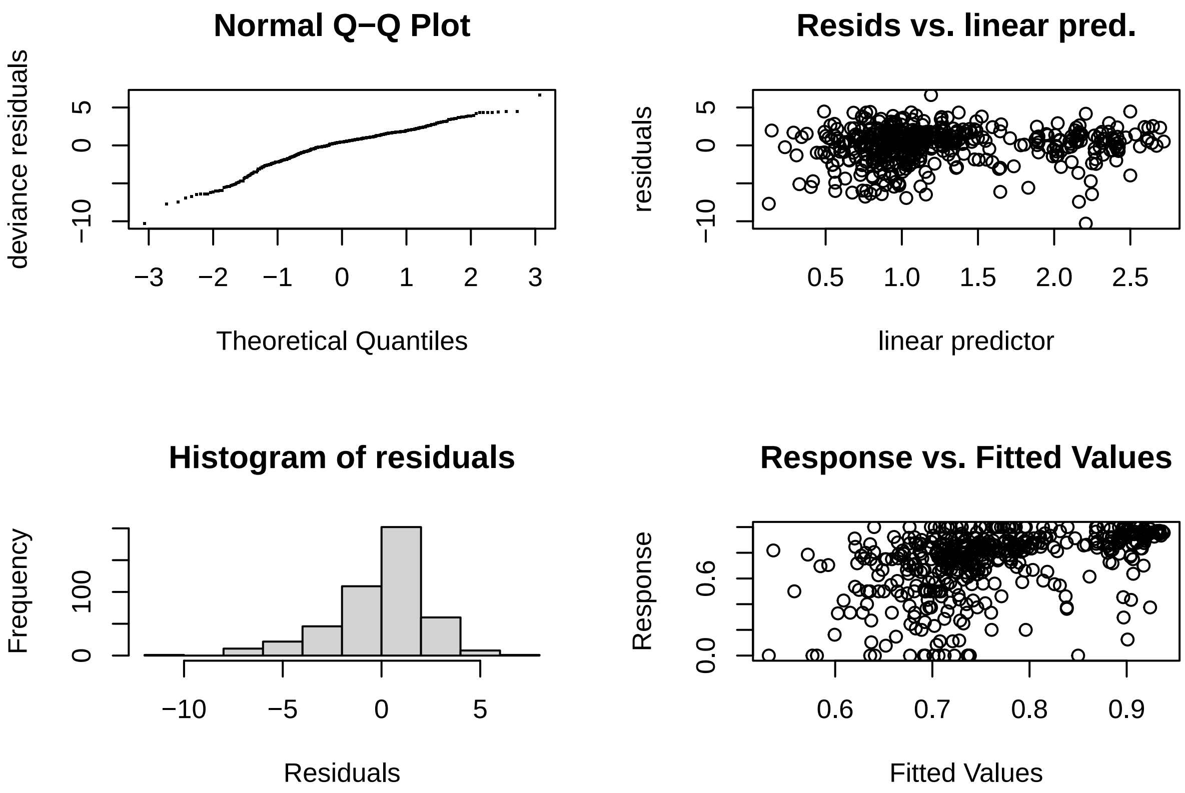


```
Method: REML   Optimizer: outer newton
full convergence after 5 iterations.
Gradient range [-3.595454e-05,1.945743e-06]
(score -64.54687 & scale 5.79569).
Hessian positive definite, eigenvalue range [0.5547283,228.5545].
Model rank =  79 / 79

Basis dimension (k) checking results. Low p-value (k-index<1) may
indicate that k is too low, especially if edf is close to k'.
```

```
                                      k'   edf k-index p-value
s(yday):municipality.facCaslano  9.00  2.78    1.05    0.84
s(yday):municipality.facMorcote  9.00  3.22    1.05    0.83
s(unique.ID)                    59.00 22.25      NA      NA
```

```r
par(mfrow = c(1, 1))
```

Then, we store the pearson residuals in the original data frame, and we plot the residuals against the fitted values to see whether there is still structure in the data.

First of all, we store the pearson residuals in the original data frame. Then we plot the residuals against the fitted values to see whether there is still structure in the data.

```r
d.ovitraps.23.mod$resid_gamm.hatched.eggs.23 <- resid(gamm.hatched.eggs.23,
                                                       type = "pearson")
##
ggplot(data = d.ovitraps.23.mod,
       mapping = aes(y = resid_gamm.hatched.eggs.23,
                     x = fitted_gamm.hatched.eggs.23)) +
  geom_hline(yintercept = 0) +
  geom_point(alpha = 0.2) +
  geom_smooth()
```

```
`geom_smooth()` using method = 'loess' and formula = 'y ~ x'
```

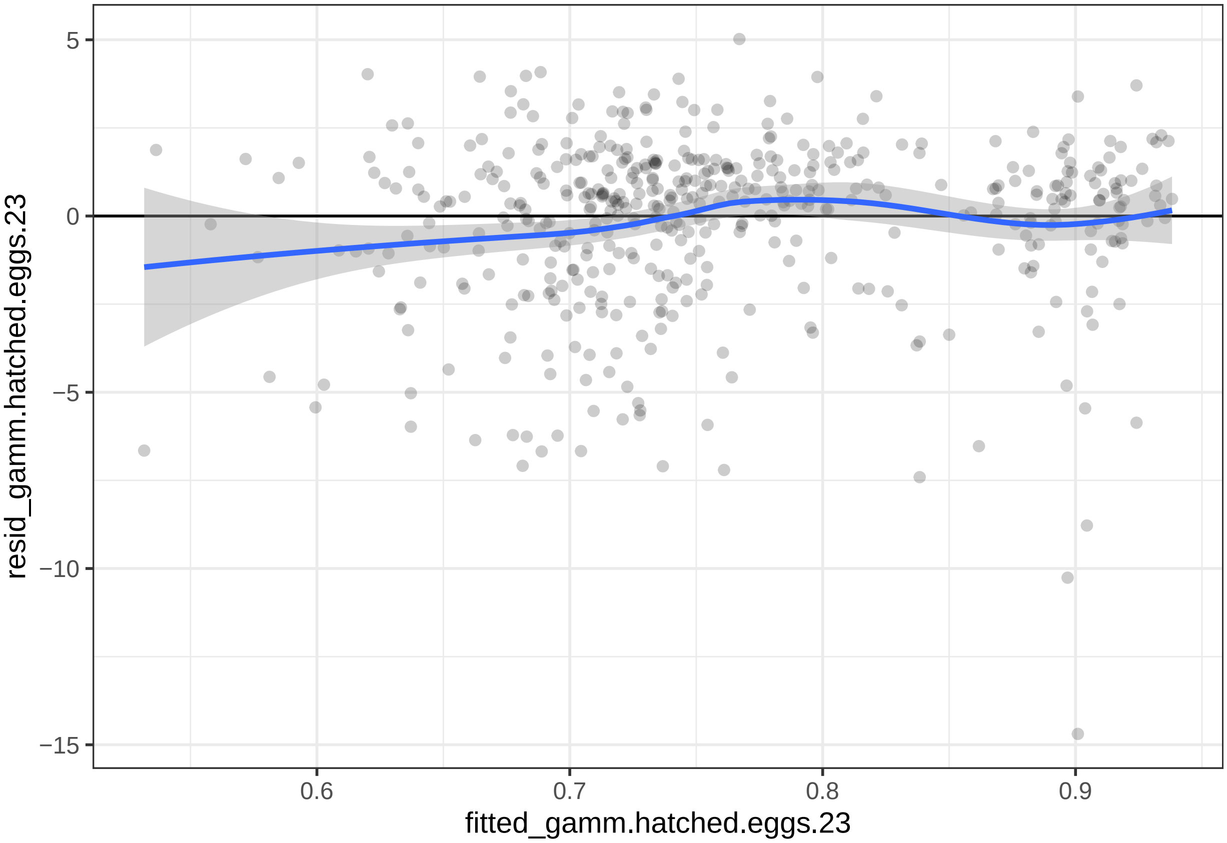

The blue line is located on the x-axis, indicating that there doesn't seem to be structure left in the residuals.

We plot the qq-plot for the random effects.

```r
## QQ for random effects
plot(sm(gamm.hatched.eggs.23, 3))
```

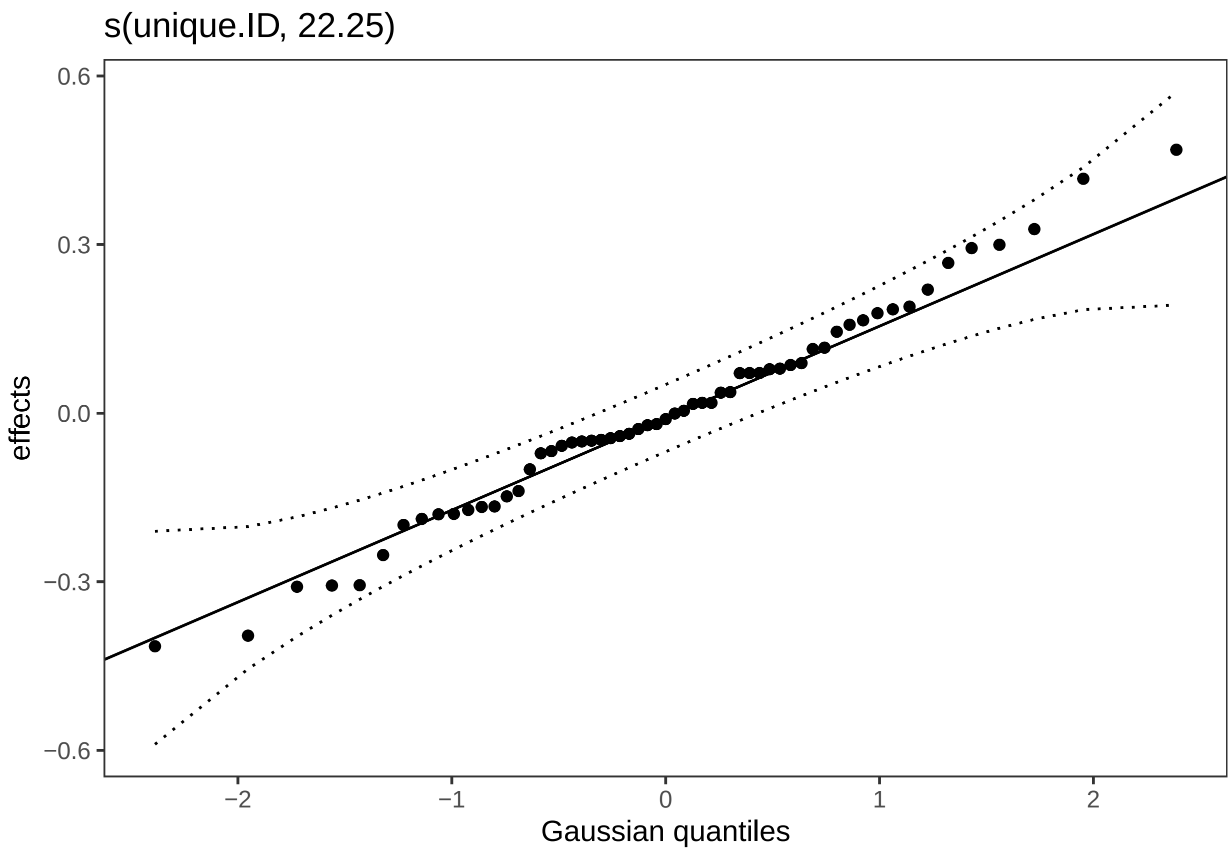

There isn't a significant departure from the normality assumption.

We now plot the residuals over time to further check whether there is any structure left in the data.

```r
ggplot(data = d.ovitraps.23.mod,
       mapping = aes(y = resid_gamm.hatched.eggs.23,
                     x = yday)) +
  geom_hline(yintercept = 0) +
  geom_point(alpha = 0.2) +
  geom_smooth(method = "loess")
```

```
`geom_smooth()` using formula = 'y ~ x'
```

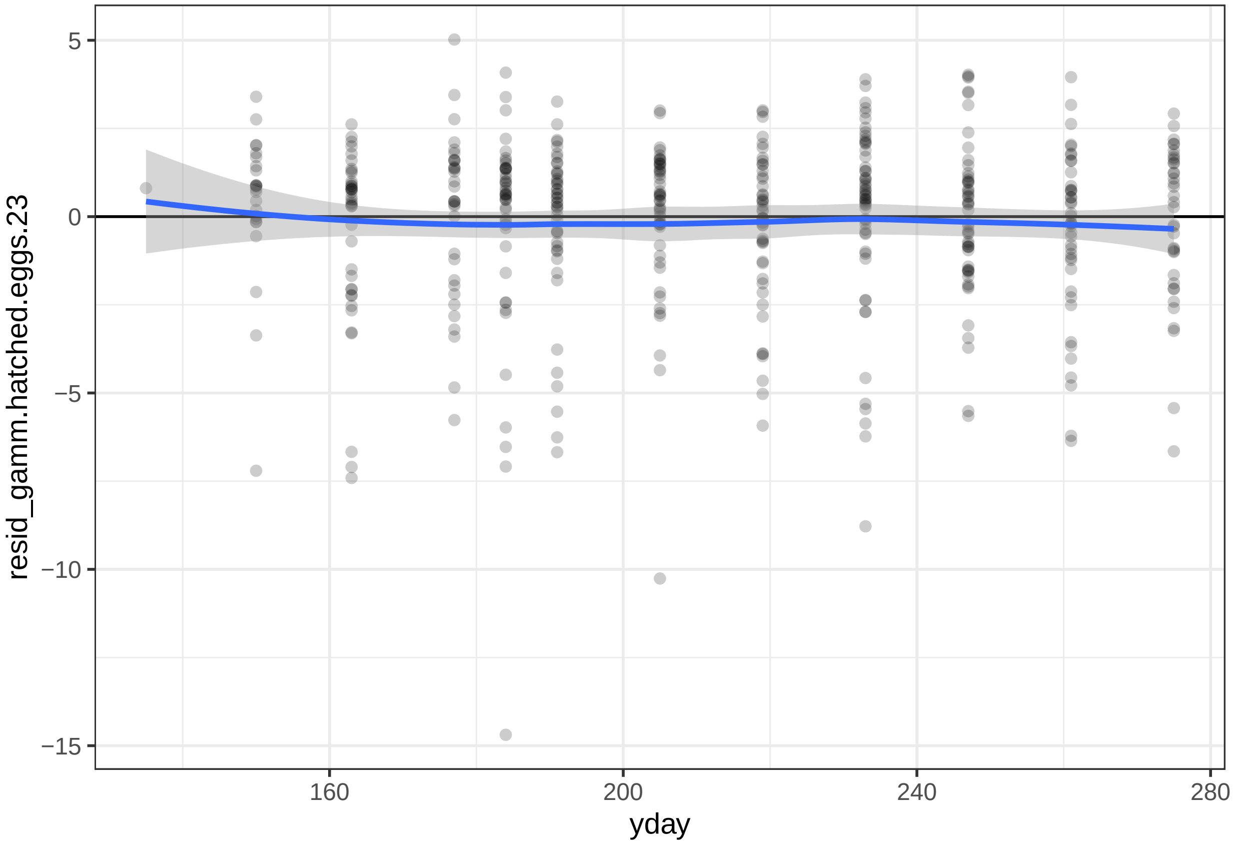

There doesn't seem to be any structure left in the data. Let's look into each single smoother (i.e. municipality).

```
ggplot(data = d.ovitraps.23.mod,
       mapping = aes(y = resid_gamm.hatched.eggs.23,
                     x = yday)) +
  geom_hline(yintercept = 0) +
  geom_point(alpha = 0.2) +
  geom_smooth(method = "loess") +
  facet_wrap(~municipality.fac, scales = "free")
```

```
`geom_smooth()` using formula = 'y ~ x'
```

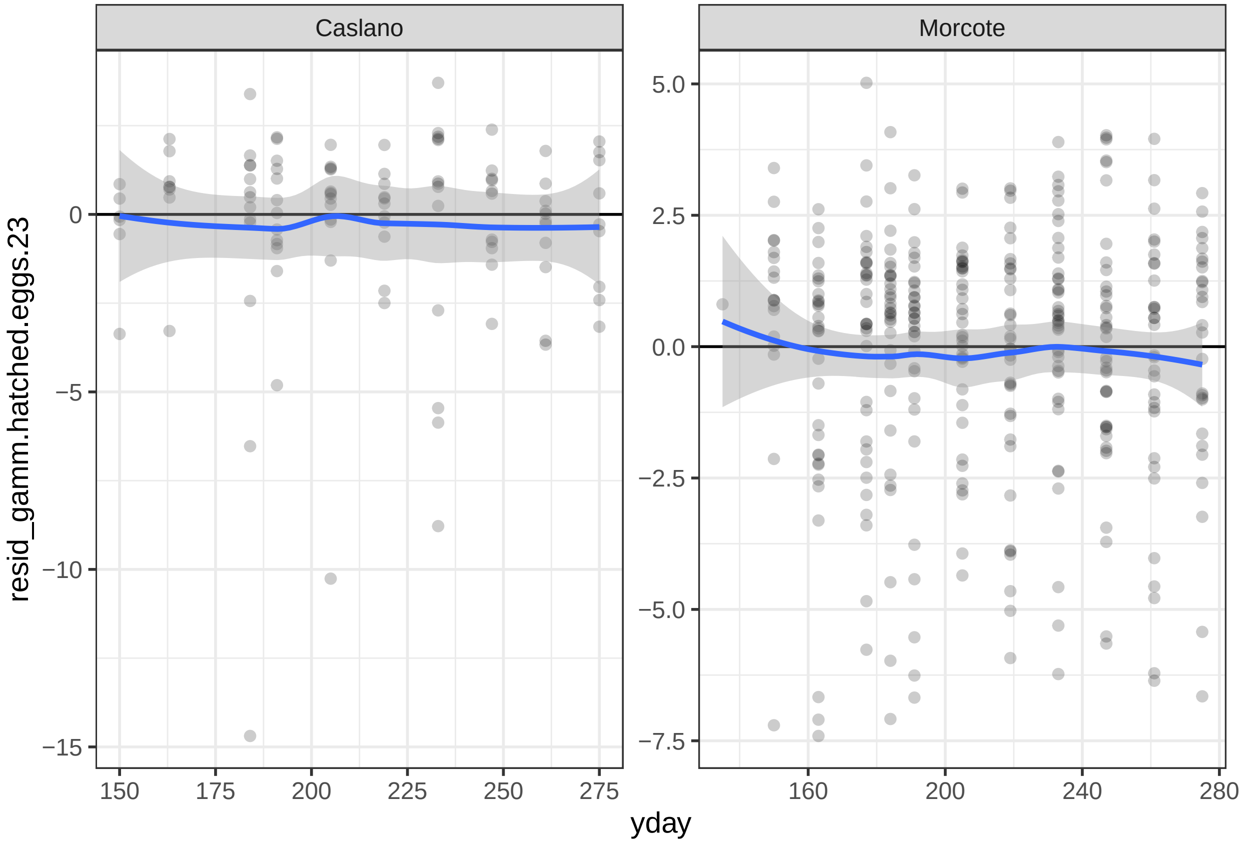

The blue lines are plotted on the x-axis, as desired.

The following two plots display the residuals in Morcote and Caslano, respectively.
The observations belonging to the same ovitrap are connected with a line. These plots are additionally drawn to check temporal correlation and variability.

```
## for Morcote
ggplot(data = filter(d.ovitraps.23.mod,
                     municipality.fac == "Morcote"),
       mapping = aes(y = resid_gamm.hatched.eggs.23,
                     x = yday,
                     group = unique.ID)) +
  geom_hline(yintercept = 0) +
  geom_point(alpha = 0.2) +
  geom_line(alpha = 0.2)
```

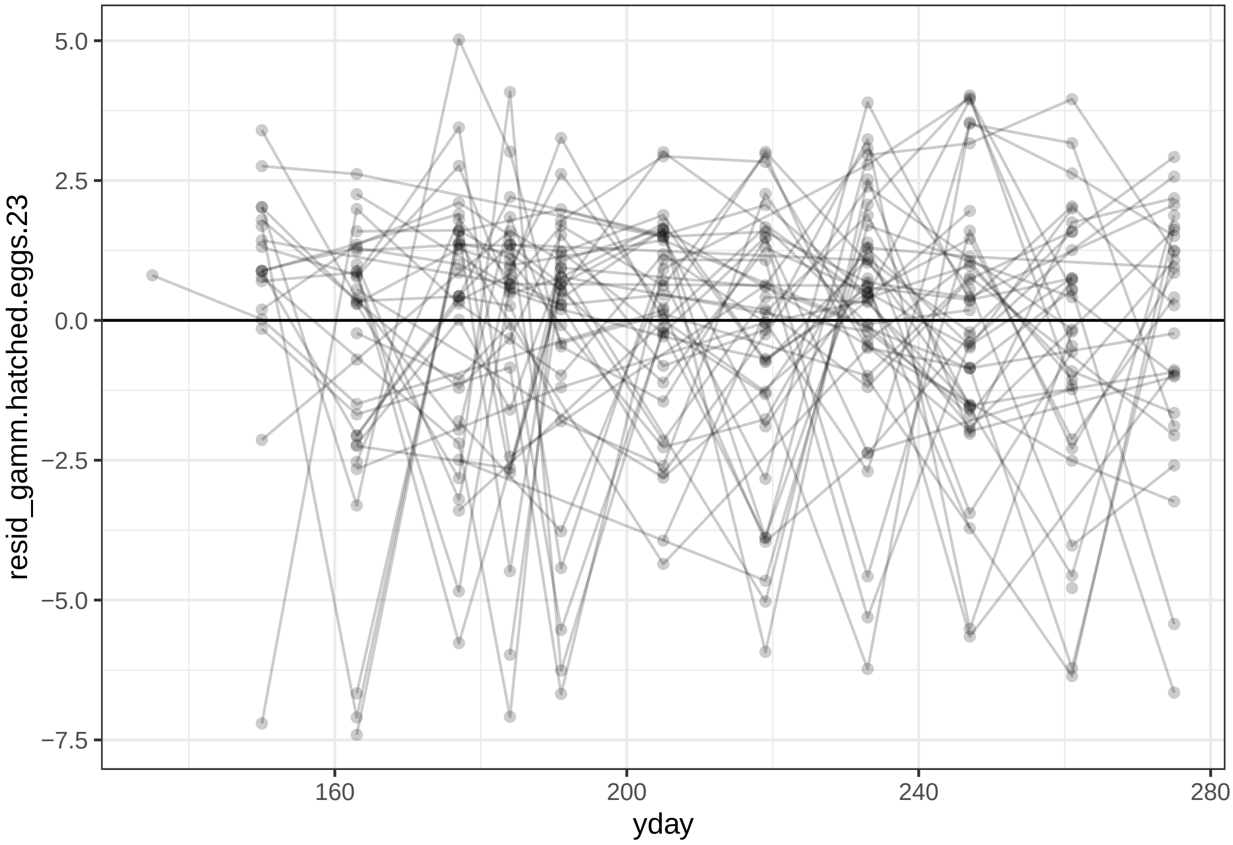


```
##
## for Caslano
ggplot(data = filter(d.ovitraps.23.mod,
                     municipality.fac == "Caslano"),
       mapping = aes(y = resid_gamm.hatched.eggs.23,
                     x = yday,
                     group = unique.ID)) +
  geom_hline(yintercept = 0) +
  geom_point(alpha = 0.2) +
  geom_line(alpha = 0.2)
```

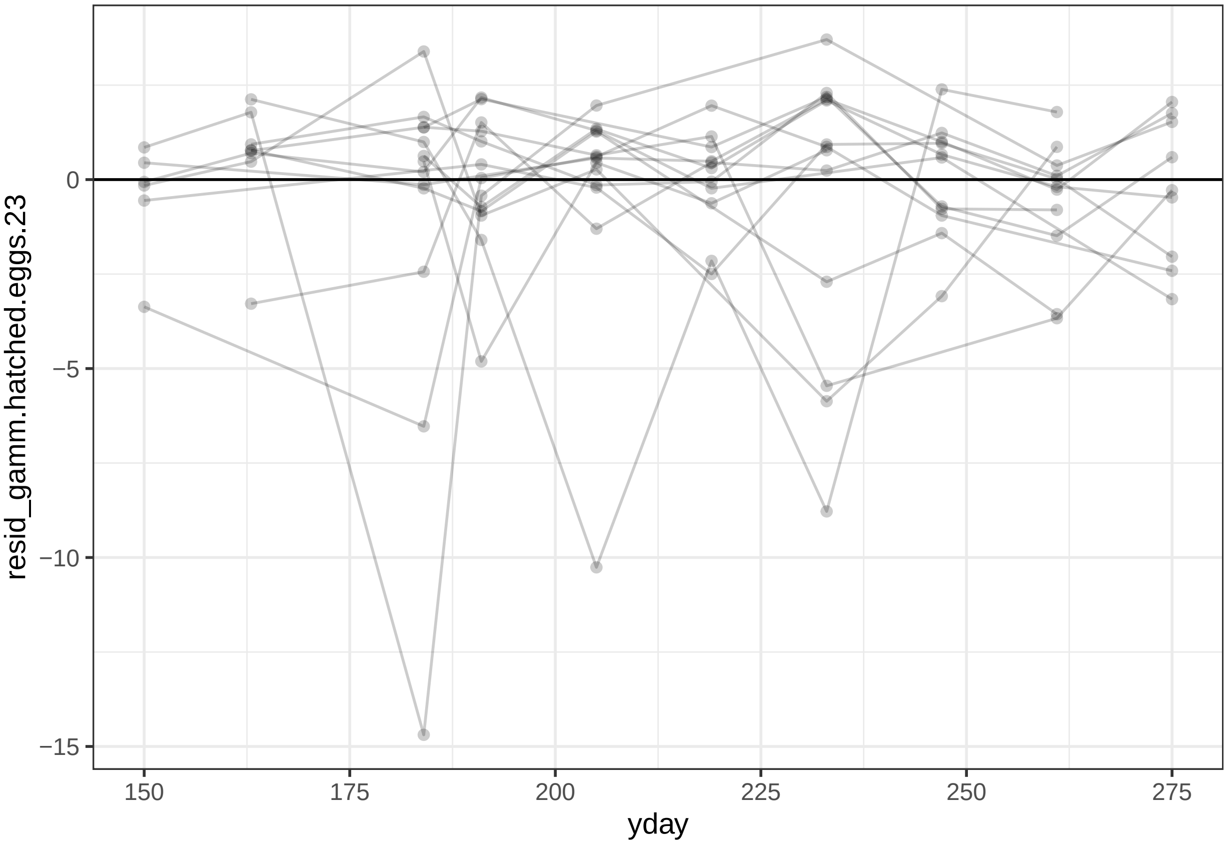

There seems to be some relevant variability between ovitraps.

Let's look at the single ovitraps.

```
## for Morcote
ggplot(data = filter(d.ovitraps.23.mod,
                     municipality.fac == "Morcote"),
       mapping = aes(y = resid_gamm.hatched.eggs.23,
                     x = yday,
                     group = unique.ID)) +
  geom_hline(yintercept = 0) +
  geom_point() +
  geom_line() +
  facet_wrap(~unique.ID) +
  theme(
    strip.background = element_blank(),
    strip.text.x = element_blank()) +
  coord_cartesian(ylim = c(-7.5, 3.5))
```

```
`geom_line()`: Each group consists of only one observation.
i Do you need to adjust the group aesthetic?
```

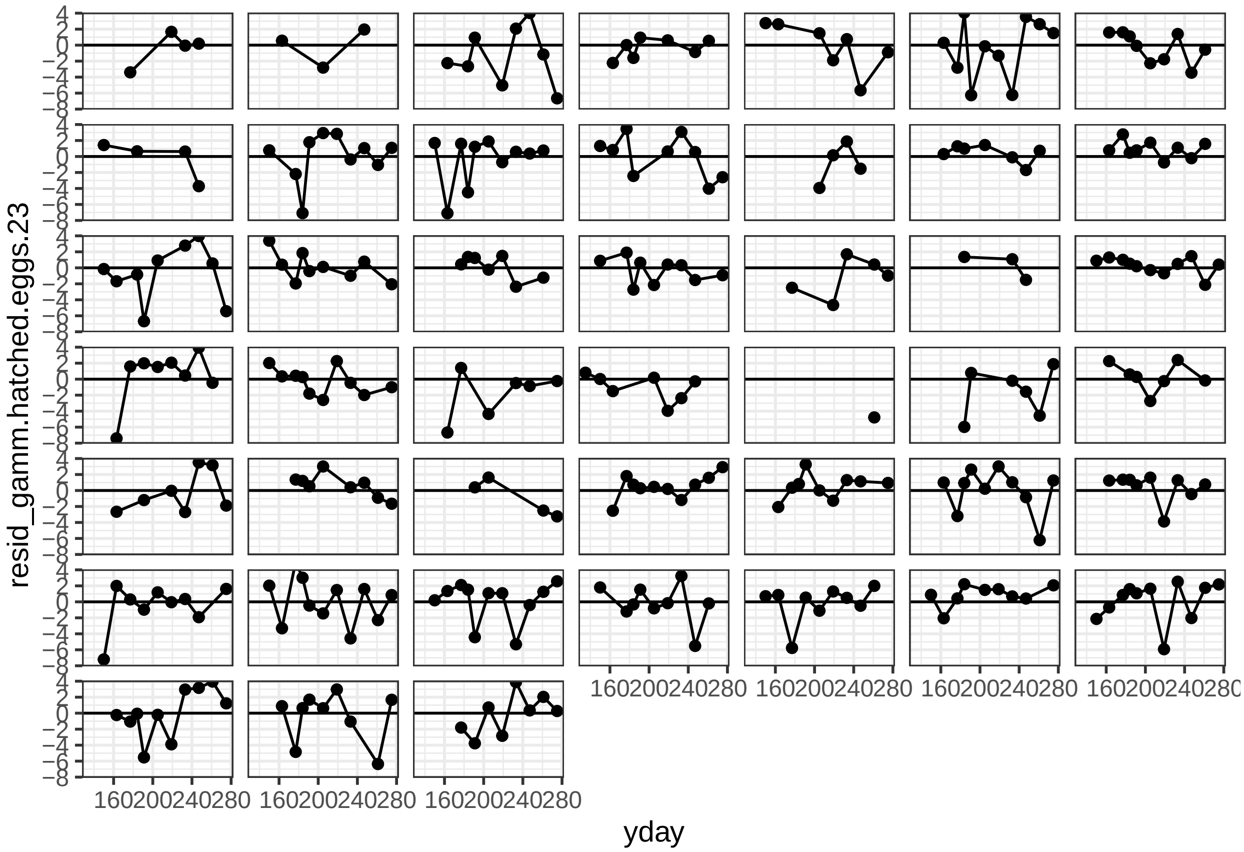


```r
##
## for Caslano
ggplot(data = filter(d.ovitraps.23.mod,
                     municipality.fac == "Caslano"),
       mapping = aes(y = resid_gamm.hatched.eggs.23,
                     x = yday,
                     group = unique.ID)) +
  geom_hline(yintercept = 0) +
  geom_point() +
  geom_line() +
  facet_wrap(~unique.ID, scales = "free_y") +
  theme(
    strip.background = element_blank(),
    strip.text.x = element_blank()) +
  coord_cartesian(ylim = c(-7.5, 3.5))
```

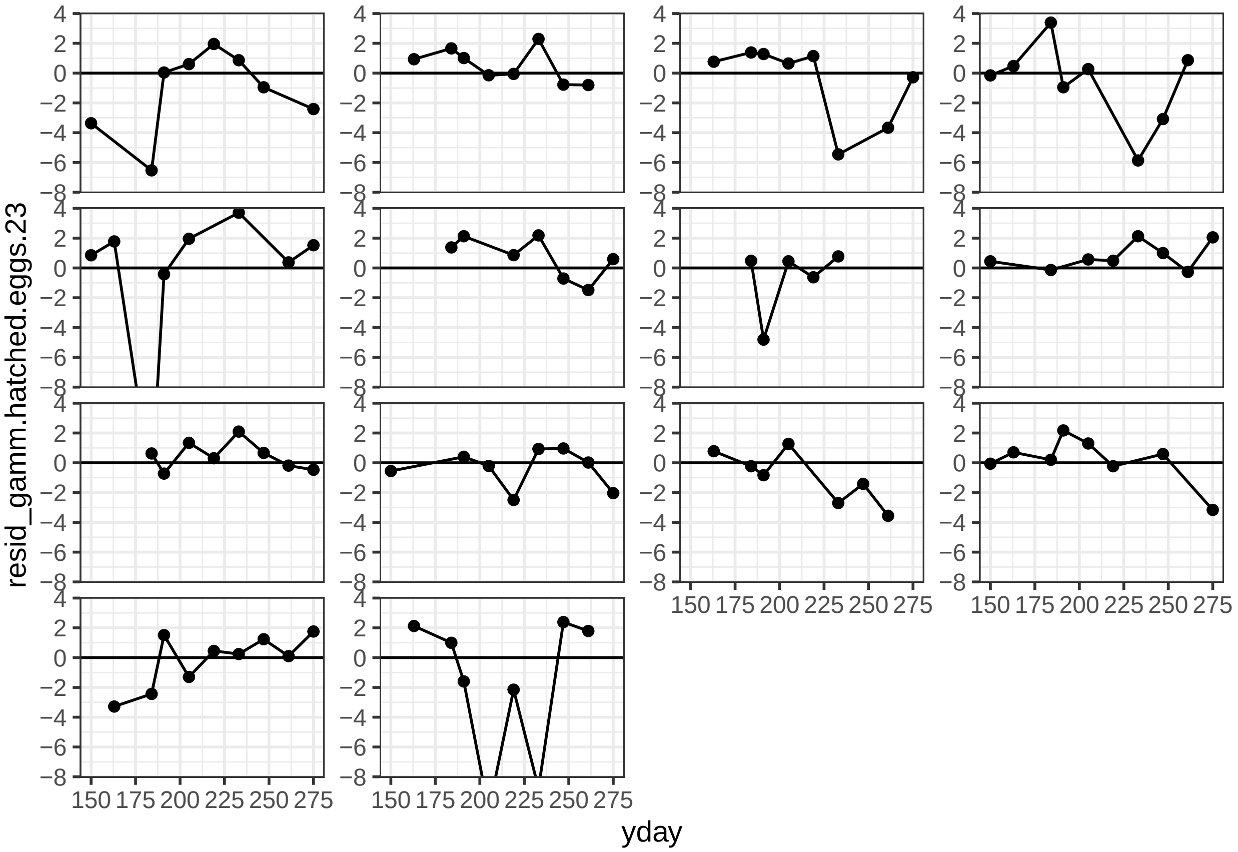

In both cases, there does not seem to be much structure left in the data.

If one were to do a formal test, there would probably be a temporal correlation. However, in this case we are more interested in modelling and understanding the effect rather than making a perfect inference, so the temporal correlation does not particularly affect our analysis.

Note that we set the limits at -7.5 and 3.5 to zoom in on the graphs, especially in the case of Caslano, where some residuals jump down to -15.

In general, there seems to be a larger variability in Caslano.

5.8 Comparing (over)dispersion in the two models

Two separate models are fitted for Morcote and Caslano. This is performed to formally check whether the overdispersion parameter is different for the two municipalities.

```
## Model for Morcote
gam.hatched.eggs.Morcote <- gamV(
  cbind(hatched, non.hatched) ~
    s(unique.ID, bs = "re"),
  family = "quasibinomial",
  data = filter(
    d.ovitraps.23.mod,
    municipality.fac == "Morcote")) ## new element!
summary(gam.hatched.eggs.Morcote)
```

```
Family: quasibinomial
```

```
Link function: logit

Formula:
cbind(hatched, non.hatched) ~ s(unique.ID, bs = "re")

Parametric coefficients:
            Estimate Std. Error t value Pr(>|t|)
(Intercept)  0.94131    0.06981   13.48   <2e-16 ***
---
Signif. codes:  0 '***' 0.001 '**' 0.01 '*' 0.05 '.' 0.1 ' ' 1

Approximate significance of smooth terms:
               edf Ref.df    F p-value
s(unique.ID) 17.08     44 0.77 0.00116 **
---
Signif. codes:  0 '***' 0.001 '**' 0.01 '*' 0.05 '.' 0.1 ' ' 1

R-sq.(adj) =  0.0822   Deviance explained = 12.6%
-REML = -20.385  Scale est. = 5.8085    n = 352
```

```
##
## Model for Caslano
gam.hatched.eggs.Caslano <- gamV(
  cbind(hatched, non.hatched) ~
    s(unique.ID, bs = "re"),
  family = "quasibinomial",
  data = filter(
    d.ovitraps.23.mod,
    municipality.fac == "Caslano")) ## new element!
summary(gam.hatched.eggs.Caslano)
```

```
Family: quasibinomial
Link function: logit

Formula:
cbind(hatched, non.hatched) ~ s(unique.ID, bs = "re")

Parametric coefficients:
            Estimate Std. Error t value Pr(>|t|)
(Intercept)   2.1307     0.1246    17.1   <2e-16 ***
---
Signif. codes:  0 '***' 0.001 '**' 0.01 '*' 0.05 '.' 0.1 ' ' 1

Approximate significance of smooth terms:
               edf Ref.df    F p-value
s(unique.ID) 4.326     13 0.53   0.106

R-sq.(adj) =  0.0332   Deviance explained = 9.92%
-REML = -38.291  Scale est. = 6.2506    n = 108
```

To compare overdispersion between the two models, we can extract the θ parameter, which controls for it. The higher the value of θ, the higher the overdispersion.

In fact, in the `gam()` model from the `{mgcv}` package, θ for quasi binomial models is described to be the

parameter such that

$$var(y) = \theta \times \mu(1-\mu), \quad \text{where} \quad \mu = \mathbb{E}(y)$$

.

Let's verify which model has highest θ, i.e., highest overdispersion.

```
summary(gam.hatched.eggs.Morcote)$dispersion
```

```
[1] 5.808461
```

```
summary(gam.hatched.eggs.Caslano)$dispersion
```

```
[1] 6.250632
```

Caslano has the highest overdispersion between the two municipalities.

6 Spatial Generalised Additive Model (spatial GAM)

We are now interested in testing whether geographical location plays a role in determining the hatching rate.

To achieve this, we first calculate the average hatching rates over the season.

This is already restricted to year 2023 as we had applied a filtering before.

```r
d.agg.hatch.proc <- d.ovitraps.23.hatch.proc.MC %>%
  group_by(unique.ID) %>%
  summarise(mean.hatched = mean(Perc.egg.hatch.per.trap,
                                na.rm = TRUE),
            X.num = first(X.num),
            Y.num = first(Y.num),
            municipality.fac = first(municipality.fac))
d.agg.hatch.proc %>%
  head(n = 10)
```

```
# A tibble: 10 x 5
   unique.ID   mean.hatched   X.num  Y.num municipality.fac
   <fct>              <dbl>   <dbl>  <dbl> <fct>
 1 Caslano.10a         68.9 711546. 92314. Caslano
 2 Morcote.10a         65.3 714371. 86745. Morcote
 3 Caslano.11a         92.6 711489. 92278. Caslano
 4 Morcote.11a         76.1 714192. 86860. Morcote
 5 Caslano.12a         83.0 711456. 92182. Caslano
 6 Morcote.12a         43.8 714119. 86945. Morcote
 7 Caslano.13a         82.4 711405. 92157. Caslano
 8 Morcote.13a         65.9 714100. 87024. Morcote
 9 Caslano.14a         84.2 711370. 92086. Caslano
10 Morcote.14a         66.7 714004. 87132. Morcote
```

6.1 Visualising the data

We visualise these rates over space for Morcote.

```r
## We calculate the min and max for the median number of eggs.
## This is used in the plots, in fact it allows us
## to use the same colour scale for the two
## municipalities and compare them more easily.
min.hatched.eggs <- min(d.agg.hatch.proc$mean.hatched)
max.hatched.eggs <- max(d.agg.hatch.proc$mean.hatched)
##
p <- ggplot(filter(d.agg.hatch.proc,
                   municipality.fac %in% c("Morcote")),
            mapping = aes(y = Y.num,
                          x = X.num,
                          colour = mean.hatched)) +
  geom_point(size = 3) +
  scale_color_gradientn(colours = c("blue", "purple", "red"),
                        values = scales::rescale(c(min.hatched.eggs,
                                                   max.hatched.eggs)),
                        limits = c(min.hatched.eggs, max.hatched.eggs)) +
  # theme(aspect.ratio = 1) +
  coord_fixed() +
```

```
  labs(title = "Morcote")
p
```

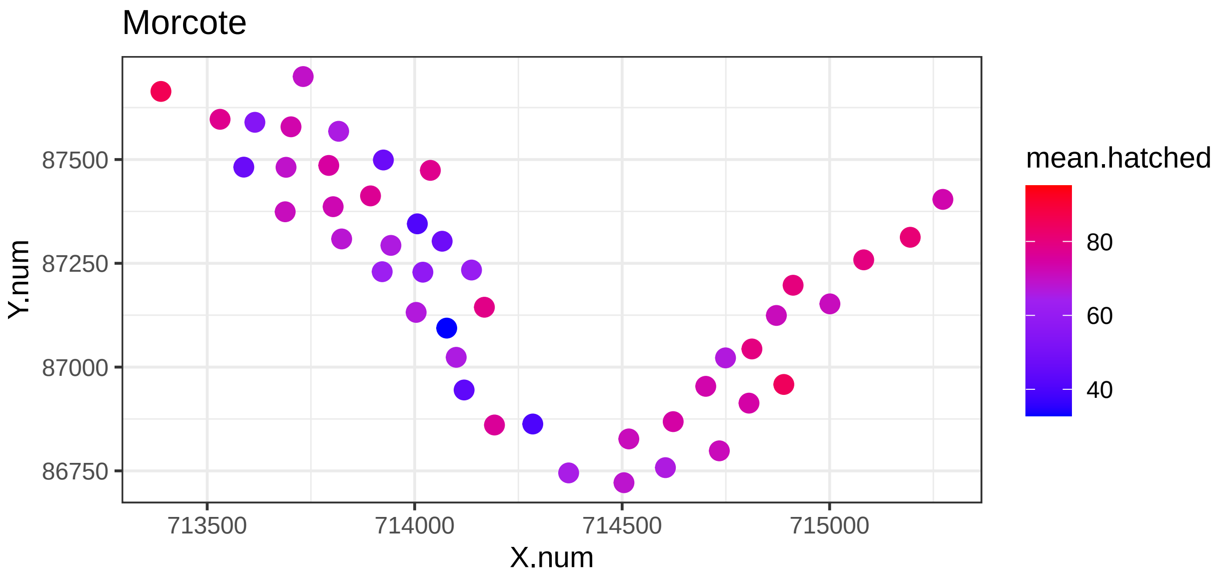


```
## save plot for future use:
saveRDS(p, file = file.path("saved_figures",
                            "2b_HathchingRatesOverSpaceMorcote_graphForPaper.rds"))
```

We can observe that there is a strong geographical pattern between the average number of eggs hatched in Morcote; in fact, the right-hand side has a higher average and less variability than the left-hand side.

We also plot the release points:

```
d.release.points.morcote <-
  read_excel("../Original_data/Coordinate_Release_points.xlsx")
head(d.release.points.morcote)
```

```
# A tibble: 6 x 4
  Municipality Release_point LV03E      LV03N
  <chr>                <dbl> <chr>      <chr>
1 Morcote                  1 715'380.43 87'541.29
2 Morcote                  2 715'324.64 87'466.31
3 Morcote                  3 715'265.00 87'393.70
4 Morcote                  4 715'208.52 87'319.81
5 Morcote                  5 715'129.26 87'268.15
6 Morcote                  6 715'059.20 87'201.06
```

```
str(d.release.points.morcote)
```

```
tibble [75 x 4] (S3: tbl_df/tbl/data.frame)
 $ Municipality : chr [1:75] "Morcote" "Morcote" "Morcote" "Morcote" ...
 $ Release_point: num [1:75] 1 2 3 4 5 6 7 8 9 10 ...
 $ LV03E        : chr [1:75] "715'380.43" "715'324.64" "715'265.00" "715'208.52" ...
 $ LV03N        : chr [1:75] "87'541.29" "87'466.31" "87'393.70" "87'319.81" ...
```

```
d.release.points.morcote <- d.release.points.morcote %>%
  mutate(LV03_E = as.numeric(gsub("'", "", LV03E)),
         LV03_N = as.numeric(gsub("'", "", LV03N)))
str(d.release.points.morcote)
```

```
tibble [75 x 6] (S3: tbl_df/tbl/data.frame)
 $ Municipality : chr [1:75] "Morcote" "Morcote" "Morcote" "Morcote" ...
 $ Release_point: num [1:75] 1 2 3 4 5 6 7 8 9 10 ...
 $ LV03E        : chr [1:75] "715'380.43" "715'324.64" "715'265.00" "715'208.52" ...
 $ LV03N        : chr [1:75] "87'541.29" "87'466.31" "87'393.70" "87'319.81" ...
 $ LV03_E       : num [1:75] 715380 715325 715265 715209 715129 ...
 $ LV03_N       : num [1:75] 87541 87466 87394 87320 87268 ...
```

```r
## Plot Morcote
p <- ggplot(filter(d.agg.hatch.proc,
                   municipality.fac %in% c("Morcote")),
            mapping = aes(y = Y.num,
                          x = X.num,
                          colour = mean.hatched)) +
  geom_point(size = 3) +
  scale_color_gradientn(colours = c("blue", "purple", "red"),
                        values = scales::rescale(c(min.hatched.eggs,
                                                   max.hatched.eggs)),
                        limits = c(min.hatched.eggs, max.hatched.eggs)) +
  geom_point(data = d.release.points.morcote,
             mapping = aes(x = LV03_E, y = LV03_N),
             colour = "black", size = 1, pch = 4) +
  coord_fixed()

p
```

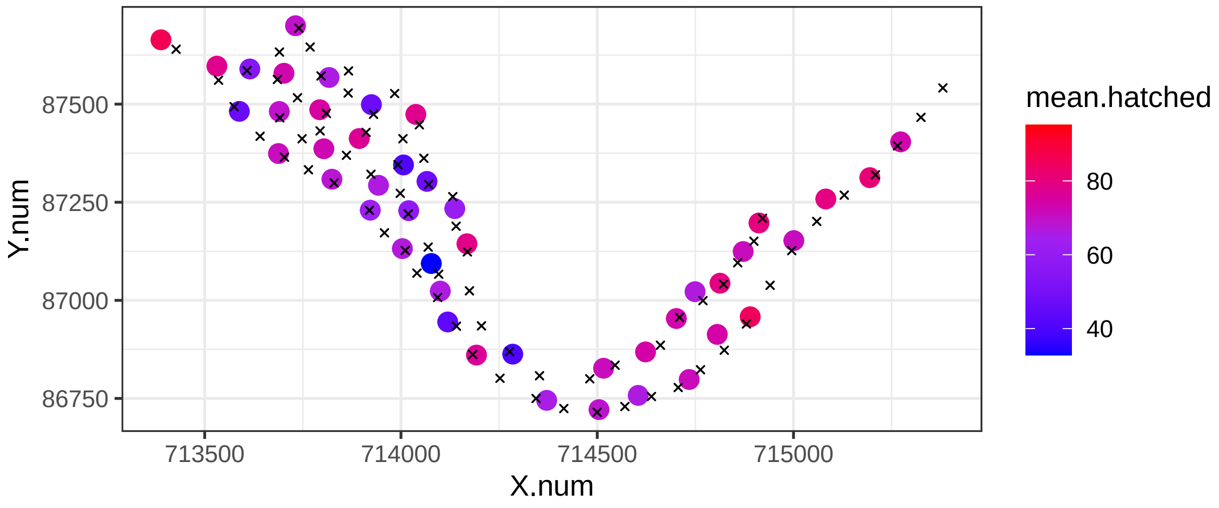


```r
## save plot for future use:
saveRDS(p, file = file.path("saved_figures",
                            "2b_HathchingRatesOverSpaceMorcote_graphForPaper_releasePoints.rds"))
```

Let's look at Caslano.

```r
p <- ggplot(filter(d.agg.hatch.proc, municipality.fac == "Caslano"),
            mapping = aes(y = Y.num,
                          x = X.num,
                          colour = mean.hatched)) +
  geom_point(size = 3) +
  scale_color_gradientn(colours = c("blue", "purple", "red"),
                        values = scales::rescale(c(min.hatched.eggs,
```

```r
                                                    max.hatched.eggs)),
                               limits = c(min.hatched.eggs, max.hatched.eggs)) +
  # theme(aspect.ratio = 1) +
  coord_fixed() +
  labs(title = "Caslano")
p
```

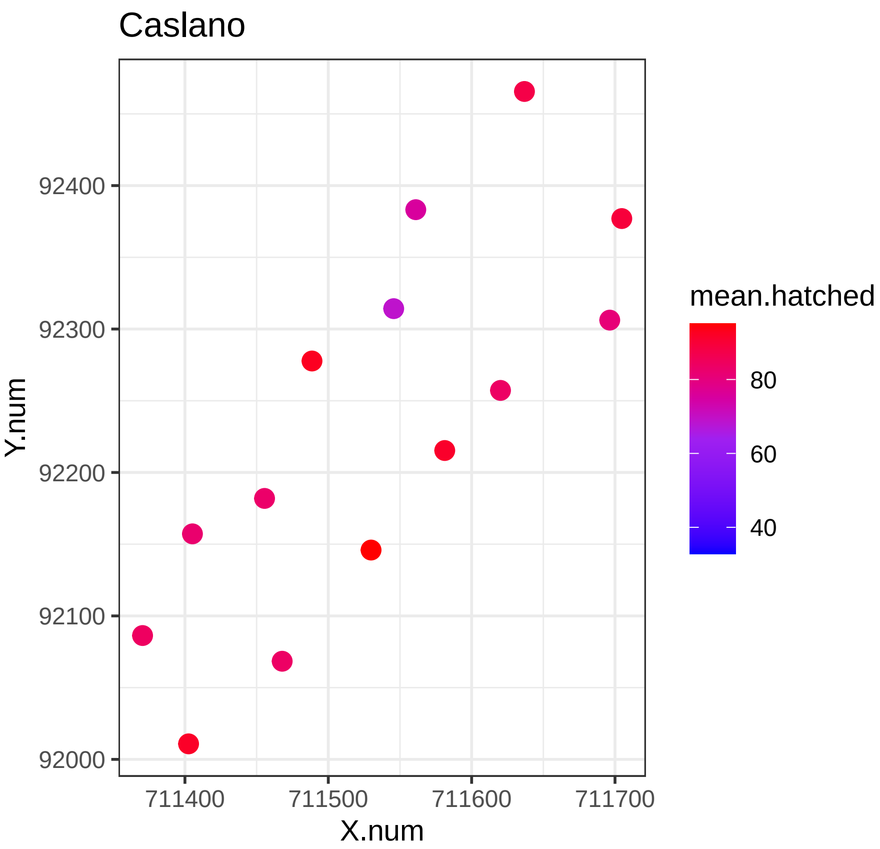


```r
## save plot for future use:
saveRDS(p, file = file.path("saved_figures",
                            "2b_HathchingRatesOverSpaceCaslano_graphForPaper.rds"))
```

In Caslano there is almost no variability in the average number of eggs hatched, and this average is very high.

6.2 Fitting the models

We aim to model the percentage of hatched *Aedes albopictus* eggs over the season (*yday*), while accounting for geographical differences.

The response variable is binomial, representing the counts of hatched versus non-hatched eggs (quantified by the variables *hatched.albo.eggs.after.proc* and *non.hatched.albo.eggs.after.proc*). To account for overdispersion, the quasi-binomial family is used for formal modelling.

yday will be included as numeric variable; the same holds true for *X.num* and *Y.num*, which represent the swiss coordinates.

We begin without assuming any specific seasonal pattern, so we will fit a Generalised Additive Model (GAM), which provides flexibility in determining the best shape for the seasonal trend. However, this flexibility comes at the cost of interpretability.

Note that we use the element `s(X.num, Y.num)` to plot spatial data. We can use this isotropic smoothing because both predictors are spatial coordinates and we assume the spatial effect is istropic (i.e., the same in all directions) and the two variables are on the same scale.

We start by fitting the model for Morcote and Vico Morcote.

```
d.ovitraps.23.morcote <- filter(
  d.ovitraps.23.mod,
  municipality.fac %in% c("Morcote"))
##
## GAM for Morcote and Vico Morcote
gam.hatch_space.Morcote <- gamV(
  cbind(hatched, non.hatched) ~
    s(yday, pc = pc.23) +
    s(X.num, Y.num), ## new element!
  family = "quasibinomial",
  data = d.ovitraps.23.morcote)
##
## save the model for future use
saveRDS(gam.hatch_space.Morcote,
        file = "Prepared_data_and_models/gam.hatch_space.Morcote.23.rds")
```

```
summary(gam.hatch_space.Morcote)
```

```


Family: quasibinomial
Link function: logit

Formula:
cbind(hatched, non.hatched) ~ s(yday, pc = pc.23) + s(X.num,
    Y.num)

Parametric coefficients:
            Estimate Std. Error t value Pr(>|t|)
(Intercept)  0.95613    0.08715   10.97   <2e-16 ***
---
Signif. codes:  0 '***' 0.001 '**' 0.01 '*' 0.05 '.' 0.1 ' ' 1

Approximate significance of smooth terms:
                  edf Ref.df     F p-value
s(yday)         3.256  4.032 3.607 0.00664 **
s(X.num,Y.num) 5.409  7.407 3.195 0.00228 **
---
Signif. codes:  0 '***' 0.001 '**' 0.01 '*' 0.05 '.' 0.1 ' ' 1

R-sq.(adj) =  0.0858   Deviance explained = 10.8%
-REML = -24.196  Scale est. = 5.9487    n = 352
```

We refit the same model for Caslano. Let's see how large k can be for the bi-dimensional smoother.

```
d.ovitraps.23.mod %>%
  filter(municipality.fac == "Caslano") %>%
  select(X.num, Y.num) %>%
  unique() %>%
  nrow()
```

```
[1] 14
```

```
d.ovitraps.23.caslano <- filter(d.ovitraps.23.mod,
                                municipality.fac == "Caslano")
gam.hatch_space.Caslano <- gamV(
  cbind(hatched, non.hatched) ~
    s(yday, pc = pc.23) +
    s(X.num, Y.num, k = 14), ## new element!
  family = "quasibinomial",
  data = d.ovitraps.23.caslano)
##
## save the model for future use
saveRDS(gam.hatch_space.Caslano,
        file = "Prepared_data_and_models/gam.hatch_space.Caslano.23.rds")
```

```
summary(gam.hatch_space.Caslano)
```

```

Family: quasibinomial
Link function: logit

Formula:
cbind(hatched, non.hatched) ~ s(yday, pc = pc.23) + s(X.num,
    Y.num, k = 14)

Parametric coefficients:
            Estimate Std. Error t value Pr(>|t|)
(Intercept)    2.143      0.198   10.82   <2e-16 ***
---
Signif. codes:  0 '***' 0.001 '**' 0.01 '*' 0.05 '.' 0.1 ' ' 1

Approximate significance of smooth terms:
                 edf Ref.df     F p-value
s(yday)        2.855  3.549 3.941 0.00697 **
s(X.num,Y.num) 2.000  2.000 1.628 0.20144
---
Signif. codes:  0 '***' 0.001 '**' 0.01 '*' 0.05 '.' 0.1 ' ' 1

R-sq.(adj) =  0.0797   Deviance explained = 16.1%
-REML = -40.867  Scale est. = 6.244     n = 108
```

6.3 Plotting the model

We now plot the results.

6.3.1 Morcote and Vico Morcote

We start with visualising the model fitted for Morcote.

```
plot.gam(gam.hatch_space.Morcote,
         select = 1,
         trans = plogis,
         rug = TRUE)
```

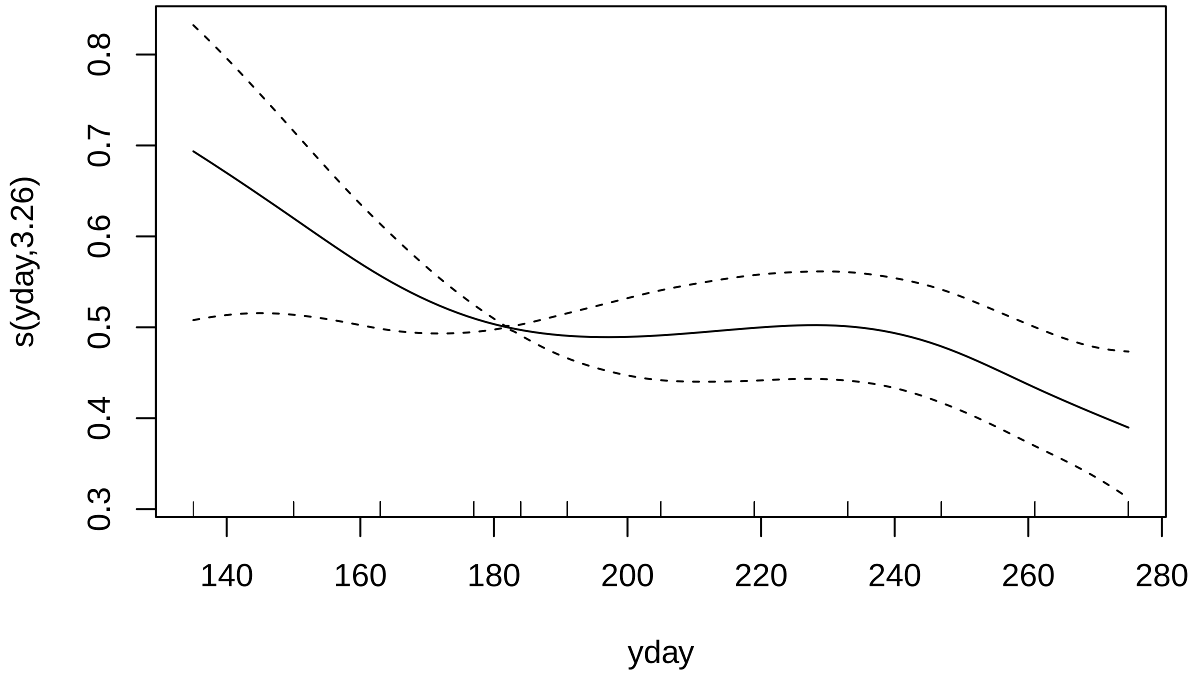


```r
##
plot.gamViz(gam.hatch_space.Morcote,
            select = 2,
            trans = plogis,
            rug = TRUE) +
  coord_fixed()
```

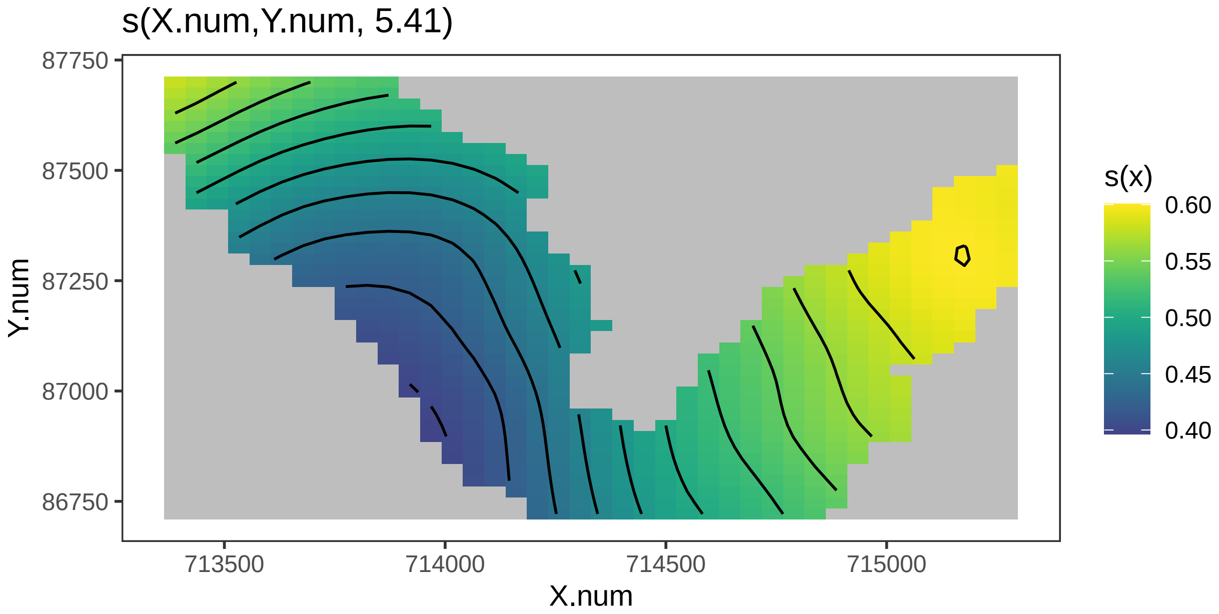

The percentage of hatched eggs is higher on the right-hand side of Morcote.

6.3.2 Caslano

We now visualise the model for Caslano.

```r
plot.gam(gam.hatch_space.Caslano,
         select = 1,
         trans = plogis)
```

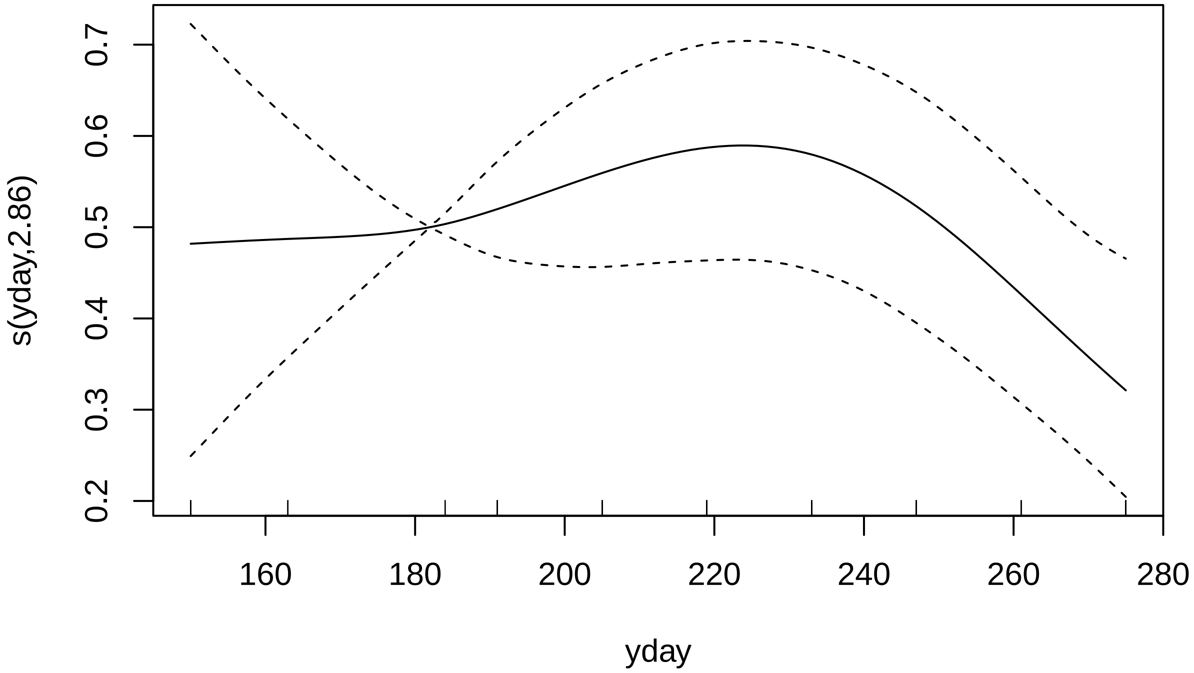


```r
##
plot.gamViz(gam.hatch_space.Caslano,
            select = 2,
            trans = plogis) +
  coord_fixed()
```

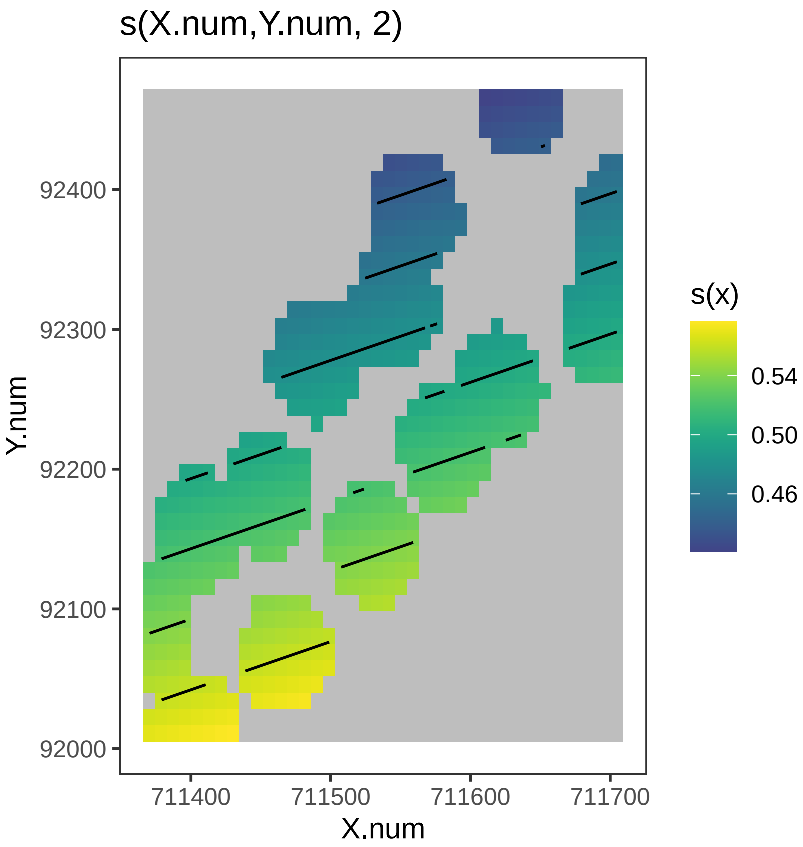

Apparently, the density is not high enough to estimate the entire surface.

Note also that the scale shows that the spatial variation is less than in Morcote.

6.4 Residual analysis

First of all, we apply the `gam.check()` function to the models, which produces some diagnostic information.

```
## Morcote
par(mfrow = c(2, 2))
gam.check(gam.hatch_space.Morcote)
```

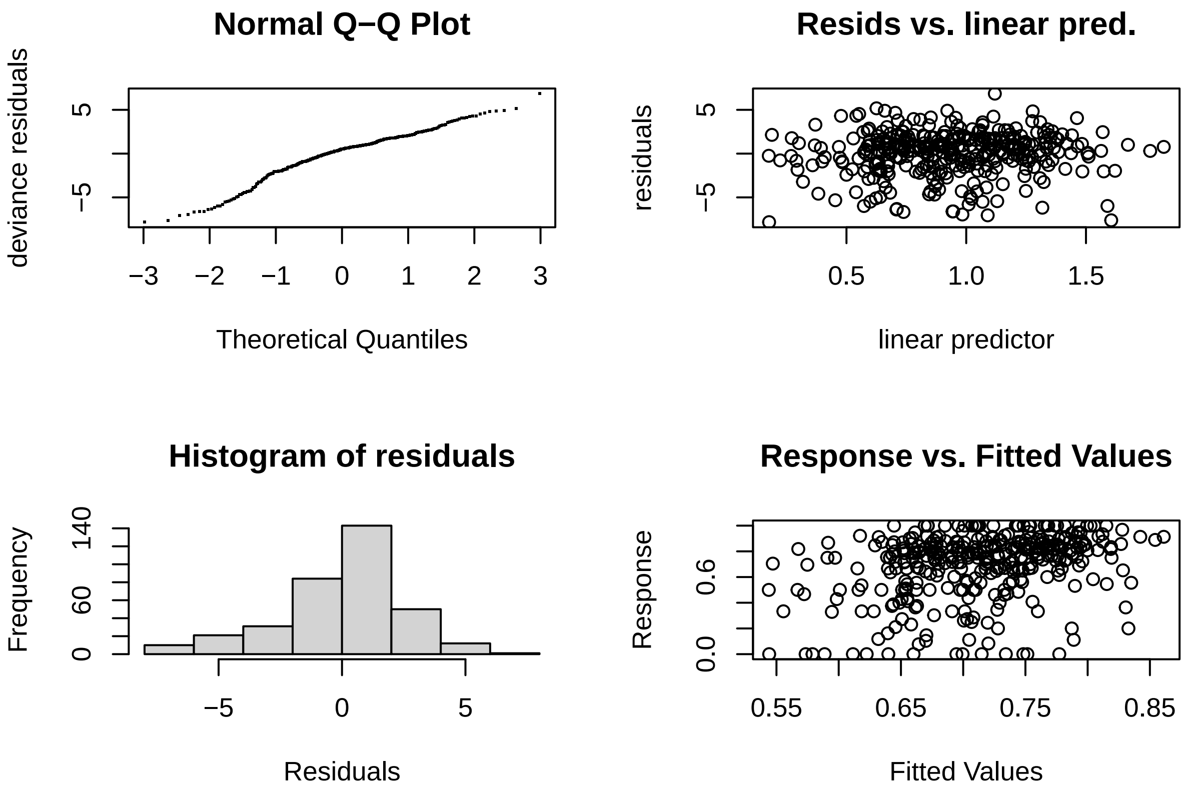


```
Method: REML   Optimizer: outer newton
full convergence after 10 iterations.
Gradient range [-2.199997e-09,-2.007994e-11]
(score -24.19594 & scale 5.948702).
Hessian positive definite, eigenvalue range [0.5425082,174.0236].
Model rank =  39 / 39

Basis dimension (k) checking results. Low p-value (k-index<1) may
indicate that k is too low, especially if edf is close to k'.

                  k'   edf k-index p-value
s(yday)         9.00  3.26    1.06    0.83
s(X.num,Y.num) 29.00  5.41    1.05    0.82
```

```
par(mfrow = c(1, 1))
##
## Caslano
par(mfrow = c(2, 2))
gam.check(gam.hatch_space.Caslano)
```

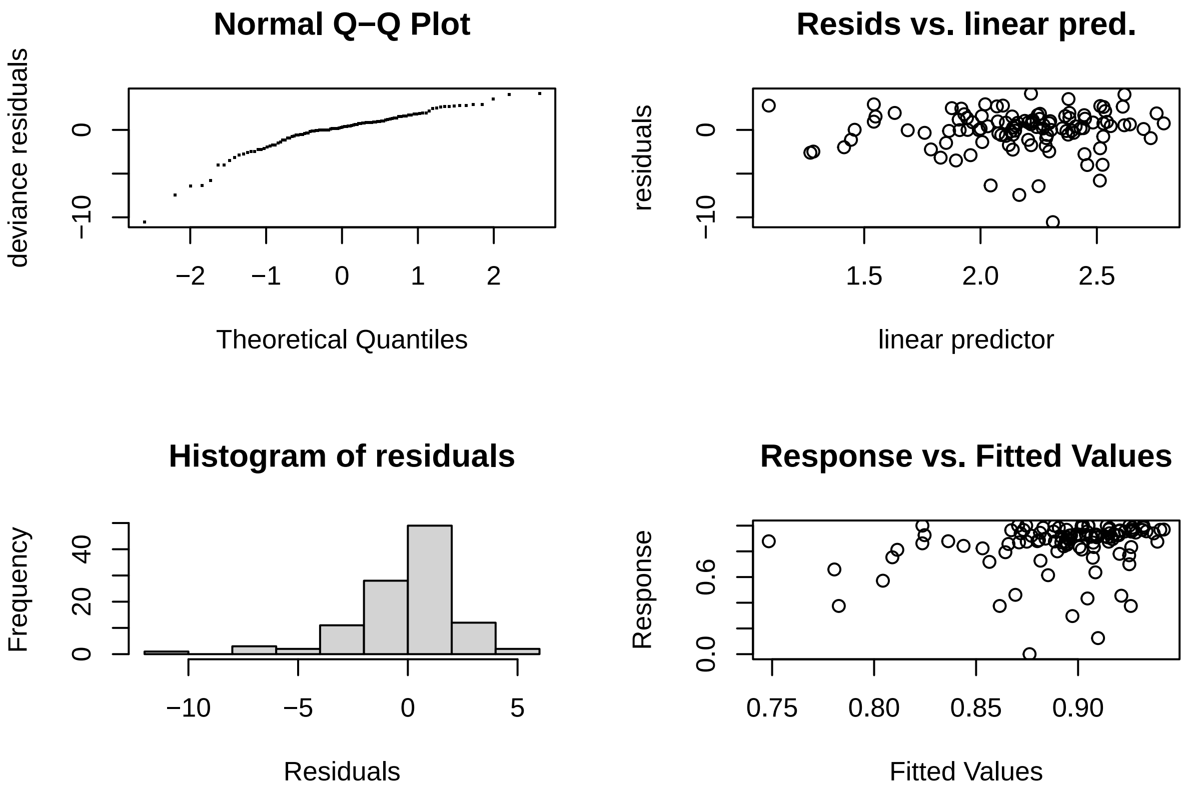


```
Method: REML   Optimizer: outer newton
full convergence after 12 iterations.
Gradient range [-1.790304e-05,2.356962e-05]
(score -40.86731 & scale 6.243991).
Hessian positive definite, eigenvalue range [1.790201e-05,52.01665].
Model rank =  23 / 23

Basis dimension (k) checking results. Low p-value (k-index<1) may
indicate that k is too low, especially if edf is close to k'.

                  k'   edf k-index p-value
s(yday)         9.00  2.86    1.22    0.99
s(X.num,Y.num) 13.00  2.00    1.16    0.92
```

```
par(mfrow = c(1, 1))
```

Then, we store the pearson residuals in the original data frame, and we plot the residuals against the fitted values to see whether there is still structure in the data.

```
## Morcote
d.ovitraps.23.morcote$resid_gam.hatch_space.Morcote <-
  resid(gam.hatch_space.Morcote,
        type = "pearson")
##
d.ovitraps.23.morcote$fitted_gam.hatch_space.Morcote <-
  fitted(gam.hatch_space.Morcote)
##
ggplot(data = d.ovitraps.23.morcote,
       mapping = aes(y = resid_gam.hatch_space.Morcote,
```

```r
                   x = fitted_gam.hatch_space.Morcote)) +
  geom_hline(yintercept = 0) +
  geom_point(alpha = 0.2) +
  geom_smooth()
```

```
`geom_smooth()` using method = 'loess' and formula = 'y ~ x'
```

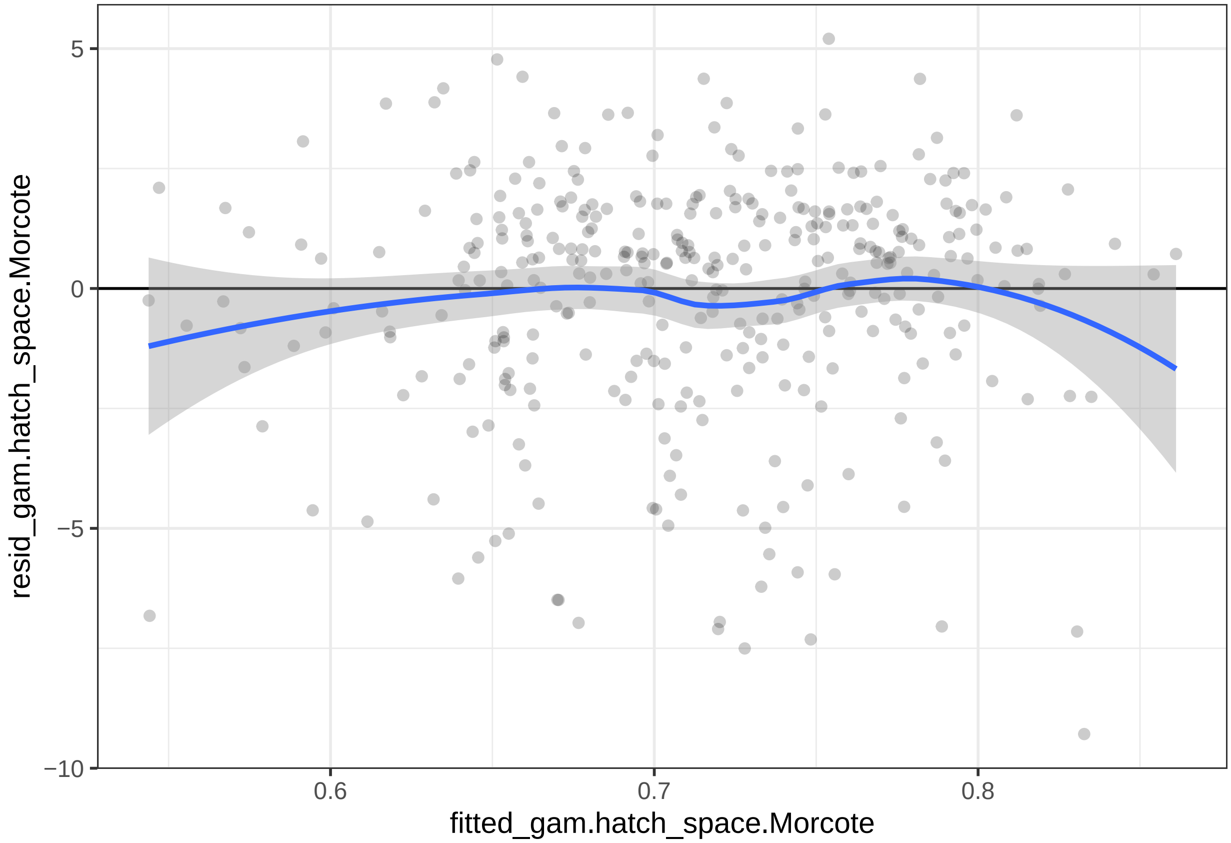


```r
##
## Caslano
d.ovitraps.23.caslano$resid_gam.hatch_space.Caslano <- resid(gam.hatch_space.Caslano,
                                                             type = "pearson")
##
##
d.ovitraps.23.caslano$fitted_gam.hatch_space.Caslano <- fitted(gam.hatch_space.Caslano)
##
ggplot(data = d.ovitraps.23.caslano,
       mapping = aes(y = resid_gam.hatch_space.Caslano,
                     x = fitted_gam.hatch_space.Caslano)) +
  geom_hline(yintercept = 0) +
  geom_point(alpha = 0.2) +
  geom_smooth()
```

```
`geom_smooth()` using method = 'loess' and formula = 'y ~ x'
```

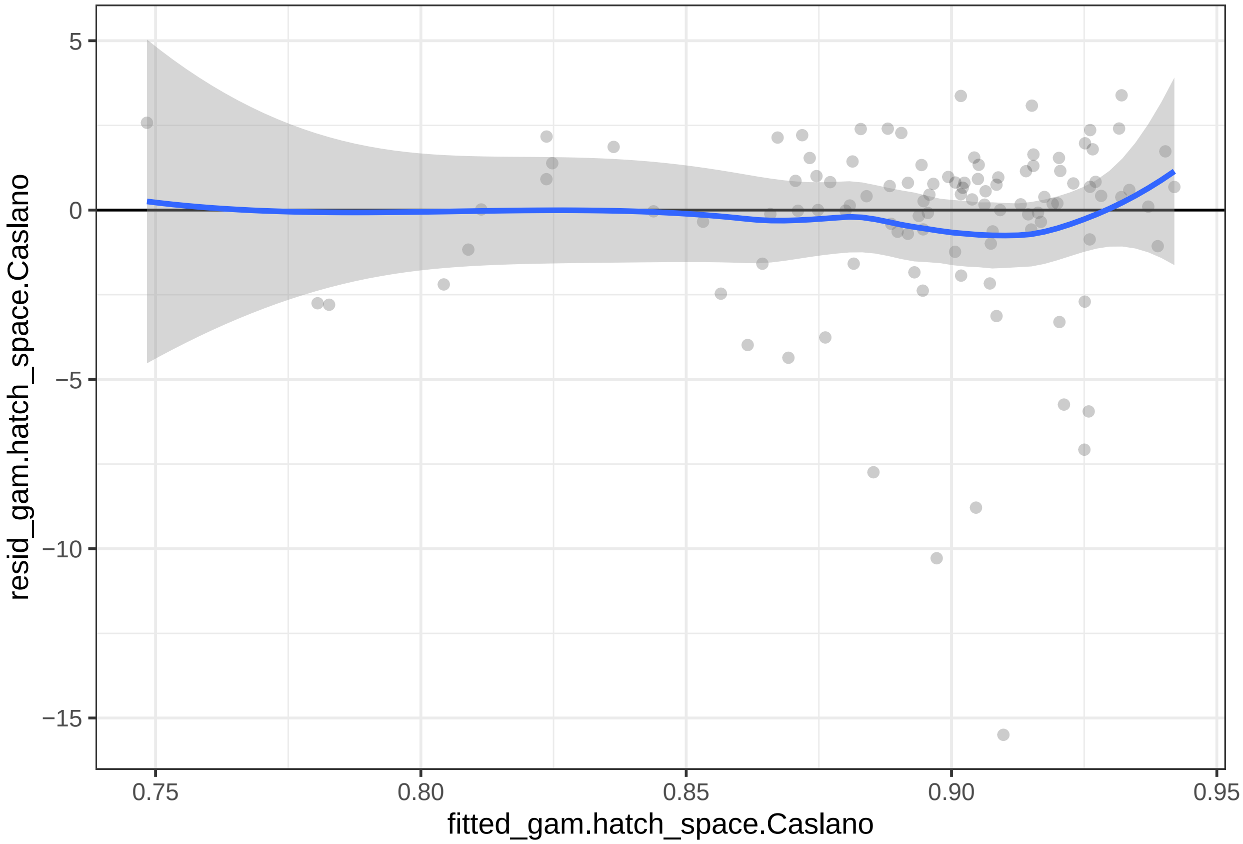

The blue lines are located on the x-axis, indicating that there doesn't seem to be structure left in the residuals.

6.5 Comparing (over)dispersion in the two models

We compare again the overdispersion in the two models.

As a reminder, in a quasi binomial model the parameter θ is estimated to be such that:

$$var(y) = \theta \times \mu(1-\mu), \quad \text{where} \quad \mu = \mathbb{E}(y)$$

.

Let's verify which model has highest θ, i.e., highest overdispersion.

```
summary(gam.hatch_space.Morcote)$dispersion
```

```
[1] 5.948702
```

```
summary(gam.hatch_space.Caslano)$dispersion
```

```
[1] 6.243991
```

Caslano has the highest overdispersion between the two municipalities; however the difference is tiny.

7 Methods

7.1 Generalised Additive Mixed-Effects Model (GAMM)

We modelled the percentage of hatched *Aedes albopictus* eggs (combining the variables *hatched.albo.eggs.after.proc* and *non.hatched.albo.eggs.after.proc* to a binomial response variable)), using a Generalized Additive Model (GAM) with a quasi binomial family to address overdispersion. The model included a smooth effect for "sampling date", represented as the day of the year (*yday*, a numeric variable ranging from 135 to 275), which interacts with municipality (*municipality.fac*). *municipality.fac* is a categorical variable, consisting of 2 levels: Caslano, Morcote ; its effect was modelled as a fixed effect.

We controlled for the non-independence of observations by including *trap ID* (*unique.ID*, a categorical variable) as a random effect, with data from 59 distinct traps.
Model complexity was evaluated, and the best-fitting model was selected using a Chi-square test and AIC and BIC criteria. All statistical analyses were conducted using R.
The significance level was set at 5%.

7.2 Spatial Generalised Additive Model (spatial GAM)

We fitted two separate models to analyse the percentage of hatched *Aedes albopictus* eggs (combining the variables *hatched.albo.eggs.after.proc* and *non.hatched.albo.eggs.after.proc* to a binomial response variable)), using a Generalized Additive Model (GAM) with a negative binomial family to account for overdispersion. One model was fitted for the municipalities of Morcote and Vico Morcote, and the other for Caslano.

Both models included a smooth effect for the "sampling date", represented as day of the year (*yday*), and a combined smooth effect for geographic coordinates to capture spatial variability within each municipality. The significance level was set at 5%.

We selected the best-fitting models by evaluating AIC and BIC criteria.
All statistical analyses were conducted using R. The significance level was set at 5%.

8 Results

8.1 Generalised Additive Mixed-Effects Model (GAMM)

model converged without problems, deviance explained is

35 %

Allowing for different shapes for the municipalities was verified to be necessary (p-value 0.2312048).

- Morcote has systematic lower hatched eggs compared to Caslano.
- Percentage of hatched eggs in Vico Morcote is in the middle of the two other municipalities

In our analysis, we observed a lower variability in the number of Aedes albopictus in Morcote (overdispersion parameter: 5.808461) compared to Calsano (overdispersion parameter: 6.2506323).

8.2 Spatial Generalised Additive Model (spatial GAM)

gam.hatch_space.Morcote

- morcote model: deviance explained is

10.8 %

- caslano model: deviance explained is

16.1 %

In our analysis, we observed a lower variability in the number of Aedes albopictus in Morcote (overdispersion parameter: 5.9487024) compared to Calsano (overdispersion parameter: 6.2439906).

9 Conclusions

Morcote consistently exhibited a lower percentage of hatched eggs compared to Caslano.

In the spatial analysis, there was an important variability in the percentage of hatched eggs within Morcote. In fact, a difference emerged between the left and right sides, with the right side showing a higher percentage of hatched eggs. In contrast, the left side had lower counts.

One potential factor influencing this disparity could be Vico Morcote, which was included in the modelling for Morcote and was untreated. In fact, it may be contributing to the higher percentage of hatcheg eggs in Morcote. However, this remains a hypothesis, and further analysis is needed to confirm it.

Caslano, by comparison, exhibited minimal geographical variation.

10 References

```r
citation("mgcViz")
```

```
To cite the mgcViz package in publications use:

  Fasiolo, M., Nedellec, R., Goude, Y. and Wood, S.N., 2020. Scalable
  visualization methods for modern generalized additive models. Journal
  of computational and Graphical Statistics, 29(1), pp.78-86.

A BibTeX entry for LaTeX users is

  @Article{,
    title = {Scalable visualisation methods for modern Generalized Additive Models.},
    journal = {Journal of the Royal Statistical Society (B)},
    volume = {29},
    number = {1},
    pages = {78-86},
    year = {2020},
    author = {{Fasiolo} and {Matteo} and {Nedellec} and {Rapha{"e}l} and {Goude} and {Yannig} and {Wood
  }

As mgcViz is often updated, you may want to cite its version number.
Find it with 'help(package=mgcViz)'.
```

```r
citation("lubridate")
```

```
To cite lubridate in publications use:

  Garrett Grolemund, Hadley Wickham (2011). Dates and Times Made Easy
  with lubridate. Journal of Statistical Software, 40(3), 1-25. URL
  https://www.jstatsoft.org/v40/i03/.

A BibTeX entry for LaTeX users is

  @Article{,
    title = {Dates and Times Made Easy with {lubridate}},
    author = {Garrett Grolemund and Hadley Wickham},
    journal = {Journal of Statistical Software},
    year = {2011},
    volume = {40},
    number = {3},
```

```
    pages = {1--25},
    url = {https://www.jstatsoft.org/v40/i03/},
  }
```


11 Appendix

11.1 Hatched eggs over discrete time

```
d.ovitraps.23.mod <- d.ovitraps.23.mod %>%
  mutate(date.municipality = interaction(Sampling_date.date, municipality.fac,
                                          drop = TRUE))
##
set.seed(6)
d.ovitraps.23.mod %>%
  sample_n(size = 5) %>%
  select(municipality.fac, Sampling_date.date, date.municipality)
```

```
# A tibble: 5 x 3
  municipality.fac Sampling_date.date date.municipality
  <fct>            <date>             <fct>
1 Morcote          2023-08-21         2023-08-21.Morcote
2 Morcote          2023-05-30         2023-05-30.Morcote
3 Morcote          2023-10-02         2023-10-02.Morcote
4 Morcote          2023-09-04         2023-09-04.Morcote
5 Caslano          2023-07-10         2023-07-10.Caslano
```

```
##
glmm.hatched.eggs.discrete <- glmmTMB(
  cbind(hatched, non.hatched) ~ date.municipality +
    # factor(Sampling_date.date) *
    # municipality.fac +
    (1 | unique.ID),
  family = "betabinomial",
  data = d.ovitraps.23.mod)
##
summary(glmm.hatched.eggs.discrete)
```

```
 Family: betabinomial  ( logit )
Formula:
cbind(hatched, non.hatched) ~ date.municipality + (1 | unique.ID)
Data: d.ovitraps.23.mod

     AIC      BIC   logLik deviance df.resid
  2669.6   2768.7  -1310.8   2621.6      436

Random effects:

Conditional model:
 Groups    Name        Variance Std.Dev.
 unique.ID (Intercept) 0.02976  0.1725
Number of obs: 460, groups:  unique.ID, 59

Dispersion parameter for betabinomial family (): 5.55
```

```
Conditional model:
                                     Estimate Std. Error z value Pr(>|z|)
(Intercept)                            1.3792     0.5575   2.474   0.0134 *
date.municipality2023-06-12.Caslano    0.5198     0.6695   0.776   0.4375
date.municipality2023-07-03.Caslano    0.2615     0.6261   0.418   0.6762
date.municipality2023-07-10.Caslano    0.3942     0.6259   0.630   0.5288
date.municipality2023-07-24.Caslano    0.5895     0.6354   0.928   0.3535
date.municipality2023-08-07.Caslano    0.5121     0.6370   0.804   0.4214
date.municipality2023-08-21.Caslano    0.3890     0.6161   0.631   0.5278
date.municipality2023-09-04.Caslano    0.4071     0.6379   0.638   0.5234
date.municipality2023-09-18.Caslano   -0.1330     0.6274  -0.212   0.8322
date.municipality2023-10-02.Caslano   -0.2193     0.6393  -0.343   0.7316
date.municipality2023-05-15.Morcote   15.5900  2799.4166   0.006   0.9956
date.municipality2023-05-30.Morcote    0.2356     0.6178   0.381   0.7030
date.municipality2023-06-12.Morcote   -0.5791     0.5864  -0.987   0.3234
date.municipality2023-06-26.Morcote   -0.2957     0.5892  -0.502   0.6157
date.municipality2023-07-03.Morcote   -0.5517     0.5843  -0.944   0.3450
date.municipality2023-07-10.Morcote   -0.5883     0.5832  -1.009   0.3131
date.municipality2023-07-24.Morcote   -0.5254     0.5791  -0.907   0.3643
date.municipality2023-08-07.Morcote   -0.6718     0.5810  -1.156   0.2476
date.municipality2023-08-21.Morcote   -0.3859     0.5763  -0.670   0.5031
date.municipality2023-09-04.Morcote   -0.6297     0.5774  -1.090   0.2755
date.municipality2023-09-18.Morcote   -0.8868     0.5849  -1.516   0.1295
date.municipality2023-10-02.Morcote   -1.0599     0.5855  -1.810   0.0703 .
---
Signif. codes:  0 '***' 0.001 '**' 0.01 '*' 0.05 '.' 0.1 ' ' 1
```

The estimates are based on few observations and thus are not so precise.

```
ranef(glmm.hatched.eggs.discrete)
```

```
$unique.ID
              (Intercept)
Caslano.10a -0.1028057013
Morcote.10a -0.0211355470
Caslano.11a  0.0411657251
Morcote.11a  0.0001755765
Caslano.12a -0.0468057576
Morcote.12a -0.1890579035
Caslano.13a -0.0145860142
Morcote.13a -0.0328436152
Caslano.14a  0.0354807243
Morcote.14a  0.0085356356
Morcote.15a -0.0508931792
Morcote.16a -0.0251811855
Morcote.17a -0.0107700656
Morcote.18a  0.0241694158
Morcote.19a -0.0650944540
Caslano.1a   0.0864158179
Morcote.1a   0.0207477044
Morcote.20a -0.0347524438
Morcote.21a  0.0511495888
Morcote.22a  0.1587062814
Morcote.23a -0.0858661703
Morcote.24a  0.0093004009
```

```
Morcote.25a  0.0321436285
Morcote.26a -0.0579237910
Morcote.27a -0.0974931420
Morcote.28a  0.0169399896
Morcote.29a  0.0607032217
Caslano.2a  -0.0302132674
Morcote.2a   0.1292811732
Morcote.30a -0.0444301987
Morcote.31a -0.1855587600
Morcote.32a -0.1343624518
Morcote.33a -0.0461215800
Morcote.34a -0.1655486679
Morcote.35a  0.0731192034
Morcote.36a  0.0082688572
Morcote.37a  0.1098482880
Morcote.38a -0.0726114845
Morcote.39a  0.1194324907
Caslano.3a   0.0965232992
Morcote.3a   0.0978253736
Morcote.40a  0.0170472700
Morcote.41a  0.0875450925
Morcote.42a -0.0437516379
Morcote.43a  0.1011778180
Morcote.44a  0.0465169883
Morcote.45a -0.0267905233
Caslano.4a   0.0382024200
Morcote.4a  -0.0098088030
Caslano.5a  -0.0207976679
Morcote.5a   0.1363866633
Caslano.6a  -0.0549943515
Morcote.6a   0.0481378278
Caslano.7a   0.0238963904
Morcote.7a   0.0215769113
Caslano.8a  -0.0092955893
Morcote.8a  -0.0390259718
Caslano.9a  -0.0666703608
Morcote.9a   0.0088186148
```

```r
d.RE_glmm.hatched.eggs <- ranef(glmm.hatched.eggs.discrete) %>%
  as.data.frame() %>%
  select(grp, condval) %>%
  rename("unique.ID" = "grp")
##
head(d.RE_glmm.hatched.eggs)
```

```
         unique.ID       condval
cond.1 Caslano.10a -0.1028057013
cond.2 Morcote.10a -0.0211355470
cond.3 Caslano.11a  0.0411657251
cond.4 Morcote.11a  0.0001755765
cond.5 Caslano.12a -0.0468057576
cond.6 Morcote.12a -0.1890579035
```

```r
str(d.RE_glmm.hatched.eggs)
```

```
'data.frame':	59 obs. of  2 variables:
 $ unique.ID: Factor w/ 59 levels "Morcote.12a",..: 5 24 44 30 14 1 26 20 42 32 ...
 $ condval  : num  -0.102806 -0.021136 0.041166 0.000176 -0.046806 ...
```

```r
##
d.ovitrap.XY <- d.ovitraps.23.hatch.proc.MC %>%
  select(municipality.fac, unique.ID, X.num, Y.num) %>%
  unique()
d.RE.XY <- d.ovitraps.23.hatch.proc.MC %>%
  left_join(d.RE_glmm.hatched.eggs,
            d.ovitrap.XY,
            by = join_by("unique.ID"))
```

```r
ggplot(filter(d.RE.XY,
              municipality.fac %in% c("Morcote", "Vico Morcote")),
       mapping = aes(y = Y.num,
                     x = X.num,
                     colour = condval)) +
  geom_point(size = 3) +
  scale_color_gradientn(colours = c("blue", "purple", "red"),
                        values = c(0, 0.5, 1)) +
  coord_fixed()
```

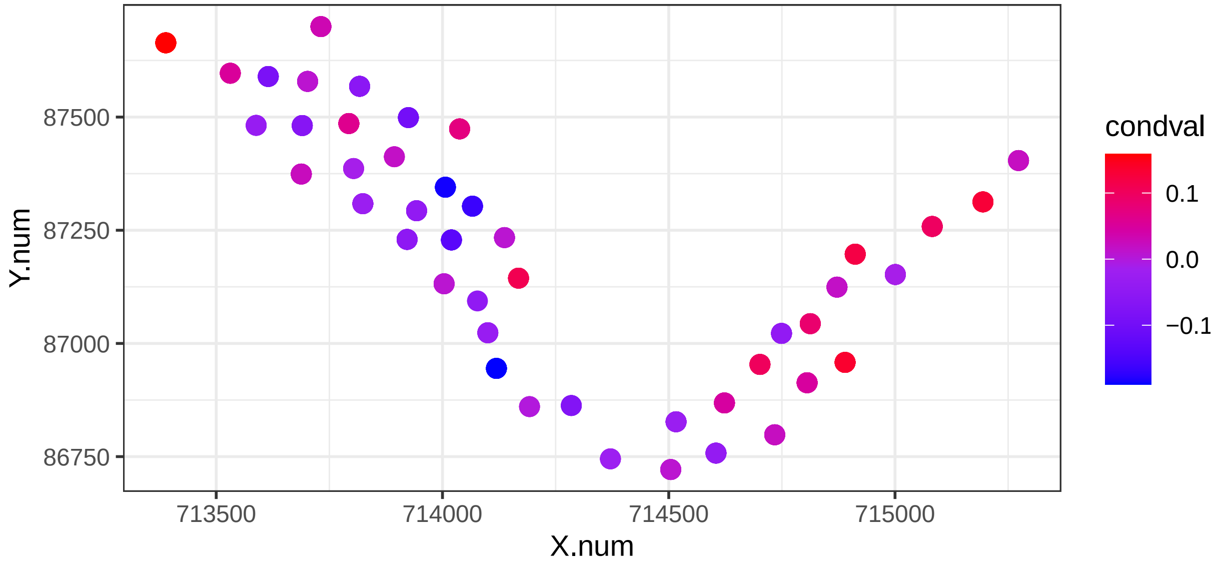

11.2 Hatched Eggs over space (varying over time)

```r
d.morcote.VM <- filter(d.ovitraps.23.mod,
                       municipality.fac %in% c("Morcote",
                                               "Vico Morcote"))
gam.hatch_space.Morcote.VM_vary <- gamV(
  cbind(hatched, non.hatched) ~
    # s(yday, pc = 182) +
    te(X.num, Y.num, yday), ## new element!
  family = "quasibinomial",
  data = d.morcote.VM)
```

```r
summary(gam.hatch_space.Morcote.VM_vary)
```

```
Family: quasibinomial
Link function: logit

Formula:
cbind(hatched, non.hatched) ~ te(X.num, Y.num, yday)

Parametric coefficients:
            Estimate Std. Error t value Pr(>|t|)
(Intercept)  0.93036    0.05219   17.83   <2e-16 ***
---
Signif. codes:  0 '***' 0.001 '**' 0.01 '*' 0.05 '.' 0.1 ' ' 1

Approximate significance of smooth terms:
                      edf Ref.df     F  p-value
te(X.num,Y.num,yday) 21.95  28.79 2.379 0.000137 ***
---
Signif. codes:  0 '***' 0.001 '**' 0.01 '*' 0.05 '.' 0.1 ' ' 1

R-sq.(adj) =  0.153   Deviance explained = 20.3%
-REML = -44.198  Scale est. = 5.5156    n = 352
```

```r
plotSlice(sm(gam.hatch_space.Morcote.VM_vary, 1),
          fix = list("yday" = quantile(d.morcote.VM$yday,
                                       probs = c(0, 0.2, 0.4, 0.6, 0.8),
                                       na.rm = TRUE)) ) +
  coord_fixed()
```

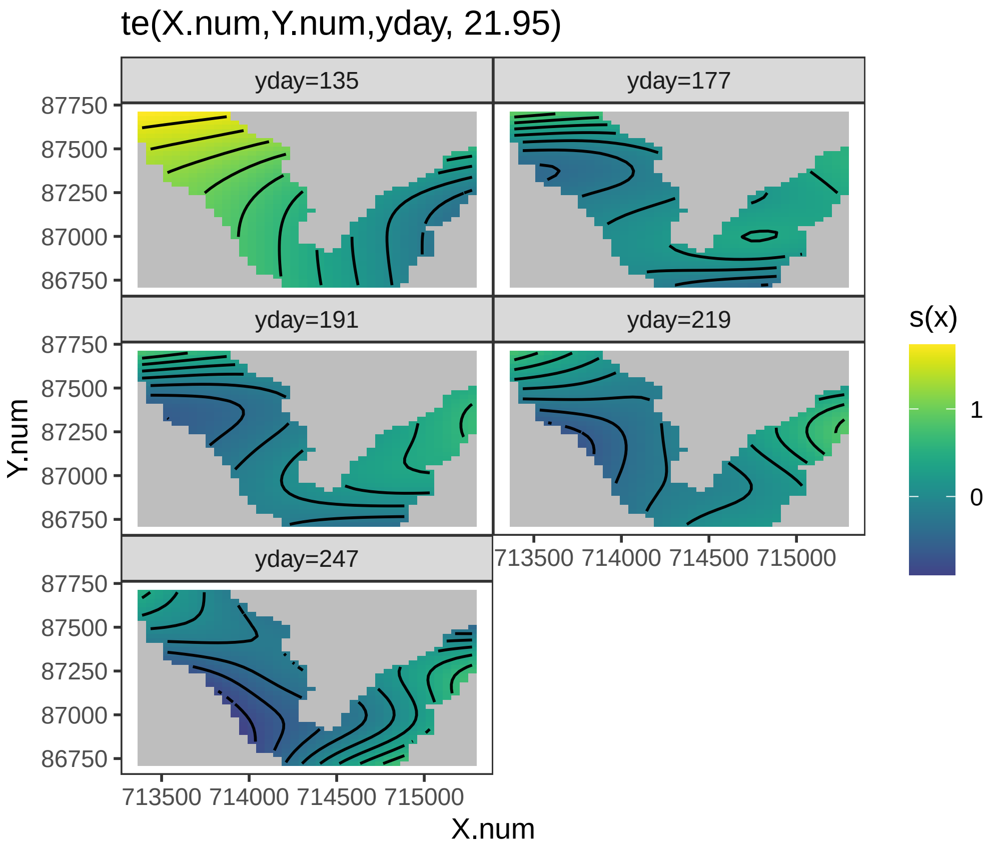

12 Appendix – Models including Vico Morcote

12.1 GAMM

12.1.1 Visualising the data

We start by displaying the percentage of eggs hatched in the traps sampled during the season. Observations from the same ovitrap are connected by lines to visualise trends over time. Above these lines, a smoothed average is plotted (with family "symmetric" to be more robust), allowing the overall trend of the percentage of eggs hatched to be seen.

```
## (messages and warnings are excluded from this chunk)
##
ggplot(data = d.ovitraps.23.hatch.proc,
       mapping = aes(y = `Perc.egg.hatch.per.trap`,
                     x = `Sampling_date.date`,
                     group = unique.ID)) +
  geom_hline(yintercept = c(0, 100), colour = "gray") +
  geom_point(alpha = 0.2) +
  geom_line(alpha = 0.2) +
  geom_smooth(mapping = aes(group = NULL),
              method = "loess",
              method.args = list(family = "symmetric"))
```

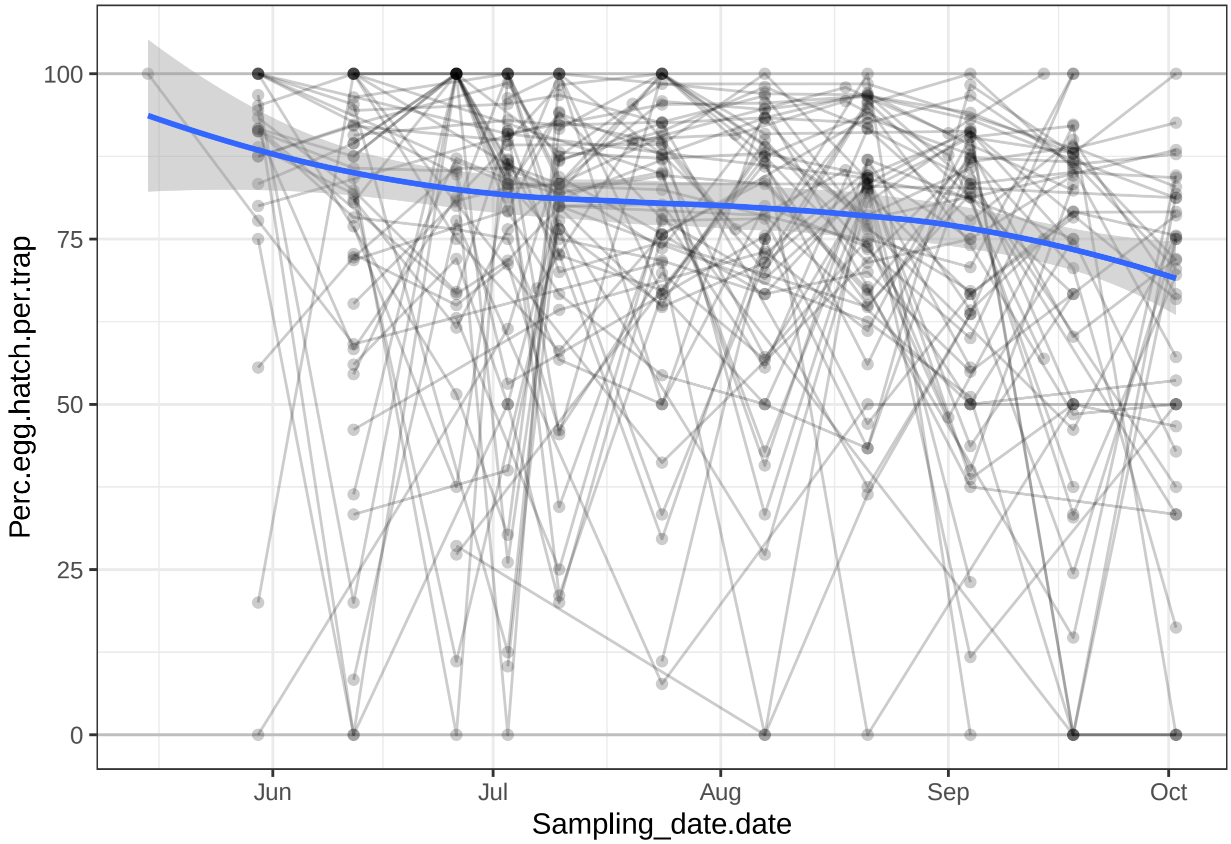

The sterile males were released only in one municipality (Morcote), while the others served as controls. For this reason, we now create a separate plot for each municipality to observe whether there are any differences between them.

```
## (messages and warnings are excluded from this chunk)
##
ggplot(data = d.ovitraps.23.hatch.proc,
       mapping = aes(y = `Perc.egg.hatch.per.trap`,
                     x = `Sampling_date.date`,
                     group = unique.ID)) +
  geom_hline(yintercept = c(0, 100), colour = "gray") +
  geom_point(alpha = 0.2) +
  coord_cartesian(ylim = c(0, 100)) +
  geom_line(alpha = 0.2) +
  facet_wrap(~municipality.fac) +
  geom_smooth(mapping = aes(group = 1), method = "loess",
              method.args = list(family = "symmetric"))
```

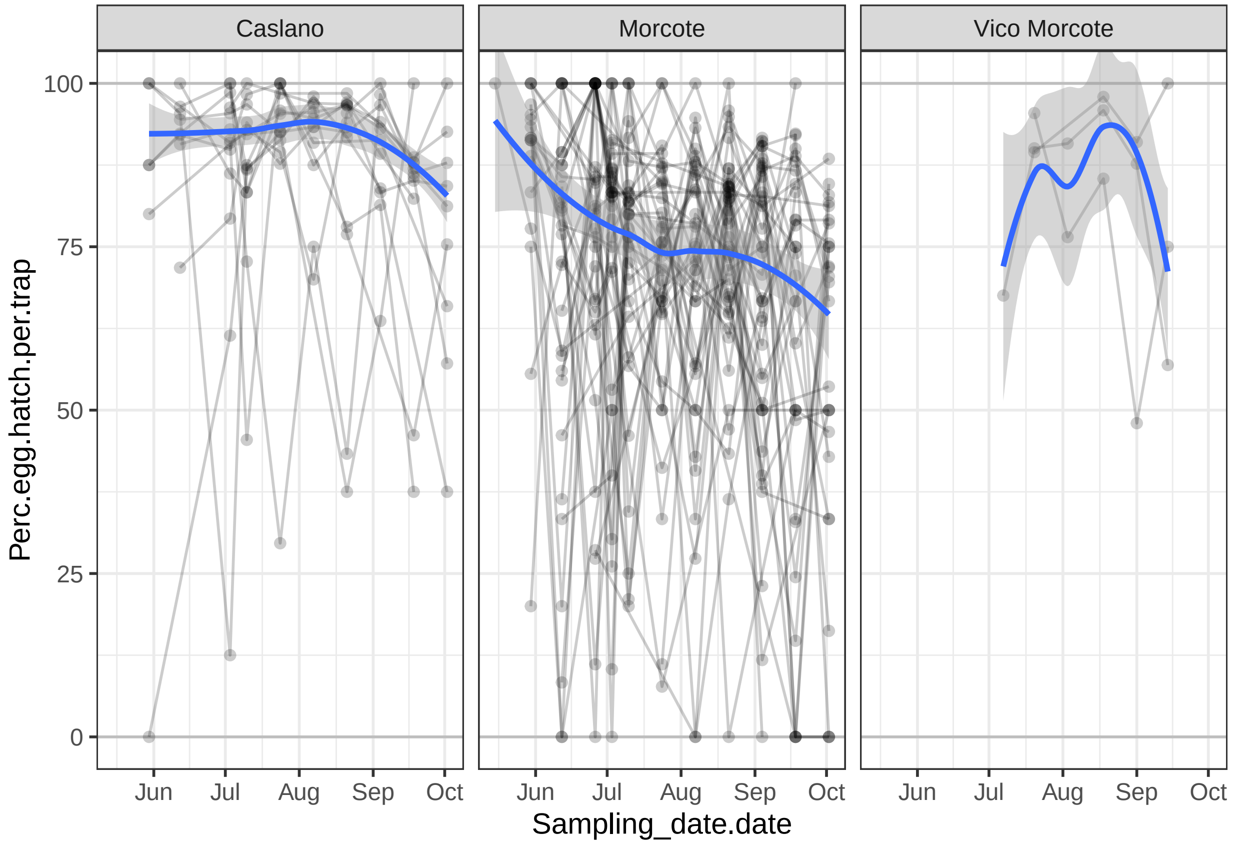

To facilitate comparison, rather than creating separate plots, we will overlay the municipalities on a single plot (using colours to distinguish them) and add a smoother for each one.

```
## (messages and warnings are excluded from this chunk)
##
ggplot(data = d.ovitraps.23.hatch.proc,
       mapping = aes(y = `Perc.egg.hatch.per.trap`,
                     x = `Sampling_date.date`,
                     group = unique.ID,
                     colour = municipality.fac)) +
  geom_hline(yintercept = c(0, 100), colour = "gray") +
  geom_point(alpha = 0.1) +
  geom_line(alpha = 0.1) +
  coord_cartesian(ylim = c(0,100)) +
  geom_smooth(mapping = aes(group = municipality.fac),
              method = "loess",
              method.args = list(family = "symmetric"))
```

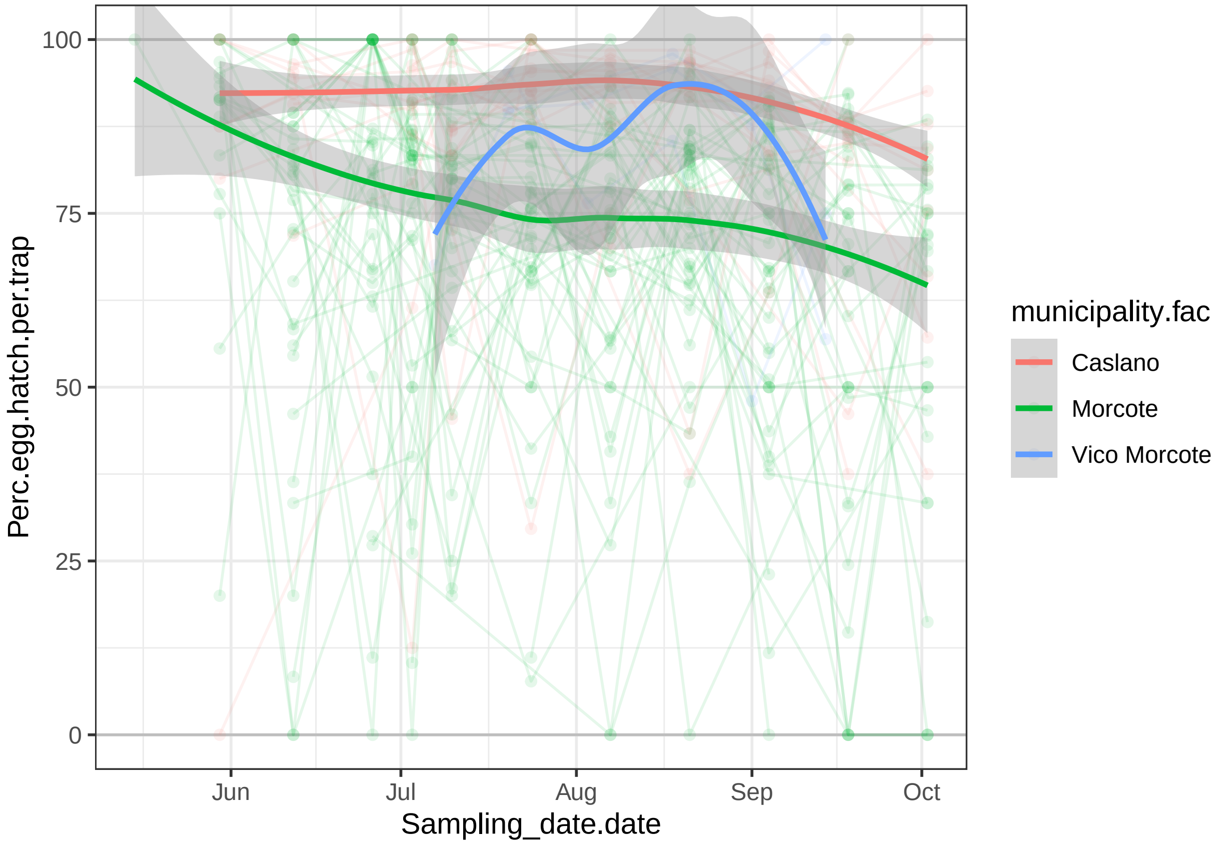

There seems to be a systematic difference between Caslano a Morcote. In Vico Morcote there are few observations to draw any strong conclusions.

Let's look at the same graph, but with mean lines instead of smoothers.

```
ggplot(data = d.ovitraps.23.hatch.proc,
       mapping = aes(y = Perc.egg.hatch.per.trap,
                     x = Sampling_date.date,
                     group = unique.ID,
                     colour = municipality.fac)) +
  geom_point(alpha = 0.1) +
  geom_line(alpha = 0.1) +
  coord_cartesian(ylim = c(0, 100)) +
  geom_hline(yintercept = c(0, 100), colour = "gray") +
  stat_summary(fun  = mean,
               fun.args = list(na.rm = TRUE),
               geom = "line",
               mapping = aes( group = municipality.fac))
```

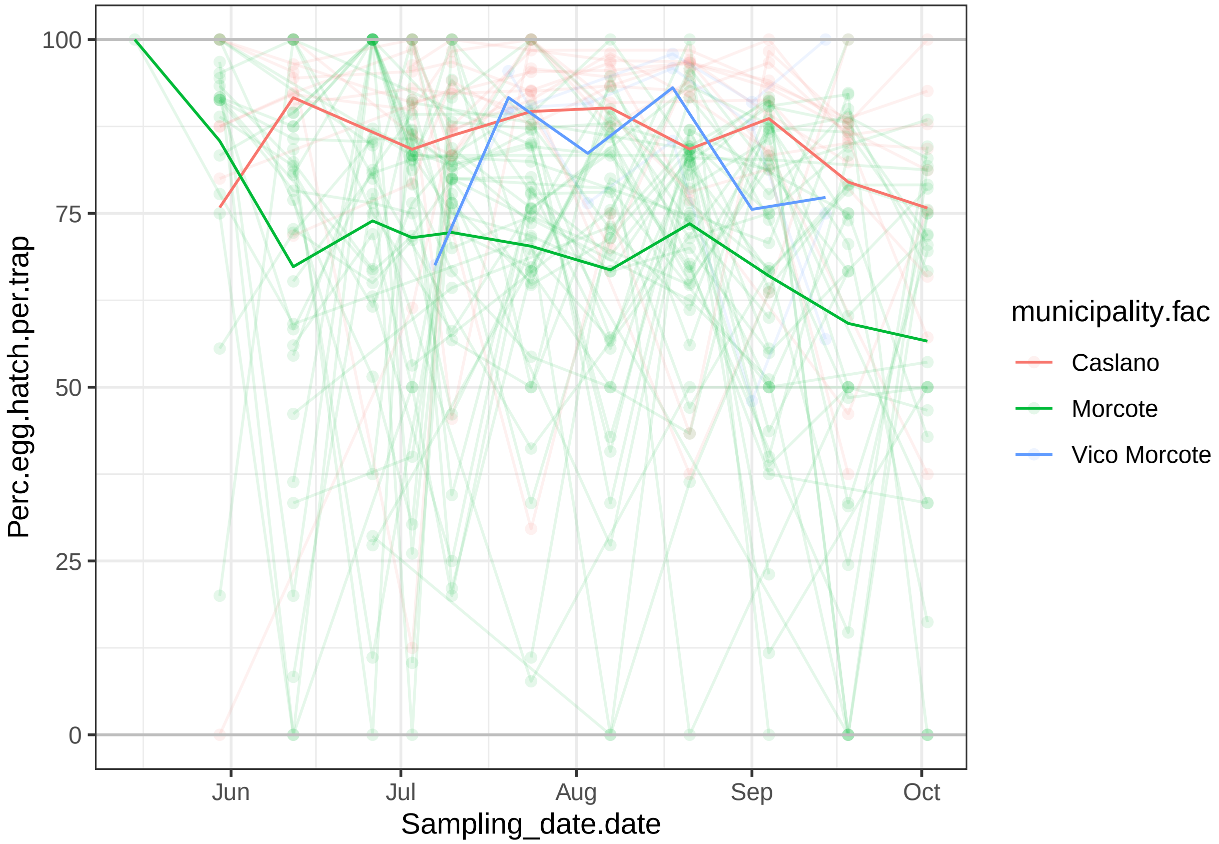

The conclusion remains the same.

Finally, we focus on Morcote to examine seasonal differences among the ovitraps. Since we are not concerned with the behaviour of individual ovitraps, we remove the labels.

```
## (messages and warnings are excluded from this chunk)
##
ggplot(data = filter(d.ovitraps.23.hatch.proc,
                     municipality.fac == "Morcote"),
       mapping = aes(y = `Perc.egg.hatch.per.trap`,
                     x = `Sampling_date.date`,
                     group = unique.ID)) +
  geom_hline(yintercept = c(0, 100), colour = "gray") +
  geom_point() +
  geom_line() +
  scale_y_sqrt(limits = c(0,NA), ) +
  facet_wrap(~unique.ID) +
  theme(
    strip.background = element_blank(),
    strip.text.x = element_blank(),
    axis.text.x = element_text(angle = 90))
```

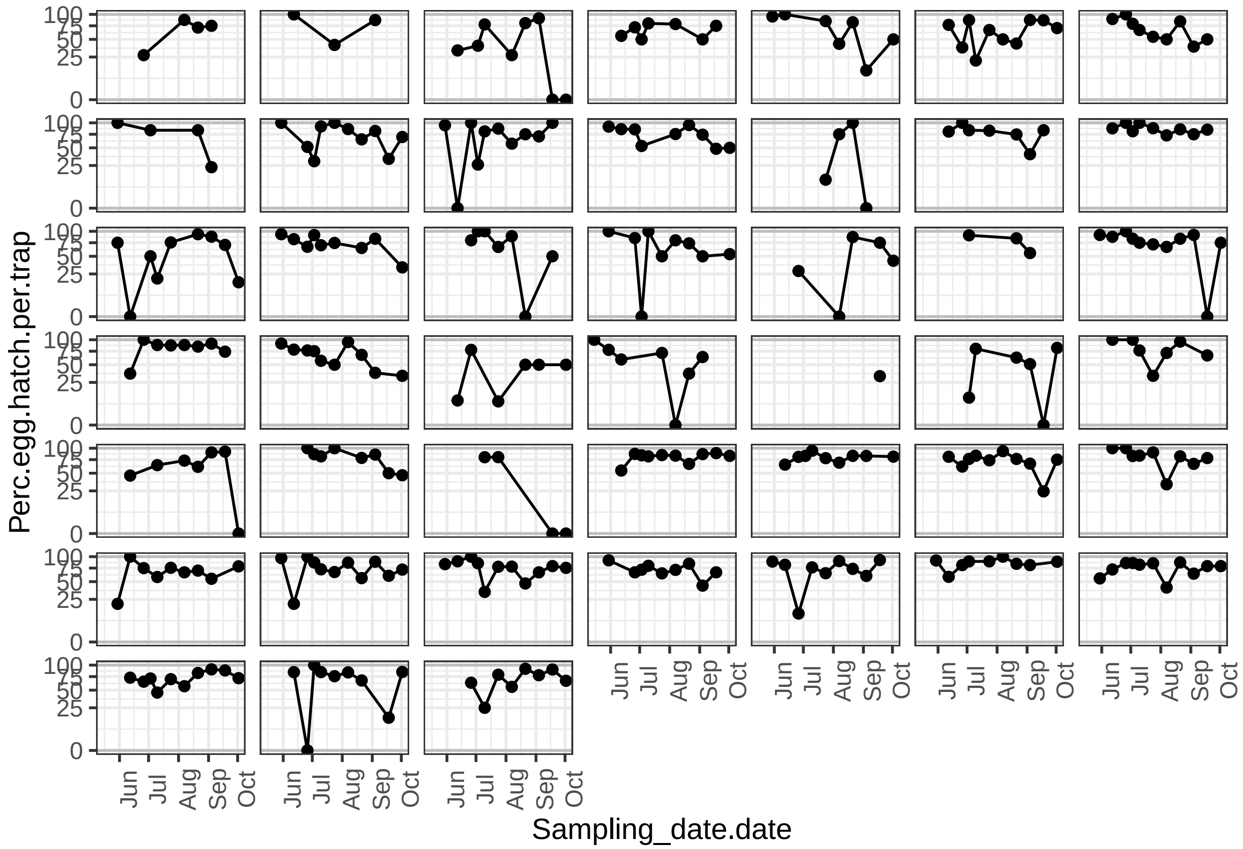

There is significant variation between ovitraps. For instance, in the second-last bottom panel, we can observe an abrupt drop from almost 100% hatched eggs one week to zero the next.

We create the same plot for Caslano to determine whether the observed behaviour is a result of the release of sterile males, or if it is a common pattern in the control municipalities as well.

```
## (messages and warnings are excluded from this chunk)
##
ggplot(data = filter(d.ovitraps.23.hatch.proc,
                     municipality.fac == "Caslano"),
       mapping = aes(y = `Perc.egg.hatch.per.trap`,
                     x = `Sampling_date.date`,
                     group = unique.ID)) +
  geom_hline(yintercept = c(0, 100), colour = "gray") +
  geom_point() +
  geom_line() +
  scale_y_sqrt(limits = c(0, NA)) +
  facet_wrap(~unique.ID) +
  theme(
    strip.background = element_blank(),
    strip.text.x = element_blank(),
    axis.text.x = element_text(angle = 90))
```

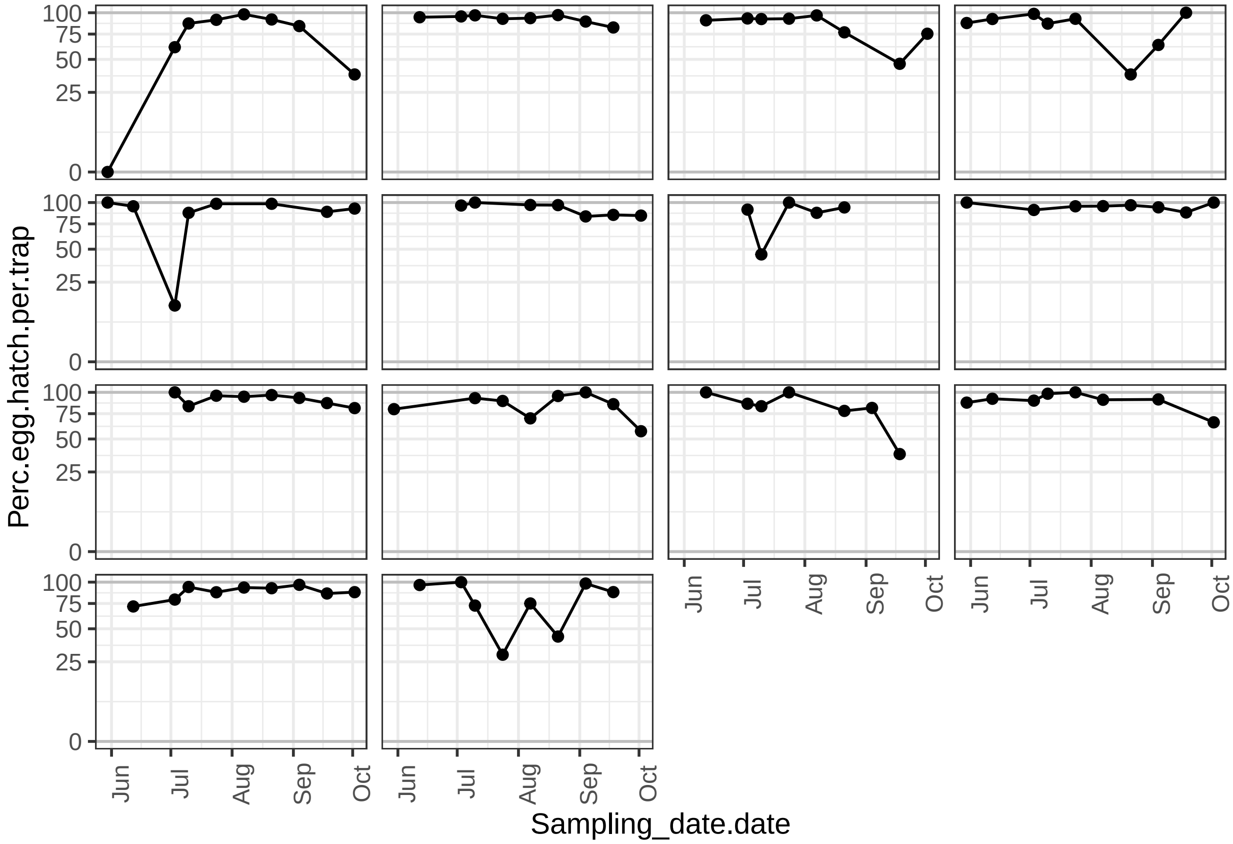

There is still some variability, but it is less pronounced compared to Morcote.

@Diego: Can this be due to the SIT males released?

12.1.2 Fitting the model

We aim to model the percentage of hatched *Aedes albopictus* eggs over the season (*yday*), while accounting for differences between municipalities (*municipality.fac*). Since sterile mosquitoes were released only in Morcote, municipalities will not be included as a random effect but rather as fixed effects to assess the actual differences.

The response variable is binomial, representing the counts of hatched versus non-hatched eggs (quantified by the variables *hatched.albo.eggs.after.proc* and *non.hatched.albo.eggs.after.proc*). To account for overdispersion, the quasi-binomial family is used for formal modelling.

Additionally, we will include an interaction between *municipality.fac* and *yday* to allow for distinct seasonal patterns across municipalities, avoiding the assumption that they behave similarly. These smooths will be centered, so the variable *municipality.fac* needs to be added as a main effect as well (because *municipality.fac* is a factor). In a subsequent section we will then test whether this distinct seasonal patterns across municipalities are needed.

yday will be included as numeric variable.

We begin without assuming any specific seasonal pattern, so we will fit a Generalised Additive Model (GAM), which provides flexibility in determining the best shape for the seasonal trend. However, this flexibility comes at the cost of interpretability.

Since observations within the same ovitrap are not independent, we will account for this dependency by setting *unique.ID* (the ovitrap variable) as a random effect. This adjustment captures the variability specific to each ovitrap.

Finally, some municipalities have fewer sampling dates, which impacts the number of knots used in the smooth term for the seasonal trend.

Let's check how many sampling dates there are for each municipality.

```
d.ovitraps.23 %>%
  group_by(municipality.fac) %>%
  summarise(nr.knots = n_distinct(yday)) %>%
  arrange(nr.knots)
```

```
# A tibble: 11 x 2
   municipality.fac  nr.knots
   <fct>                <int>
 1 Melide                   5
 2 Gravesano                6
 3 Manno                    6
 4 TaverneTorricella        6
 5 Vico Morcote             9
 6 Balerna                 10
 7 Canobbio                10
 8 Chiasso                 10
 9 Gordola                 10
10 Morcote                 25
11 Caslano                 34
```

Some municipalities have only five distinct values per *yday*. By default, when using the "by" argument into the `gam()` function, it adjust the maximum complexity based on the data available for each level of the variable specified in "by". For example, for municipalities like Morcote, which have more data points, the model will start from a larger basis dimension. However, this variation should not affect the overall conclusions.

Additionally, we will use a "point constraint" for *yday* (specifically at day 182), meaning all seasonal effects for *yday* are relative to the 1st of July.

```
as.Date("2023-07-01") %>% yday()
```

```
[1] 182
```

We will first remove the observations having missing values for the relevant variables.

```
d.ovitraps.23.mod <- d.ovitraps.23.hatch.proc %>%
  select(hatched.albo.eggs.after.proc,
         municipality.fac,
         non.hatched.albo.eggs.after.proc,
         Activation.time, unique.ID,
         Sampling_date.date,
         yday,
         municipality.ord,
         X.num, Y.num) %>%
  na.omit() %>%
  droplevels()
##
## check
dim(d.ovitraps.23)
```

```
[1] 2503   33
```

```
dim(d.ovitraps.23.mod)
```

```
[1] 475  10
```

We rename *hatched.albo.eggs.after.proc* and *non.hatched.albo.eggs.after.proc* to have simpler and smaller variable names.

```
d.ovitraps.23.mod <- d.ovitraps.23.mod %>%
  rename(hatched = hatched.albo.eggs.after.proc,
         non.hatched = non.hatched.albo.eggs.after.proc)
```

Now, we can fit the model. We use the `gamV()` function instead of `gam()`, because it fits the `gam()` model and automatically converts it to a `gamViz` object, making visualisation easier.
The model's estimates are exactly the same.

Note that the fitting procedure takes a few minutes. Therefore, the model is fitted and stored as RDS file and does not need to be refitted at each compilation.

```
## (This chunk is not evaluated)
##
gamm.hatched.eggs.23.VM <- gamV(
  cbind(hatched, non.hatched) ~
    s(yday, by = municipality.fac) +
    municipality.fac +
    s(unique.ID, bs = "re"),
  family = "quasibinomial",
  data = d.ovitraps.23.mod)

saveRDS(gamm.hatched.eggs.23.VM,
        file = "Prepared_data_and_models/GAMM_hatched_eggs.23.VM.RDS")
```

We also extract the standard deviation corresponding to the random effect.

```
gam.vcomp(gamm.hatched.eggs.23.VM) %>%
  tail(n = 1)
```

```
Standard deviations and 0.95 confidence intervals:

                                     std.dev        lower      upper
s(yday):municipality.facCaslano      0.004573667 0.0014229285 0.01470097
s(yday):municipality.facMorcote      0.002860517 0.0007877359 0.01038744
s(yday):municipality.facVico Morcote 0.017471574 0.0058000362 0.05263000
s(unique.ID)                         0.315672114 0.1992666432 0.50007810
scale                                2.438259177 2.2747924492 2.61347263

Rank: 5/5

       std.dev    lower    upper
scale 2.438259 2.274792 2.613473
```

12.1.3 Plotting the model

We now plot the fitted model. Since we allowed the model to fit a different trend for each municipality, there will be a separate plot for each one.

```
print(plot(gamm.hatched.eggs.23.VM,
           trans = plogis),
      pages = 1)
```

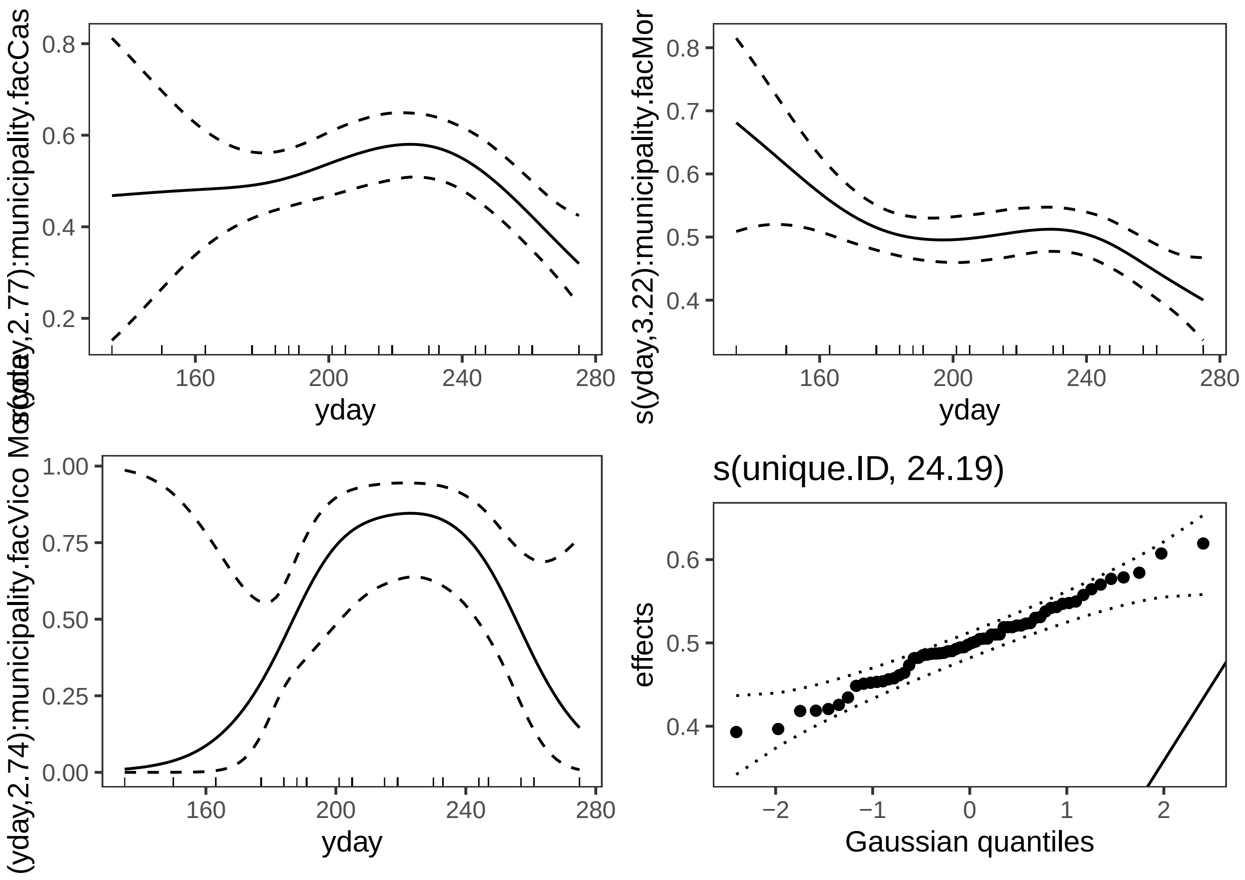

We can draw the graphs on the same plot with confidence intervals. This is performed to compare the different shapes.

```
## Extract data from the plots
gamm.hatched.eggs.23.VM.plot.tmp <- lapply(plot(gamm.hatched.eggs.23.VM)$plots,
                                           function(x) x$data$fit)
##
gamm.hatched.eggs.23.VM.plot.tmp <- lapply(1:(length(gamm.hatched.eggs.23.VM.plot.tmp)-1),
                                           function(ii) {
                                             out <- gamm.hatched.eggs.23.VM.plot.tmp[[ii]]
                                             out$municipality <- ii
                                             return(out)
                                           } )
##
## Combine data in a unique data set
gamm.hatched.eggs.23.VM.plot <- do.call("rbind", gamm.hatched.eggs.23.VM.plot.tmp)
##
## Create a factor for the municipality group
gamm.hatched.eggs.23.VM.plot$municipality <- as.factor(gamm.hatched.eggs.23.VM.plot$municipality)
##
## Create CI
gamm.hatched.eggs.23.VM.plot$upper <- gamm.hatched.eggs.23.VM.plot$ty + 2 * gamm.hatched.eggs.23.VM.plo
gamm.hatched.eggs.23.VM.plot$lower <- gamm.hatched.eggs.23.VM.plot$ty - 2 * gamm.hatched.eggs.23.VM.plo
##
## Plot the data
ggplot(data = gamm.hatched.eggs.23.VM.plot,
```

```
      mapping = aes(x = x, y = ty,
                    colour = municipality,
                    group = municipality,
                    fill = municipality)) +
  geom_line() +
  geom_ribbon(data = subset(gamm.hatched.eggs.23.VM.plot,
                            lower < y & y < upper),
              aes(ymin = lower, ymax = upper),
              alpha = 0.3,
              lty = 2) +
  coord_cartesian(ylim = c(-1, 2))
```

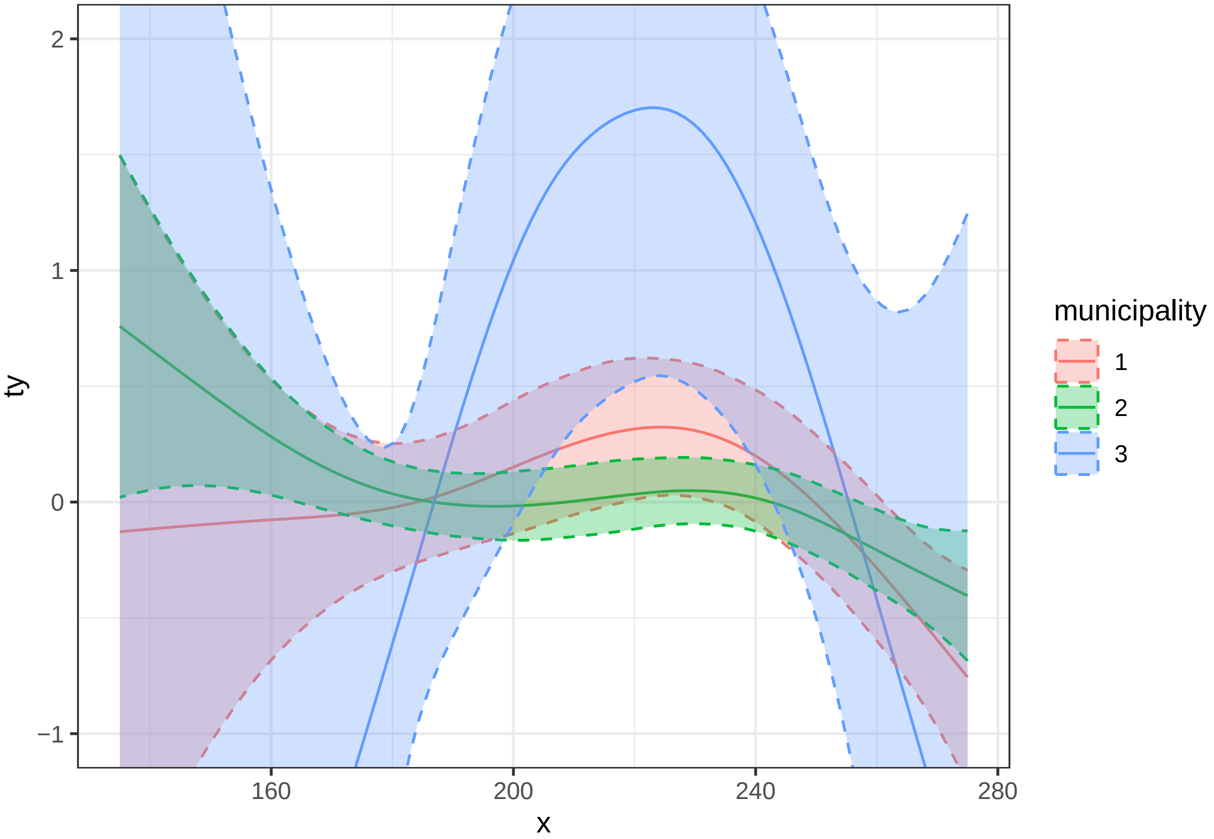

From this graph, allowing different shapes for the smoothers seems necessary. We will formally verify this later in the sections.

We draw again the same plots, separately, but adding the correct shift to all of them.

```
plot.gam(gamm.hatched.eggs.23.VM,
         trans = plogis,
         select = 1,
         shift = coef(gamm.hatched.eggs.23.VM)["(Intercept)"],
         main = levels(d.ovitraps.23.mod$municipality.fac)[1])
abline(h = c(0, 1), col = "grey", lty = 1)
```

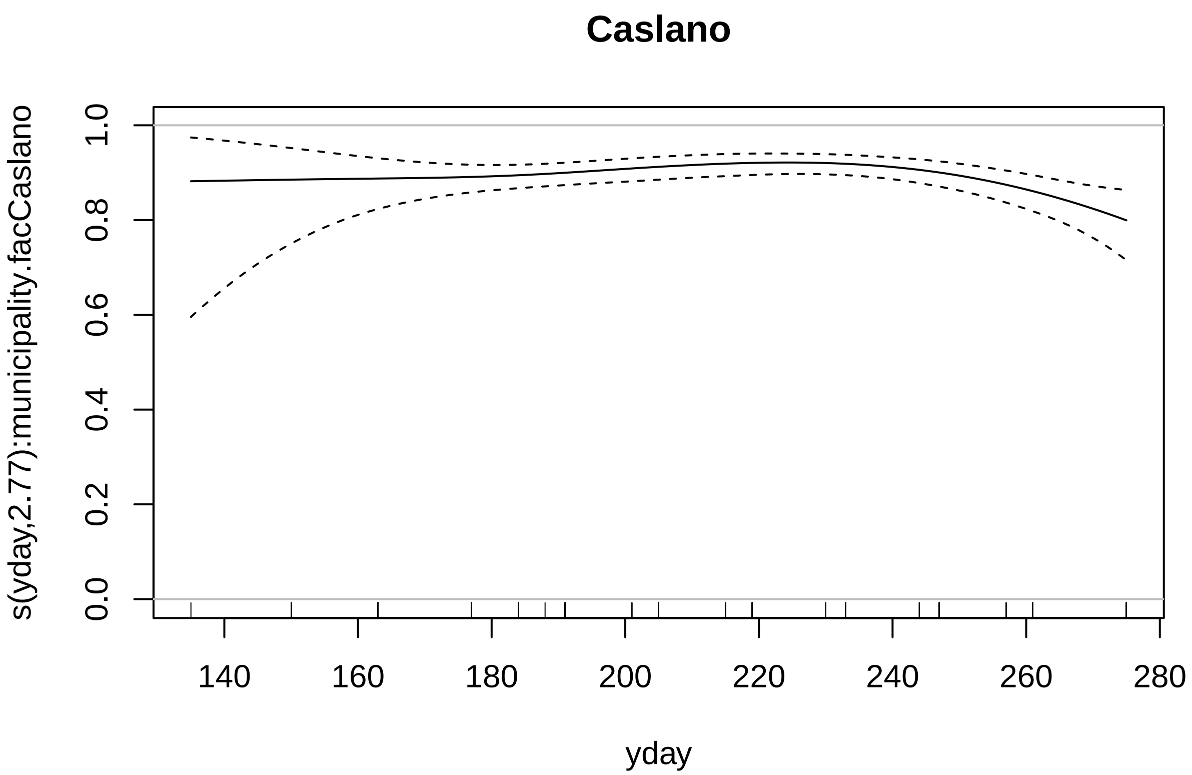


```
##
for (i in 2:nlevels(d.ovitraps.23.mod$municipality.fac)) {

  plot.gam(gamm.hatched.eggs.23.VM,
           trans = plogis,
           select = i,
           shift = coef(gamm.hatched.eggs.23.VM)["(Intercept)"] + coef(gamm.hatched.eggs.23.VM)[i],
           main =levels(d.ovitraps.23.mod$municipality.fac)[i])
  abline(h = c(0, 1), col = "grey", lty = 1)

}
```

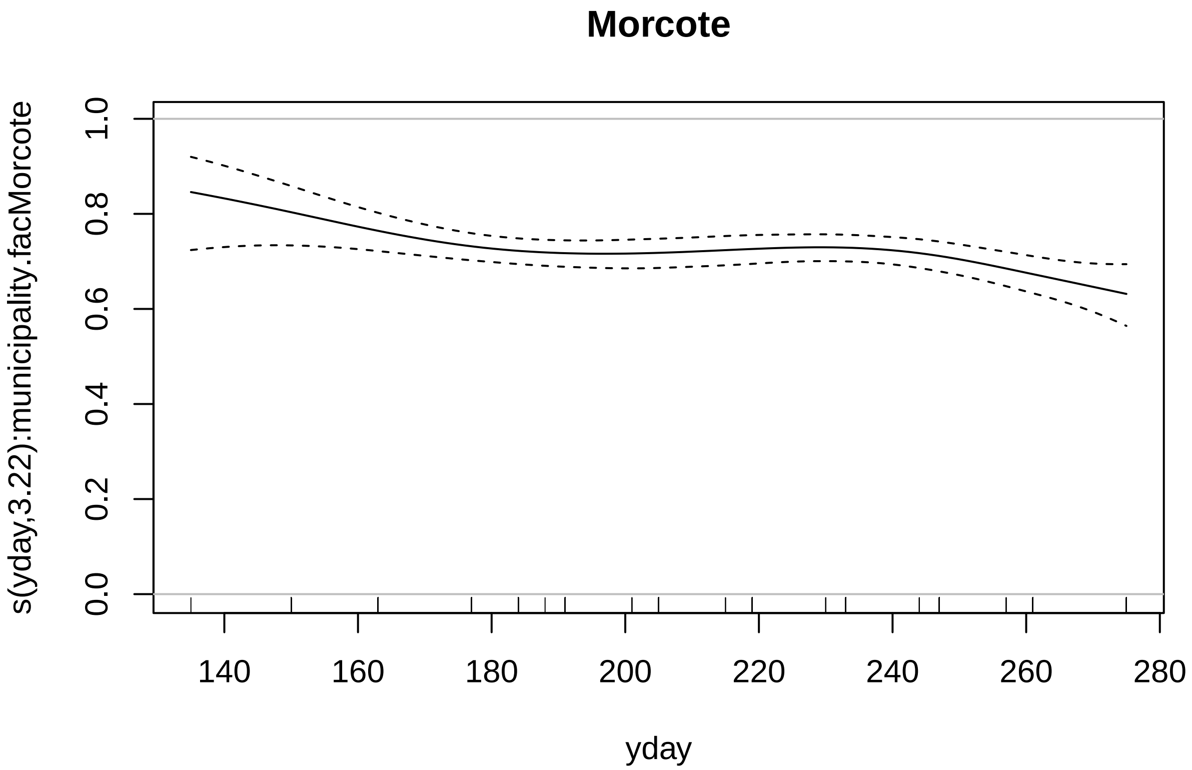

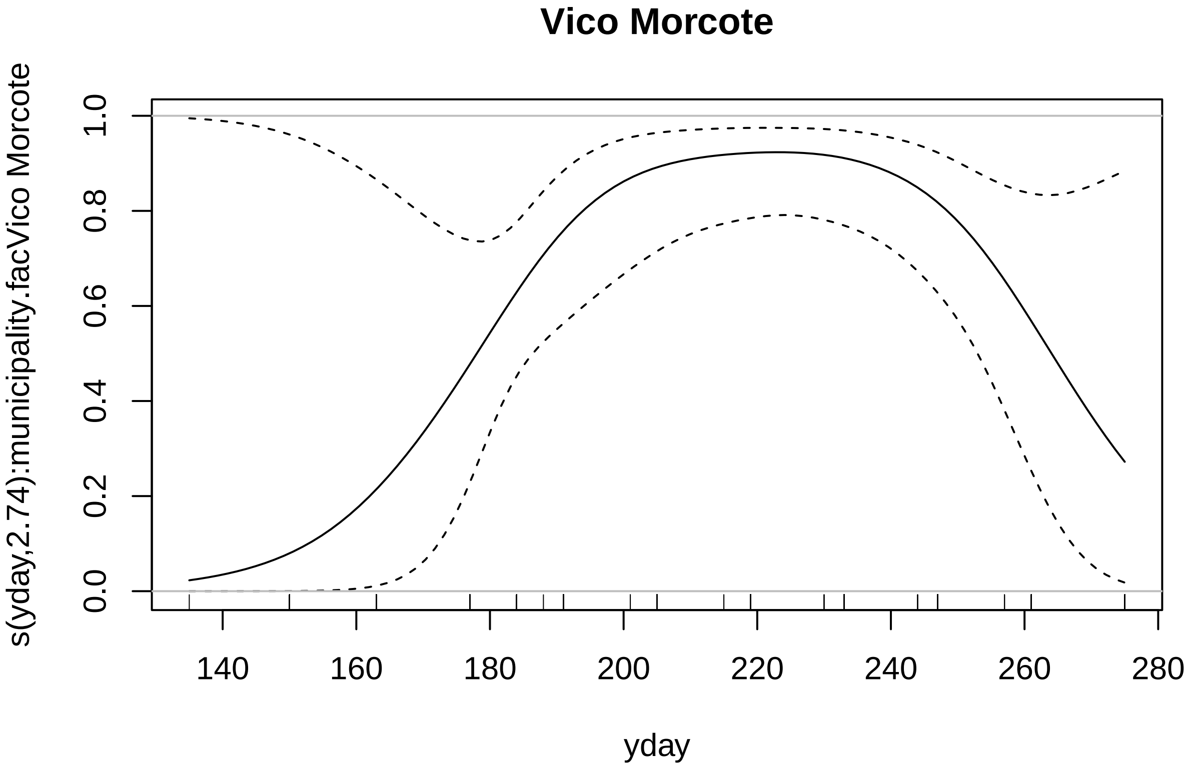

12.1.4 Fitted values

First, we begin by plotting the fitted values over time, creating a separate graph for each municipality. Observations from the same ovitrap will be connected by lines to visualise the trends.

```r
d.ovitraps.23.mod$fitted_gamm.hatched.eggs.23.VM <- fitted(gamm.hatched.eggs.23.VM)
##
ggplot(data = d.ovitraps.23.mod,
       mapping = aes(y = fitted_gamm.hatched.eggs.23.VM,
                     x = Sampling_date.date,
                     group = unique.ID)) +
  geom_hline(yintercept = c(0, 1)) +
  geom_point(alpha = 0.1) +
  geom_line(alpha = 0.1) +
  scale_y_sqrt() +
  facet_wrap(~municipality.fac)
```

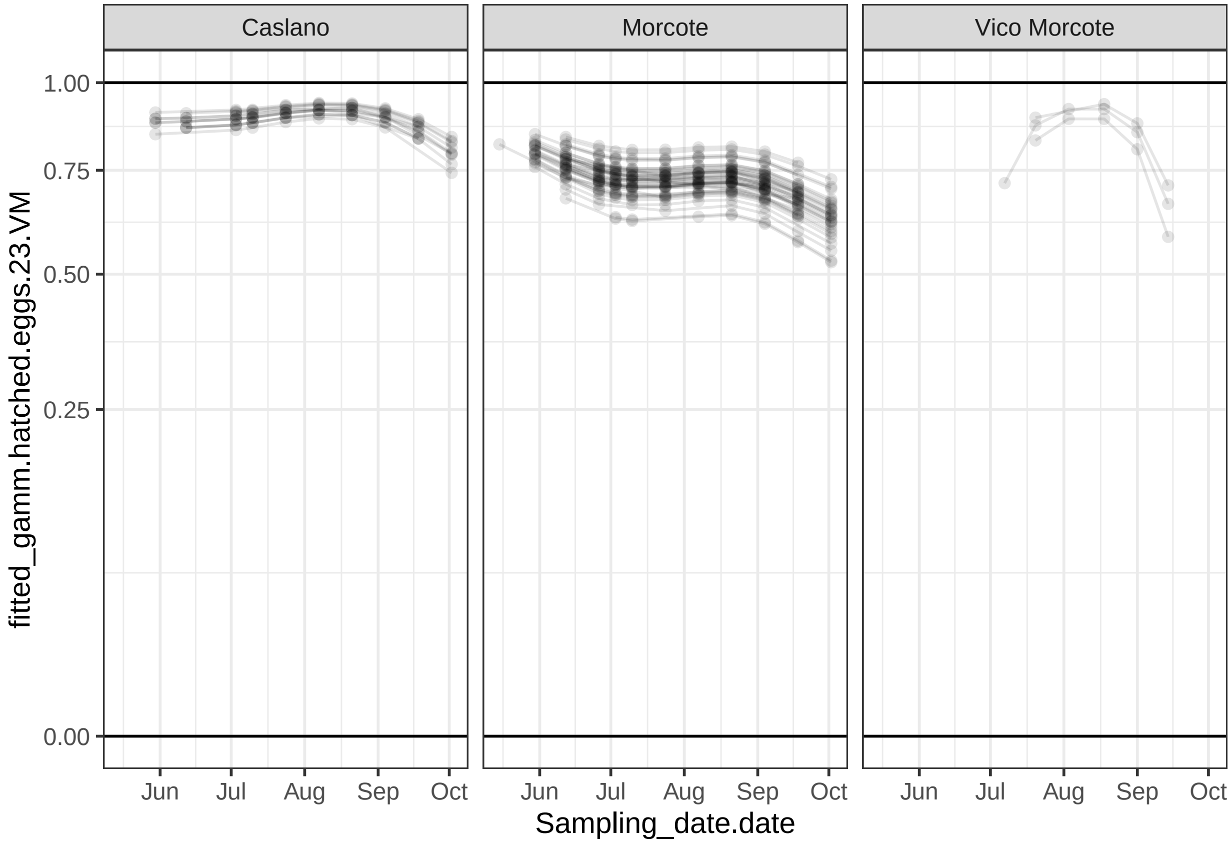

Note that there is quite some variability among ovitraps within Morcote.

We plot the three municipalities on the same graph to facilitate comparison.

```r
ggplot(data = d.ovitraps.23.mod,
       mapping = aes(y = fitted_gamm.hatched.eggs.23.VM,
                     x = Sampling_date.date,
                     group = unique.ID,
                     colour = municipality.fac)) +
  geom_point(alpha = 0.3) +
  geom_line(alpha = 0.3) +
  geom_hline(yintercept = c(0,1))
```

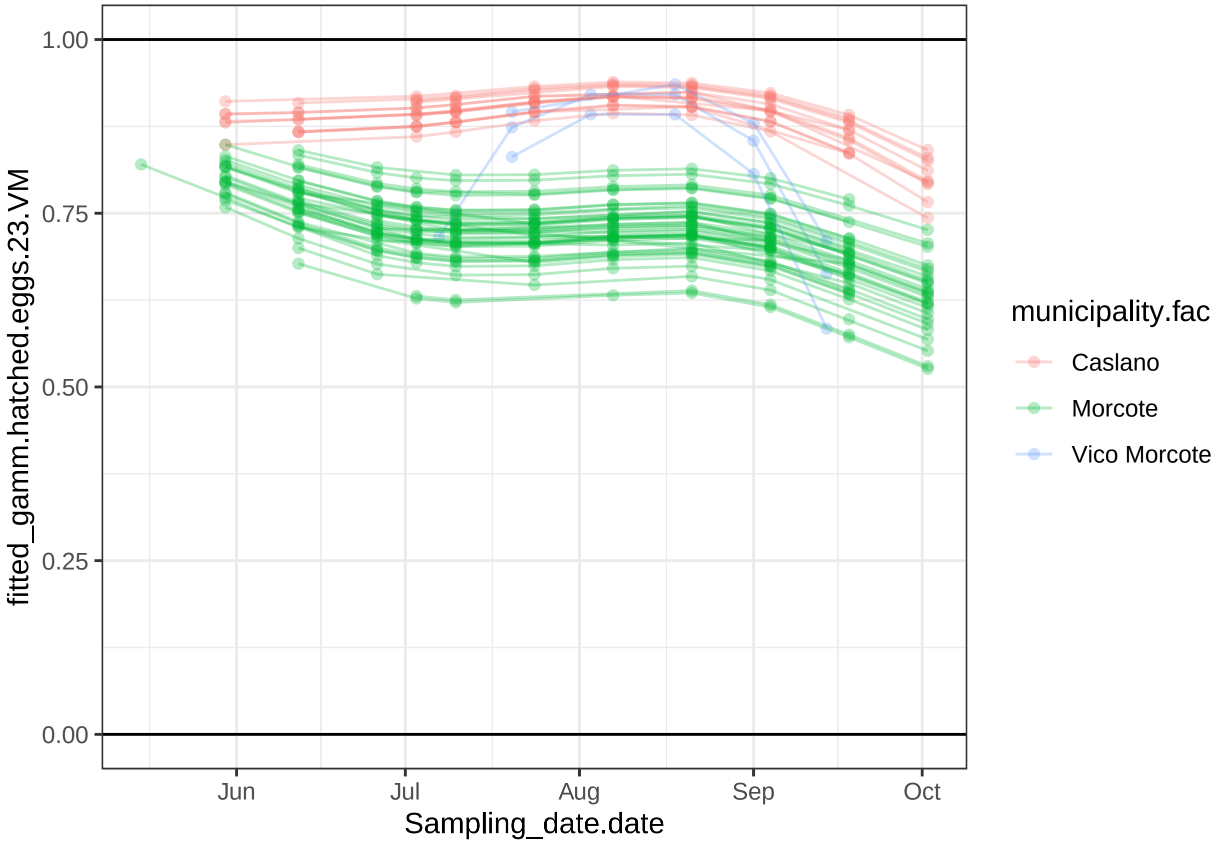

The fitted values for Morcote are systematically lower than Caslano.

12.1.5 Predicted values

Let's create the data set to make predictions on.

```
formula(gamm.hatched.eggs.23.VM)
```

```
cbind(hatched, non.hatched) ~ s(yday, by = municipality.fac) +
    municipality.fac + s(unique.ID, bs = "re")
```

```
##
## 1. We create a dataset with municipality and ovitrap ID
d.muni.ovitrap.short <- d.ovitraps.23.mod %>%
  select(municipality.fac, unique.ID) %>%
  unique()
##
head(d.muni.ovitrap.short)
```

```
# A tibble: 6 x 2
  municipality.fac unique.ID
  <fct>            <fct>
1 Vico Morcote     Vico Morcote.2
2 Vico Morcote     Vico Morcote.4
3 Vico Morcote     Vico Morcote.7
4 Morcote          Morcote.32a
5 Morcote          Morcote.1a
6 Morcote          Morcote.4a
```

```r
nrow(d.muni.ovitrap.short)
```

```
[1] 62
```

```r
##
## 2. We create dataset with varying yday and ovitrap ID
d.pred.gamm <- expand.grid(
  yday = seq(from = min(d.ovitraps.23.mod$yday),
             to = max(d.ovitraps.23.mod$yday),
             length.out = 100),
  # municipality = unique(d.modelling$municipality),
  unique.ID = d.muni.ovitrap.short$unique.ID)
##
## 3. We join the two datasets
## note that not all ovitraps exist in all municipalities.
## So, we can't simply use expand.grid()
d.pred.gamm_aug <- left_join(d.pred.gamm, d.muni.ovitrap.short,
                             by = join_by("unique.ID"))
str(d.pred.gamm_aug)
```

```
'data.frame':   6200 obs. of  3 variables:
 $ yday            : num  135 136 138 139 141 ...
 $ unique.ID       : Factor w/ 62 levels "Caslano.10a",..: 18 18 18 18 18 18 18 18 18 18 ...
 $ municipality.fac: Factor w/ 3 levels "Caslano","Morcote",..: 3 3 3 3 3 3 3 3 3 3 ...
 - attr(*, "out.attrs")=List of 2
  ..$ dim     : Named int [1:2] 100 62
  .. ..- attr(*, "names")= chr [1:2] "yday" "unique.ID"
  ..$ dimnames:List of 2
  .. ..$ yday     : chr [1:100] "yday=135.0000" "yday=136.4141" "yday=137.8283" "yday=139.2424" ...
  .. ..$ unique.ID: chr [1:62] "unique.ID=Vico Morcote.2" "unique.ID=Vico Morcote.4" "unique.ID=Vico Mo
```

We make the predictions on the newly created data set, at ovitrap and population level, and we plot the result.

```r
## Predictions at ovitrap level
d.pred.gamm_aug$predicted_gamm.hatched.eggs.23.VM <- predict(
  gamm.hatched.eggs.23.VM,
  newdata = d.pred.gamm_aug,
  type = "response")
##
## Predictions at population level
d.pred.gamm_aug$predicted_gamm.hatched.eggs.23.VM.pop <- predict(
  gamm.hatched.eggs.23.VM,
  newdata = d.pred.gamm_aug,
  type = "response",
  exclude = 's(unique.ID)')
##
ggplot(data = d.pred.gamm_aug,
       mapping = aes(y = predicted_gamm.hatched.eggs.23.VM,
                     x = yday,
                     group = unique.ID)) +
  geom_line(alpha = 0.2) +
  geom_line(mapping = aes(y = predicted_gamm.hatched.eggs.23.VM.pop),
            colour = "red",
            linewidth = 1) +
```

```
  geom_hline(yintercept = c(0, 1)) +
  facet_wrap(~municipality.fac)
```

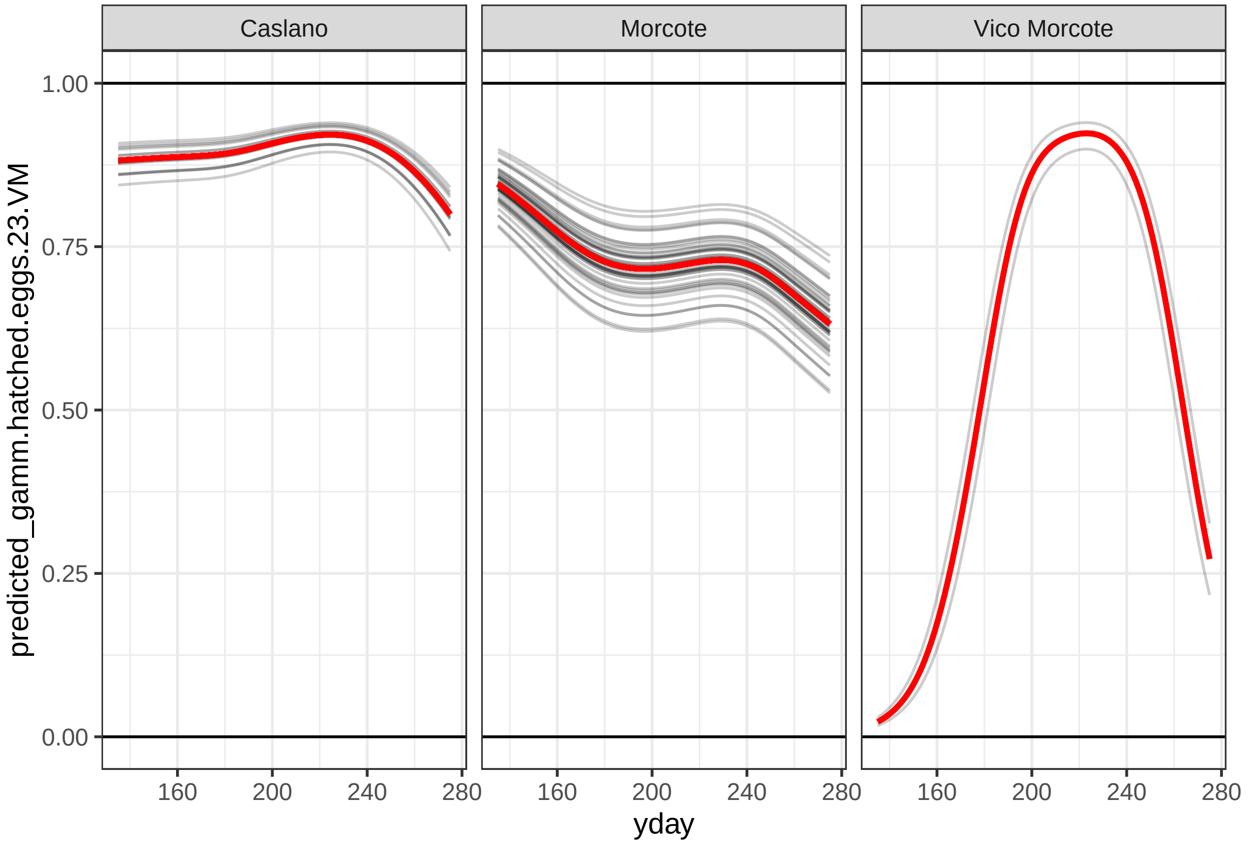

Each black line corresponds to the prediction for a given ovitrap, whereas the red line corresponds to the prediction at population level.

The shapes can be quite different between municipalities. The variability within Morcote is also quite significant.
Note that Vico Morcote has fewer observations than the other municipalities. Also, the measurements started later in the season and stopped earlier for this municipality, so the predicted values at the extremes cannot be reliable.

We plot the three predictions at population level on the same plot.

```
ggplot(data = d.pred.gamm_aug,
       mapping = aes(y = predicted_gamm.hatched.eggs.23.VM.pop,
                     x = yday,
                     colour = municipality.fac)) +
  geom_line() +
  geom_hline(yintercept = c(0, 1))
```

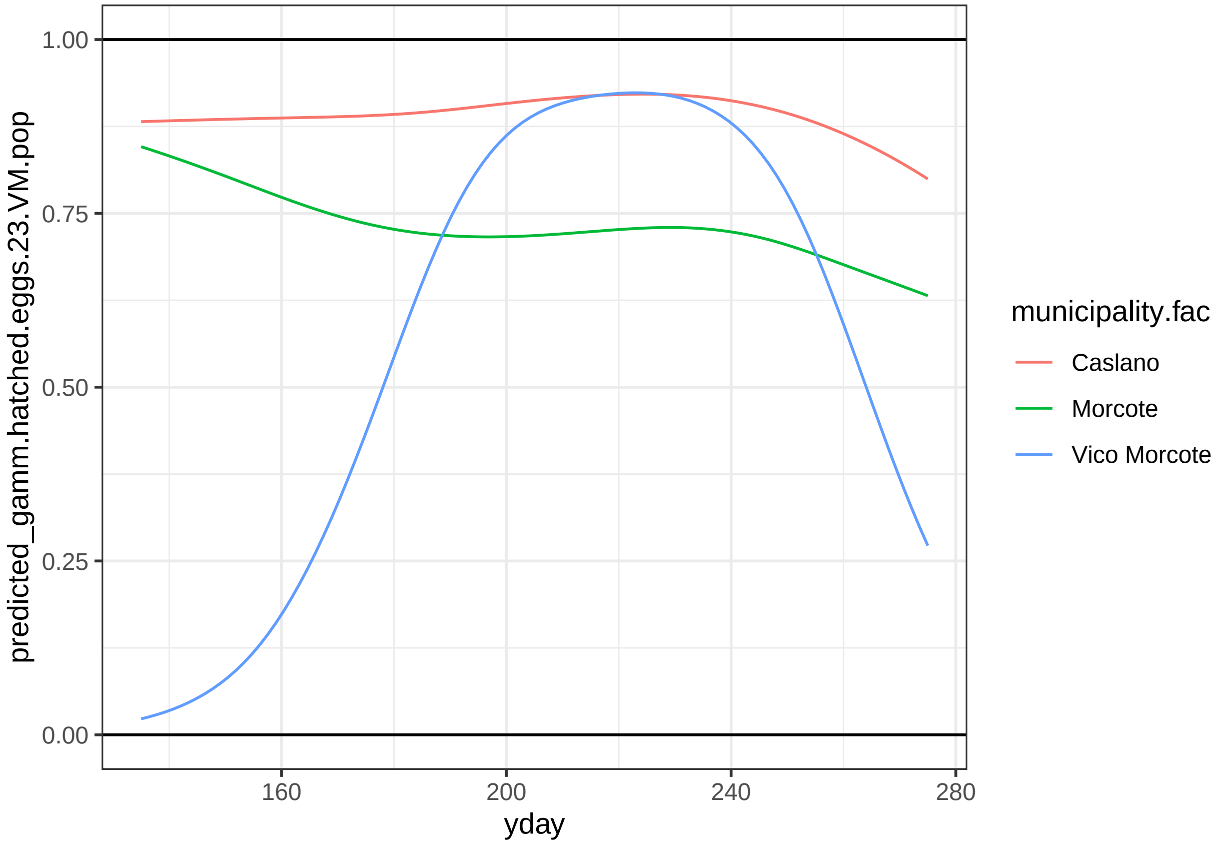

As already mentioned, Vico Morcote has fewer observations than the other municipalities, in particular at the beginning and end of the season, therefore the predictions are not reliable. However, for Caslano and Morcote there is a systematic difference, with Morcote systematically lower than Caslano.

We conclude that Vico Morcote shows intermediate results as it is close to SIT.

12.1.6 Model selection – Shape

We fitted a model allowing a different smoother for each municipality. Now, we will assess whether this flexibility is truly necessary.

With this purpose in mind, we refit the model, but this time without allowing for different shapes across municipalities. We will then compare the two models to determine if the difference between them is statistically significant.

For performing this comparison, we need to refit the first model using an ordered factor (*municipality.ord*) instead of the classical one. This allows us to have nested model matrices and formally compare the two models.

```
## (this chunk is not evaluated)
gamm.hatched.eggs.23.VM.ord <- gam(
  cbind(hatched, non.hatched) ~
    s(unique.ID, bs = "re") +
    municipality.fac +
    s(yday, pc = 182) +

    s(yday, by = municipality.ord),
  family = "quasibinomial",
```

```r
  data = d.ovitraps.23.mod)
##
saveRDS(gamm.hatched.eggs.23.VM.ord,
        "Prepared_data_and_models/GAMM_hatched_eggs_ord.23.VM.RDS")
##
gamm.hatched.eggs.23.VM.smooth <- gam(
  cbind(hatched, non.hatched) ~
    s(unique.ID, bs = "re") +
    municipality.fac +
    s(yday, pc = 182),

  # s(yday, by = municipality.ord) +,
  family = "quasibinomial",
  data = d.ovitraps.23.mod)
##
saveRDS(gamm.hatched.eggs.23.VM.smooth,
        "Prepared_data_and_models/GAMM_hatched_eggs_one_smooth.23.VM.RDS")
```

Let's get the previously fitted models.

```r
gamm.hatched.eggs.23.VM.ord <- readRDS(paste0("Prepared_data_and_models/",
                                              "GAMM_hatched_eggs_ord.23.VM.RDS"))
gamm.hatched.eggs.23.VM.smooth <- readRDS(paste0("Prepared_data_and_models/",
                                                 "GAMM_hatched_eggs_one_smooth.23.VM.RDS"))
##
summary(gamm.hatched.eggs.23.VM.ord)
```

```

Family: quasibinomial
Link function: logit

Formula:
cbind(hatched, non.hatched) ~ s(unique.ID, bs = "re") + municipality.fac +
    s(yday, pc = 182) + s(yday, by = municipality.ord)

Parametric coefficients:
                             Estimate Std. Error t value Pr(>|t|)
(Intercept)                    2.1347     0.2017  10.583  < 2e-16 ***
municipality.facMorcote       -1.2201     0.1615  -7.556 2.46e-13 ***
municipality.facVico Morcote  -1.1934     0.4746  -2.515   0.0123 *
---
Signif. codes:  0 '***' 0.001 '**' 0.01 '*' 0.05 '.' 0.1 ' ' 1

Approximate significance of smooth terms:
                                    edf Ref.df     F  p-value
s(unique.ID)                     26.844 59.000 0.900 0.000164 ***
s(yday)                           3.916  4.782 3.635 0.004052 **
s(yday):municipality.ordMorcote   2.055  2.544 0.936 0.405061
s(yday):municipality.ordVico Morcote  2.231  2.771 2.122 0.077853 .
---
Signif. codes:  0 '***' 0.001 '**' 0.01 '*' 0.05 '.' 0.1 ' ' 1

R-sq.(adj) =  0.288   Deviance explained = 38.5%
GCV = 6.4038  Scale est. = 5.7078    n = 475
```

```
summary(gamm.hatched.eggs.23.VM.smooth)
```

```

Family: quasibinomial
Link function: logit

Formula:
cbind(hatched, non.hatched) ~ s(unique.ID, bs = "re") + municipality.fac +
    s(yday, pc = 182)

Parametric coefficients:
                             Estimate Std. Error t value Pr(>|t|)
(Intercept)                    2.1361     0.1526  13.995  < 2e-16 ***
municipality.facMorcote       -1.2370     0.1524  -8.116  4.8e-15 ***
municipality.facVico Morcote  -0.3661     0.2956  -1.238    0.216
---
Signif. codes:  0 '***' 0.001 '**' 0.01 '*' 0.05 '.' 0.1 ' ' 1

Approximate significance of smooth terms:
                edf Ref.df     F  p-value
s(unique.ID) 25.530 59.000 0.841 0.000256 ***
s(yday)       4.041  4.947 5.641  5.5e-05 ***
---
Signif. codes:  0 '***' 0.001 '**' 0.01 '*' 0.05 '.' 0.1 ' ' 1

R-sq.(adj) =  0.275   Deviance explained = 36.1%
GCV =  6.487  Scale est. = 5.7937    n = 475
```

We check whether the two models are nested. If they are, then we can test whether the most complicated one is necessary, i.e., if the difference of the two models is statistically significant.

```
m.gamm.hatched.eggs.23.VM.ord <- model.matrix(gamm.hatched.eggs.23.VM.ord)
m.gamm.hatched.eggs.23.VM.smooth <- model.matrix(gamm.hatched.eggs.23.VM.smooth) ##
dim(m.gamm.hatched.eggs.23.VM.ord)
```

```
[1] 475  92
```

```
dim(m.gamm.hatched.eggs.23.VM.smooth)
```

```
[1] 475  74
```

```
##
n1 <- ncol(m.gamm.hatched.eggs.23.VM.smooth)
##
## check that sum is zero
sum(matrix( m.gamm.hatched.eggs.23.VM.smooth[, 1:n1] -
             m.gamm.hatched.eggs.23.VM.ord[, 1:n1] ) != 0)
```

```
[1] 0
```

The two model matrices are nested, therefore we can now compare the two models with the Chi-square test.

```
anova.gam(gamm.hatched.eggs.23.VM.smooth, gamm.hatched.eggs.23.VM.ord, test = "Chisq")
```

```
Analysis of Deviance Table

Model 1: cbind(hatched, non.hatched) ~ s(unique.ID, bs = "re") + municipality.fac +
    s(yday, pc = 182)
```

```
Model 2: cbind(hatched, non.hatched) ~ s(unique.ID, bs = "re") + municipality.fac +
    s(yday, pc = 182) + s(yday, by = municipality.ord)
  Resid. Df Resid. Dev     Df Deviance Pr(>Chi)
1    428.83     2673.2
2    422.29     2574.0 6.5461   99.211  0.01142 *
---
Signif. codes:  0 '***' 0.001 '**' 0.01 '*' 0.05 '.' 0.1 ' ' 1
```

The difference between the two models is statistically significant, which means that the additional flexibility, allowing different shapes for the smoother in each municipality, is necessary.

Please note that AIC and BIC cannot be calculated for models having a "quasi" family, thus we cannot double check the anova result with these additional tools.

12.1.7 Residual analysis

Note that the residual analysis is run on the main model (i.e., *gamm.hatched.eggs.23.VM*).

First of all, we apply the `gam.check()` function to the model, which produces some diagnostic information.

```r
par(mfrow = c(2, 2))
gam.check(gamm.hatched.eggs.23.VM)
```

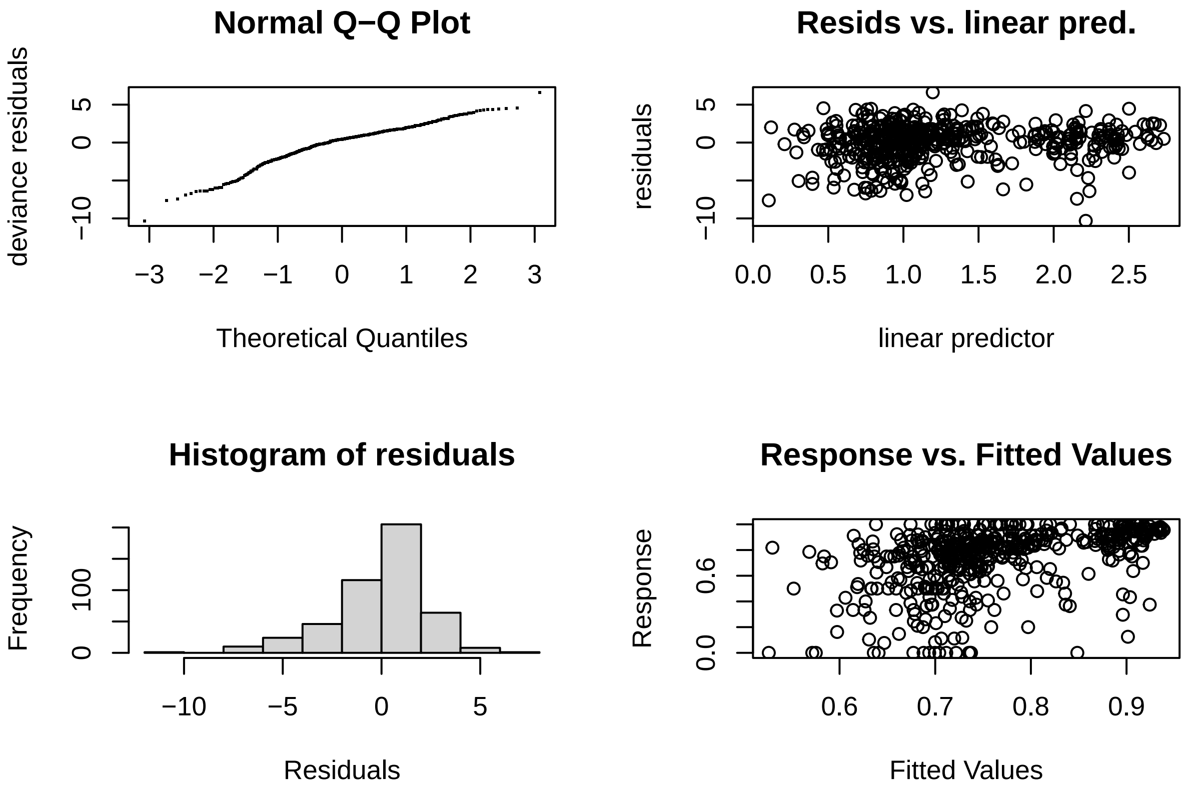


```
Method: REML   Optimizer: outer newton
full convergence after 6 iterations.
Gradient range [-2.006654e-09,7.656809e-11]
(score -71.50903 & scale 5.789144).
Hessian positive definite, eigenvalue range [0.5704011,235.1395].
Model rank =  92 / 92
```

```
Basis dimension (k) checking results. Low p-value (k-index<1) may
indicate that k is too low, especially if edf is close to k'.

                                        k'   edf k-index p-value
s(yday):municipality.facCaslano       9.00  2.77    1.05    0.84
s(yday):municipality.facMorcote       9.00  3.22    1.05    0.80
s(yday):municipality.facVico Morcote  9.00  2.74    1.05    0.81
s(unique.ID)                         62.00 24.19      NA      NA
```

```
par(mfrow = c(1, 1))
```

Then, we store the pearson residuals in the original data frame, and we plot the residuals against the fitted values to see whether there is still structure in the data.

First of all, we store the pearson residuals in the original data frame. Then we plot the residuals against the fitted values to see whether there is still structure in the data.

```
d.ovitraps.23.mod$resid_gamm.hatched.eggs.23.VM <-
  resid(gamm.hatched.eggs.23.VM, type = "pearson")
##
ggplot(data = d.ovitraps.23.mod,
       mapping = aes(y = resid_gamm.hatched.eggs.23.VM,
                     x = fitted_gamm.hatched.eggs.23.VM)) +
  geom_hline(yintercept = 0) +
  geom_point(alpha = 0.2) +
  geom_smooth()
```

```
`geom_smooth()` using method = 'loess' and formula = 'y ~ x'
```

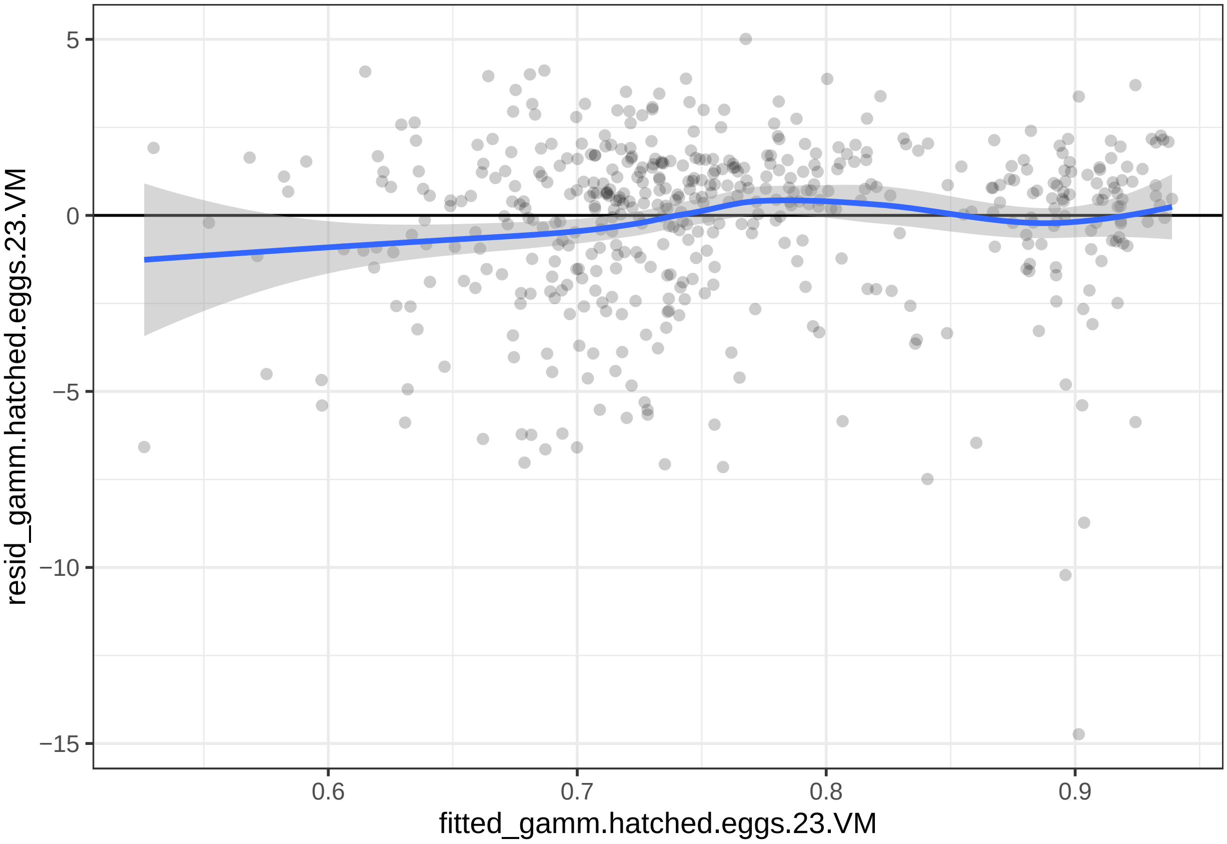

The blue line is located on the x-axis, indicating that there doesn't seem to be structure left in the residuals.

We plot the qq-plot for the random effects.

```
## QQ for random effects
plot(sm(gamm.hatched.eggs.23.VM, 4))
```

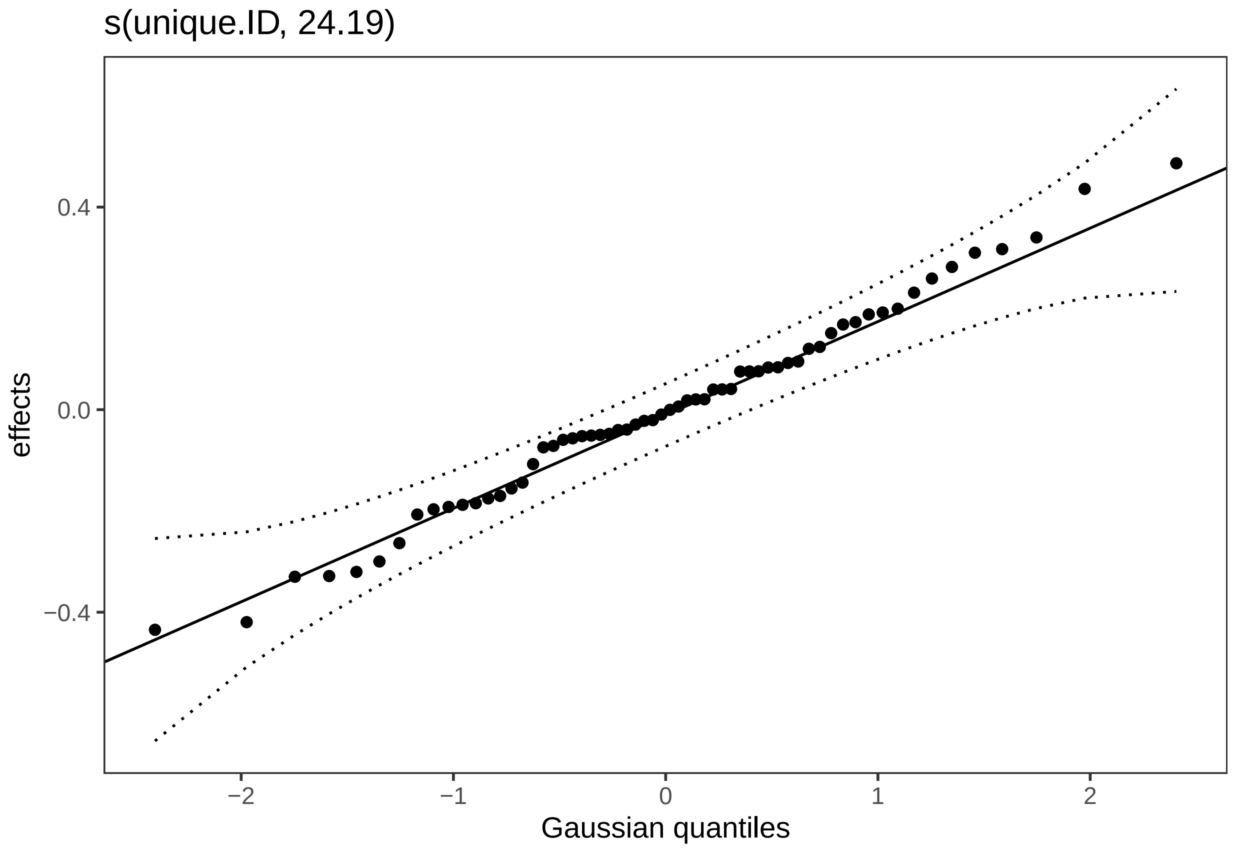

There isn't a significant departure from the normality assumption.

We now plot the residuals over time to further check whether there is any structure left in the data.

```
ggplot(data = d.ovitraps.23.mod,
       mapping = aes(y = resid_gamm.hatched.eggs.23.VM,
                     x = yday)) +
  geom_hline(yintercept = 0) +
  geom_point(alpha = 0.2) +
  geom_smooth(method = "loess")
```

```
`geom_smooth()` using formula = 'y ~ x'
```

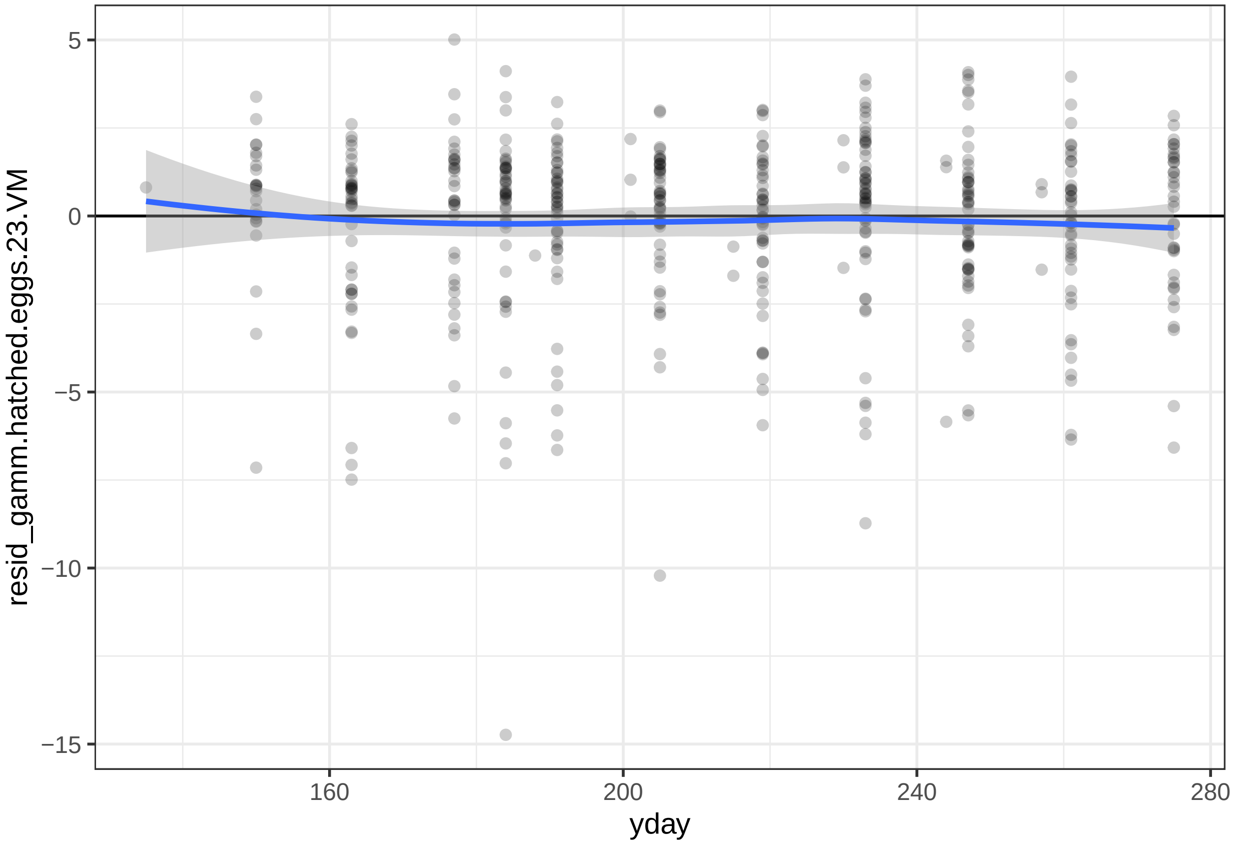

There doesn't seem to be any structure left in the data. Let's look into each single smoother (i.e. municipality).

```
ggplot(data = d.ovitraps.23.mod,
       mapping = aes(y = resid_gamm.hatched.eggs.23.VM,
                     x = yday)) +
  geom_hline(yintercept = 0) +
  geom_point(alpha = 0.2) +
  geom_smooth(method = "loess") +
  facet_wrap(~municipality.fac, scales = "free")
```

```
`geom_smooth()` using formula = 'y ~ x'
```

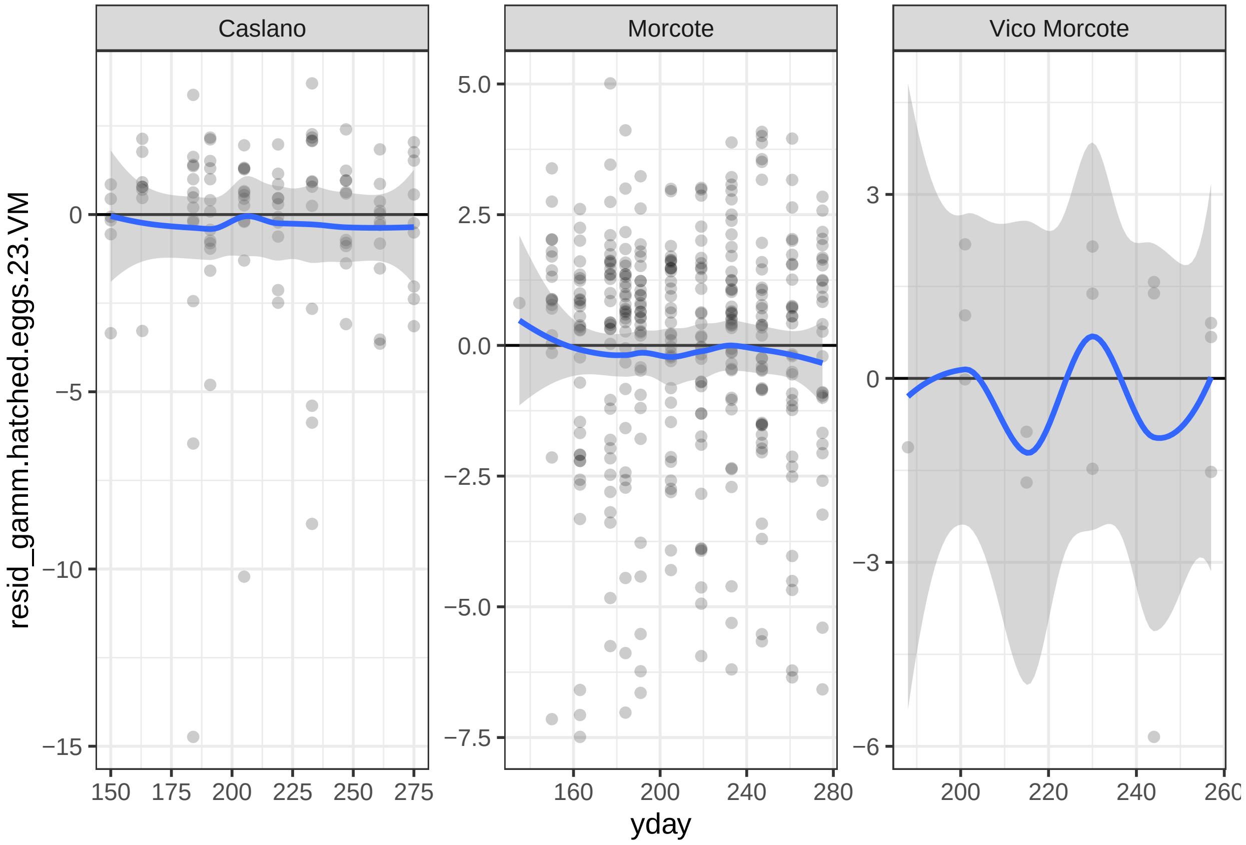

The blue lines are plotted on the x-axis, as desired.

The following two plots display the residuals in Morcote and Caslano, respectively.
The observations belonging to the same ovitrap are connected with a line. These plots are additionally drawn to check temporal correlation and variability.

```
## for Morcote
ggplot(data = filter(d.ovitraps.23.mod,
                     municipality.fac == "Morcote"),
       mapping = aes(y = resid_gamm.hatched.eggs.23.VM,
                     x = yday,
                     group = unique.ID)) +
  geom_hline(yintercept = 0) +
  geom_point(alpha = 0.2) +
  geom_line(alpha = 0.2)
```

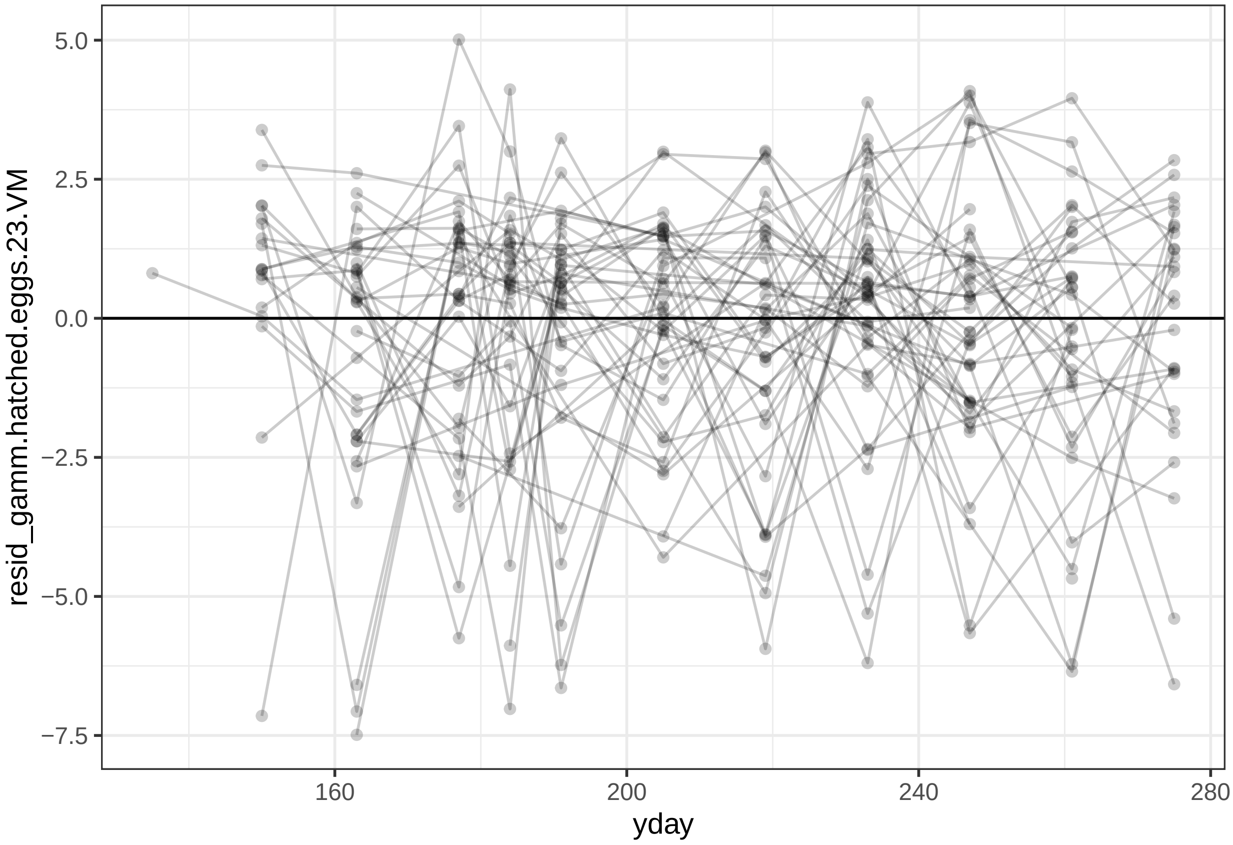


```
##
## for Caslano
ggplot(data = filter(d.ovitraps.23.mod,
                     municipality.fac == "Caslano"),
       mapping = aes(y = resid_gamm.hatched.eggs.23.VM,
                     x = yday,
                     group = unique.ID)) +
  geom_hline(yintercept = 0) +
  geom_point(alpha = 0.2) +
  geom_line(alpha = 0.2)
```

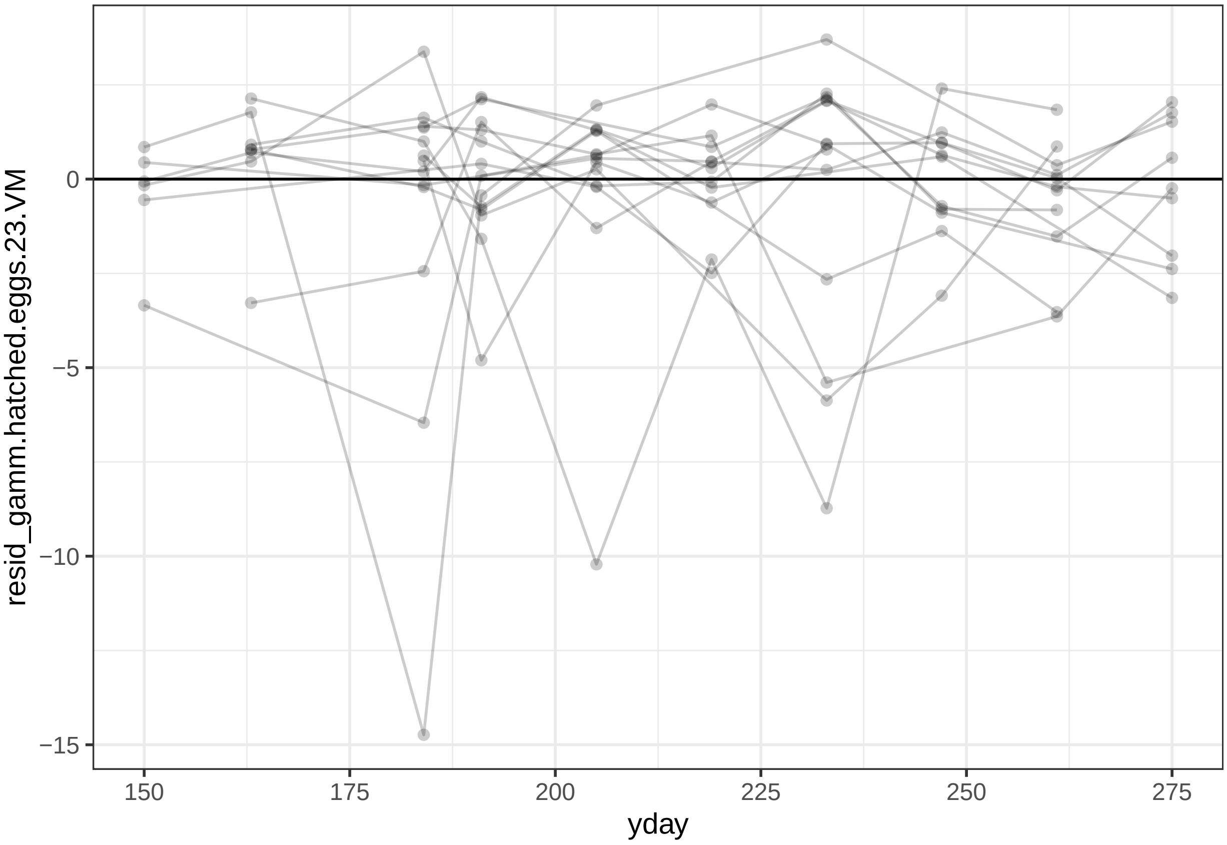

There seems to be some relevant variability between ovitraps.

Let's look at the single ovitraps.

```
## for Morcote
ggplot(data = filter(d.ovitraps.23.mod,
                     municipality.fac == "Morcote"),
       mapping = aes(y = resid_gamm.hatched.eggs.23.VM,
                     x = yday,
                     group = unique.ID)) +
  geom_hline(yintercept = 0) +
  geom_point() +
  geom_line() +
  facet_wrap(~unique.ID) +
  theme(
    strip.background = element_blank(),
    strip.text.x = element_blank()) +
  coord_cartesian(ylim = c(-7.5, 3.5))
```

```
`geom_line()`: Each group consists of only one observation.
i Do you need to adjust the group aesthetic?
```

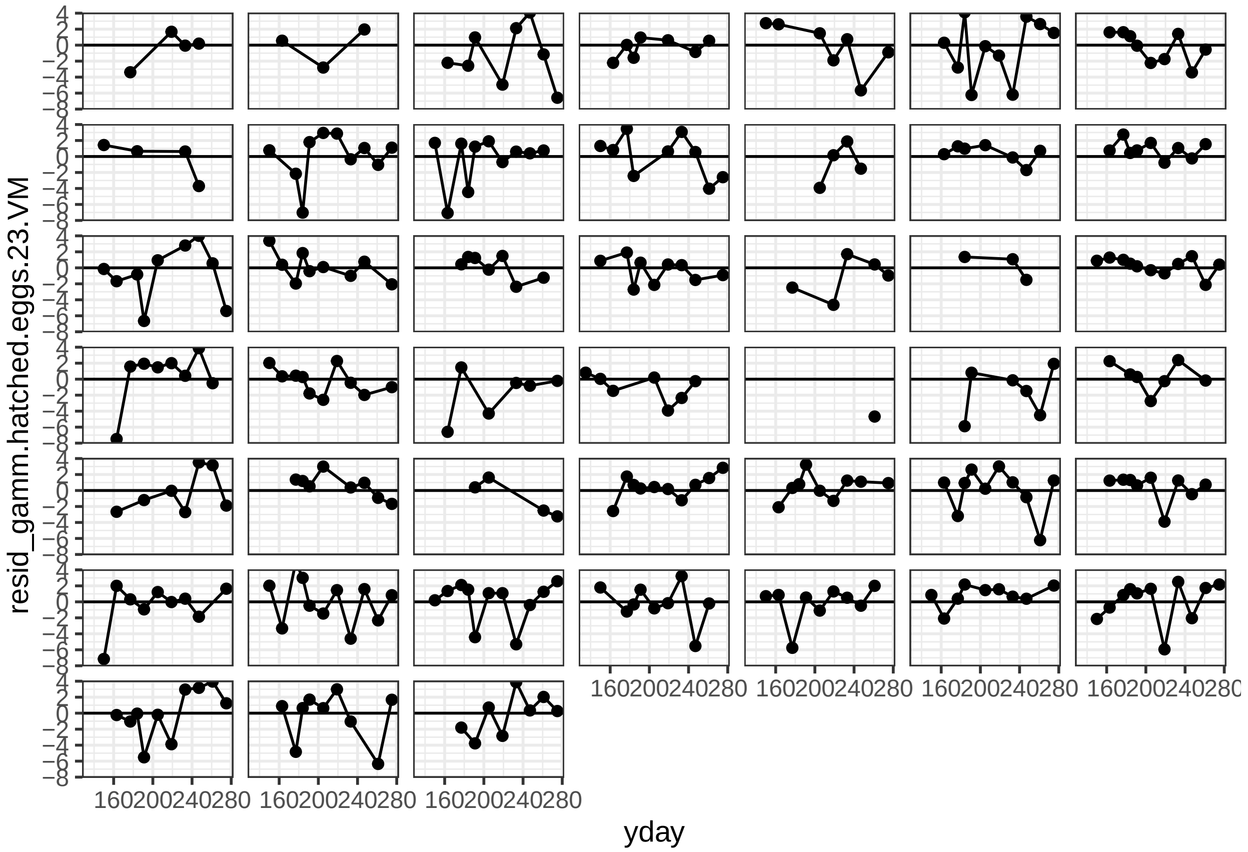


```r
##
## for Caslano
ggplot(data = filter(d.ovitraps.23.mod,
                     municipality.fac == "Caslano"),
       mapping = aes(y = resid_gamm.hatched.eggs.23.VM,
                     x = yday,
                     group = unique.ID)) +
  geom_hline(yintercept = 0) +
  geom_point() +
  geom_line() +
  facet_wrap(~unique.ID, scales = "free_y") +
  theme(
    strip.background = element_blank(),
    strip.text.x = element_blank()) +
  coord_cartesian(ylim = c(-7.5, 3.5))
```

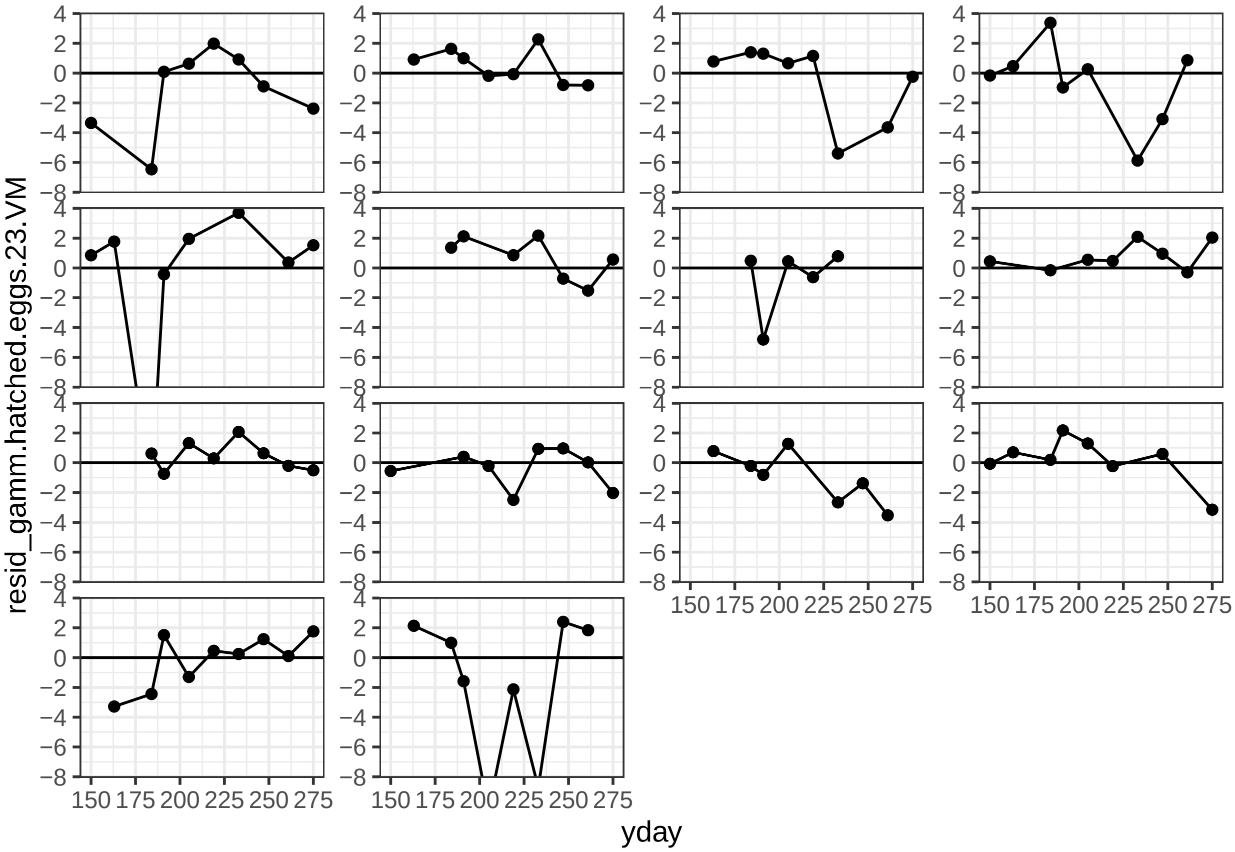

In both cases, there does not seem to be much structure left in the data.

If one were to do a formal test, there would probably be a temporal correlation. However, in this case we are more interested in modelling and understanding the effect rather than making a perfect inference, so the temporal correlation does not particularly affect our analysis.

Note that we set the limits at -7.5 and 3.5 to zoom in on the graphs, especially in the case of Caslano, where some residuals jump down to -15.

In general, there seems to be a larger variability in Caslano.

12.2 Spatial Generalised Additive Models

12.2.1 Visualising the data

We visualise these rates over space for Morcote and Vico Morcote.

```
## We calculate the min and max for the median number of eggs.
## This is used in the plots, in fact it allows us
## to use the same colour scale for the two
## municipalities and compare them more easily.
min.hatched.eggs <- min(d.agg.hatch.proc$mean.hatched)
max.hatched.eggs <- max(d.agg.hatch.proc$mean.hatched)
##
ggplot(filter(d.agg.hatch.proc,
              municipality.fac %in% c("Morcote", "Vico Morcote")),
       mapping = aes(y = Y.num,
                     x = X.num,
```

```
                          colour = mean.hatched)) +
  geom_point(size = 3) +
  scale_color_gradientn(colours = c("blue", "purple", "red"),
                        values = scales::rescale(c(min.hatched.eggs,
                                                   max.hatched.eggs)),
                        limits = c(min.hatched.eggs, max.hatched.eggs)) +
  coord_fixed()
```

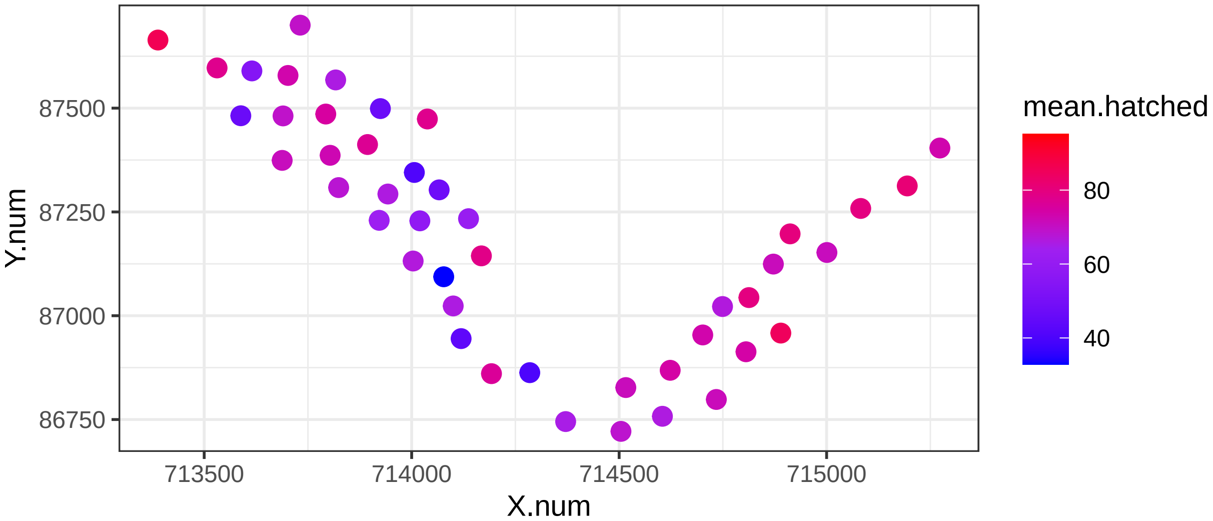

We can observe that there is a strong geographical pattern between the average number of eggs hatched in Morcote; in fact, the right-hand side has a higher average and less variability than the left-hand side.

We also plot the release points:

```
d.release.points.morcote <-
  read_excel("../Original_data/Coordinate_Release_points.xlsx")
head(d.release.points.morcote)
```

```
# A tibble: 6 x 4
  Municipality Release_point LV03E      LV03N
  <chr>                <dbl> <chr>      <chr>
1 Morcote                  1 715'380.43 87'541.29
2 Morcote                  2 715'324.64 87'466.31
3 Morcote                  3 715'265.00 87'393.70
4 Morcote                  4 715'208.52 87'319.81
5 Morcote                  5 715'129.26 87'268.15
6 Morcote                  6 715'059.20 87'201.06
```

```
str(d.release.points.morcote)
```

```
tibble [75 x 4] (S3: tbl_df/tbl/data.frame)
 $ Municipality : chr [1:75] "Morcote" "Morcote" "Morcote" "Morcote" ...
 $ Release_point: num [1:75] 1 2 3 4 5 6 7 8 9 10 ...
 $ LV03E        : chr [1:75] "715'380.43" "715'324.64" "715'265.00" "715'208.52" ...
 $ LV03N        : chr [1:75] "87'541.29" "87'466.31" "87'393.70" "87'319.81" ...
```

```
d.release.points.morcote <- d.release.points.morcote %>%
  mutate(LV03_E = as.numeric(gsub("'", "", LV03E)),
         LV03_N = as.numeric(gsub("'", "", LV03N)))
str(d.release.points.morcote)
```

```
tibble [75 x 6] (S3: tbl_df/tbl/data.frame)
 $ Municipality : chr [1:75] "Morcote" "Morcote" "Morcote" "Morcote" ...
 $ Release_point: num [1:75] 1 2 3 4 5 6 7 8 9 10 ...
 $ LV03E        : chr [1:75] "715'380.43" "715'324.64" "715'265.00" "715'208.52" ...
 $ LV03N        : chr [1:75] "87'541.29" "87'466.31" "87'393.70" "87'319.81" ...
 $ LV03_E       : num [1:75] 715380 715325 715265 715209 715129 ...
 $ LV03_N       : num [1:75] 87541 87466 87394 87320 87268 ...
```

```r
## Plot Morcote
ggplot(filter(d.agg.hatch.proc,
              municipality.fac %in% c("Morcote", "Vico Morcote")),
       mapping = aes(y = Y.num,
                     x = X.num,
                     colour = mean.hatched)) +
  geom_point(size = 3) +
  scale_color_gradientn(colours = c("blue", "purple", "red"),
                        values = scales::rescale(c(min.hatched.eggs,
                                                   max.hatched.eggs)),
                        limits = c(min.hatched.eggs, max.hatched.eggs)) +
  geom_point(data = d.release.points.morcote,
             mapping = aes(x = LV03_E, y = LV03_N),
             colour = "black", size = 1, pch = 4) +
  coord_fixed()
```

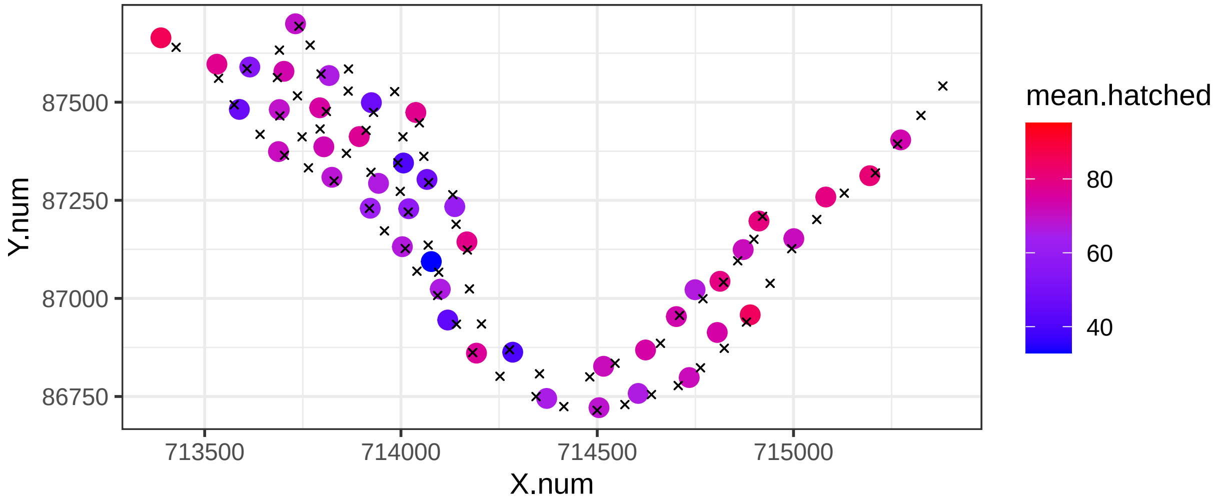

12.2.2 Fitting the models

We aim to model the percentage of hatched *Aedes albopictus* eggs over the season (*yday*), while accounting for geographical differences.

The response variable is binomial, representing the counts of hatched versus non-hatched eggs (quantified by the variables *hatched.albo.eggs.after.proc* and *non.hatched.albo.eggs.after.proc*). To account for overdispersion, the quasi-binomial family is used for formal modelling.

yday will be included as numeric variable; the same holds true for *X.num* and *Y.num*, which represent the swiss coordinates.

We begin without assuming any specific seasonal pattern, so we will fit a Generalised Additive Model (GAM), which provides flexibility in determining the best shape for the seasonal trend. However, this flexibility comes at the cost of interpretability.

Note that we use the element `s(X.num, Y.num)` to plot spatial data. We can use this isotropic smoothing because both predictors are spatial coordinates and we assume the spatial effect is istropic (i.e., the same in all directions) and the two variables are on the same scale.

We start by fitting the model for Morcote and Vico Morcote.

```
d.ovitraps.23.morcote.VM <- filter(
  d.ovitraps.23.mod,
  municipality.fac %in% c("Morcote",
                          "Vico Morcote"))
##
## GAM for Morcote and Vico Morcote
gam.hatch_space.Morcote.VM <- gamV(
  cbind(hatched, non.hatched) ~
    s(yday, pc = 182) +
    s(X.num, Y.num), ## new element!
  family = "quasibinomial",
  data = d.ovitraps.23.morcote.VM)
summary(gam.hatch_space.Morcote.VM)
```

```

Family: quasibinomial
Link function: logit

Formula:
cbind(hatched, non.hatched) ~ s(yday, pc = 182) + s(X.num, Y.num)

Parametric coefficients:
            Estimate Std. Error t value Pr(>|t|)
(Intercept)  0.95324    0.08939   10.66   <2e-16 ***
---
Signif. codes:  0 '***' 0.001 '**' 0.01 '*' 0.05 '.' 0.1 ' ' 1

Approximate significance of smooth terms:
                 edf Ref.df     F  p-value
s(yday)        3.661  4.515 3.872  0.00303 **
s(X.num,Y.num) 6.689  9.188 5.287 9.82e-07 ***
---
Signif. codes:  0 '***' 0.001 '**' 0.01 '*' 0.05 '.' 0.1 ' ' 1

R-sq.(adj) =  0.134   Deviance explained = 17.4%
-REML = -28.249  Scale est. = 6.0875    n = 367
```

We refit the same model for Caslano. Let's see how large k can be for the bi-dimensional smoother.

```
d.ovitraps.23.mod %>%
  filter(municipality.fac == "Caslano") %>%
  select(X.num, Y.num) %>%
  unique() %>%
  nrow()
```

```
[1] 14
```

12.2.3 Plotting the model

We now plot the results.

12.2.4 Morcote and Vico Morcote

We start with visualising the model fitted for Morcote and Vico Morcote.

```
plot.gam(gam.hatch_space.Morcote.VM,
         select = 1,
         trans = plogis,
         rug = TRUE)
```

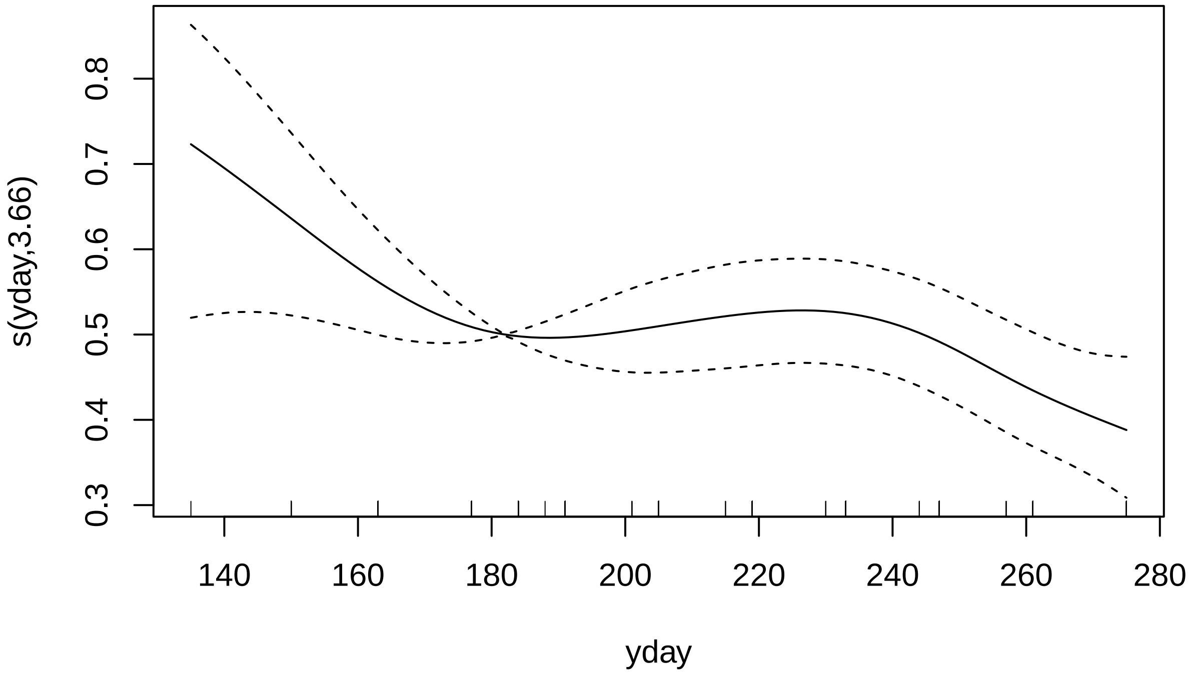


```
##
plot.gamViz(gam.hatch_space.Morcote.VM,
            select = 2,
            trans = plogis,
            rug = TRUE) +
  coord_fixed()
```

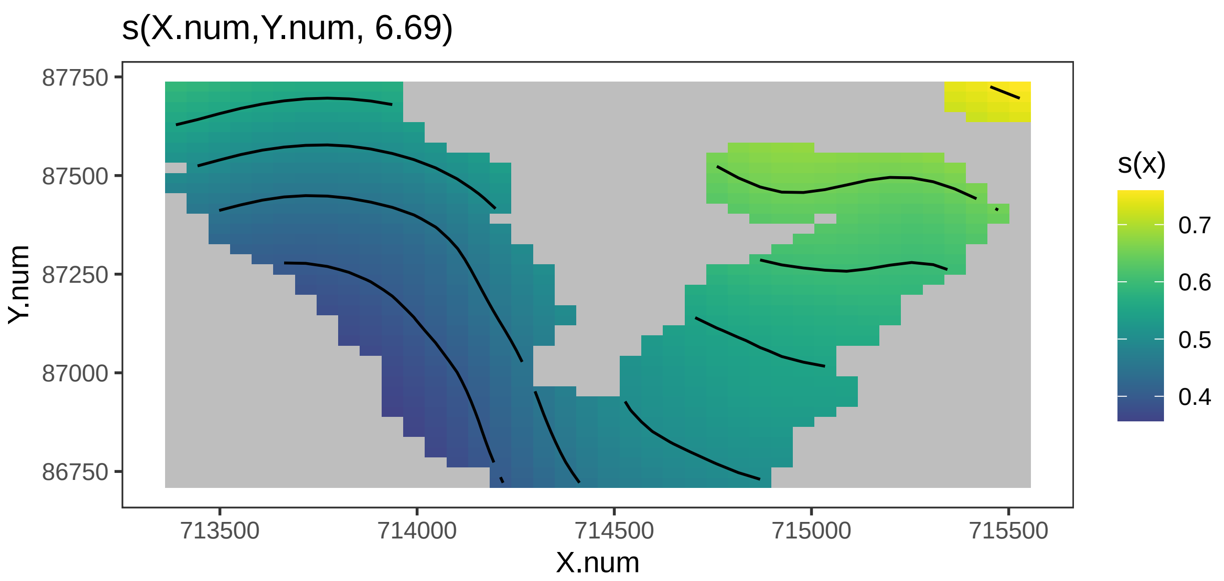

The percentage of hatched eggs is higher on the right-hand side of Morcote (which also includes Vico Morcote).

12.2.5 Residual analysis

First of all, we apply the `gam.check()` function to the models, which produces some diagnostic information.

```
## Morcote
par(mfrow = c(2, 2))
gam.check(gam.hatch_space.Morcote.VM)
```

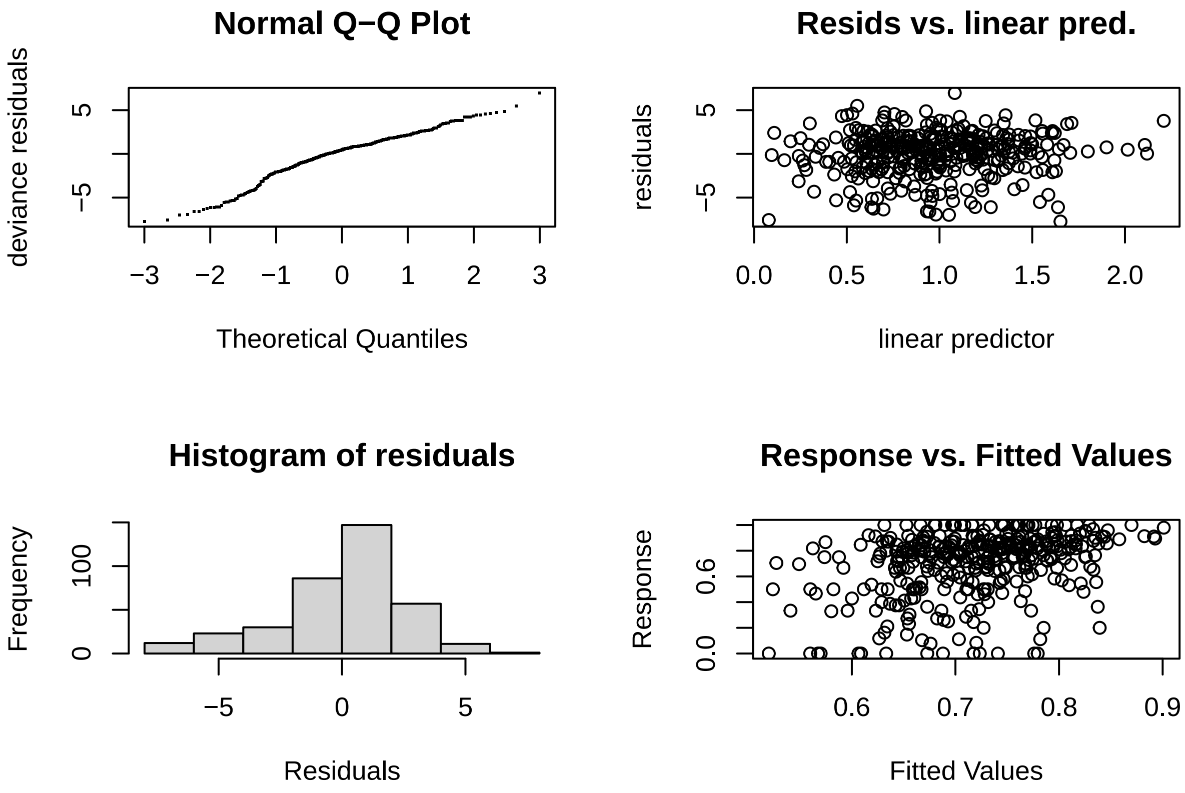

```
Method: REML   Optimizer: outer newton
full convergence after 6 iterations.
Gradient range [-3.89731e-06,2.337878e-06]
(score -28.24869 & scale 6.087471).
Hessian positive definite, eigenvalue range [0.7146155,181.5388].
Model rank =  39 / 39

Basis dimension (k) checking results. Low p-value (k-index<1) may
indicate that k is too low, especially if edf is close to k'.

                   k'   edf k-index p-value
s(yday)          9.00  3.66    1.06    0.90
s(X.num,Y.num) 29.00  6.69    0.99    0.42
```

```r
par(mfrow = c(1, 1))
##
## Caslano
par(mfrow = c(2, 2))
gam.check(gam.hatch_space.Caslano)
```

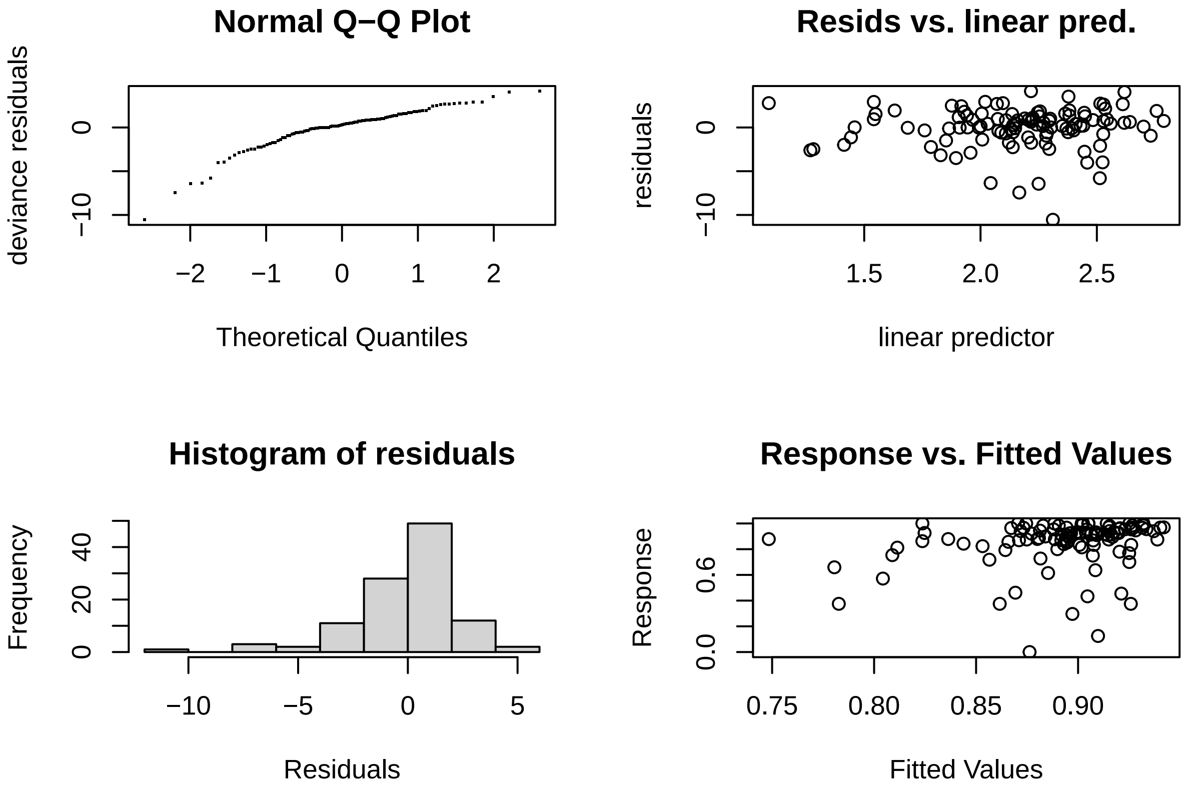


```
Method: REML   Optimizer: outer newton
full convergence after 12 iterations.
Gradient range [-1.790304e-05,2.356962e-05]
(score -40.86731 & scale 6.243991).
Hessian positive definite, eigenvalue range [1.790201e-05,52.01665].
Model rank =  23 / 23

Basis dimension (k) checking results. Low p-value (k-index<1) may
indicate that k is too low, especially if edf is close to k'.
```

```
                    k'    edf k-index p-value
s(yday)           9.00  2.86    1.22    1.00
s(X.num,Y.num) 13.00  2.00    1.16    0.92
```

```
par(mfrow = c(1, 1))
```

Then, we store the pearson residuals in the original data frame, and we plot the residuals against the fitted values to see whether there is still structure in the data.

```
## Morcote
d.ovitraps.23.morcote.VM$resid_gam.hatch_space.Morcote.VM <-
  resid(gam.hatch_space.Morcote.VM,
        type = "pearson")
##
d.ovitraps.23.morcote.VM$fitted_gam.hatch_space.Morcote.VM <-
  fitted(gam.hatch_space.Morcote.VM)
##
ggplot(data = d.ovitraps.23.morcote.VM,
       mapping = aes(y = resid_gam.hatch_space.Morcote.VM,
                     x = fitted_gam.hatch_space.Morcote.VM)) +
  geom_hline(yintercept = 0) +
  geom_point(alpha = 0.2) +
  geom_smooth()
```

```
`geom_smooth()` using method = 'loess' and formula = 'y ~ x'
```

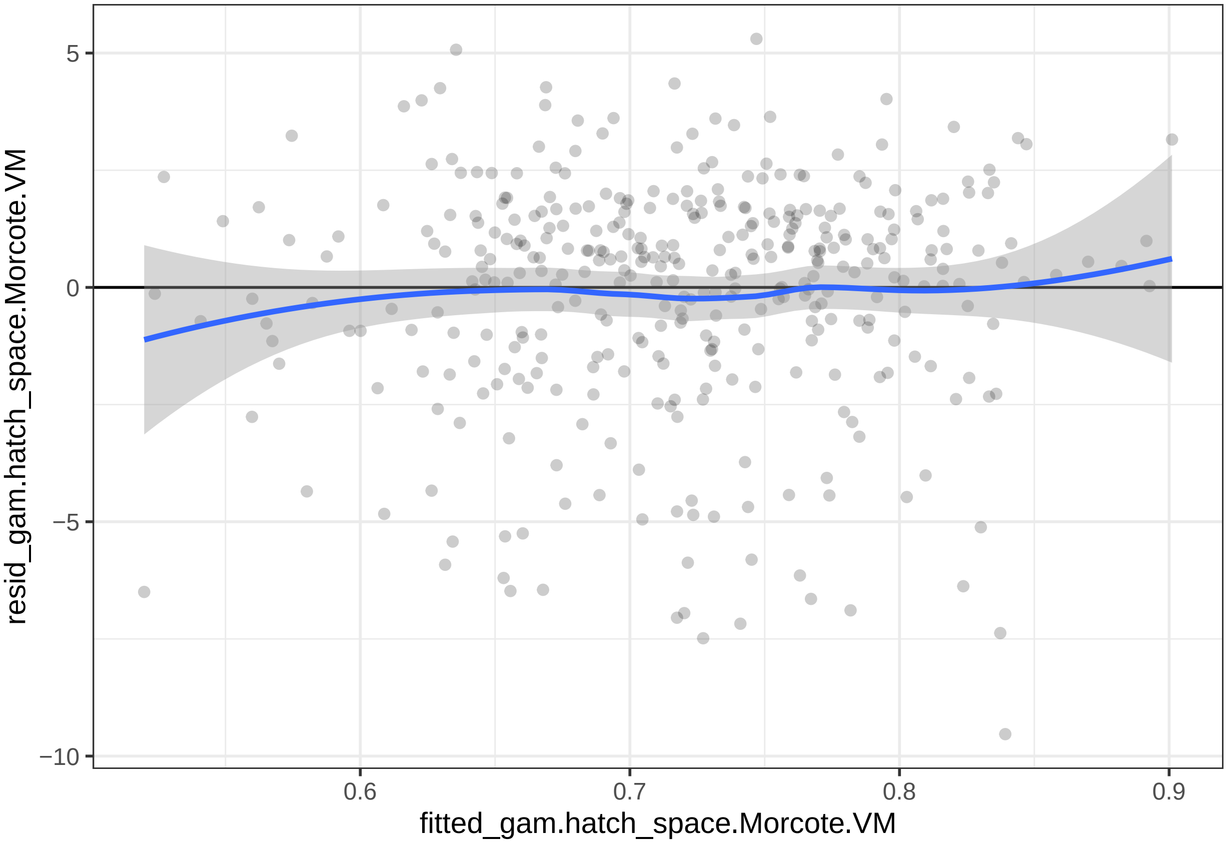


```
##
## Caslano
```

```r
d.ovitraps.23.caslano$resid_gam.hatch_space.Caslano <- resid(gam.hatch_space.Caslano,
                                                             type = "pearson")
##
##
d.ovitraps.23.caslano$fitted_gam.hatch_space.Caslano <- fitted(gam.hatch_space.Caslano)
##
ggplot(data = d.ovitraps.23.caslano,
       mapping = aes(y = resid_gam.hatch_space.Caslano,
                     x = fitted_gam.hatch_space.Caslano)) +
  geom_hline(yintercept = 0) +
  geom_point(alpha = 0.2) +
  geom_smooth()
```

```
`geom_smooth()` using method = 'loess' and formula = 'y ~ x'
```

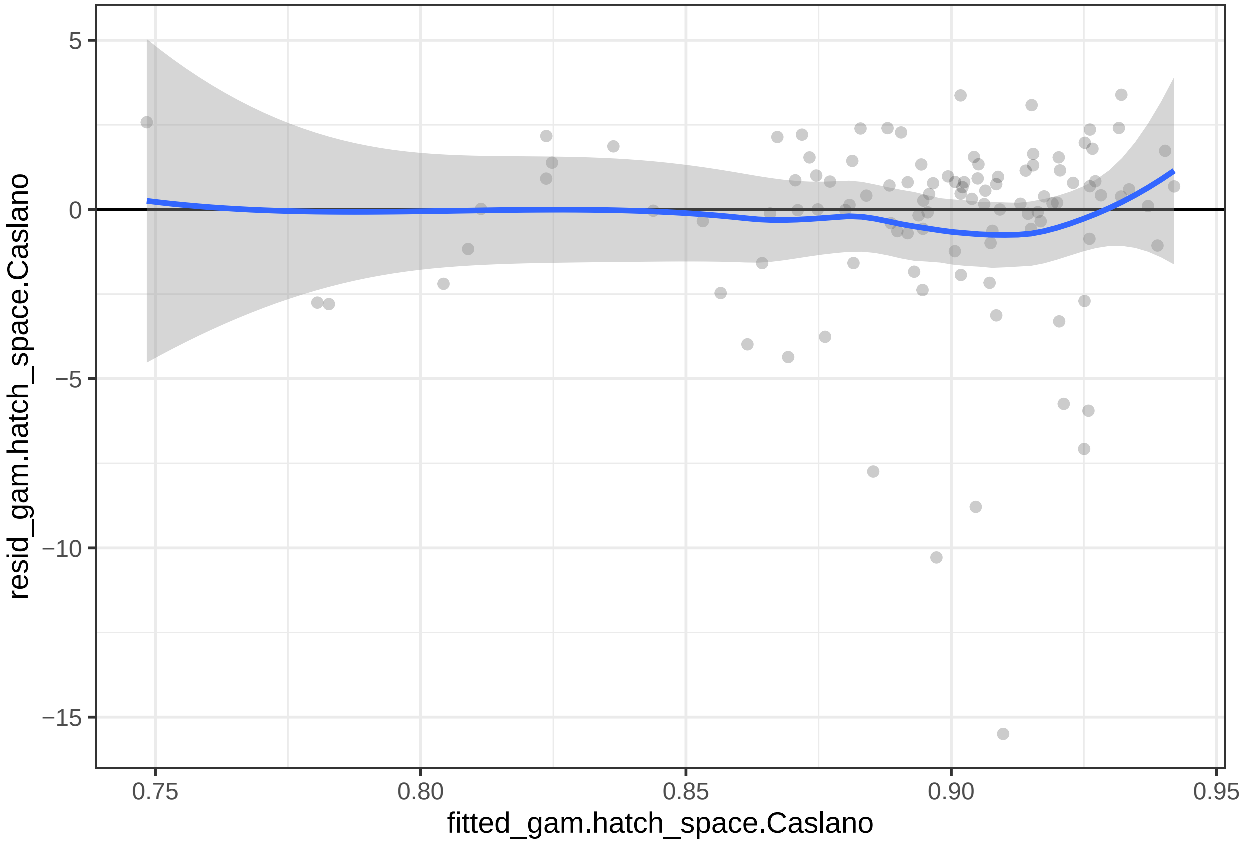

The blue lines are located on the x-axis, indicating that there doesn't seem to be structure left in the residuals.

12.2.6 Comparing (over)dispersion in the two models

We compare again the overdispersion in the two models.

As a reminder, in a quasi binomial model the parameter θ is estimated to be such that:

$$var(y) = \theta \times \mu(1-\mu), \quad \text{where} \quad \mu = \mathbb{E}(y)$$

.

Let's verify which model has highest θ, i.e., highest overdispersion.

```r
summary(gam.hatch_space.Morcote.VM)$dispersion
```

```
[1] 6.087471
```

```r
summary(gam.hatch_space.Caslano)$dispersion
```

```
[1] 6.243991
```

Caslano has the highest overdispersion between the two municipalities; however the difference is tiny.
