# Supplementary material for: Effectiveness of the sterile insect technique in controlling Aedes albopictus as part of an integrated control measure: evidence from a first small-scale field trial in Switzerland
Source: Infect Dis Poverty. 2025 Aug 22;14:90. doi: 10.1186/s40249-025-01360-2 (PMC12372186; doi:10.1186/s40249-025-01360-2)
Supplement: Supplementary file 4 — Supplementary Material 4. Analysis number of Ae. albopictus adult females. [file 40249_2025_1360_MOESM4_ESM.pdf]

Modelling: Paper Morcote Project

Number of adults

Author: Nisia Trisconi & Dr. Matteo Tanadini | Zurich Data Scientists
Reviewer: Dr. Luisa Barbanti | Zurich Data Scientists

February 3, 2025

Contents

1 Freeze Package versions 3

2 Settings 3

3 Getting data 4

4 Aim and design 5

5 Generalised Additive Mixed-Effects Model – Females (GAMM) 5
- 5.1 Visualising the data . . . 5
- 5.2 Fitting the model . . . 10
- 5.3 Plotting the model . . . 13
- 5.4 Fitted values . . . 16
- 5.5 Predicted values . . . 17
- 5.6 Model selection . . . 20
  - 5.6.1 Shape . . . 20
  - 5.6.2 Shift . . . 23
- 5.7 Residual analysis . . . 24
- 5.8 Comparing (over)dispersion in the two models . . . 32

6 Spatial Generalised Additive Model – Females (spatial GAM) 35
- 6.1 Visualising the data . . . 35
- 6.2 Fitting the models . . . 37
- 6.3 Plotting the models . . . 40
  - 6.3.1 Morcote . . . 40
  - 6.3.2 Caslano . . . 41
- 6.4 Comparing (over)dispersion in the two models . . . 43

7 Spatial Generalised Additive Model over time – Females 44
- 7.1 Plotting the model . . . 45
- 7.2 Comparison between models . . . 48

8 Spatial Generalised Additive Model – Males (spatial GAM) 50
- 8.1 Visualising the data . . . 50
- 8.2 Fitting the models . . . 52
- 8.3 Plotting the smoothers . . . 55
  - 8.3.1 Morcote . . . 55
  - 8.3.2 Caslano . . . 56

8.4 Comparing (over)dispersion in the two models . . . 57

9 Spatial Generalised Additive Model over time – Males **58**
9.1 Plotting the models . . . 59
9.1.1 Morcote . . . 59
9.1.2 Caslano . . . 61

10 Methods **63**
10.1 Generalised Additive Mixed-Effects Model – Females (GAMM) . . . 63
10.2 Spatial Generalised Additive Model – Females (spatial GAM) . . . 63
10.3 Spatial Generalised Additive Model – Males (spatial GAM) . . . 64

11 Results **64**
11.1 Generalised Additive Mixed-Effects Model – Females (GAMM) . . . 64
11.2 Spatial Generalised Additive Model – Females (spatial GAM) . . . 64
11.3 Spatial Generalised Additive Model – Males (spatial GAM) . . . 65

12 Conclusions **65**

13 References **65**

14 Appendix **66**

1 Freeze Package versions

```
## (messages are omitted from this chunk)
##
library(groundhog)
pkgs <- c("dplyr",
          "ggplot2",
          "lubridate",
          "mgcViz")
groundhog.library(pkgs, date = "2024-11-01")
```

2 Settings

Global settings:

```
Sys.setenv(lang = "en_US")
theme_set(theme_bw())

if (!dir.exists("Prepared_data_and_models")) {
  dir.create("Prepared_data_and_models")
}
```

3 Getting data

```
## temporal analysis
d.ovitraps <- readRDS(file = paste0("Prepared_data_and_models/",
                                    "d.adults_PreparedData.RDS"))
```

For this file, we only consider the year 2023. So we define a reduced data set:

```
d.ovitraps.23 <- d.ovitraps %>%
  filter(Year == 2023)

## check
d.ovitraps.23 %>%
  select(Year) %>%
  unique()
```

```
# A tibble: 1 x 1
   Year
  <dbl>
1  2023
```

Overview of the data:

```
dim(d.ovitraps.23)
```

```
[1] 396  29
```

```
head(d.ovitraps.23)[1:min(ncol(d.ovitraps.23), 30)]
```

```
# A tibble: 6 x 29
  BG.round Activation.time Ae.albopictus.female Ae.albopictus.male
     <dbl>           <dbl>                <dbl>              <dbl>
1        1               1                   NA                 NA
2        1               1                   NA                 NA
3        1               1                   NA                 NA
4        1               1                   NA                 NA
5        1               1                   NA                 NA
6        1               1                   NA                 NA
# i 25 more variables: Cx.pipiens.female <dbl>, Cx.pipiens.male <dbl>,
#   Ae.japonicus.female <dbl>, Ae.japonicus.male <dbl>,
#   Ae.koreicus.female <dbl>, Ae.koreicus.male <dbl>, Year <dbl>, X.num <dbl>,
#   Y.num <dbl>, BG_round.fac <fct>, Setting_date.date <date>,
#   Sampling_date.date <date>, municipality.fac <fct>, municipality.ord <ord>,
#   ID_BG_trap.fac <fct>, status.fac <fct>, Year.fac <fct>,
#   Ae.albopictus.tot <dbl>, Ae.albopictus.sex.ratio <dbl>, ...
```

```
str(d.ovitraps.23)
```

```
tibble [396 x 29] (S3: tbl_df/tbl/data.frame)
 $ BG.round                : num [1:396] 1 1 1 1 1 1 1 1 1 1 ...
 $ Activation.time         : num [1:396] 1 1 1 1 1 1 1 1 1 1 ...
 $ Ae.albopictus.female    : num [1:396] NA NA NA NA NA NA NA NA NA NA ...
 $ Ae.albopictus.male      : num [1:396] NA NA NA NA NA NA NA NA NA NA ...
 $ Cx.pipiens.female       : num [1:396] NA NA NA NA NA NA NA NA NA NA ...
 $ Cx.pipiens.male         : num [1:396] NA NA NA NA NA NA NA NA NA NA ...
 $ Ae.japonicus.female     : num [1:396] NA NA NA NA NA NA NA NA NA NA ...
 $ Ae.japonicus.male       : num [1:396] NA NA NA NA NA NA NA NA NA NA ...
```

```
 $ Ae.koreicus.female     : num [1:396] NA NA NA NA NA NA NA NA NA NA ...
 $ Ae.koreicus.male       : num [1:396] NA NA NA NA NA NA NA NA NA NA ...
 $ Year                   : num [1:396] 2023 2023 2023 2023 2023 ...
 $ X.num                  : num [1:396] 714887 714803 714705 714545 715235 ...
 $ Y.num                  : num [1:396] 87150 87018 86965 86840 87338 ...
 $ BG_round.fac           : Factor w/ 12 levels "1","2","3","4",..: 1 1 1 1 1 1 1 1 1 1 ...
 $ Setting_date.date      : Date[1:396], format: "2023-05-24" "2023-05-24" ...
 $ Sampling_date.date     : Date[1:396], format: "2023-05-25" "2023-05-25" ...
 $ municipality.fac       : Factor w/ 3 levels "buffer area",..: 3 3 3 3 3 3 3 3 3 3 ...
 $ municipality.ord       : Ord.factor w/ 3 levels "buffer area"<..: 3 3 3 3 3 3 3 3 3 3 ...
 $ ID_BG_trap.fac         : Factor w/ 41 levels "1","10","11",..: 1 12 23 28 29 30 31 32 33 2 ...
 $ status.fac             : Factor w/ 3 levels "A","F","M": 3 3 3 3 3 3 3 3 3 3 ...
 $ Year.fac               : Factor w/ 2 levels "2023","2024": 1 1 1 1 1 1 1 1 1 1 ...
 $ Ae.albopictus.tot      : num [1:396] NA NA NA NA NA NA NA NA NA NA ...
 $ Ae.albopictus.sex.ratio: num [1:396] NA NA NA NA NA NA NA NA NA NA ...
 $ Ae.albopictus.male.prop: num [1:396] NA NA NA NA NA NA NA NA NA NA ...
 $ yday                   : num [1:396] 145 145 145 145 145 145 145 145 145 145 ...
 $ week                   : num [1:396] 21 21 21 21 21 21 21 21 21 21 ...
 $ unique.ID              : Factor w/ 41 levels "Morcote.1","Morcote.10",..: 1 12 23 28 29 30 31 32 33
 $ unique.ID.Year         : Factor w/ 74 levels "Morcote.1.2023",..: 1 12 23 28 29 30 31 32 33 2 ...
 $ super.municipality.year: Factor w/ 6 levels "buffer area:2023",..: 3 3 3 3 3 3 3 3 3 3 ...
```

4 Aim and design

In 2023, SUPSI received approval from the FOEN to release sterile male tiger mosquitoes in the municipality of Morcote as part of a field experiment aimed at reducing the number of eggs laid by female mosquitoes.

The experiment involved weekly releases of sterile male *Aedes albopictus* mosquitoes in Morcote during the active mosquito season, from May to September 2023. Approximately 3,000 sterile males per hectare were released each week through 75 predefined stations spaced 50 to 80 meters apart.

Ovitraps were sampled multiple times over time in Morcote (treated) and Caslano (untreated) to assess whether the release of sterile males had an impact on the number of Ae albopictus adults.

In total, the data was collected on `12` different dates. Both municipalities were sampled `12` times.

5 Generalised Additive Mixed-Effects Model – Females (GAMM)

5.1 Visualising the data

The purpose of this analysis is to determine whether the release of sterilised males in a given area helps reduce the number of adult mosquitoes.

A large number of males were released by the researchers, so we cannot include the male count in the analysis when assessing the reduction in mosquito populations, as this would lead to inaccurate results. Therefore, only the number of females is monitored to evaluate whether the procedure is effective.

With this purpose in mind, we begin by displaying the number of Aa albopictus females found in the traps sampled throughout the season. As we are dealing with count data, we apply a square-root transformation to the response variable *Ae.albopictus.female* to stabilise variance.

Furthermore, observations from the same ovitrap are connected with a line to visualise trends over time. On top of these lines, we add a smoothed average that allows us to see the global trend.

```
## (messages and warnings are excluded from this chunk)
##
```

```r
p <- ggplot(data = d.ovitraps.23,
            mapping = aes(y = `Ae.albopictus.female`,
                          x = Sampling_date.date,
                          group = ID_BG_trap.fac)) +
  geom_hline(yintercept = 0) +
  geom_point(alpha = 0.2) +
  geom_line(alpha = 0.2) +
  scale_y_sqrt() +
  geom_smooth(mapping = aes(group = NULL))
p
```

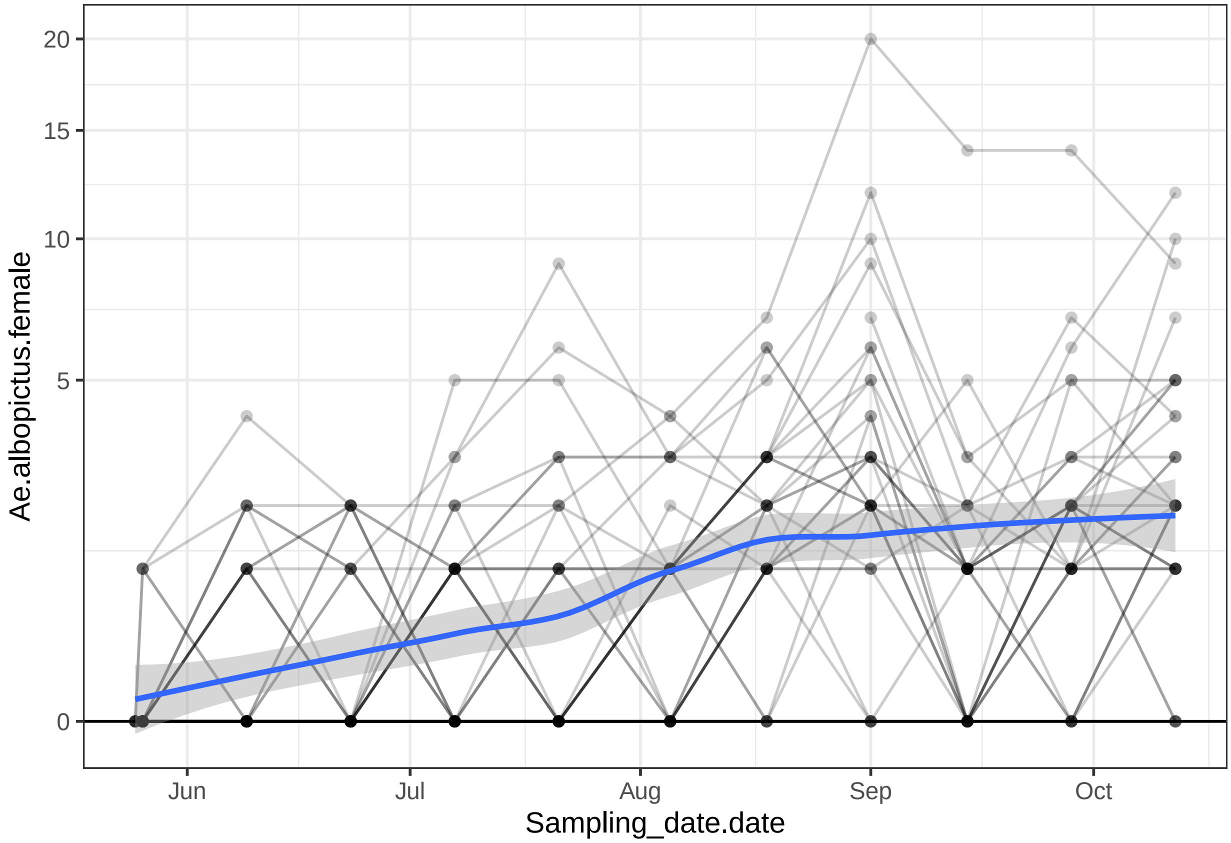


```r
## save plot for future use:
saveRDS(p, file = file.path("saved_figures",
                            "2c_femalesOverTime.rds"))
```

The number of females seems to constantly increase over the course of the season.

The sterile males were released only in Morcote, while Caslano served as control. For this reason, we now create a separate plot for each municipality to observe whether there are any differences between them.

```r
## (messages and warnings are excluded from this chunk)
##
p <- ggplot(data = d.ovitraps.23,
            mapping = aes(y = `Ae.albopictus.female`,
                          x = Sampling_date.date,
                          group = ID_BG_trap.fac)) +
  geom_hline(yintercept = 0) +
```

```
  geom_point(alpha = 0.2) +
  geom_line(alpha = 0.2) +
  scale_y_sqrt() +
  facet_wrap(~ municipality.fac) +
  geom_smooth(mapping = aes(group = 1),
              method = "loess")
p
```

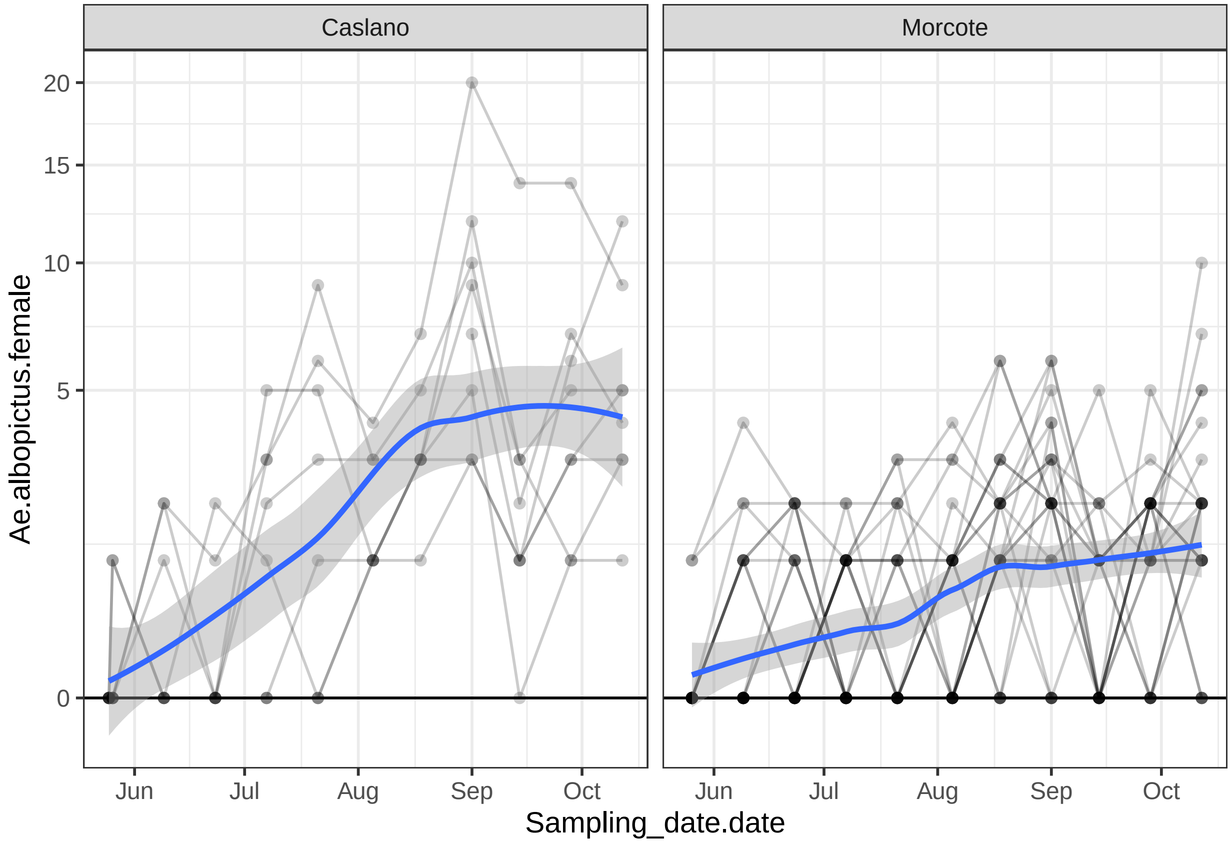


```
## save plot for future use:
saveRDS(p, file = file.path("saved_figures",
                            "2c_TotalfemalesMuniPanel.rds"))
```

Morcote seems to have, on average, fewer females than Caslano.

To facilitate comparison, rather than creating separate plots, we overlay the two municipalities on a single plot (colouring them with different colours) and add a smoother for each one.

```
## (messages and warnings are excluded from this chunk)
##
p <- ggplot(data = d.ovitraps.23,
            mapping = aes(y = `Ae.albopictus.female`,
                          x = Sampling_date.date,
                          group = ID_BG_trap.fac,
                          colour = municipality.fac)) +
  geom_hline(yintercept = 0) +
  geom_point(alpha = 0.1) +
  geom_line(alpha = 0.1) +
```

```
  scale_y_sqrt(limits = c(0,NA), ) +
  geom_smooth(mapping = aes(group = municipality.fac),
              method = "loess",
              alpha = 0.2)
p
```

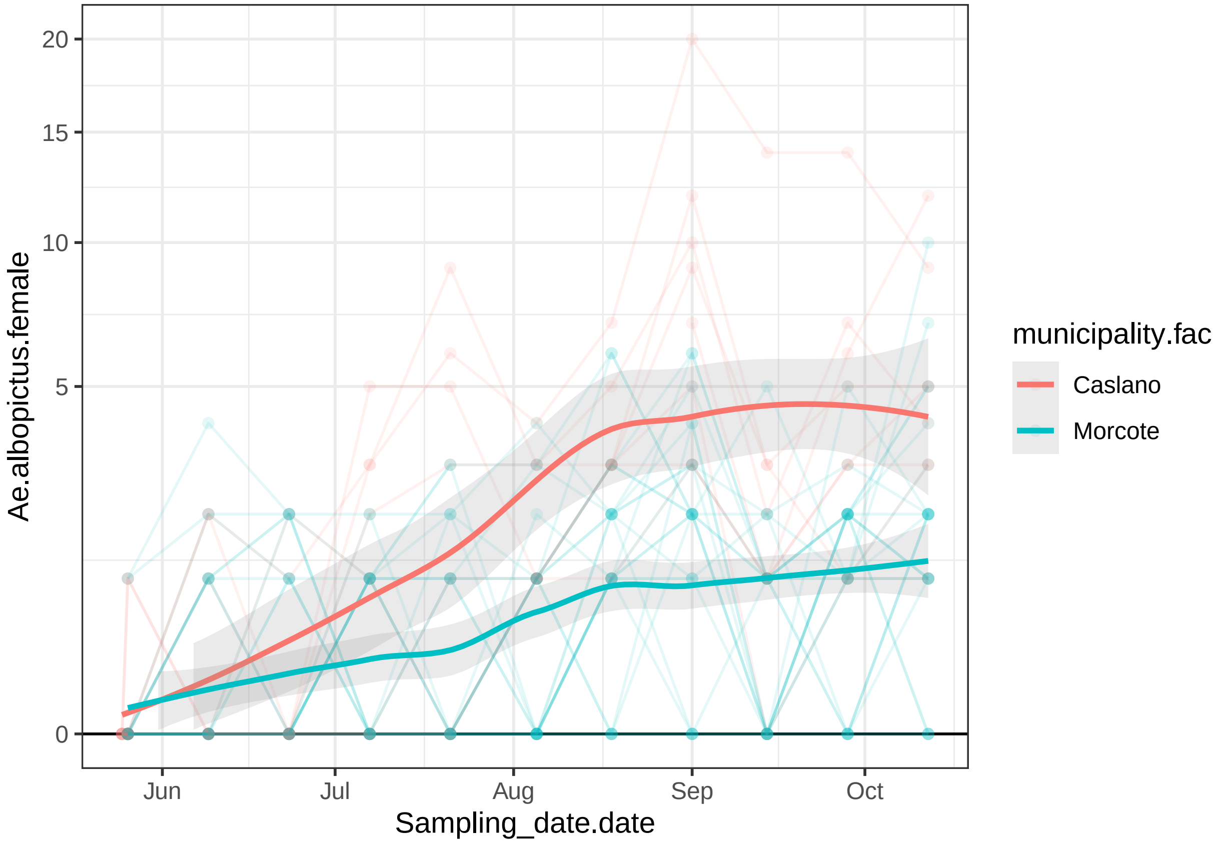


```
## save plot for future use:
saveRDS(p, file = file.path("saved_figures",
                            "2c_TotalfemalesMuniSmooth.rds"))
```

This graph confirms that the number of females in Morcote is lower than in Caslano for the 2023 season.

We now focus on Morcote to examine seasonal differences among the ovitraps. Since we are not concerned with the behaviour of individual ovitraps, we remove the labels.

```
## (messages and warnings are excluded from this chunk)
##
p <- ggplot(data = filter(d.ovitraps.23, municipality.fac == "Morcote"),
            mapping = aes(y = `Ae.albopictus.female`,
                          x = Sampling_date.date,
                          group = ID_BG_trap.fac)) +
  geom_hline(yintercept = 0) +
  geom_point() +
  geom_line() +
  scale_y_sqrt(limits = c(0, NA)) +
  facet_wrap(~ ID_BG_trap.fac) +
  theme(
```

```
    strip.background = element_blank(),
    strip.text.x = element_blank(),
    axis.text.x = element_text(angle = 90)) +
  labs(title = "Morcote")
p
```

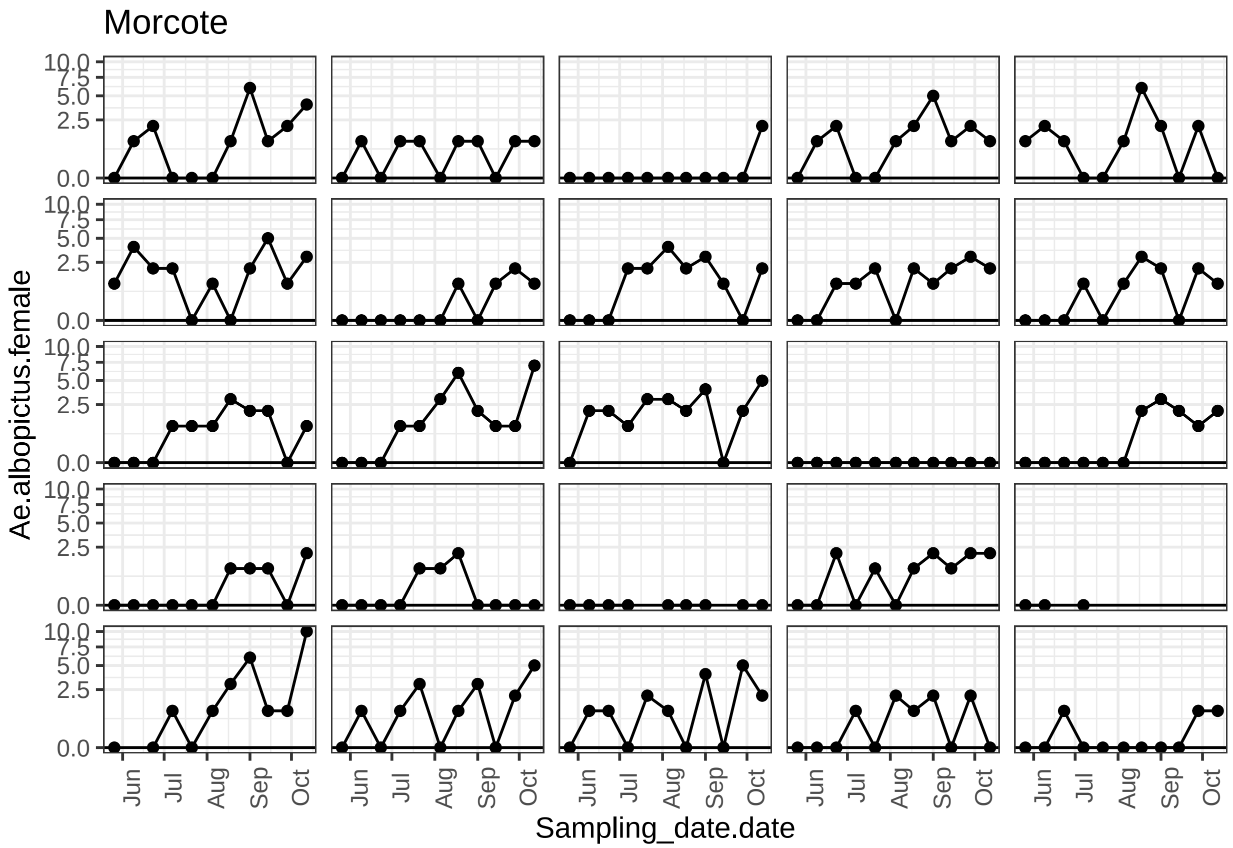


```
## save plot for future use:
saveRDS(p, file = file.path("saved_figures",
                            "2c_MorcotefemalesOverTimeNolabels.rds"))
```

There is some variation within and between ovitraps.

We create the same plot for Caslano to determine whether the observed behaviour is a result of the release of sterile males, or if it is a common pattern in the control municipalities as well.

```
## (messages and warnings are excluded from this chunk)
##
p <- ggplot(data = filter(d.ovitraps.23, municipality.fac == "Caslano"),
            mapping = aes(y = `Ae.albopictus.female`,
                          x = Sampling_date.date,
                          group = ID_BG_trap.fac)) +
  geom_hline(yintercept = 0) +
  geom_point() +
  geom_line() +
  scale_y_sqrt(limits = c(0,NA), ) +
  facet_wrap(~ ID_BG_trap.fac) +
  theme(
```

```
    strip.background = element_blank(),
    strip.text.x = element_blank(),
    axis.text.x = element_text(angle = 90)) +
  labs(title = "Caslano")
p
```

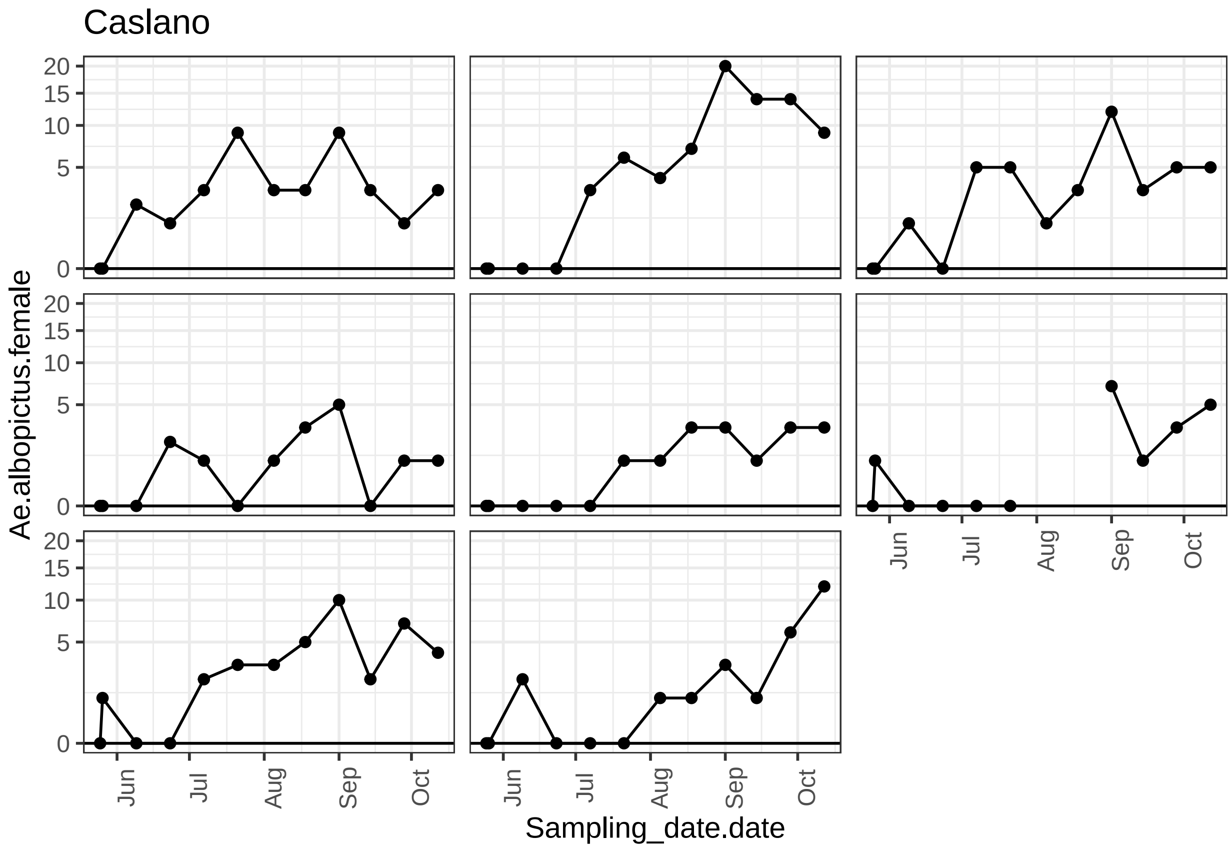


```
## save plot for future use:
saveRDS(p, file = file.path("saved_figures",
                             "2c_CaslanofemalesOverTimeNolabels.rds"))
```

There is still some variability, but it is less pronounced compared to Morcote.

5.2 Fitting the model

We aim to model the total number of *Aedes albopictus* females (*Ae.albopictus.females*) over the season (*yday*), while accounting for differences between municipalities (*municipality.fac*). Since sterile mosquitoes were released only in Morcote, municipalities will not be included as a random effect but rather as fixed effects to assess the actual differences.

Additionally, we will include an interaction between *municipality.fac* and *yday* to allow for distinct seasonal patterns across municipalities, avoiding the assumption that they behave similarly. These smooths will be centered, so the variable *municipality.fac* needs to be added as a main effect as well (because *municipality.fac* is a factor). In a subsequent section we will then test whether this distinct seasonal patterns across municipalities are needed.

yday will be included as numeric variable.

Given that *Ae.albopictus.females* represents count data, we will use a negative binomial model, which is appropriate for handling overdispersion in count data.

We don't want to assume any specific seasonal pattern, therefore we will fit a Generalised Additive Model (GAM), which provides flexibility in determining the best shape for the seasonal trends. However, this flexibility comes at the cost of interpretability.

Since observations within the same ovitrap are not independent, we will account for this dependency by setting *ID_BG_trap.fac* (the ovitrap unique identifier) as a random effect. This adjustment captures the variability specific to each ovitrap.

Let's check how many sampling dates there are for each municipality.

```
d.ovitraps.23 %>%
  group_by(municipality.fac) %>%
  summarise(nr.knots = n_distinct(yday)) %>%
  arrange(nr.knots)
```

```
# A tibble: 2 x 2
  municipality.fac nr.knots
  <fct>               <int>
1 Caslano                12
2 Morcote                12
```

Additionally, we will use a "point constraint" for *yday* (specifically at day 182), meaning all seasonal effects for *yday* are relative to the 1st of July.

```
pc.23 <- as.Date("2023-07-01") %>% yday()
pc.23
```

```
[1] 182
```

We first remove the observations having missing values for the relevant variables.

```
d.ovitraps.23.mod <- d.ovitraps.23 %>%
  select(Ae.albopictus.female,
         municipality.fac,
         ID_BG_trap.fac,
         yday,
         municipality.ord) %>%
  na.omit() %>%
  droplevels()
##
## check
dim(d.ovitraps.23)
```

```
[1] 396  29
```

```
dim(d.ovitraps.23.mod)
```

```
[1] 358   5
```

Now, we can fit the model. We use the `gamV()` function instead of `gam()`, because it fits the `gam()` model and automatically converts it to a `gamViz` object, making visualisation easier.
The model's estimates are exactly the same.

Note that the fitting procedure takes a few minutes. Therefore, the model is fitted and stored as RDS file and does not need to be refitted at each compilation.

```r
## (this chunk is not evaluated. It takes about 2 minutes to be evaluated)
##
gamm.tot.females.23 <- gamV(Ae.albopictus.female ~
                              s(yday, by = municipality.fac, pc = pc.23) +
                              municipality.fac +
                              s(ID_BG_trap.fac, bs = "re"),
                            family = "nb",
                            data = d.ovitraps.23.mod)
##
saveRDS(gamm.tot.females.23,
        file = "Prepared_data_and_models/GAMM_tot_females.23.RDS")
```

We load the previously fitted model.

```r
gamm.tot.females.23 <- readRDS("Prepared_data_and_models/GAMM_tot_females.23.RDS")
##
summary(gamm.tot.females.23)
```

```
Family: Negative Binomial(4.651)
Link function: log

Formula:
Ae.albopictus.female ~ s(yday, by = municipality.fac, pc = pc.23) +
    municipality.fac + s(ID_BG_trap.fac, bs = "re")

Parametric coefficients:
                         Estimate Std. Error z value Pr(>|z|)
(Intercept)             -0.007543   0.311940  -0.024   0.9807
municipality.facMorcote -0.691653   0.396662  -1.744   0.0812 .
---
Signif. codes:  0 '***' 0.001 '**' 0.01 '*' 0.05 '.' 0.1 ' ' 1

Approximate significance of smooth terms:
                                   edf Ref.df Chi.sq p-value
s(yday):municipality.facCaslano  3.293  4.091  71.12  <2e-16 ***
s(yday):municipality.facMorcote  5.924  7.081  64.69  <2e-16 ***
s(ID_BG_trap.fac)               23.645 31.000  94.72  <2e-16 ***
---
Signif. codes:  0 '***' 0.001 '**' 0.01 '*' 0.05 '.' 0.1 ' ' 1

R-sq.(adj) =  0.565   Deviance explained = 58.9%
-REML =  508.3  Scale est. = 1         n = 358
```

We also extract the standard deviation corresponding to the random effect.

```r
gam.vcomp(gamm.tot.females.23) %>%
  tail(n = 1)
```

```
Standard deviations and 0.95 confidence intervals:

                                    std.dev       lower      upper
s(yday):municipality.facCaslano 0.004531963 0.001054416 0.01947873
s(yday):municipality.facMorcote 0.013620883 0.005142554 0.03607710
s(ID_BG_trap.fac)               0.654465816 0.446562741 0.95916086
```

```
Rank: 3/3

                    std.dev     lower     upper
s(ID_BG_trap.fac) 0.6544658 0.4465627 0.9591609
```

5.3 Plotting the model

We now plot the fitted model. Since we allowed the model to fit a different trend for each municipality, there will be a separate plot for each of them.

```
plot.gam(gamm.tot.females.23,
         pages = 1,
         trans = exp)
```

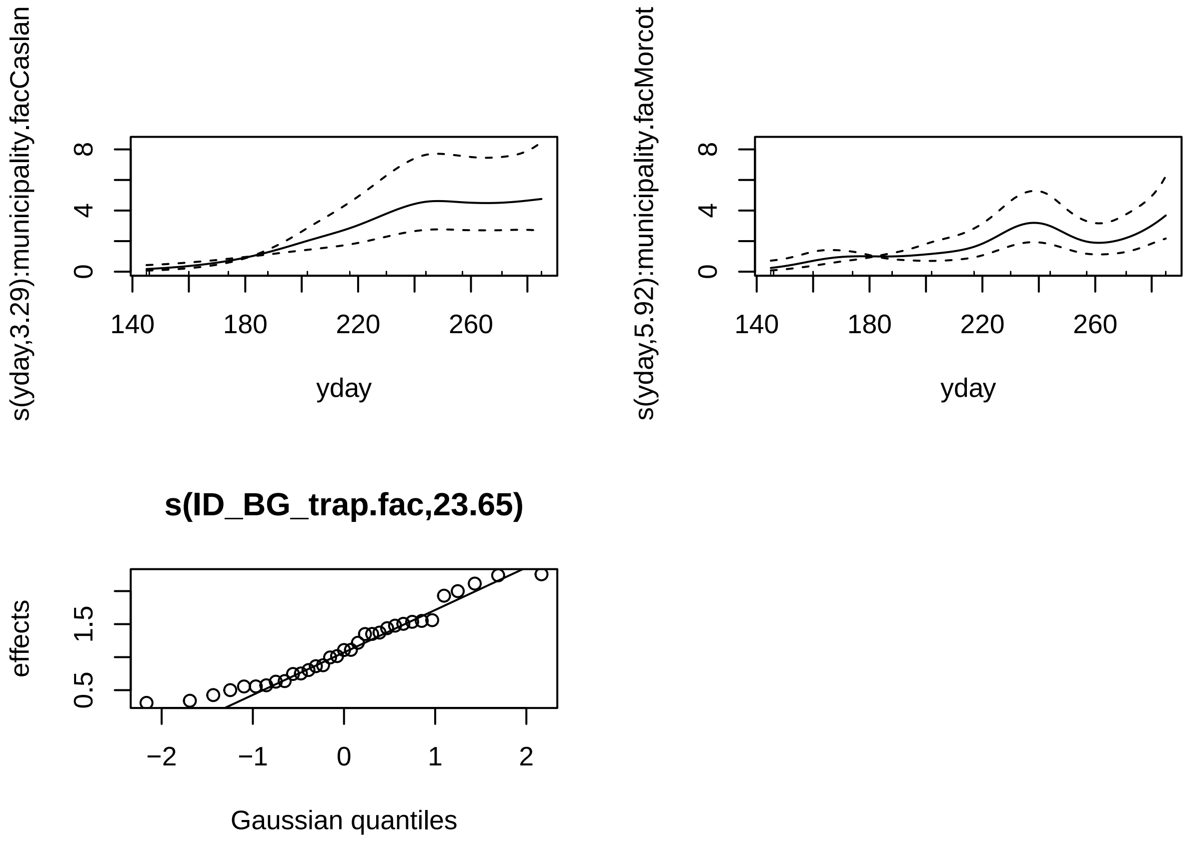

We observe some variability among the two municipalities. These differences will be formally tested in the next steps.

We can draw the graphs on the same plot with confidence intervals. This is performed to compare the different shapes.

```
## Extract data from the plots
gamm.tot.females.23.plot.tmp <- lapply(plot(gamm.tot.females.23)$plots,
                                       function(x) x$data$fit)
##
gamm.tot.females.23.plot.tmp <-
  lapply(1:(length(gamm.tot.females.23.plot.tmp)-1),
         function(ii) {
```

```
            out <- gamm.tot.females.23.plot.tmp[[ii]]
            out$municipality <- ii
            return(out)
          } )

## Combine data in a unique data set
gamm.tot.females.23.plot <- do.call("rbind", gamm.tot.females.23.plot.tmp)
##
## Create a factor for the municipality group
gamm.tot.females.23.plot$municipality <- as.factor(gamm.tot.females.23.plot$municipality)
##
## Create CI
gamm.tot.females.23.plot$upper <- gamm.tot.females.23.plot$ty + 2 * gamm.tot.females.23.plot$se
gamm.tot.females.23.plot$lower <- gamm.tot.females.23.plot$ty - 2 * gamm.tot.females.23.plot$se
##
## Plot the data
ggplot(data = gamm.tot.females.23.plot, mapping = aes(x = x, y = ty,
                                                       colour = municipality,
                                                       group = municipality,
                                                       fill = municipality)) +
  geom_line() +
  geom_ribbon(data = subset(gamm.tot.females.23.plot, lower < y & y < upper),
              aes(ymin = lower, ymax = upper),
              alpha = 0.3,
              lty = 2)
```

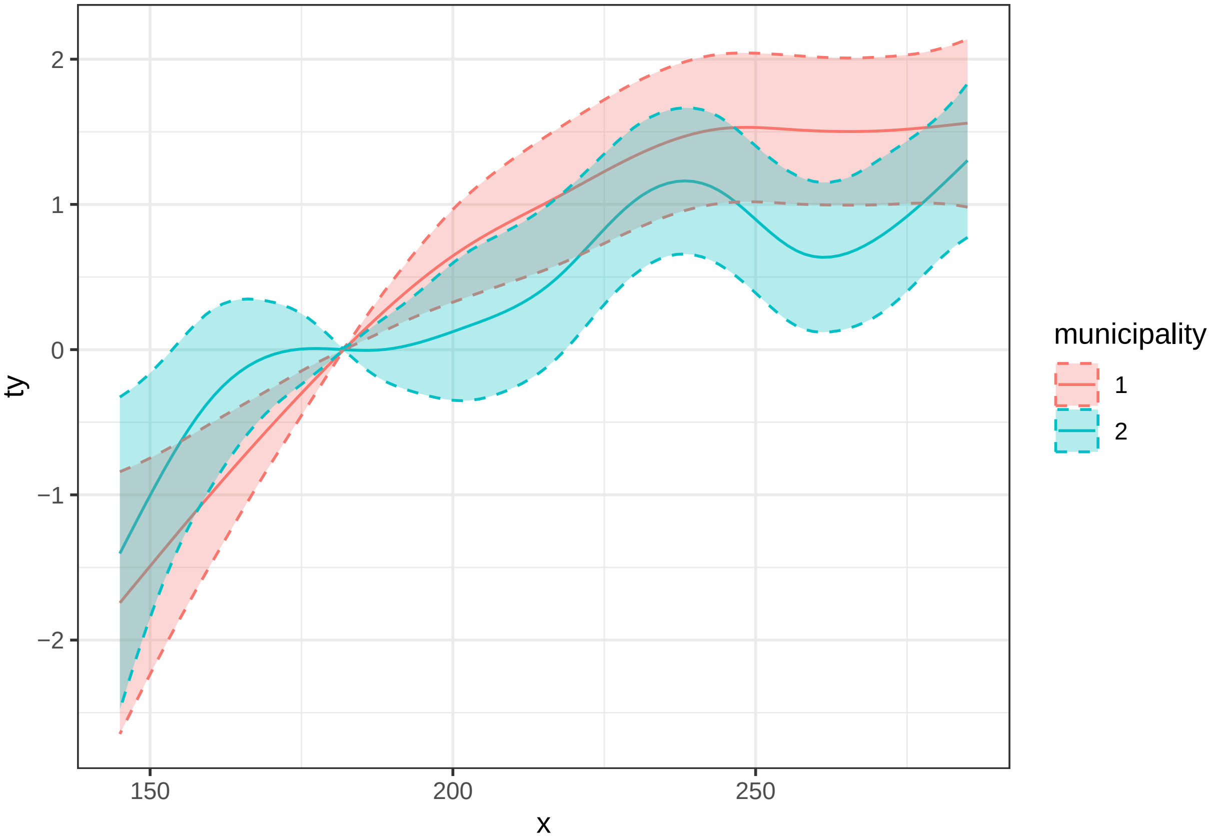

From this graph, it seems necessary to allow different shapes for smoothers. We will formally verify this later

in the sections.

We draw again the same plots, separately, but adding the correct shift to all of them.

```
plot.gam(gamm.tot.females.23,
         select = 1,
         shift = coef(gamm.tot.females.23)["(Intercept)"],
         trans = exp,
         main = levels(d.ovitraps.23.mod$municipality.fac)[1])
```

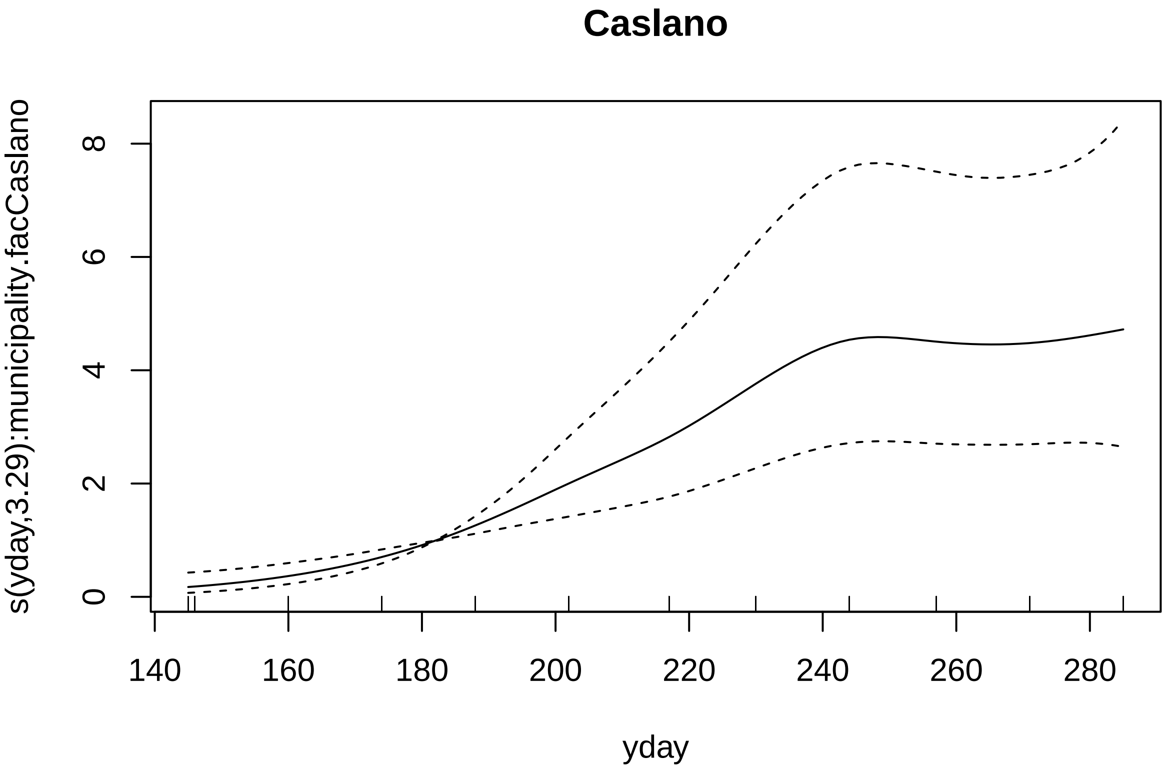


```
##
plot.gam(gamm.tot.females.23,
         select = 2,
         shift = coef(gamm.tot.females.23)["(Intercept)"] +
           coef(gamm.tot.females.23)["municipality.facMorcote"],
         trans = exp,
         main = levels(d.ovitraps.23.mod$municipality.fac)[2])
```

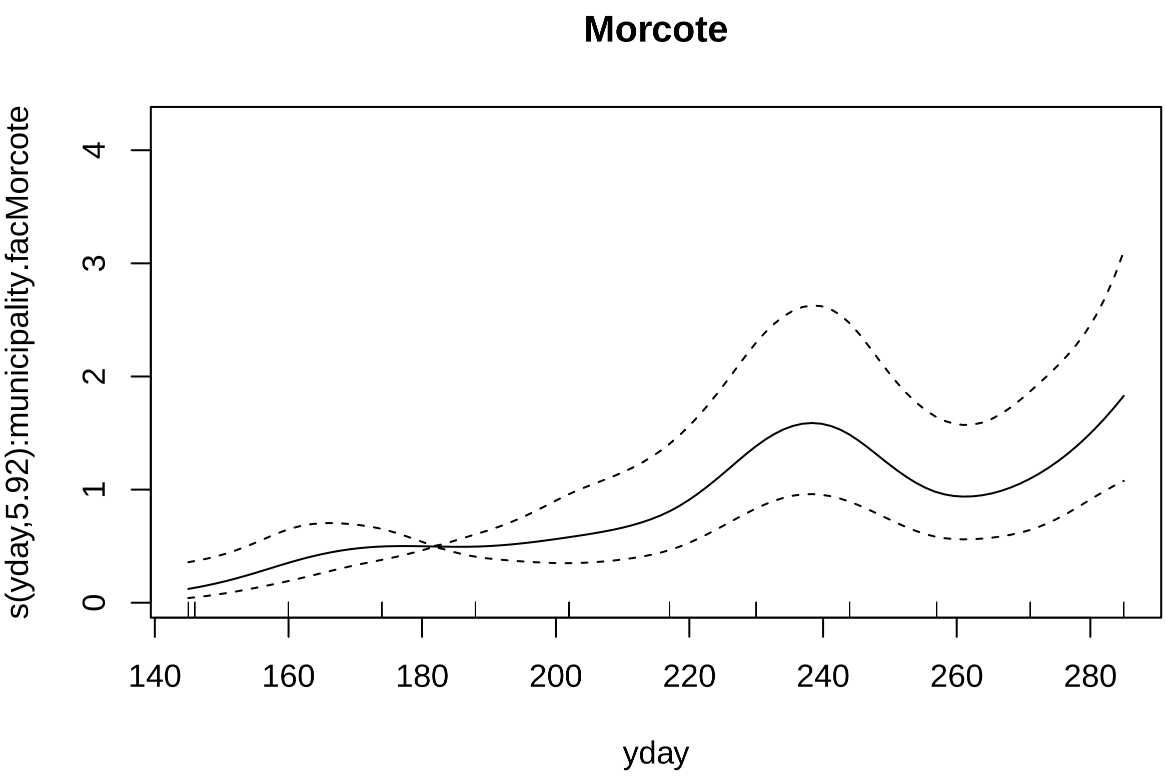

5.4 Fitted values

First, we begin by plotting the fitted values over time, creating a separate graph for each municipality. Observations from the same ovitrap will be connected by lines to visualise the trends.

```
d.ovitraps.23.mod$fitted_gamm.tot.females.23 <- fitted(gamm.tot.females.23)
##
ggplot(data = d.ovitraps.23.mod,
       mapping = aes(y = fitted_gamm.tot.females.23,
                     x = yday,
                     group = ID_BG_trap.fac)) +
  geom_hline(yintercept = 0) +
  geom_point(alpha = 0.1) +
  geom_line(alpha = 0.1) +
  scale_y_sqrt() +
  facet_wrap(~ municipality.fac)
```

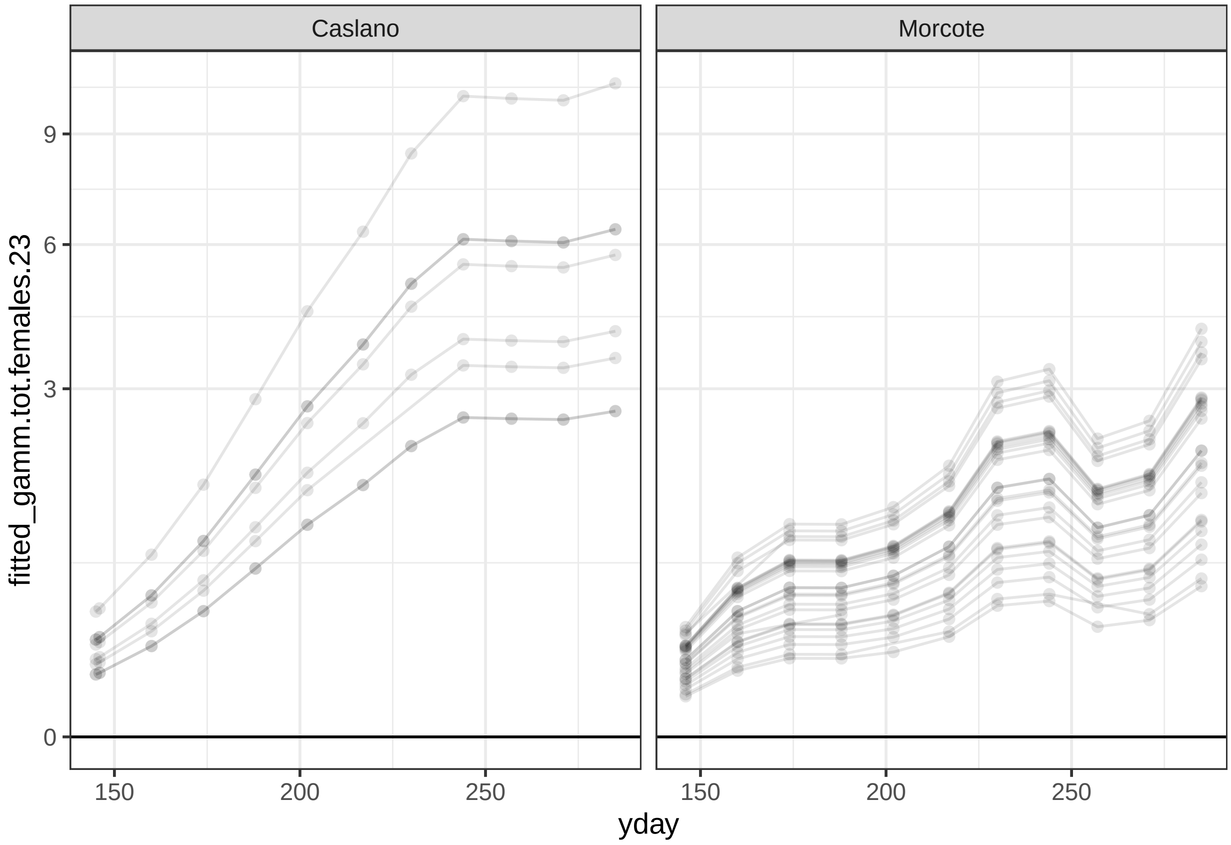

Note that there is significant variability among ovitraps within both Morcote and Caslano.

5.5 Predicted values

Let's create the data set to make predictions on.

```
## (messages are omitted from this chunk)
##
formula(gamm.tot.females.23)
```

```
Ae.albopictus.female ~ s(yday, by = municipality.fac, pc = pc.23) +
    municipality.fac + s(ID_BG_trap.fac, bs = "re")
```

```
##
## 1. We create a data set with municipality.fac and ovitrap ID
d.muni.ovitrap.short <- d.ovitraps.23.mod %>%
  select(municipality.fac, ID_BG_trap.fac) %>%
  unique()
##
head(d.muni.ovitrap.short)
```

```
# A tibble: 6 x 2
  municipality.fac ID_BG_trap.fac
  <fct>            <fct>
1 Caslano          26
2 Caslano          27
3 Caslano          28
4 Caslano          29
```

```
5 Caslano          30
6 Caslano          31
```

```r
nrow(d.muni.ovitrap.short)
```

```
[1] 33
```

```r
##
## 2. We create a data set with varying yday for each ovitrap ID
d.pred.gamm <- expand.grid(
  yday = seq(from = min(d.ovitraps.23.mod$yday),
             to = max(d.ovitraps.23.mod$yday),
             length.out = 100),
  ID_BG_trap.fac = d.muni.ovitrap.short$ID_BG_trap.fac)
##
## 3. We join the two data sets.
## Note that not all ovitraps exist in all municipalities.
## So, we can't simply use expand.grid()
d.pred.gamm_aug <- left_join(d.pred.gamm, d.muni.ovitrap.short)
str(d.pred.gamm_aug)
```

```
'data.frame':   3300 obs. of  3 variables:
 $ yday            : num  145 146 148 149 151 ...
 $ ID_BG_trap.fac  : Factor w/ 33 levels "1","10","11",..: 19 19 19 19 19 19 19 19 19 19 ...
 $ municipality.fac: Factor w/ 2 levels "Caslano","Morcote": 1 1 1 1 1 1 1 1 1 1 ...
 - attr(*, "out.attrs")=List of 2
  ..$ dim     : Named int [1:2] 100 33
  .. ..- attr(*, "names")= chr [1:2] "yday" "ID_BG_trap.fac"
  ..$ dimnames:List of 2
  .. ..$ yday          : chr [1:100] "yday=145.0000" "yday=146.4141" "yday=147.8283" "yday=149.2424" ..
  .. ..$ ID_BG_trap.fac: chr [1:33] "ID_BG_trap.fac=26" "ID_BG_trap.fac=27" "ID_BG_trap.fac=28" "ID_BG_
```

We make the predictions on the newly created data set, at ovitrap and population level, and we plot the result.

```r
## prediction at ovitrap level
d.pred.gamm_aug$predicted_gamm.tot.females.23 <- predict(
  gamm.tot.females.23,
  newdata = d.pred.gamm_aug,
  type = "response")
##
## prediction at population level
d.pred.gamm_aug$predicted_gamm.tot.females.23.pop <- predict(
  gamm.tot.females.23,
  newdata = d.pred.gamm_aug,
  type = "response",
  exclude = 's(ID_BG_trap.fac)')
##
p <- ggplot(data = d.pred.gamm_aug,
            mapping = aes(y = predicted_gamm.tot.females.23,
                          x = yday,
                          group = ID_BG_trap.fac)) +
  geom_hline(yintercept = 0) +
  geom_line(alpha = 0.2) +
  scale_y_sqrt() +
  geom_line(mapping = aes(y = predicted_gamm.tot.females.23.pop), colour = "red") +
```

```r
  facet_wrap(~ municipality.fac)
p
```

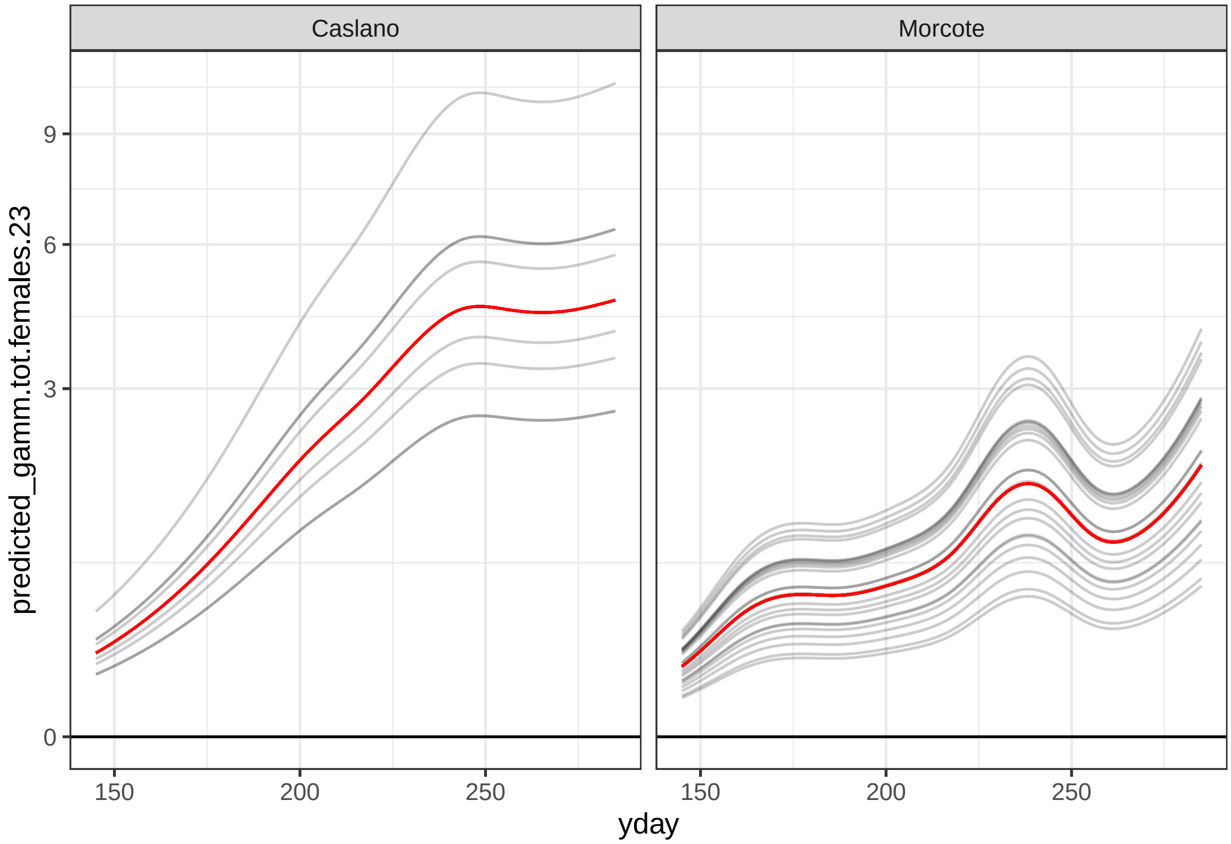


```r
## save plot for future use:
saveRDS(p, file = file.path("saved_figures",
                            "2c_MakePredictionGammTotfemales.rds"))
```

Each black line corresponds to the prediction for a given ovitrap, whereas the red line corresponds to the prediction at population level.

The variability within both municipalities is quite pronounced.

We plot the predictions at population level on the same plot.

```r
p <- ggplot(data = d.pred.gamm_aug,
            mapping = aes(y = predicted_gamm.tot.females.23.pop,
                          x = yday,
                          colour = municipality.fac)) +
  geom_line() +
  geom_hline(yintercept = 0, colour = "grey")
p
```

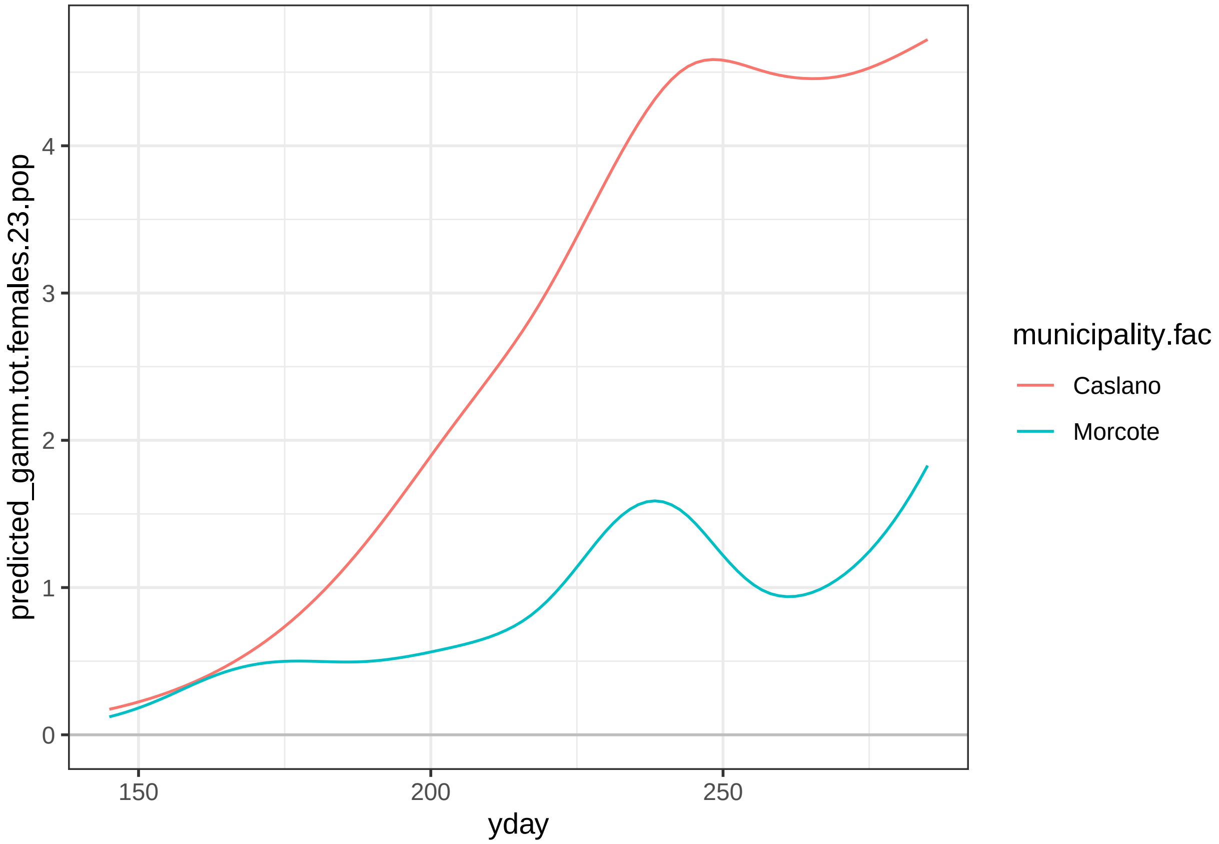


```
## save plot for future use:
saveRDS(p, file = file.path("saved_figures",
                            "2c_MakePredictionGammTotfemales_popLevel.rds"))
```

Predictions for females in Morcote are systematically lower than in Caslano.

5.6 Model selection

5.6.1 Shape

We fitted a model allowing a different smoother for each municipality. Now, we will assess whether this flexibility is truly necessary.

With this purpose in mind, we refit the model, but this time without allowing for different shapes across municipalities. We will then compare the two models to determine if the difference between them is statistically significant.

For performing this comparison, we need to refit the first model using an ordered factor (*municipality.ord*) instead of the classical one. This allows us to have nested model matrices and formally compare the two models.

```
## (this chunk is not evaluated)
gamm.tot.females.23.ord <- gamV(
  Ae.albopictus.female ~
    s(ID_BG_trap.fac, bs = "re") +
    s(yday, pc = pc.23) +
    municipality.fac +
    s(yday, pc = pc.23, by = municipality.ord),
```

```r
  family = "nb",
  data = d.ovitraps.23.mod)
##
saveRDS(gamm.tot.females.23.ord,
        "Prepared_data_and_models/GAMM_tot_females_ord.23.RDS")
##
gamm.tot.females.23_one.smooth <- gamV(
  Ae.albopictus.female ~
    s(ID_BG_trap.fac, bs = "re") +
    s(yday, pc = pc.23) +
    municipality.fac,
  # s(yday, pc = 182, by = municipality.ord),
  family = "nb",
  data = d.ovitraps.23.mod)
##
saveRDS(gamm.tot.females.23_one.smooth,
        "Prepared_data_and_models/GAMM_tot_females_one_smooth.23.RDS")
```

Let's get the previously fitted models.

```r
gamm.tot.females.23.ord <- readRDS(paste0("Prepared_data_and_models/",
                                          "GAMM_tot_females_ord.23.RDS"))
gamm.tot.females.23_one.smooth <- readRDS(paste0("Prepared_data_and_models/",
                                                 "GAMM_tot_females_one_smooth.23.RDS"))
##
summary(gamm.tot.females.23.ord)
```

```

Family: Negative Binomial(5.986)
Link function: log

Formula:
Ae.albopictus.female ~ s(ID_BG_trap.fac, bs = "re") + s(yday,
    pc = pc.23) + municipality.fac + s(yday, pc = pc.23, by = municipality.ord)

Parametric coefficients:
                        Estimate Std. Error z value Pr(>|z|)
(Intercept)             -0.01006    0.32268  -0.031   0.9751
municipality.facMorcote -0.74522    0.34468  -2.162   0.0306 *
---
Signif. codes:  0 '***' 0.001 '**' 0.01 '*' 0.05 '.' 0.1 ' ' 1

Approximate significance of smooth terms:
                                   edf Ref.df  Chi.sq p-value
s(ID_BG_trap.fac)               23.971 31.000 103.319  <2e-16 ***
s(yday)                          7.385  8.342 101.073  <2e-16 ***
s(yday):municipality.ordMorcote  1.942  2.422   5.028   0.138
---
Signif. codes:  0 '***' 0.001 '**' 0.01 '*' 0.05 '.' 0.1 ' ' 1

R-sq.(adj) =  0.606   Deviance explained =   61%
-REML = 504.24  Scale est. = 1         n = 358
```

```r
summary(gamm.tot.females.23_one.smooth)
```

```
Family: Negative Binomial(5.708)
Link function: log

Formula:
Ae.albopictus.female ~ s(ID_BG_trap.fac, bs = "re") + s(yday,
    pc = pc.23) + municipality.fac

Parametric coefficients:
                        Estimate Std. Error z value Pr(>|z|)
(Intercept)               0.1957     0.3007   0.651 0.515181
municipality.facMorcote  -1.0818     0.2929  -3.694 0.000221 ***
---
Signif. codes:  0 '***' 0.001 '**' 0.01 '*' 0.05 '.' 0.1 ' ' 1

Approximate significance of smooth terms:
                     edf Ref.df Chi.sq p-value
s(ID_BG_trap.fac) 23.886 31.000  102.5  <2e-16 ***
s(yday)            7.444  8.391  148.8  <2e-16 ***
---
Signif. codes:  0 '***' 0.001 '**' 0.01 '*' 0.05 '.' 0.1 ' ' 1

R-sq.(adj) =  0.595   Deviance explained = 60.1%
-REML = 505.32  Scale est. = 1         n = 358
```

We check whether the two models are nested. If they are, then we can test whether the most complex one is necessary, i.e., if we have evidence indicating that the more complex model improves the more simple model.

```
## Extract model matrices
m.gamm.tot.females.23.ord <- model.matrix(gamm.tot.females.23.ord)
m.gamm.tot.females.23_one.smooth <- model.matrix(gamm.tot.females.23_one.smooth) ##
dim(m.gamm.tot.females.23.ord)
```

```
[1] 358  53
```

```
dim(m.gamm.tot.females.23_one.smooth)
```

```
[1] 358  44
```

```
##
n1 <- ncol(m.gamm.tot.females.23_one.smooth)
## check that sum is zero
sum(matrix( m.gamm.tot.females.23_one.smooth[, 1:n1] - m.gamm.tot.females.23.ord[, 1:n1] ) != 0)
```

```
[1] 0
```

The two model matrices are nested, therefore we can now compare the two models with the Chi-square test.

```
anova.gam(gamm.tot.females.23_one.smooth, gamm.tot.females.23.ord, test = "Chisq")
```

```
Analysis of Deviance Table

Model 1: Ae.albopictus.female ~ s(ID_BG_trap.fac, bs = "re") + s(yday,
    pc = pc.23) + municipality.fac
Model 2: Ae.albopictus.female ~ s(ID_BG_trap.fac, bs = "re") + s(yday,
    pc = pc.23) + municipality.fac + s(yday, pc = pc.23, by = municipality.ord)
  Resid. Df Resid. Dev     Df Deviance Pr(>Chi)
1    317.15     900.04
```

```
2    313.56     893.69 3.5909   6.3495   0.1395
```

We have no evidence that the more complex model improves the simpler mode, which means that the additional flexibility, allowing different shapes for the smoother in each municipality, is not necessary.

For good practice, we also look at AIC and BIC.

```
AIC(gamm.tot.females.23.ord, gamm.tot.females.23_one.smooth)
```

```
                                      df      AIC
gamm.tot.females.23.ord         39.15168 971.9955
gamm.tot.females.23_one.smooth 36.02928 972.1002
```

```
BIC(gamm.tot.females.23.ord, gamm.tot.females.23_one.smooth)
```

```
                                      df      BIC
gamm.tot.females.23.ord         39.15168 1123.925
gamm.tot.females.23_one.smooth 36.02928 1111.913
```

AIC and BIC disagree on the need of a smoother per municipality, therefore we choose the most parsimonious model.

5.6.2 Shift

We have found that allowing two different shapes for the smoothers is not necessary. Therefore, we can keep the simpler model and test whether two different intercepts for municipalities are necessary.

This can be performed by looking at the summary of the model with a different intercept for each municipality.

```
summary(gamm.tot.females.23_one.smooth)
```

```

Family: Negative Binomial(5.708)
Link function: log

Formula:
Ae.albopictus.female ~ s(ID_BG_trap.fac, bs = "re") + s(yday,
    pc = pc.23) + municipality.fac

Parametric coefficients:
                        Estimate Std. Error z value Pr(>|z|)
(Intercept)               0.1957     0.3007   0.651 0.515181
municipality.facMorcote  -1.0818     0.2929  -3.694 0.000221 ***
---
Signif. codes:  0 '***' 0.001 '**' 0.01 '*' 0.05 '.' 0.1 ' ' 1

Approximate significance of smooth terms:
                     edf Ref.df Chi.sq p-value
s(ID_BG_trap.fac) 23.886 31.000  102.5  <2e-16 ***
s(yday)            7.444  8.391  148.8  <2e-16 ***
---
Signif. codes:  0 '***' 0.001 '**' 0.01 '*' 0.05 '.' 0.1 ' ' 1

R-sq.(adj) =  0.595   Deviance explained = 60.1%
-REML = 505.32  Scale est. = 1         n = 358
```

The analysis indicates that a unique intercept is necessary for each municipality. Specifically, the p-value of 2.2110596×10^{-4}, being less than the 0.05 threshold, suggests that the intercept for Morcote significantly

differs from that of Caslano.

The fitted model indicates that the presence of female Ae albopictus mosquitoes in Morcote is 33.9 % of that observed in Caslano.

5.7 Residual analysis

Note that the residual analysis is run on *gamm.tot.females.23_one.smooth*.

First of all, we apply the `gam.check()` function to the model, which produces some diagnostic information.

```
par(mfrow = c(2, 2))
gam.check(gamm.tot.females.23_one.smooth)
```

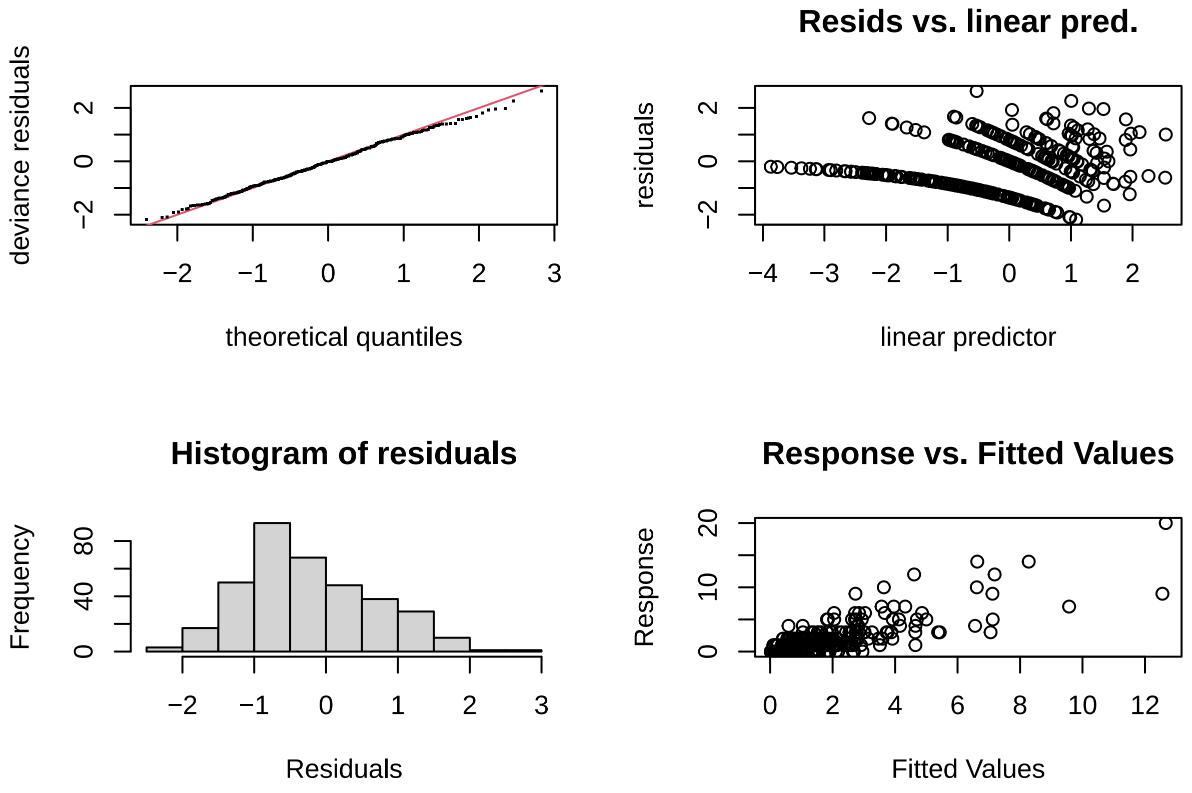


```
Method: REML   Optimizer: outer newton
full convergence after 5 iterations.
Gradient range [-0.0002272096,-3.271129e-06]
(score 505.317 & scale 1).
Hessian positive definite, eigenvalue range [1.545385,7.033695].
Model rank =  44 / 44

Basis dimension (k) checking results. Low p-value (k-index<1) may
indicate that k is too low, especially if edf is close to k'.

                      k'   edf k-index p-value
s(ID_BG_trap.fac) 33.00 23.89      NA      NA
s(yday)            9.00  7.44    0.88    0.09 .
---
Signif. codes:  0 '***' 0.001 '**' 0.01 '*' 0.05 '.' 0.1 ' ' 1
```

```r
par(mfrow = c(1, 1))
```

Then, we store the pearson residuals in the original data frame and we plot the residuals against the fitted values to see whether there is still structure in the data.

```r
d.ovitraps.23.mod$resid_gamm.tot.females.23_one.smooth <- resid(gamm.tot.females.23_one.smooth,
                                                              type = "pearson")
d.ovitraps.23.mod$fitted_gamm.tot.females.23_one.smooth <- fitted(gamm.tot.females.23_one.smooth,
                                                                type = "pearson")
##
ggplot(data = d.ovitraps.23.mod,
       mapping = aes(y = resid_gamm.tot.females.23_one.smooth,
                     x = fitted_gamm.tot.females.23_one.smooth)) +
  geom_hline(yintercept = 0) +
  geom_point(alpha = 0.2) +
  geom_smooth()
```

```
`geom_smooth()` using method = 'loess' and formula = 'y ~ x'
```

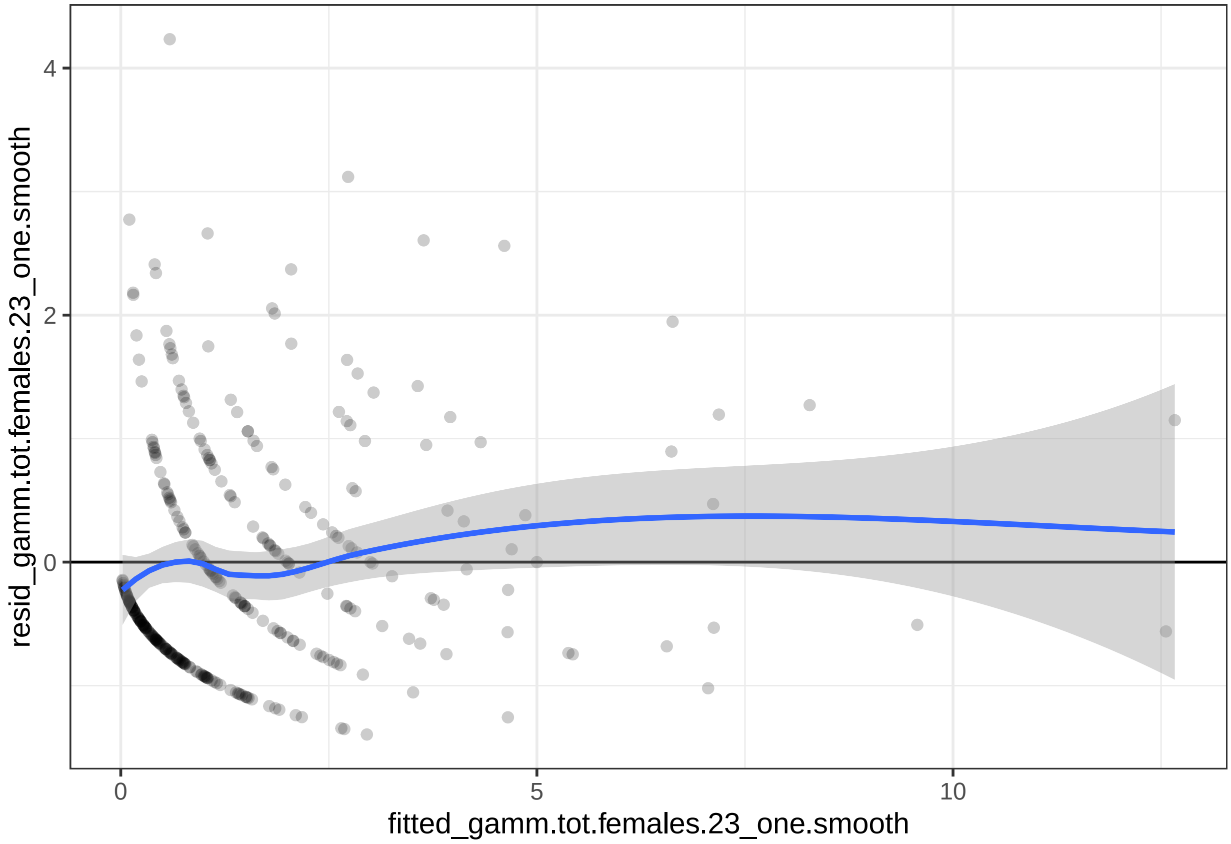

The blue line is located around the x-axis, as desired.

We plot the qq-plot for the random effects.

```r
## QQ for random effects
plot(sm(gamm.tot.females.23_one.smooth, 1))
```

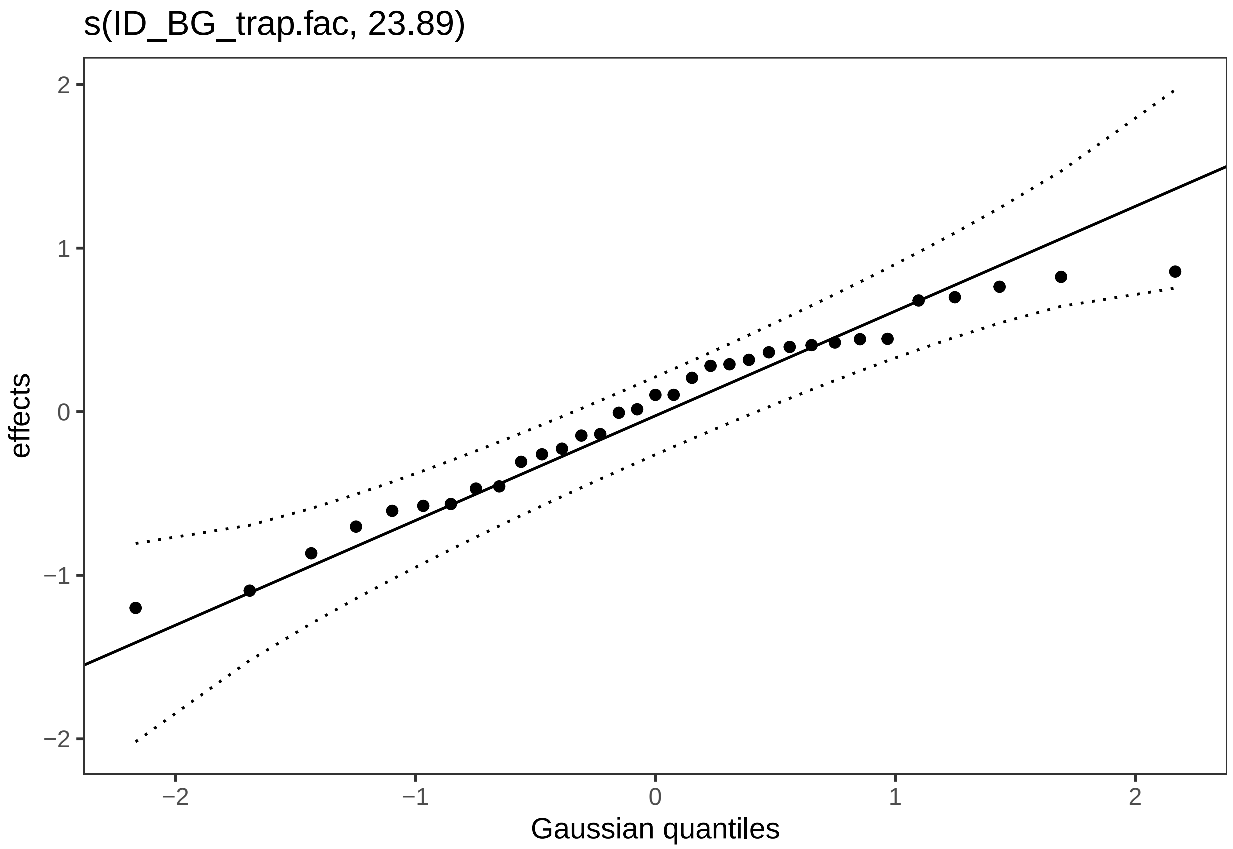

There doesn't seem to be a significant departure from the normality assumption.

We now plot the residuals over time to further check whether there is any structure left in the data.

```
ggplot(data = d.ovitraps.23.mod,
       mapping = aes(y = resid_gamm.tot.females.23_one.smooth,
                     x = yday)) +
  geom_hline(yintercept = 0) +
  geom_point(alpha = 0.2) +
  geom_smooth(method = "loess")
```

```
`geom_smooth()` using formula = 'y ~ x'
```

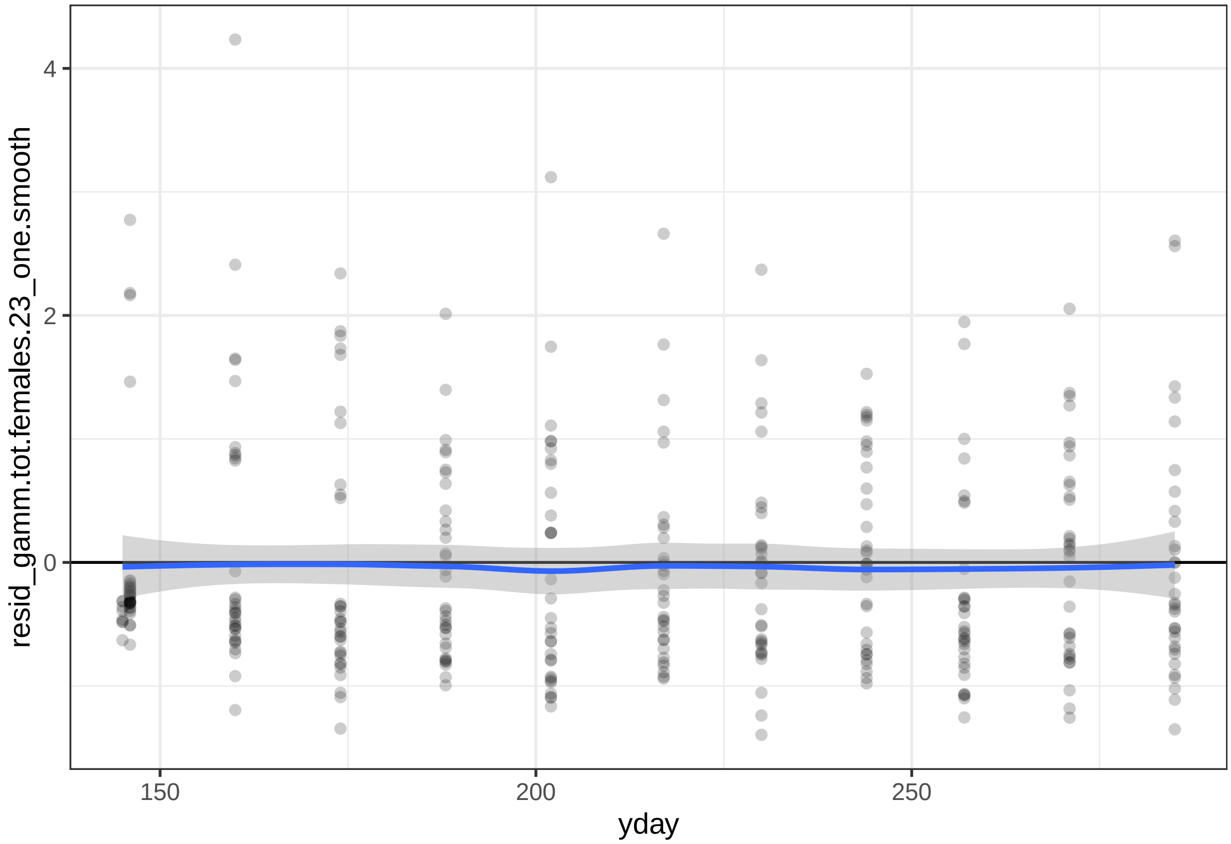

There does not seem to be any structure left in the data. Let's look into each single smoother (i.e. municipality).

```
ggplot(data = d.ovitraps.23.mod,
       mapping = aes(y = resid_gamm.tot.females.23_one.smooth,
                     x = yday)) +
  geom_hline(yintercept = 0) +
  geom_point(alpha = 0.2) +
  geom_smooth(method = "loess") +
  facet_wrap(~ municipality.fac, scales = "free")
```

```
`geom_smooth()` using formula = 'y ~ x'
```

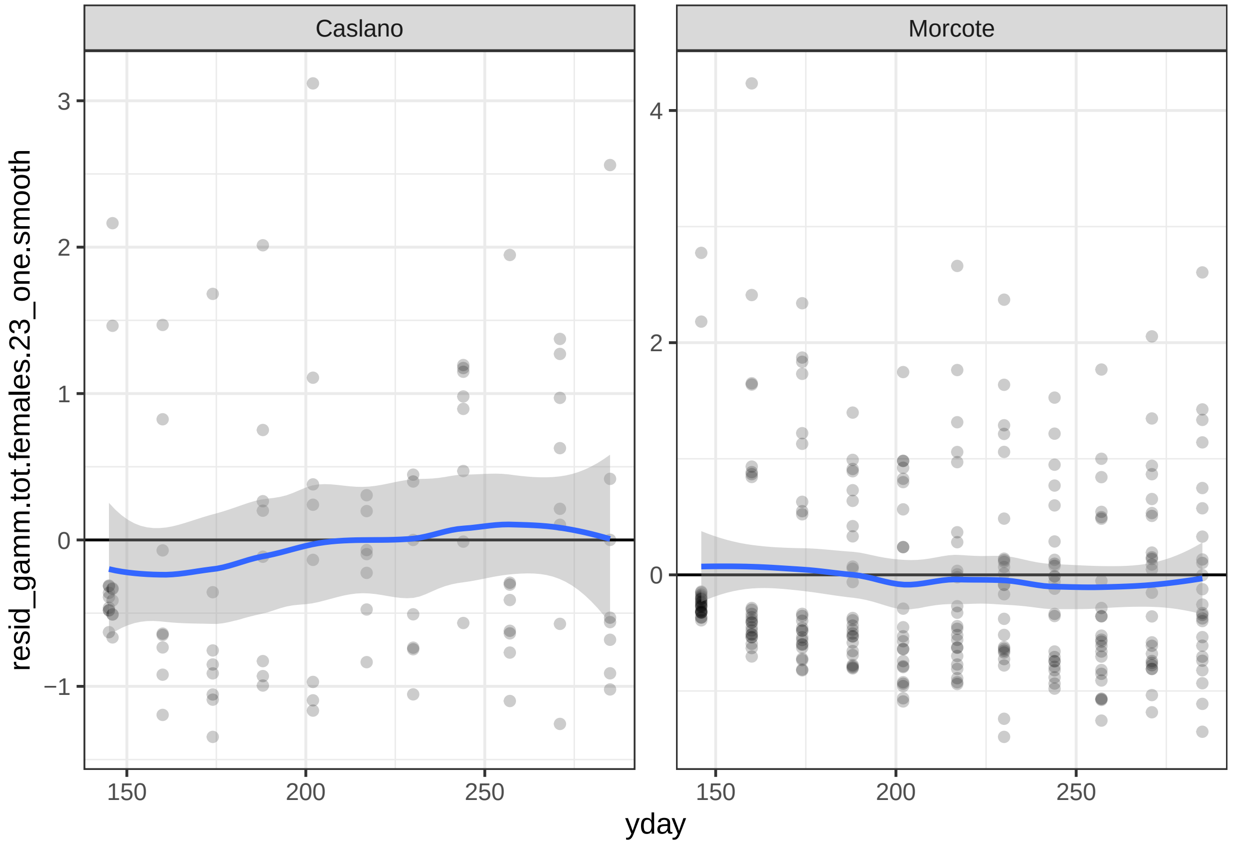

The blue lines are plotted on the x-axis, as desired.

The following two plots display the residuals in Morcote and Caslano, respectively.
The observations belonging to the same ovitrap are connected with a line. These plots are additionally drawn to check temporal correlation and variability.

```
## for Morcote
ggplot(data = filter(d.ovitraps.23.mod,
                     municipality.fac == "Morcote"),
       mapping = aes(y = resid_gamm.tot.females.23_one.smooth,
                     x = yday,
                     group = ID_BG_trap.fac)) +
  geom_hline(yintercept = 0) +
  geom_point(alpha = 0.2) +
  geom_line(alpha = 0.2) +
  labs(title = "Morcote")
```

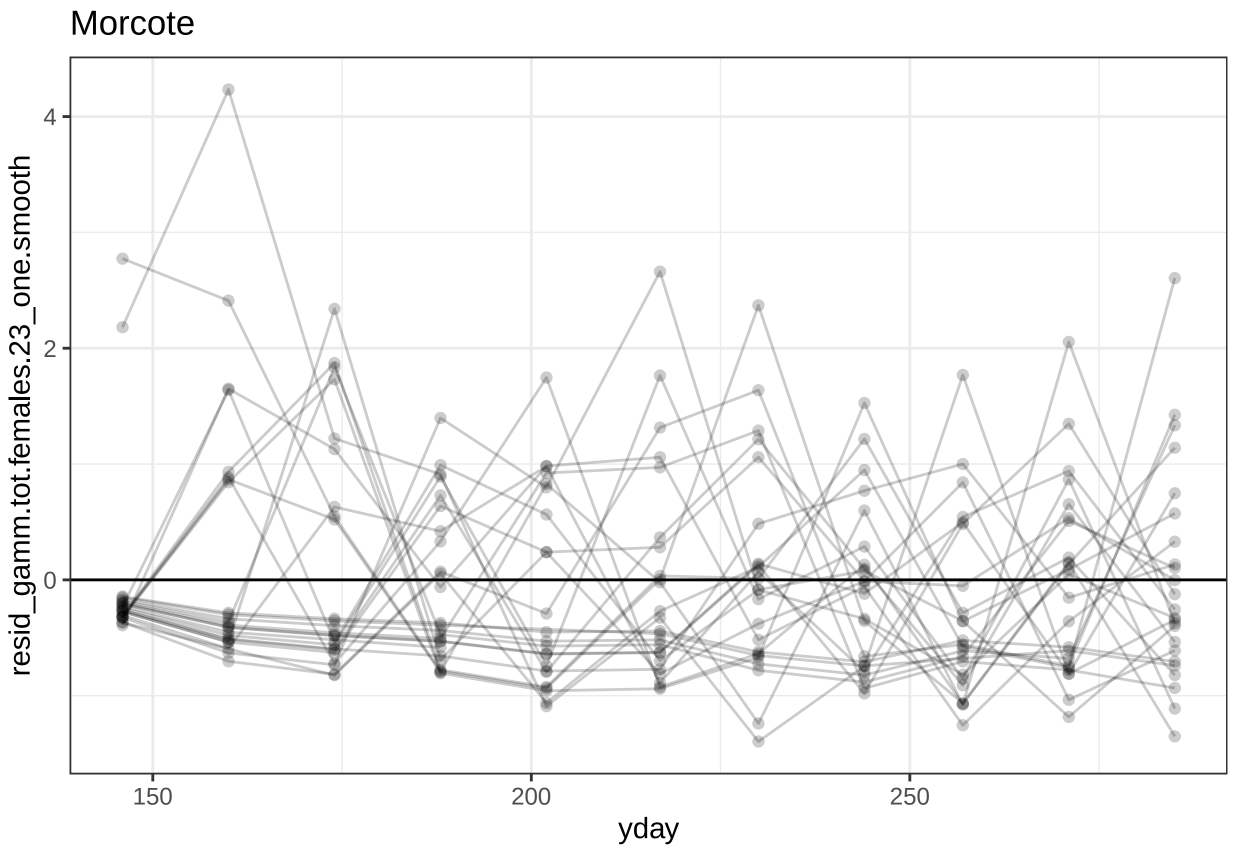


```
##
## for Caslano
ggplot(data = filter(d.ovitraps.23.mod,
                     municipality.fac == "Caslano"),
       mapping = aes(y = resid_gamm.tot.females.23_one.smooth,
                     x = yday,
                     group = ID_BG_trap.fac)) +
  geom_hline(yintercept = 0) +
  geom_point(alpha = 0.2) +
  geom_line(alpha = 0.2) +
  labs(title = "Caslano")
```

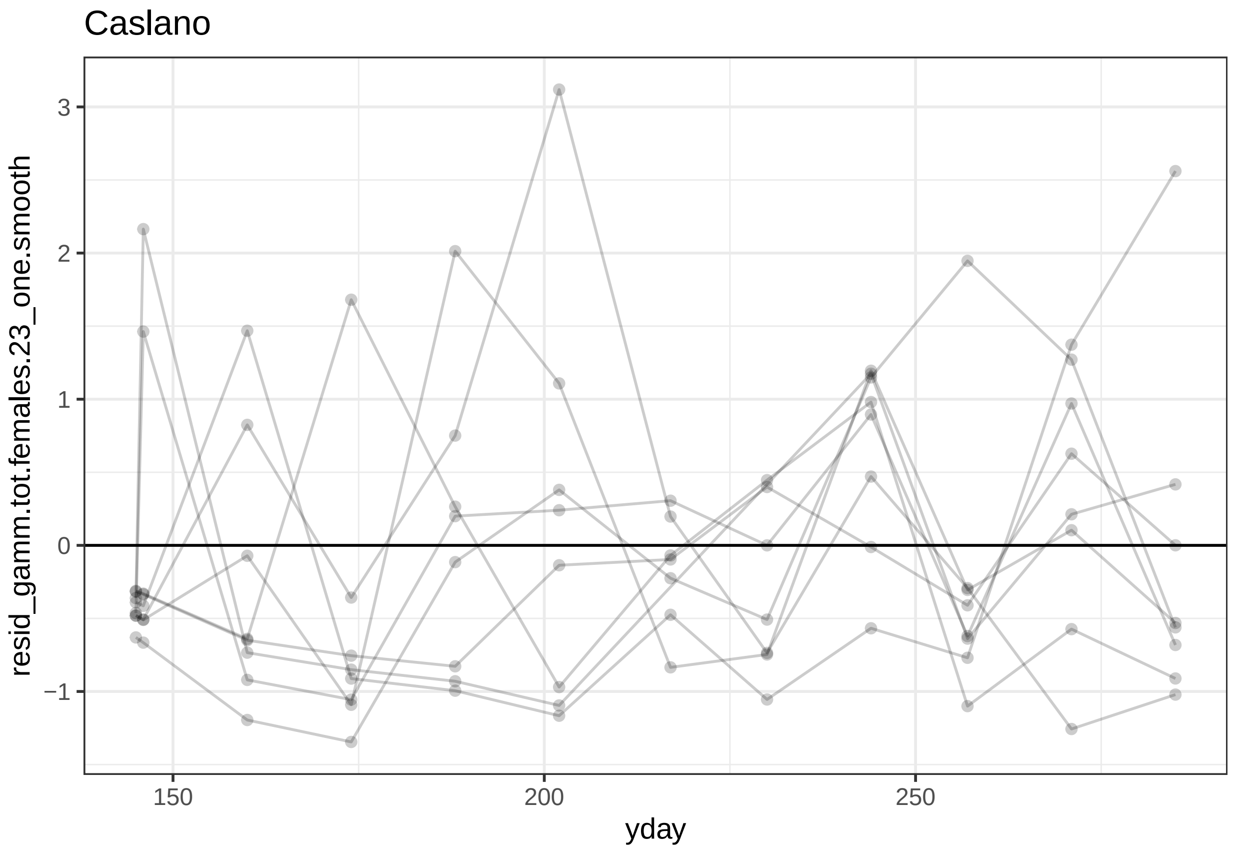

Let's look at the single ovitraps.

```
## for Morcote
ggplot(data = filter(d.ovitraps.23.mod,
                     municipality.fac == "Morcote"),
       mapping = aes(y = resid_gamm.tot.females.23_one.smooth,
                     x = yday,
                     group = ID_BG_trap.fac)) +
  geom_hline(yintercept = 0) +
  geom_point() +
  geom_line() +
  facet_wrap(~ ID_BG_trap.fac) +
  theme(
    strip.background = element_blank(),
    strip.text.x = element_blank()) +
  labs(title = "Morcote")
```

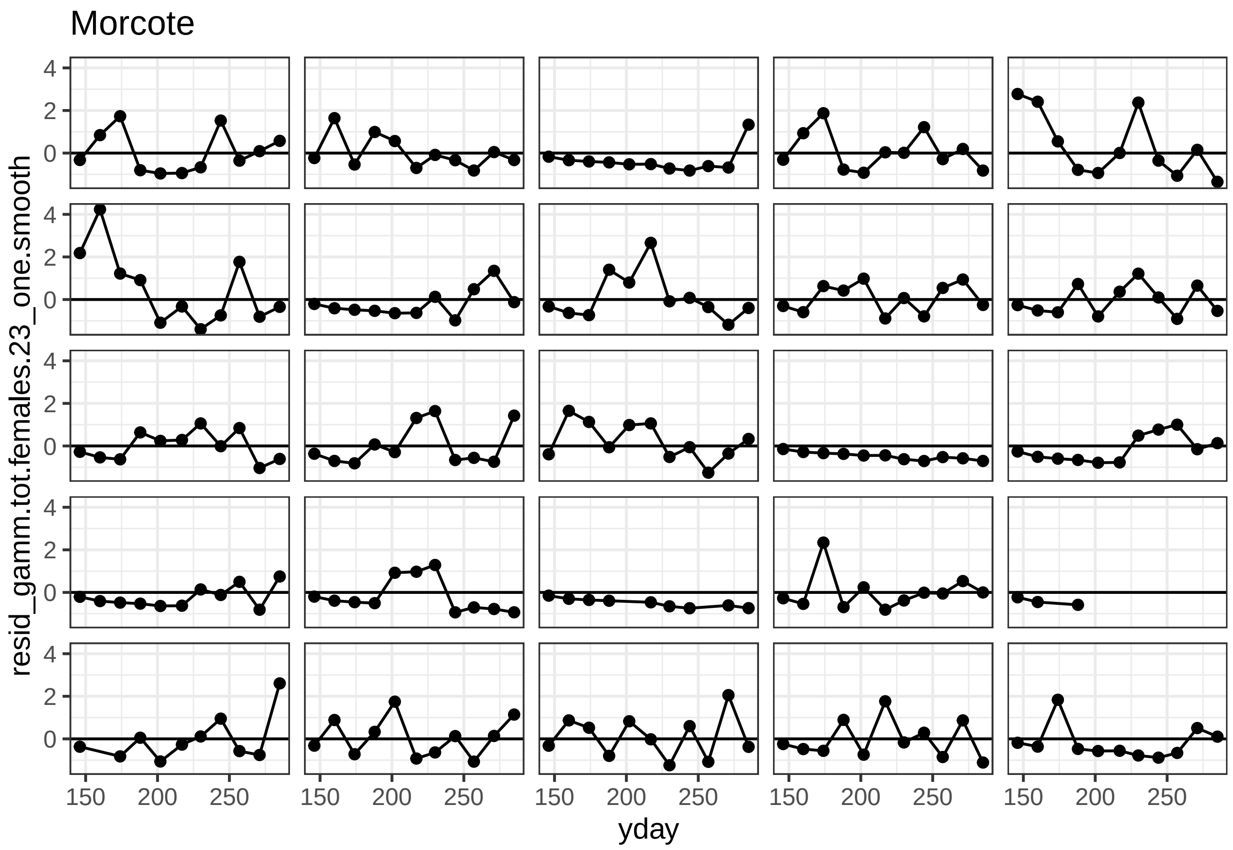


```
##
## for Caslano
ggplot(data = filter(d.ovitraps.23.mod,
                     municipality.fac == "Caslano"),
       mapping = aes(y = resid_gamm.tot.females.23_one.smooth,
                     x = yday,
                     group = ID_BG_trap.fac)) +
  geom_hline(yintercept = 0) +
  geom_point() +
  geom_line() +
  facet_wrap(~ ID_BG_trap.fac) +
  theme(
    strip.background = element_blank(),
    strip.text.x = element_blank()) +
  labs(title = "Caslano")
```

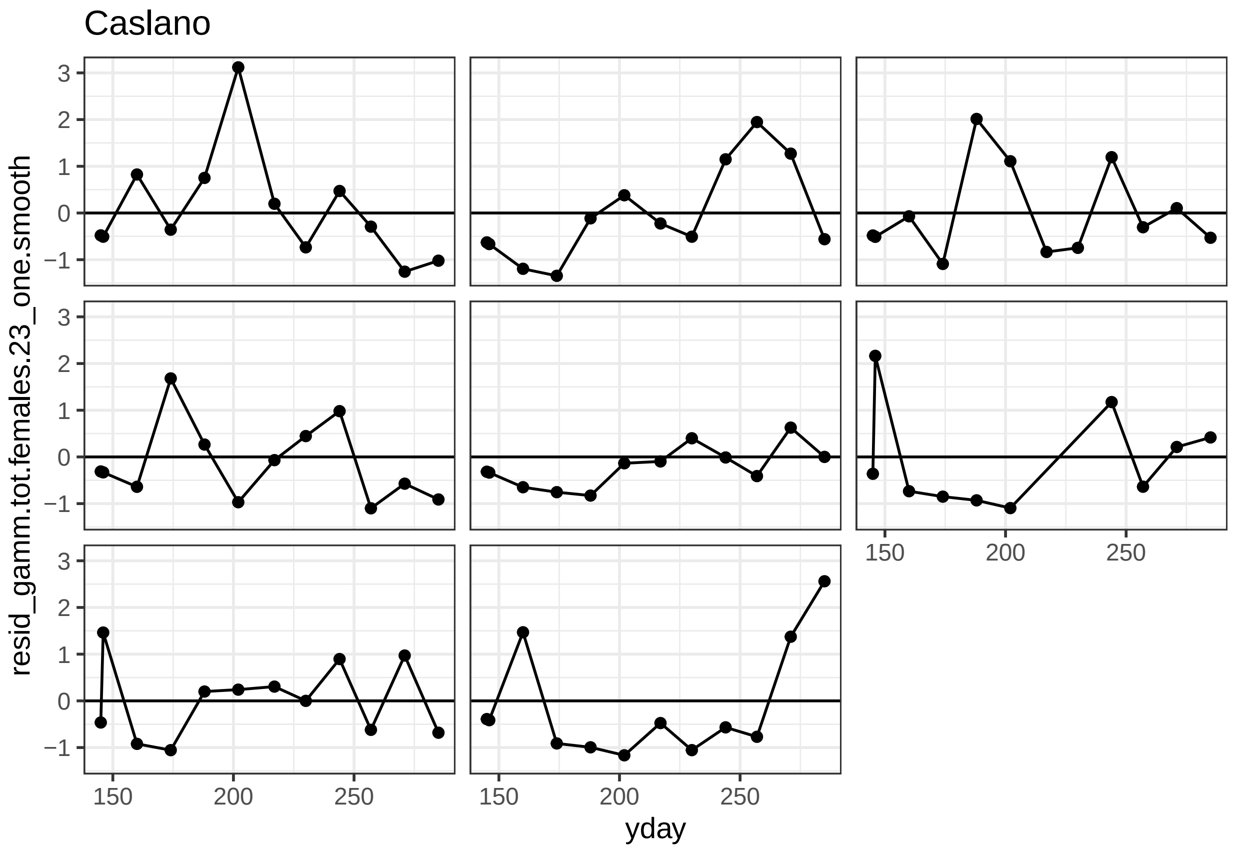

In both cases, there does not seem to be much structure left in the data.

If one were to do a formal test, there would probably be a temporal correlation. However, in this case we are more interested in modelling and understanding the effect rather than making a perfect inference, so the temporal correlation does not particularly affect our analysis.

5.8 Comparing (over)dispersion in the two models

Two separate models are fitted for Morcote and Caslano. This is performed to formally check whether the overdispersion parameter is different for the two municipalities.

```
## Model for Morcote
gam.tot.females.Morcote.23 <- gam(Ae.albopictus.female ~
                                    s(yday, pc = pc.23) +
                                    s(ID_BG_trap.fac, bs = "re"),
                                  family = "nb",
                                  data = filter(
                                    d.ovitraps.23.mod,
                                    municipality.fac == "Morcote")) ## new element!
summary(gam.tot.females.Morcote.23)
```

```
Family: Negative Binomial(7.372)
Link function: log

Formula:
Ae.albopictus.female ~ s(yday, pc = pc.23) + s(ID_BG_trap.fac,
    bs = "re")
```

```
Parametric coefficients:
            Estimate Std. Error z value Pr(>|z|)
(Intercept)  -0.7216     0.2526  -2.856  0.00428 **
---
Signif. codes:  0 '***' 0.001 '**' 0.01 '*' 0.05 '.' 0.1 ' ' 1

Approximate significance of smooth terms:
                     edf Ref.df Chi.sq p-value
s(yday)            6.129  7.282  70.27  <2e-16 ***
s(ID_BG_trap.fac) 18.641 24.000  70.46  <2e-16 ***
---
Signif. codes:  0 '***' 0.001 '**' 0.01 '*' 0.05 '.' 0.1 ' ' 1

R-sq.(adj) =  0.401   Deviance explained = 47.3%
-REML = 335.15  Scale est. = 1         n = 264
```

```r
##
## Model for Caslano
gam.tot.females.Caslano.23 <- gam(Ae.albopictus.female ~
                                    s(yday, pc = pc.23) +
                                    s(ID_BG_trap.fac, bs = "re"),
                                  family = "nb",
                                  data = filter(
                                    d.ovitraps.23.mod,
                                    municipality.fac == "Caslano")) ## new element!
summary(gam.tot.females.Caslano.23)
```

```
Family: Negative Binomial(3.386)
Link function: log

Formula:
Ae.albopictus.female ~ s(yday, pc = pc.23) + s(ID_BG_trap.fac,
    bs = "re")

Parametric coefficients:
             Estimate Std. Error z value Pr(>|z|)
(Intercept) 0.0003443  0.2753015   0.001    0.999

Approximate significance of smooth terms:
                    edf Ref.df Chi.sq  p-value
s(yday)           3.067  3.815  70.44  < 2e-16 ***
s(ID_BG_trap.fac) 5.419  7.000  24.38 4.74e-05 ***
---
Signif. codes:  0 '***' 0.001 '**' 0.01 '*' 0.05 '.' 0.1 ' ' 1

R-sq.(adj) =  0.547   Deviance explained = 62.3%
-REML = 172.21  Scale est. = 1         n = 94
```

To compare overdispersion between the two models, we can extract the θ parameter, which controls for it. The lower the value of θ, the higher the overdispersion.

In fact, in the `gam()` model from the `{mgcv}` package, θ for a negative binomial model is described to be the parameter such that

$$var(y) = \mu + \mu^2/\theta, \quad \text{where} \quad \mu = \mathbb{E}(y).$$

(see the help page of the `negbin()` function from the `{mgcv}` package).

Let's verify which model has lowest θ, i.e., highest overdispersion.

```
gam.tot.females.Morcote.23$family$getTheta(TRUE)
```

```
[1] 7.372314
```

```
gam.tot.females.Caslano.23$family$getTheta(TRUE)
```

```
[1] 3.385845
```

Caslano has the highest overdispersion between the two municipalities.

6 Spatial Generalised Additive Model – Females (spatial GAM)

We are now interested in testing whether geographical location plays a role in determining the number of females laid.

6.1 Visualising the data

Let us first take a look at the median number of females in the traps in Morcote. We calculate the median because we are dealing with skewed data.

```
## (warnings and messages are omitted from this chunk)
##
d.ovitraps.23.median <- d.ovitraps.23 %>%
  group_by(ID_BG_trap.fac, municipality.fac, X.num, Y.num) %>%
  summarise(median.females = median(Ae.albopictus.female, na.rm = TRUE)) %>%
  ungroup()
##
## We calculate the min and max for the median number of eggs.
## This is used in the plots, to use the same colour scale for the two
## municipalities and compare them more easily.
max.females.median <- max(d.ovitraps.23.median$median.females)
min.females.median <- min(d.ovitraps.23.median$median.females)
##
##
p <- ggplot(filter(d.ovitraps.23.median,
                   municipality.fac == "Morcote"),
            mapping = aes(y = Y.num,
                          x = X.num,
                          colour = median.females)) +
  geom_point(size = 3) +
  scale_color_gradientn(colours = c("blue", "purple", "red"),
                        values = scales::rescale(c(min.females.median,
                                                   max.females.median)),
                        limits = c(min.females.median, max.females.median)) +
  # theme(aspect.ratio = 1) +
  coord_fixed() +
  labs(title = "Morcote")
p
```

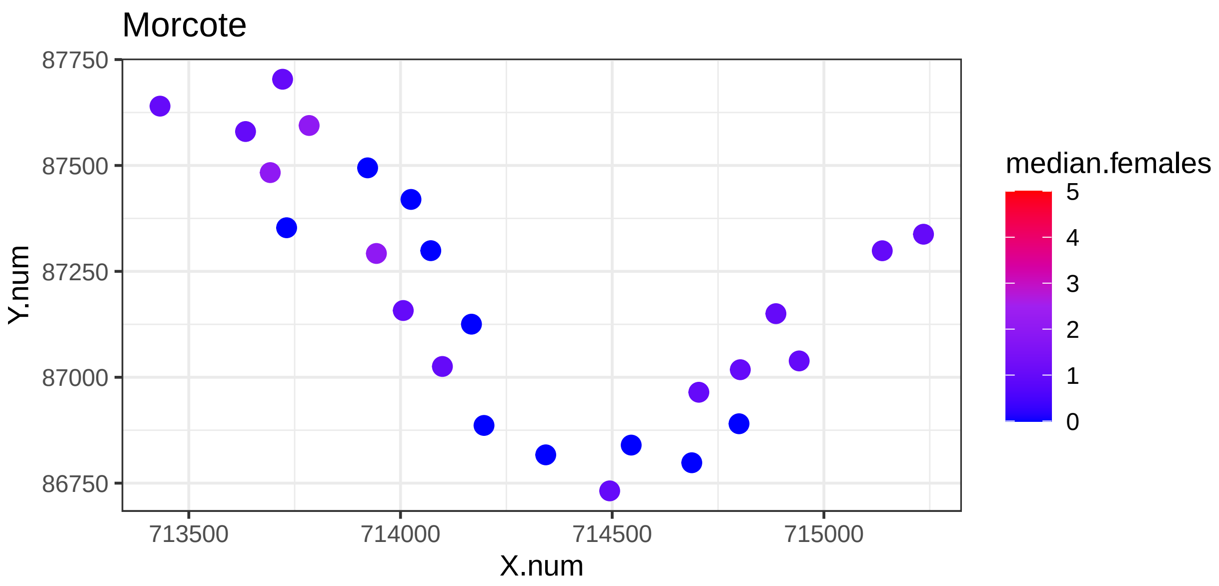


```r
## save plot for future use:
saveRDS(p, file = file.path("saved_figures",
                            "2c_meanEgssOverSpaceMorcote_graphForPaper.rds"))
```

There doesn't seem to be a strong spatial pattern; the median number of females is very low on the entire territory of Morcote.

```r
## (warnings are omitted from this chunk)
##
p <- ggplot(filter(d.ovitraps.23.median,
                   municipality.fac == "Caslano"),
            mapping = aes(y = Y.num,
                          x = X.num,
                          colour = median.females)) +
  geom_point(size = 3) +
  scale_color_gradientn(colours = c("blue", "purple", "red"),
                        values = scales::rescale(c(min.females.median, max.females.median)),
                        limits = c(min.females.median, max.females.median)) +
  # theme(aspect.ratio = 1) +
  coord_fixed() +
  labs(title = "Caslano")
p
```

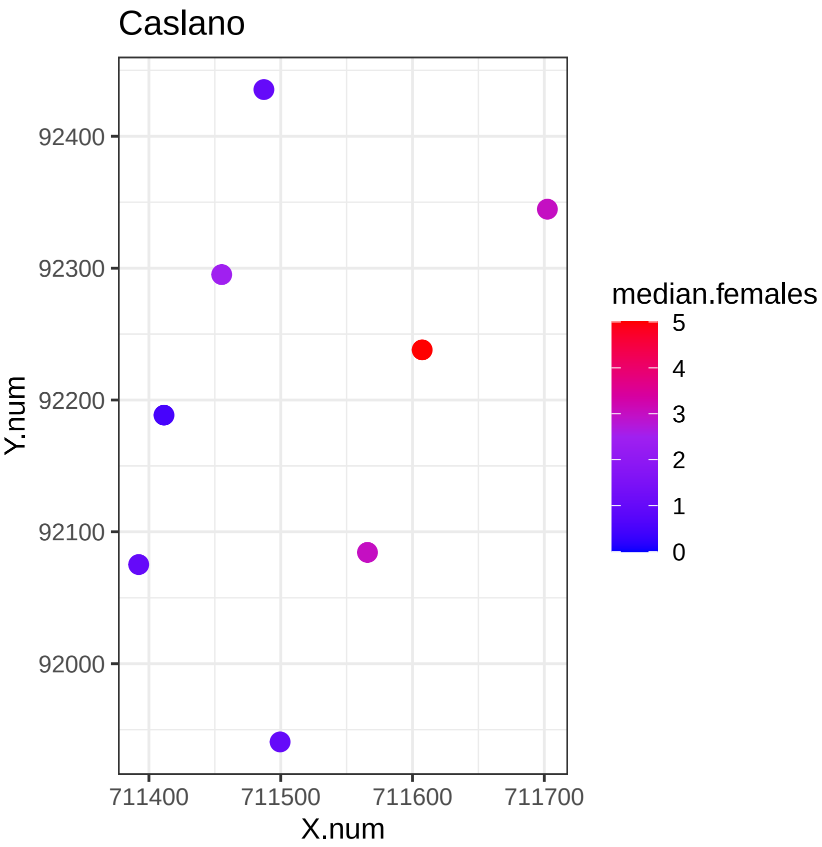


```
## save plot for future use:
saveRDS(p, file = file.path("saved_figures",
                            "2c_meanEgssOverSpaceCaslano_graphForPaper.rds"))
```

There is some variability on the territory of Caslano.

6.2 Fitting the models

We filter out the observations having missing values for the variables of interest.

```
d.ovitraps.23.spatial <- d.ovitraps.23 %>%
  select(`Ae.albopictus.female`,
         municipality.fac,
         ID_BG_trap.fac,
         yday,
         X.num, Y.num) %>%
  na.omit() %>%
  droplevels()
##
## check
unique(d.ovitraps.23.spatial$municipality.fac)
```

```
[1] Caslano Morcote
Levels: Caslano Morcote
```

```
dim(d.ovitraps.23)
```

```
[1] 396  29
```

```r
dim(d.ovitraps.23.spatial)
```

```
[1] 358   6
```

We use a point-constrained for *yday* to the 1st of July.

```r
as.Date("2023-07-01") %>% yday()
```

```
[1] 182
```

Let's fit the model for Morcote.

First, we look how large can be k for the bi-dimensional smoother.

```r
d.ovitraps.23.spatial %>%
  filter(municipality.fac == "Morcote") %>%
  select(X.num, Y.num) %>%
  unique() %>%
  nrow()
```

```
[1] 25
```

Note that we use the element `s(X.num, Y.num)` to plot spatial data. We can use this isotropic smoothing because both predictors are spatial coordinates and we assume the spatial effect is isotropic (i.e., the same in all directions) and the two variables are on the same scale.

```r
## (this chunk is not evaluated)
##
gam.tot.females_space.Morcote.23 <- gamV(Ae.albopictus.female ~
                                           s(yday, pc = pc.23) +
                                           s(X.num, Y.num, k = 25), ## new element!

                                         family = "nb",
                                         data = filter(d.ovitraps.23.spatial,
                                                       municipality.fac == "Morcote"))
##
saveRDS(gam.tot.females_space.Morcote.23,
        file = "Prepared_data_and_models/GAM_tot_females_space_Morcote.23.RDS")
```

Let's get the previously fitted model.

```r
gam.tot.females_space.Morcote.23 <- readRDS(paste0("Prepared_data_and_models/",
                                                   "GAM_tot_females_space_Morcote.23.RDS"))
##
summary(gam.tot.females_space.Morcote.23)
```

```

Family: Negative Binomial(7.117)
Link function: log

Formula:
Ae.albopictus.female ~ s(yday, pc = pc.23) + s(X.num, Y.num,
    k = 25)

Parametric coefficients:
            Estimate Std. Error z value Pr(>|z|)
(Intercept)  -0.7061     0.2053   -3.44 0.000582 ***
---
```

```
Signif. codes:  0 '***' 0.001 '**' 0.01 '*' 0.05 '.' 0.1 ' ' 1

Approximate significance of smooth terms:
                  edf Ref.df Chi.sq  p-value
s(yday)         6.118  7.272  69.28  < 2e-16 ***
s(X.num,Y.num) 17.077 20.522  62.48 3.38e-06 ***
---
Signif. codes:  0 '***' 0.001 '**' 0.01 '*' 0.05 '.' 0.1 ' ' 1

R-sq.(adj) =  0.403   Deviance explained =   46%
-REML = 338.66  Scale est. = 1         n = 264
```

We refit the same model for Caslano. For Caslano we have less unique coordinates, therefore we need to adapt k before fitting the model.

Let's see how large can be k for the bi-dimensional smoother.

```
d.ovitraps.23.spatial %>%
  filter(municipality.fac == "Caslano") %>%
  select(X.num, Y.num) %>%
  unique() %>%
  nrow()
```

```
[1] 8
```

Let's incorporate this information in the model call.

```
gam.tot.females_space.Caslano.23 <- gamV(Ae.albopictus.female ~
                                           s(yday, pc = pc.23) +
                                           s(X.num, Y.num, k = 8),  ## new element!
                                         family = "nb",
                                         data = filter(
                                           d.ovitraps.23.spatial,
                                           municipality.fac == "Caslano")) ## new element!
##
saveRDS(gam.tot.females_space.Caslano.23,
        file = "Prepared_data_and_models/gam.tot.females_space.Caslano.23.RDS")
```

Let's get the previously fitted model.

```
gam.tot.females_space.Caslano.23 <- readRDS(paste0("Prepared_data_and_models/",
                                                   "gam.tot.females_space.Caslano.23.RDS"))
##
summary(gam.tot.females_space.Caslano.23)
```

```
Family: Negative Binomial(3.35)
Link function: log

Formula:
Ae.albopictus.female ~ s(yday, pc = pc.23) + s(X.num, Y.num,
    k = 8)

Parametric coefficients:
            Estimate Std. Error z value Pr(>|z|)
(Intercept) -0.02618    0.21201  -0.123    0.902
```

```
Approximate significance of smooth terms:
                  edf Ref.df Chi.sq  p-value
s(yday)         3.045  3.788  71.44  < 2e-16 ***
s(X.num,Y.num) 4.698  5.768  26.77 0.000145 ***
---
Signif. codes:  0 '***' 0.001 '**' 0.01 '*' 0.05 '.' 0.1 ' ' 1

R-sq.(adj) =  0.526   Deviance explained = 62.4%
-REML = 170.33  Scale est. = 1         n = 94
```

6.3 Plotting the models

6.3.1 Morcote

Let's visualise the results for Morcote.

```
plot.gam(gam.tot.females_space.Morcote.23,
         select = 1,
         trans = exp)
```

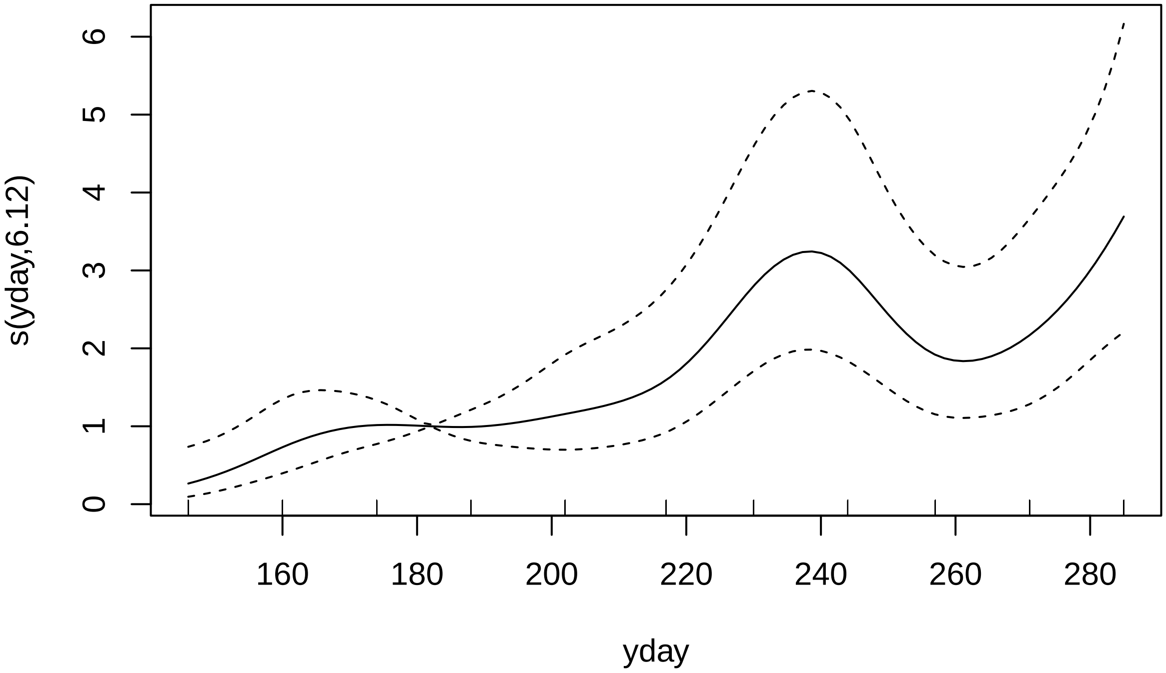


```
##
plot.gamViz(gam.tot.females_space.Morcote.23,
            select = 2,
            trans = exp) +
  coord_fixed()
```

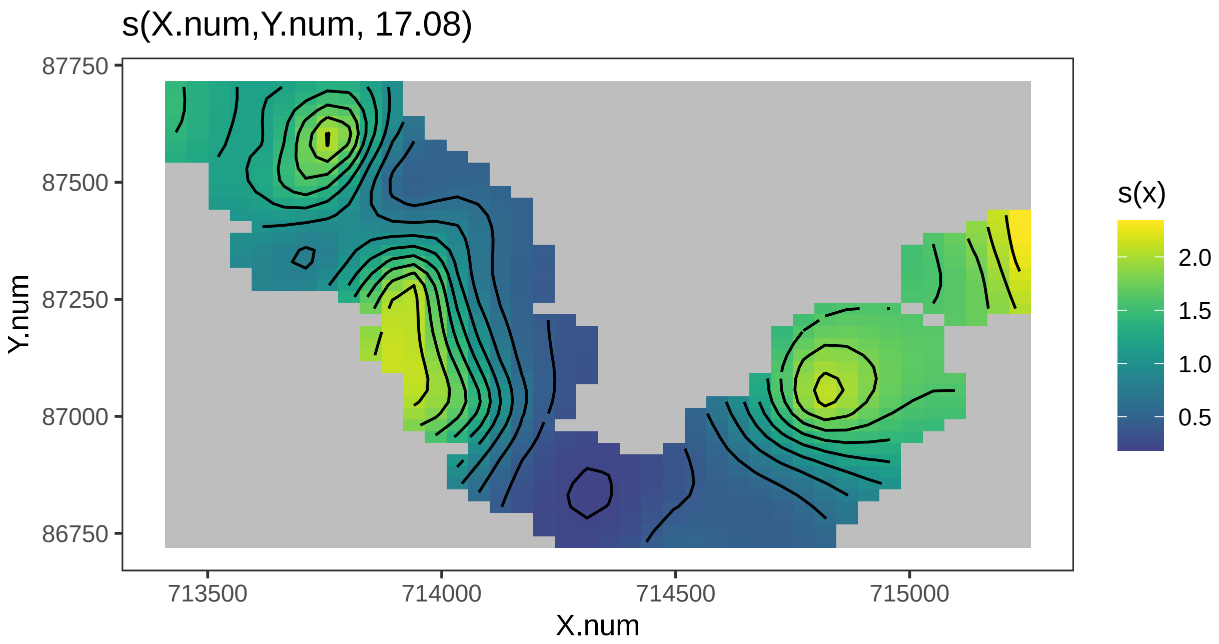

There appear to be some zones with a high number of females, while other areas show very low counts. In fact, looking at the scale, there is a notable variability across the territory (from 0.5 to 2 times the number of females).

6.3.2 Caslano

Let's now visualise the results for Caslano.

```
plot(gam.tot.females_space.Caslano.23,
     select = 1,
     trans = exp)
```

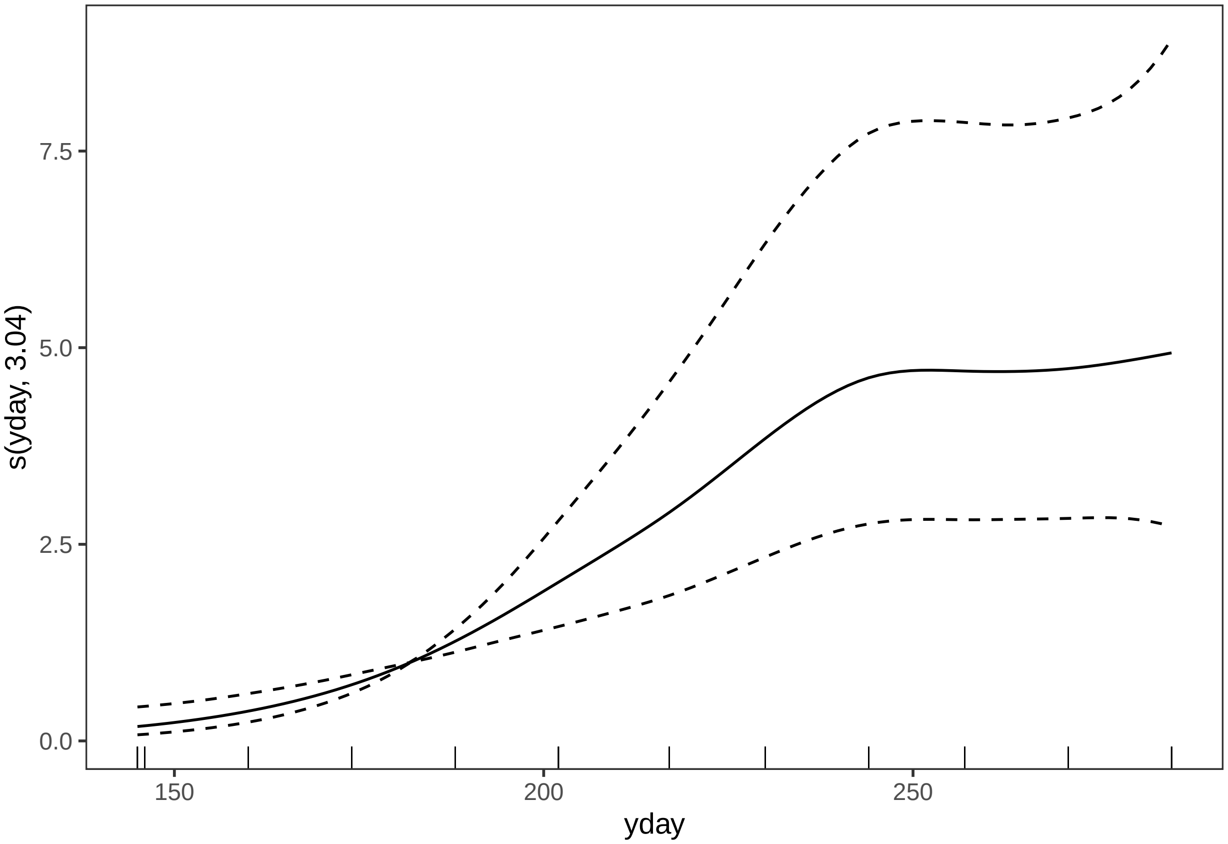


```
##
plot.gamViz(gam.tot.females_space.Caslano.23,
            select = 2,
            trans = exp) +
  coord_fixed()
```

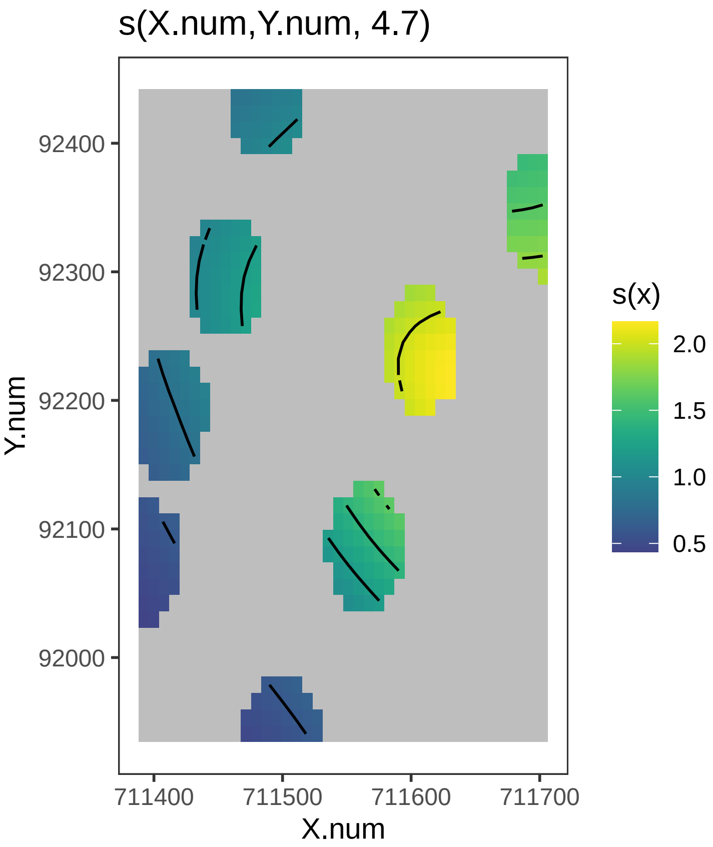

Apparently, the density is not high enough to estimate the surface.

Internal comment: (relevant for SIT design).

Note also that the scale shows that the spatial variation is similar to Morcote.

6.4 Comparing (over)dispersion in the two models

We compare again the overdispersion in the two models.

As a reminder, in a negative binomial model the parameter θ is estimated to be such that:

$$var(y) = \mu + \mu^2/\theta, \quad \text{where} \quad \mu = \mathbb{E}(y)$$

.

Therefore, the lower the parameter value, the higher the overdispersion.

```
gam.tot.females_space.Morcote.23$family$getTheta(TRUE)
```

```
[1] 7.116712
```

```
gam.tot.females_space.Caslano.23$family$getTheta(TRUE)
```

```
[1] 3.349796
```

Overdispersion is higher in Caslano.

7 Spatial Generalised Additive Model over time – Females

We now concentrate on the spatial distribution of females over time. For this reason, we fit a model similar to the previous one, but this time we model the time component as a tensor product together with the coordinates.
We fit two separate models, one for Morcote and one for Caslano

```
d.adults.morcote <- filter(d.ovitraps.23.spatial,
                           municipality.fac %in% c("Morcote"))
d.adults.caslano <- filter(d.ovitraps.23.spatial,
                           municipality.fac %in% c("Caslano"))
##
## Morcote
gam.tot.females_space.Morcote.23_vary <- gamV(
  Ae.albopictus.female ~
    te(X.num, Y.num, yday), ## new element!
  family = "nb",
  data = d.adults.morcote)
summary(gam.tot.females_space.Morcote.23_vary)
```

```
Family: Negative Binomial(2.652)
Link function: log

Formula:
Ae.albopictus.female ~ te(X.num, Y.num, yday)

Parametric coefficients:
            Estimate Std. Error z value Pr(>|z|)
(Intercept) -0.20609    0.08469  -2.433    0.015 *
---
Signif. codes:  0 '***' 0.001 '**' 0.01 '*' 0.05 '.' 0.1 ' ' 1

Approximate significance of smooth terms:
                        edf Ref.df Chi.sq p-value
te(X.num,Y.num,yday) 12.48  15.87  80.54  <2e-16 ***
---
Signif. codes:  0 '***' 0.001 '**' 0.01 '*' 0.05 '.' 0.1 ' ' 1

R-sq.(adj) =  0.266   Deviance explained = 28.1%
-REML =  329.9  Scale est. = 1         n = 264
```

```
##
## Caslano
gam.tot.females_space.Caslano.23_vary <- gamV(
  Ae.albopictus.female ~
    te(X.num, Y.num, yday), ## new element!
  family = "nb",
  data = d.adults.caslano)
summary(gam.tot.females_space.Caslano.23_vary)
```

```
Family: Negative Binomial(5.731)
Link function: log

Formula:
```

```
Ae.albopictus.female ~ te(X.num, Y.num, yday)

Parametric coefficients:
            Estimate Std. Error z value Pr(>|z|)
(Intercept)   0.3252     0.1333    2.44   0.0147 *
---
Signif. codes:  0 '***' 0.001 '**' 0.01 '*' 0.05 '.' 0.1 ' ' 1

Approximate significance of smooth terms:
                       edf Ref.df Chi.sq p-value
te(X.num,Y.num,yday) 17.31     22  119.7  <2e-16 ***
---
Signif. codes:  0 '***' 0.001 '**' 0.01 '*' 0.05 '.' 0.1 ' ' 1

R-sq.(adj) =  0.651   Deviance explained =   72%
-REML = 151.75  Scale est. = 1         n = 94
```

7.1 Plotting the model

We plot the resulting model over time and space.

First for Morcote.

```
## Linear predictor space
plotSlice(sm(gam.tot.females_space.Morcote.23_vary, select = 1),
          fix = list("yday" = quantile(d.adults.morcote$yday,
                                         probs = c(0, 0.2, 0.4, 0.6, 0.8)))) +
  coord_fixed()
```

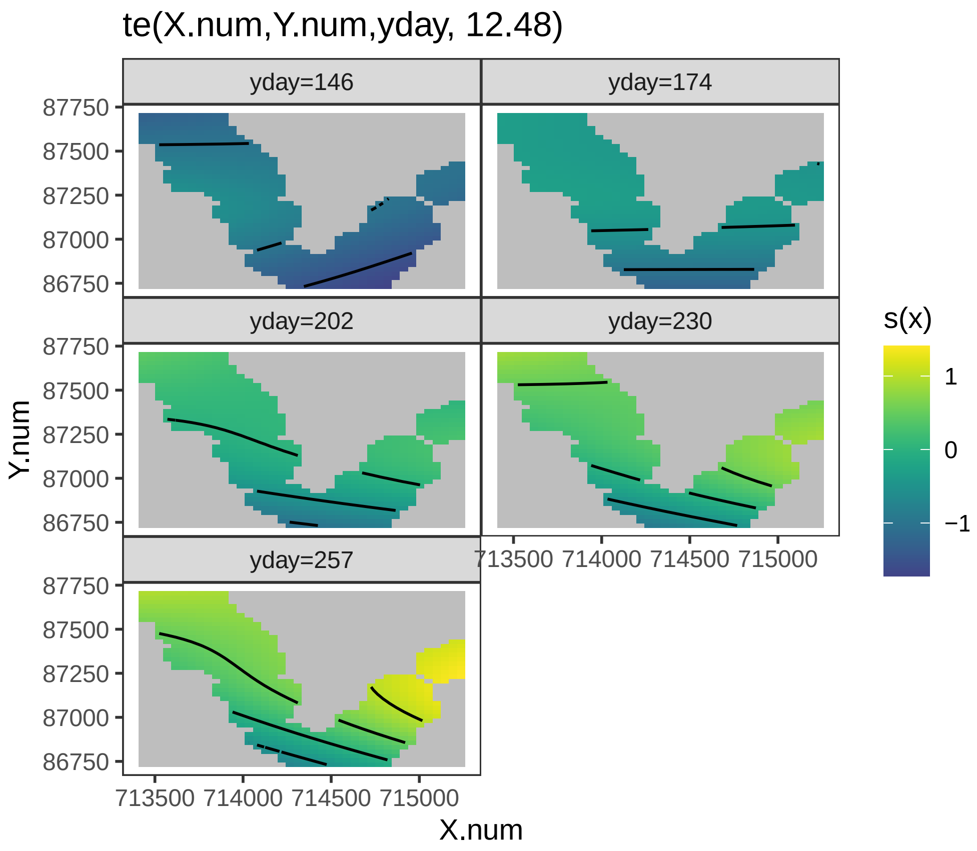

```r
## Back transformed
plotSlice(sm(gam.tot.females_space.Morcote.23_vary, select = 1),
          trans = exp,
          fix = list("yday" = quantile(d.adults.morcote$yday,
                                       probs = c(0, 0.2, 0.4, 0.6, 0.8)))) +
  coord_fixed()
```

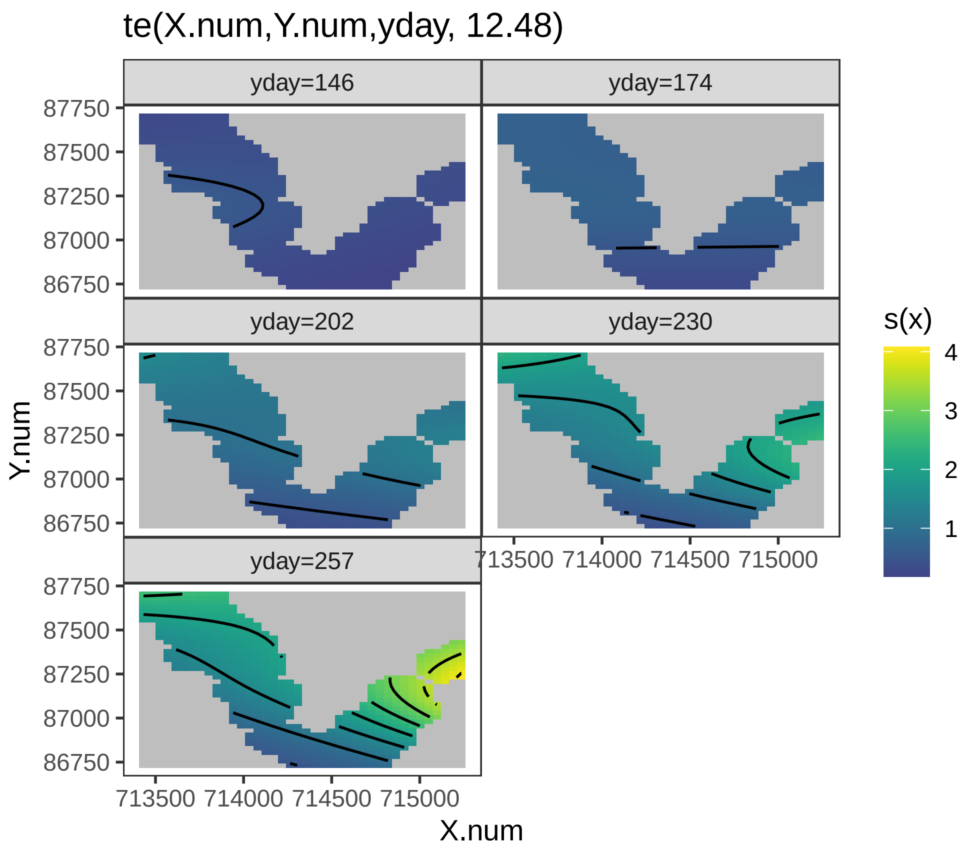

In Morcote, the number of females remained fairly constant throughout the season, with a slight increase only toward the end of the season, and more on the right side of the municipality.

We now plot the model for Caslano.

```r
## (warnings are omitted from this chunk)
##
## Linear predictor space
plotSlice(sm(gam.tot.females_space.Caslano.23_vary, select = 1),
          fix = list("yday" = quantile(d.adults.caslano$yday,
                                       probs = c(0, 0.2, 0.4, 0.6, 0.8)))) +
  coord_fixed()
```

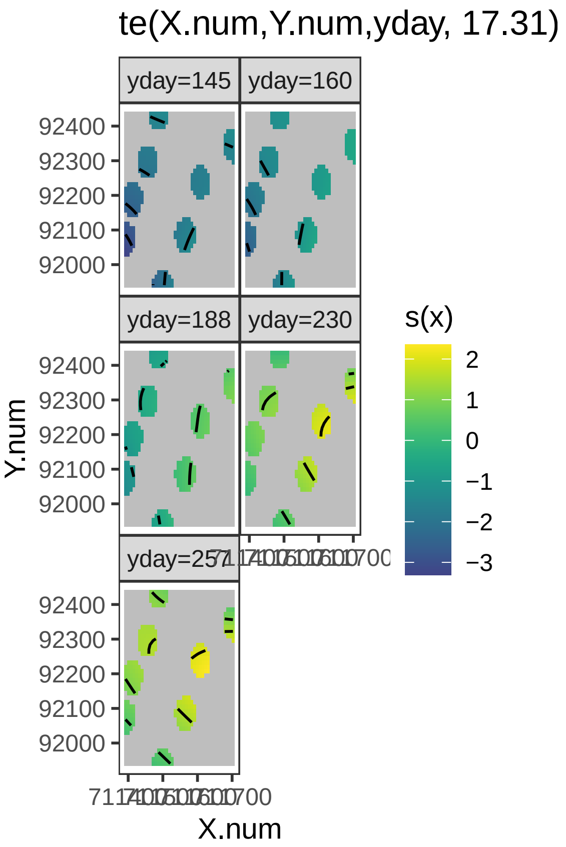


```r
## Back transformed
plotSlice(sm(gam.tot.females_space.Caslano.23_vary, select = 1),
          trans = exp,
          fix = list("yday" = quantile(d.adults.caslano$yday,
                                       probs = c(0, 0.2, 0.4, 0.6, 0.8)))) +
  coord_fixed()
```

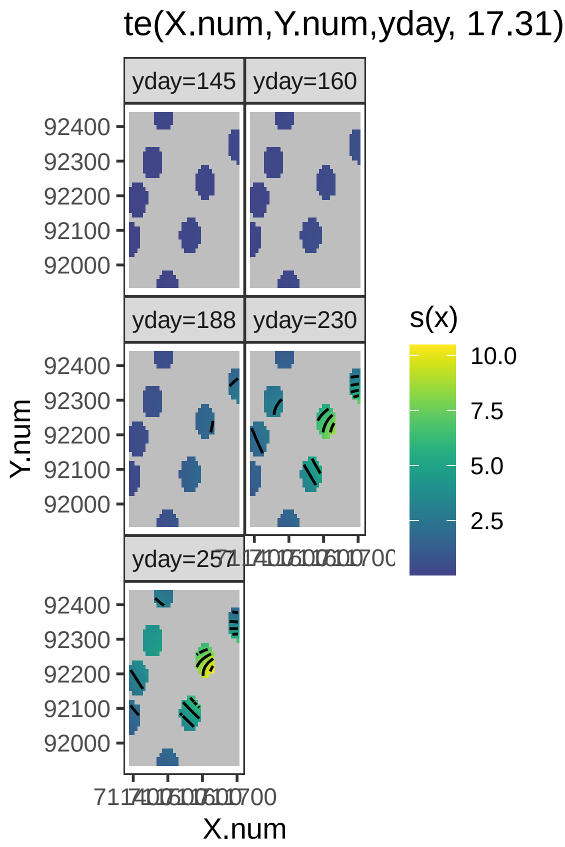

The number of females increases considerably during the season in Caslano, with a greater increase toward the northeastern part of the municipality. It can be seen from the scale in the legend that there are more females in Caslano than in Morcote.

7.2 Comparison between models

We are interested in whether it is necessary to add the temporal component to the spatial model. Therefore, we compare these models using AIC and BIC.

We start by looking at the Morcote models.

```
## Morcote
AIC(gam.tot.females_space.Morcote.23_vary, gam.tot.females_space.Morcote.23)
```

```
                                             df      AIC
gam.tot.females_space.Morcote.23_vary 17.87303 697.8226
gam.tot.females_space.Morcote.23      27.81230 648.6351
```

```
BIC(gam.tot.females_space.Morcote.23_vary, gam.tot.females_space.Morcote.23)
```

```
                                             df      BIC
gam.tot.females_space.Morcote.23_vary 17.87303 761.7357
gam.tot.females_space.Morcote.23      27.81230 748.0905
```

AIC and BIC agree that the more complex model is not necessary.

We now check Caslano.

```
## Caslano
AIC(gam.tot.females_space.Caslano.23_vary, gam.tot.females_space.Caslano.23)
```

```
                                         df      AIC
gam.tot.females_space.Caslano.23_vary 21.67535 336.4691
gam.tot.females_space.Caslano.23      10.97437 339.1817
```

```
BIC(gam.tot.females_space.Caslano.23_vary, gam.tot.females_space.Caslano.23)
```

```
                                         df      BIC
gam.tot.females_space.Caslano.23_vary 21.67535 391.5959
gam.tot.females_space.Caslano.23      10.97437 367.0928
```

AIC and BIC don't agree on the need of the more complex model. In these situations, the simpler model is preferred.

8 Spatial Generalised Additive Model – Males (spatial GAM)

An additional analysis of interest is to examine where there is the highest concentration of mosquito males.

8.1 Visualising the data

Let us first take a look at the median number of males in the traps of Morcote. We calculate the median because we are dealing with skewed data.

```
## (warnings ande messages are omitted from this chunk)
##
d.ovitraps.23.median.males <- d.ovitraps.23 %>%
  group_by(ID_BG_trap.fac, municipality.fac, X.num, Y.num) %>%
  summarise(median.males = median(Ae.albopictus.male, na.rm = TRUE)) %>%
  ungroup()
##
## We calculate the min and max for the median number of eggs.
## This is used in the plots, in fact it allows us
## to use the same colour scale for the two
## municipalities and compare them more easily.
max.males.median <- max(d.ovitraps.23.median.males$median.males)
min.males.median <- min(d.ovitraps.23.median.males$median.males)
##
##
p <- ggplot(filter(d.ovitraps.23.median.males,
                   municipality.fac == "Morcote"),
            mapping = aes(y = Y.num,
                          x = X.num,
                          colour = median.males)) +
  geom_point(size = 3) +
  scale_color_gradientn(colours = c("blue", "purple", "red"),
                        values = scales::rescale(c(min.males.median,
                                                   max.males.median)),
                        limits = c(min.males.median, max.males.median)) +
  # theme(aspect.ratio = 1) +
  coord_fixed() +
  labs(title = "Morcote")
p
```

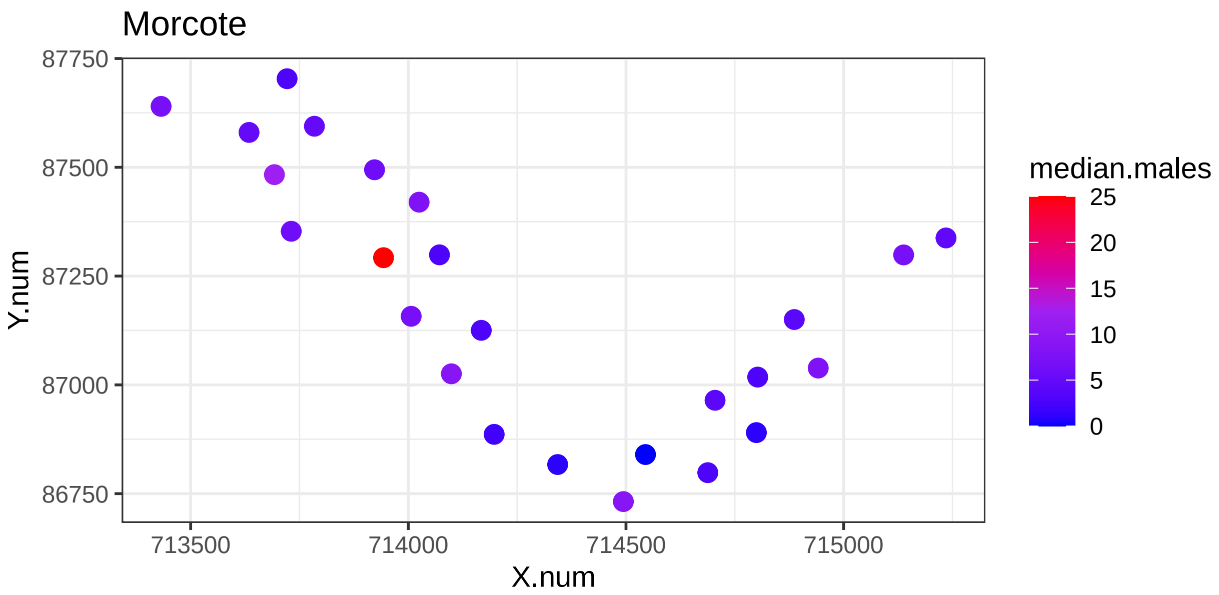


```
## save plot for future use:
saveRDS(p, file = file.path("saved_figures",
                            "2c_meanEgssOverSpaceMorcote_graphForPaper_males.rds"))
```

In general, there doesn't seem to be a strong spatial pattern. However, there is a trap with a high median.

Let's look at Caslano.

```
## (warnings are omitted from this chunk)
##
p <- ggplot(filter(d.ovitraps.23.median.males,
                   municipality.fac == "Caslano"),
            mapping = aes(y = Y.num,
                          x = X.num,
                          colour = median.males)) +
  geom_point(size = 3) +
  scale_color_gradientn(colours = c("blue", "purple", "red"),
                        values = scales::rescale(c(min.males.median, max.males.median)),
                        limits = c(min.males.median, max.males.median)) +
  # theme(aspect.ratio = 1) +
  coord_fixed() +
  labs(title = "Caslano")
p
```

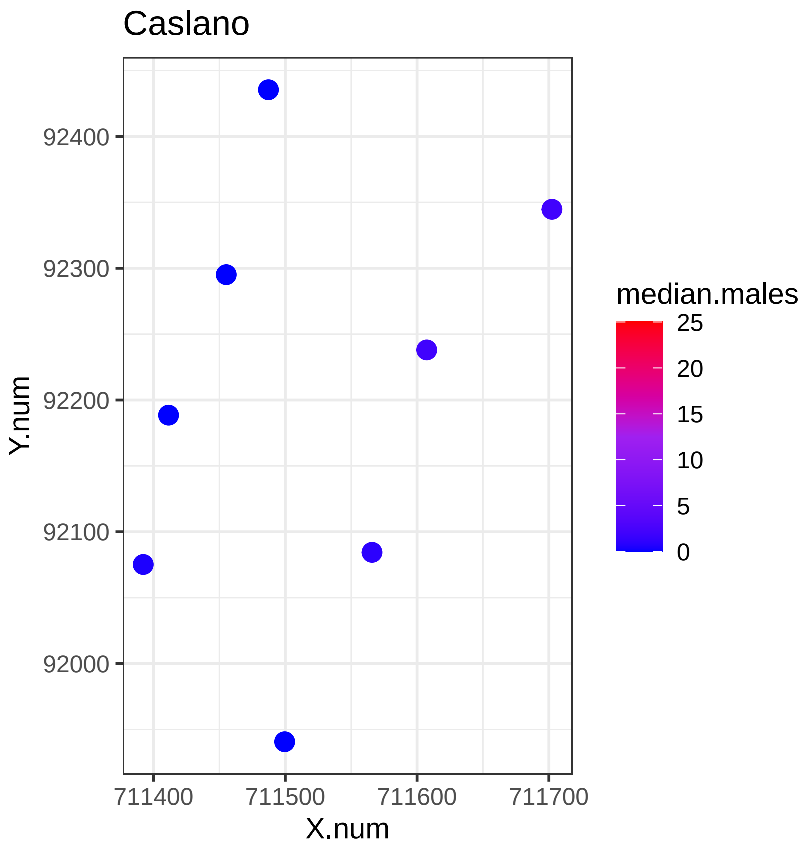


```r
## save plot for future use:
saveRDS(p, file = file.path("saved_figures",
                            "2c_meanEgssOverSpaceCaslano_graphForPaper_males.rds"))
```

The median of males in Caslano seems to be very low, without much variation.

8.2 Fitting the models

We start by removing all the observations having missing values for the variables of interest.

```r
d.ovitraps.23.spatial.males <- d.ovitraps.23 %>%
  select(`Ae.albopictus.male`,
         `Activation.time`,
         municipality.fac,
         ID_BG_trap.fac,
         yday,
         X.num, Y.num) %>%
  na.omit() %>%
  droplevels()
##
## check
unique(d.ovitraps.23.spatial.males$municipality.fac)
```

```
[1] Caslano Morcote
Levels: Caslano Morcote
```

```r
dim(d.ovitraps.23)
```

```
[1] 396  29
```

```r
dim(d.ovitraps.23.spatial.males)
```

```
[1] 358   7
```

We fit two separate models, one for Morcote, one for Caslano, respectively.

We use a point-constrained for *yday* to the 1st of July.

```r
as.Date("2023-07-01") %>% yday()
```

```
[1] 182
```

Let's fit the model for Morcote.

First, we look how large k can be for the bi-dimensional smoother.

```r
d.ovitraps.23.spatial.males %>%
  filter(municipality.fac == "Morcote") %>%
  select(X.num, Y.num) %>%
  unique() %>%
  nrow()
```

```
[1] 25
```

Note that we use the element `s(X.num, Y.num)` to plot spatial data. We can use this isotropic smoothing because both predictors are spatial coordinates and we assume the spatial effect is isotropic (i.e., the same in all directions) and the two variables are on the same scale.

```r
## (this chunk is not evaluated)
##
gam.tot.males_space.Morcote.23 <- gamV(Ae.albopictus.male ~
                                         s(yday, pc = pc.23) +
                                         s(X.num, Y.num, k = 25), ## new element!

                                       family = "nb",
                                       data = filter(d.ovitraps.23.spatial.males,
                                                     municipality.fac == "Morcote"))
##
saveRDS(gam.tot.males_space.Morcote.23,
        file = "Prepared_data_and_models/GAM_tot_males_space_Morcote.23.RDS")
```

Let's get the previously fitted model.

```r
gam.tot.males_space.Morcote.23 <- readRDS(paste0("Prepared_data_and_models/",
                                                 "GAM_tot_males_space_Morcote.23.RDS"))
##
summary(gam.tot.males_space.Morcote.23)
```

```

Family: Negative Binomial(0.728)
Link function: log

Formula:
Ae.albopictus.male ~ s(yday, pc = pc.23) + s(X.num, Y.num, k = 25)

Parametric coefficients:
            Estimate Std. Error z value Pr(>|z|)
```

```
(Intercept)   2.2291     0.1945   11.46   <2e-16 ***
---
Signif. codes:  0 '***' 0.001 '**' 0.01 '*' 0.05 '.' 0.1 ' ' 1

Approximate significance of smooth terms:
                  edf Ref.df Chi.sq p-value
s(yday)         7.049  8.129  77.54  <2e-16 ***
s(X.num,Y.num) 12.150 15.942  32.37  0.0088 **
---
Signif. codes:  0 '***' 0.001 '**' 0.01 '*' 0.05 '.' 0.1 ' ' 1

R-sq.(adj) =  0.151   Deviance explained = 31.4%
-REML = 848.69  Scale est. = 1         n = 264
```

We refit the same model for Caslano. For Caslano we have less unique coordinates, therefore we need to adapt k before fitting the model.

Let's see how large k can be for the bi-dimensional smoother.

```
d.ovitraps.23.spatial %>%
  filter(municipality.fac == "Caslano") %>%
  select(X.num, Y.num) %>%
  unique() %>%
  nrow()
```

```
[1] 8
```

Let's incorporate this information in the model call.

```
gam.tot.males_space.Caslano.23 <- gamV(Ae.albopictus.male ~
                                         s(yday, pc = pc.23) +
                                         s(X.num, Y.num, k = 8),  ## new element!
                                       family = "nb",
                                       data = filter(
                                         d.ovitraps.23.spatial.males,
                                         municipality.fac == "Caslano")) ## new element!
##
saveRDS(gam.tot.males_space.Caslano.23,
        file = "Prepared_data_and_models/gam.tot.males_space.Caslano.23.RDS")
```

Let's get the previously fitted model.

```
gam.tot.males_space.Caslano.23 <- readRDS(paste0("Prepared_data_and_models/",
                                                 "gam.tot.males_space.Caslano.23.RDS"))
##
summary(gam.tot.males_space.Caslano.23)
```

```
Family: Negative Binomial(1.159)
Link function: log

Formula:
Ae.albopictus.male ~ s(yday, pc = pc.23) + s(X.num, Y.num, k = 8)

Parametric coefficients:
            Estimate Std. Error z value Pr(>|z|)
(Intercept)  -1.1186     0.3343  -3.346 0.000819 ***
```

```
---
Signif. codes:  0 '***' 0.001 '**' 0.01 '*' 0.05 '.' 0.1 ' ' 1

Approximate significance of smooth terms:
                  edf Ref.df Chi.sq  p-value
s(yday)         2.580  3.221  32.84 1.31e-07 ***
s(X.num,Y.num) 2.062  2.122  24.95 4.78e-06 ***
---
Signif. codes:  0 '***' 0.001 '**' 0.01 '*' 0.05 '.' 0.1 ' ' 1

R-sq.(adj) =   0.13   Deviance explained = 53.3%
-REML = 122.54  Scale est. = 1         n = 94
```

8.3 Plotting the smoothers

8.3.1 Morcote

Let's visualise the results; we start with Morcote.

```r
plot.gam(gam.tot.males_space.Morcote.23,
         select = 1,
         trans = exp)
```

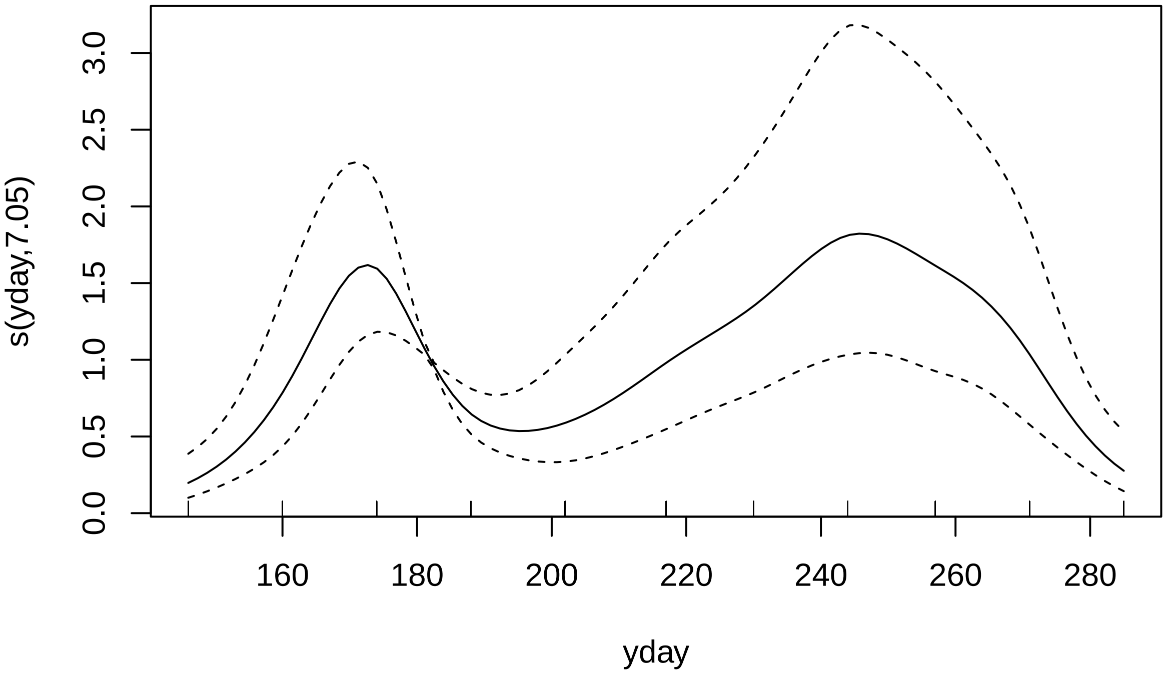


```r
##
plot.gamViz(gam.tot.males_space.Morcote.23,
            select = 2,
            trans = exp) +
  coord_fixed()
```

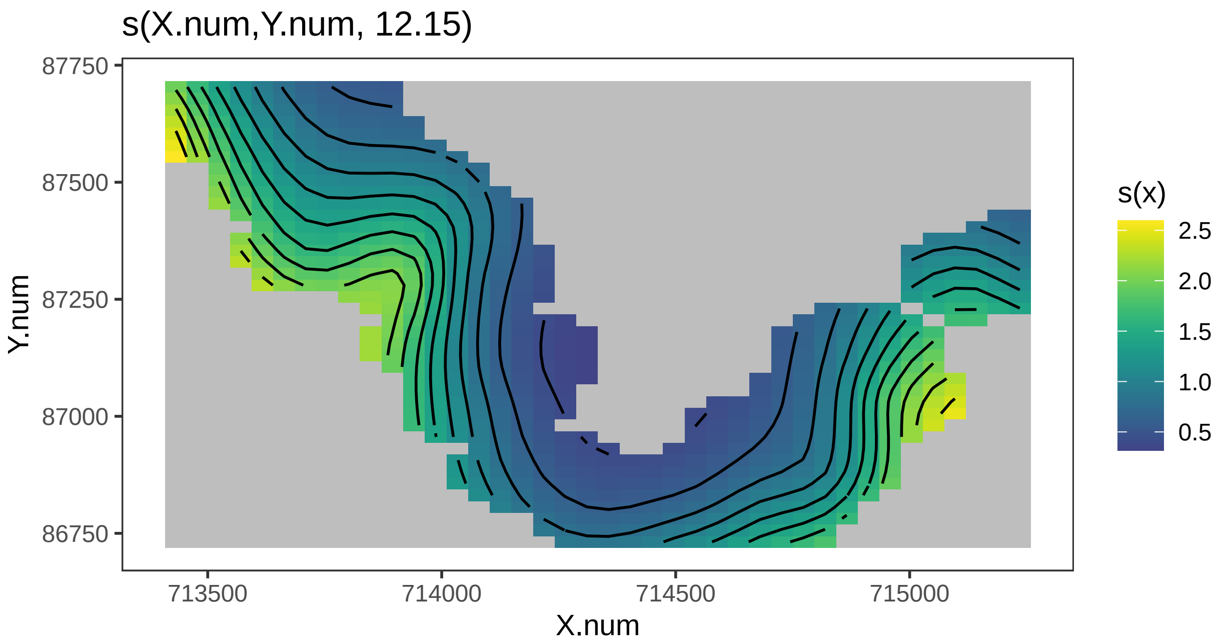

There seems to be a spatial pattern. In fact, there seems to be a higher count of males south. Looking at a map, it is where water is.

8.3.2 Caslano

We now look at the results for Caslano.

```
plot.gam(gam.tot.males_space.Caslano.23,
         select = 1,
         trans = exp)
```

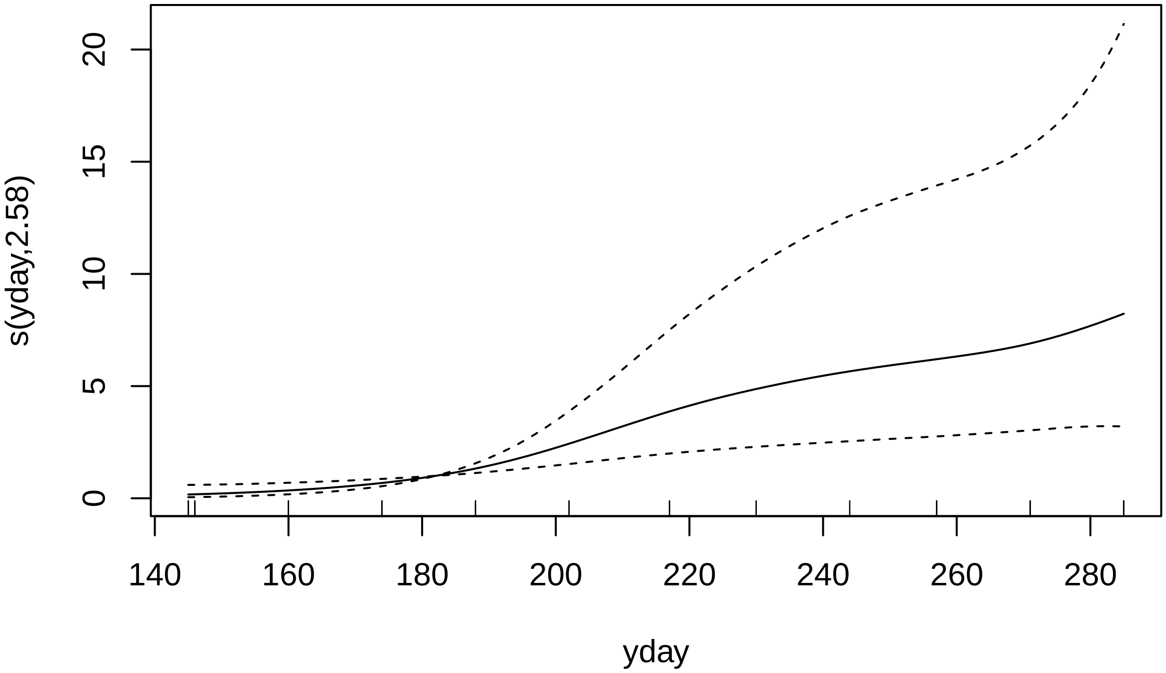


```
##
plot.gamViz(gam.tot.males_space.Caslano.23,
```

```
             select = 2,
             trans = exp) +
  coord_fixed()
```

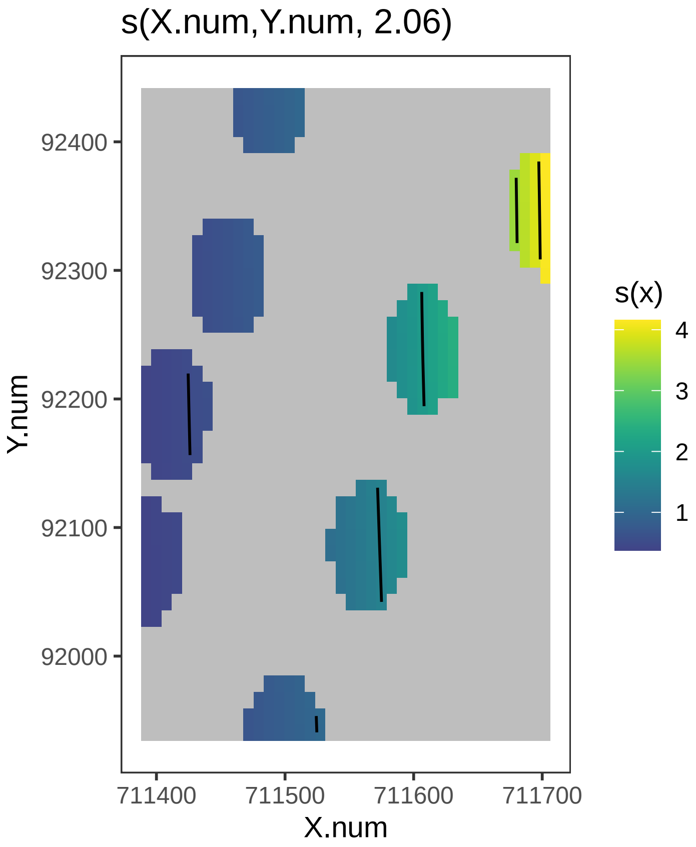

Apparently, the density is not high enough to estimate the surface.

Internal comment: (relevant for SIT design).

This time males seem to prefer going north-east. Looking at the map, it is where water is.

8.4 Comparing (over)dispersion in the two models

We compare again the overdispersion in the two models.

As a reminder, in a negative binomial model the parameter θ is estimated to be such that:

$$var(y) = \mu + \mu^2/\theta, \quad \text{where} \quad \mu = \mathbb{E}(y).$$

Therefore, the lower the parameter value, the higher the overdispersion.

```
gam.tot.males_space.Morcote.23$family$getTheta(TRUE)
```

```
[1] 0.7279496
```

```
gam.tot.males_space.Caslano.23$family$getTheta(TRUE)
```

```
[1] 1.158951
```

Overdispersion is higher in Morcote.

9 Spatial Generalised Additive Model over time – Males

We are interested in the movement of male mosquitoes. For this reason, we fit a model similar to the previous one, but this time we model the time component as a tensor product together with the coordinates.

Since sterile males are released only in Morcote, it makes sense to concentrate on this municipality. However, for completeness we fit the model also for Caslano.

```
## Morcote
d.adults.morcote <- filter(d.ovitraps.23.spatial.males,
                           municipality.fac %in% c("Morcote"))
gam.tot.males_space.Morcote.23_vary <- gamV(
  Ae.albopictus.male ~
    # s(yday) +
    te(X.num, Y.num, yday), ## new element!
  family = "nb",
  data = d.adults.morcote)
summary(gam.tot.males_space.Morcote.23_vary)
```

```

Family: Negative Binomial(0.711)
Link function: log

Formula:
Ae.albopictus.male ~ te(X.num, Y.num, yday)

Parametric coefficients:
            Estimate Std. Error z value Pr(>|z|)
(Intercept)  1.98125    0.07825   25.32   <2e-16 ***
---
Signif. codes:  0 '***' 0.001 '**' 0.01 '*' 0.05 '.' 0.1 ' ' 1

Approximate significance of smooth terms:
                         edf Ref.df Chi.sq p-value
te(X.num,Y.num,yday) 34.69  43.35  125.7  <2e-16 ***
---
Signif. codes:  0 '***' 0.001 '**' 0.01 '*' 0.05 '.' 0.1 ' ' 1

R-sq.(adj) =  0.104   Deviance explained = 34.5%
-REML = 842.83  Scale est. = 1         n = 264
```

```
##
## Caslano
d.adults.caslano <- filter(d.ovitraps.23.spatial.males,
                           municipality.fac %in% c("Caslano"))
gam.tot.males_space.Caslano.23_vary <- gamV(
  Ae.albopictus.male ~
    # s(yday) +
    te(X.num, Y.num, yday), ## new element!
  family = "nb",
  data = d.adults.caslano)
summary(gam.tot.males_space.Caslano.23_vary)
```

```

Family: Negative Binomial(2.258)
Link function: log
```

```
Formula:
Ae.albopictus.male ~ te(X.num, Y.num, yday)

Parametric coefficients:
            Estimate Std. Error z value Pr(>|z|)
(Intercept)  -0.8189     0.2812  -2.912  0.00359 **
---
Signif. codes:  0 '***' 0.001 '**' 0.01 '*' 0.05 '.' 0.1 ' ' 1

Approximate significance of smooth terms:
                       edf Ref.df Chi.sq p-value
te(X.num,Y.num,yday) 16.36  20.32  70.77  <2e-16 ***
---
Signif. codes:  0 '***' 0.001 '**' 0.01 '*' 0.05 '.' 0.1 ' ' 1

R-sq.(adj) =  0.447   Deviance explained = 70.2%
-REML =  102.6  Scale est. = 1         n = 94
```

9.1 Plotting the models

9.1.1 Morcote

We start by looking at Morcote.

```
## (warnings are omitted from this chunk)
##
## linear predictor scale
plotSlice(sm(gam.tot.males_space.Morcote.23_vary, select = 1),
          fix = list("yday" = quantile(d.adults.morcote$yday,
                                       probs = c(0, 0.2, 0.4, 0.6, 0.8)))) +
  coord_fixed()
```

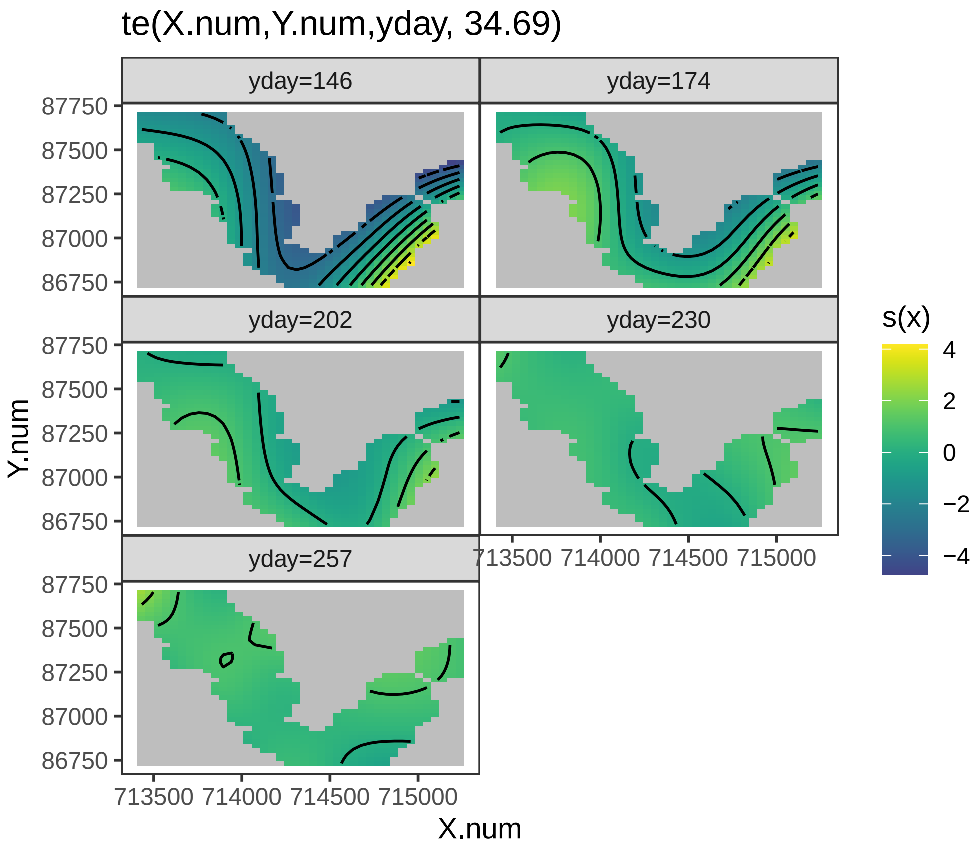


```
##
## back transformed
plotSlice(sm(gam.tot.males_space.Morcote.23_vary, select = 1),
          trans = exp,
          fix = list("yday" = quantile(d.adults.morcote$yday,
                                       probs = c(0, 0.2, 0.4, 0.6, 0.8)))) +
  coord_fixed()
```

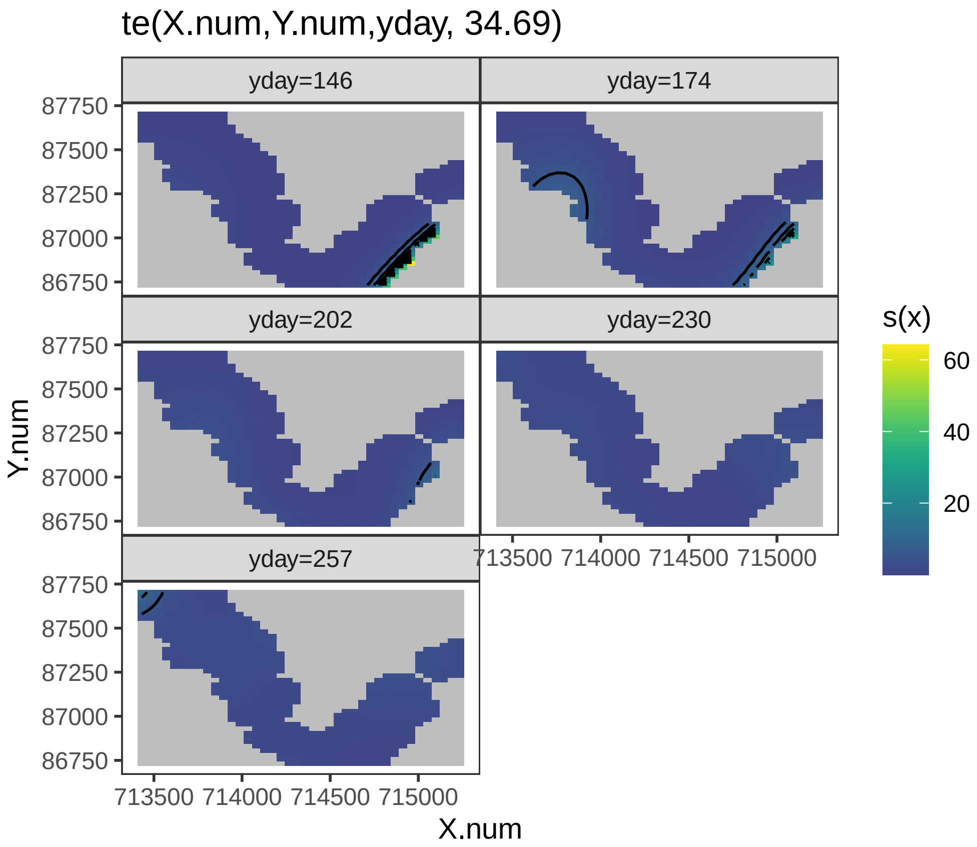

At the beginning of the experiment, a spatial difference appears to be present, with more males concentrated along the lower borders. However, this pattern seems to change over time and eventually disappears.

Note that the first plot is in the linear predictor scale, while the second one was back transformed.

9.1.2 Caslano

We now look at Caslano.

```
## (warnings are omitted from this chunk)
##
## linear predictor scale
plotSlice(sm(gam.tot.males_space.Caslano.23_vary, select = 1),
          fix = list("yday" = quantile(d.adults.caslano$yday,
                                       probs = c(0, 0.2, 0.4, 0.6, 0.8)))) +
  coord_fixed()
```

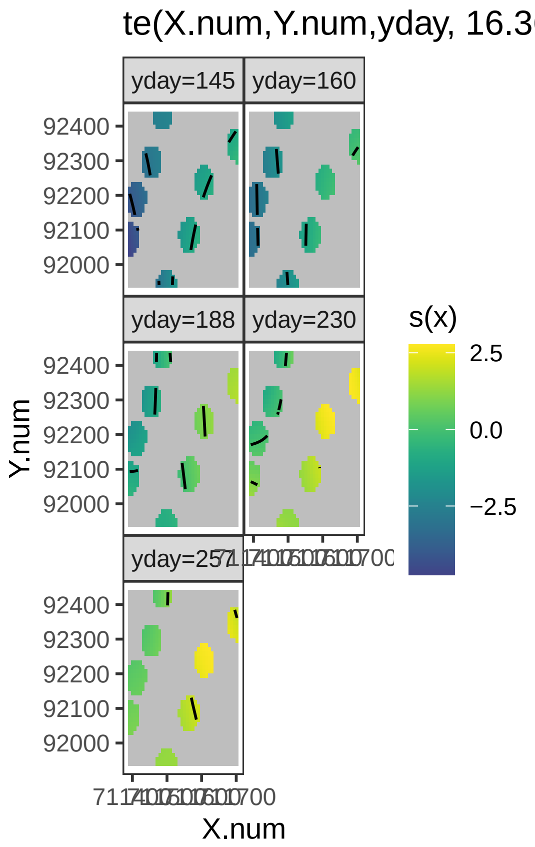


```
##
## back transformed
plotSlice(sm(gam.tot.males_space.Caslano.23_vary, select = 1),
          trans = exp,
          fix = list("yday" = quantile(d.adults.caslano$yday,
                                       probs = c(0, 0.2, 0.4, 0.6, 0.8)))) +
  coord_fixed()
```

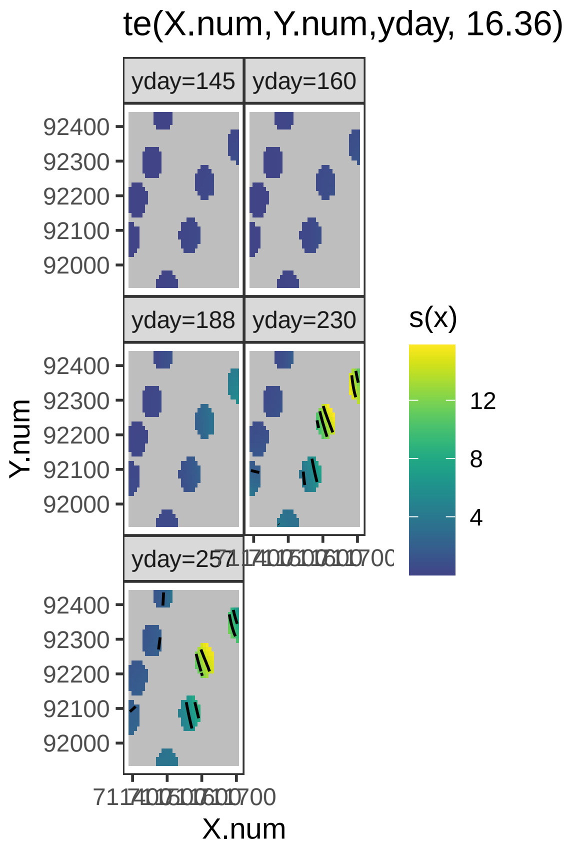

We can observe that the number of mosquitoes increases throughout the season, with a greater concentration in the northeastern part of the municipality. However, the distribution over the territory seems to remain fairly similar throughout the season.

10 Methods

10.1 Generalised Additive Mixed-Effects Model – Females (GAMM)

In the first section, *Generalised Additive Mixed-Effects Model (GAMM)*, we modeled the response variable, "number of Ae. albopictus females" (*Ae.albopictus.female*, a count variable ranging from 0 to 20), using a Generalized Additive Model (GAM) with a negative binomial family to address overdispersion. The model includes a smooth effect for sampling date, represented as the day of the year (*yday*), a numeric variable ranging from 145 to 285 , which interacts with municipality. The municipality variable consists of 2 levels (Caslano, Morcote), and its effect was modeled as a fixed effect.

We further controlled for the non-independence of observations by including *trap ID* (*ID_BG_trap.fac*) as a random effect, with data from 33 distinct traps.
The significance level was set at 5%.

Model complexity was evaluated, and the best-fitting model was selected using AIC and BIC criteria. All statistical analyses were conducted using R, and further details on the modelling approach are provided in the appendix.

10.2 Spatial Generalised Additive Model – Females (spatial GAM)

In the second section, *Spatial Generalised Additive Model (spatial GAM)*, we fitted two separate models to analyse the response variable, "number of Ae. albopictus females" (*Ae.albopictus.female*, a count variable

ranging from 0 to 20), using Generalized Additive Models (GAM) with a negative binomial family to account for overdispersion. One model was fitted for the municipality of Morcote, and the other for Caslano.

Both models included a smooth effect for the sampling date, represented as day of the year (*yday*), and a combined smooth effect for geographic coordinates to capture spatial variability within each municipality. This allowed us to assess the temporal trends and spatial distribution of egg counts separately for Morcote and Caslano.
The significance level was set at 5%.

We selected the best-fitting models by evaluating AIC and BIC criteria. All statistical analyses were conducted using R, and further details on the modeling approach are provided in the appendix.

The same approach was applied to males.

10.3 Spatial Generalised Additive Model – Males (spatial GAM)

The same model as for section *Spatial Generalised Additive Model – females (spatial GAM)* was fitted, only for males.

11 Results

11.1 Generalised Additive Mixed-Effects Model – Females (GAMM)

- The model converged without problems, with a deviance explained of

60.1 %

- different shapes are not necessary
- different intercepts are necessary (pvalue

2.2110596×10^{-4}

)

- The number of mosquitoes in morcote is estimated to be 33.9 % of the number present in Caslano.
- Morcote has lower overdispersion, theta of

7.1167123

compared to Calsano

3.3497961

11.2 Spatial Generalised Additive Model – Females (spatial GAM)

- Model for morcote converged without problems, deviance explained is 31.5%

47.3 %

- Model for Morcote converged without problems.
- Model for caslano converged without problems, deviance explained is

62.3 %

- Model for Caslano converged without problems.

62.3 %

Morcote has more variability (overdispersion parameter: 7.1167123) compared to Calsano (overdispersion parameter: 3.3497961).

Caslano doesn't have much variability in the number of eggs over the territory.

11.3 Spatial Generalised Additive Model – Males (spatial GAM)

- Morcote deviance explained is 31.5%
- Caslano deviance explained is 53.3%
- Model for Morcote converged without problems.

31.4 %

right-hand side of morcote, which is under the influence of non treated municipality Vicomorcote, has almost XXX times higher counts for mosquitoes eggs than the left-hand side of Morcote.

- Model for Caslano converged without problems.

53.3 %

Morcote has more / less variability (overdispersion parameter: 0.7279496) compared to Calsano (overdispersion parameter: 1.1589505).

Caslano doesn't have much variability in the number of eggs over the territory.

12 Conclusions

13 References

```r
citation("mgcViz")
```

```
To cite the mgcViz package in publications use:

  Fasiolo, M., Nedellec, R., Goude, Y. and Wood, S.N., 2020. Scalable
  visualization methods for modern generalized additive models. Journal
  of computational and Graphical Statistics, 29(1), pp.78-86.

A BibTeX entry for LaTeX users is

  @Article{,
    title = {Scalable visualisation methods for modern Generalized Additive Models.},
    journal = {Journal of the Royal Statistical Society (B)},
    volume = {29},
    number = {1},
    pages = {78-86},
    year = {2020},
    author = {{Fasiolo} and {Matteo} and {Nedellec} and {Rapha{"e}l} and {Goude} and {Yannig} and {Wood
  }

As mgcViz is often updated, you may want to cite its version number.
Find it with 'help(package=mgcViz)'.
```

```r
citation("lubridate")
```

```
To cite lubridate in publications use:

  Garrett Grolemund, Hadley Wickham (2011). Dates and Times Made Easy
  with lubridate. Journal of Statistical Software, 40(3), 1-25. URL
  https://www.jstatsoft.org/v40/i03/.

A BibTeX entry for LaTeX users is
```

```
@Article{,
  title = {Dates and Times Made Easy with {lubridate}},
  author = {Garrett Grolemund and Hadley Wickham},
  journal = {Journal of Statistical Software},
  year = {2011},
  volume = {40},
  number = {3},
  pages = {1--25},
  url = {https://www.jstatsoft.org/v40/i03/},
}
```

14 Appendix
